# Comparison of methods to handle missing values in a continuous index test in a diagnostic accuracy study – a simulation study

**DOI:** 10.1186/s12874-025-02594-2

**Published:** 2025-05-27

**Authors:** Katharina Stahlmann, Bastiaan Kellerhuis, Johannes B. Reitsma, Nandini Dendukuri, Antonia Zapf

**Affiliations:** 1 Institute of Medical Biometry and Epidemiology, University Medical Center Hamburg Eppendorf, Hamburg, Germany; 2 Julius Center for Health Sciences and Primary Care, University Medical Center Utrecht, Utrecht University, Utrecht, The Netherlands; 3 Blavatnik School of Government, University of Oxford, Oxford, UK; 4 Department of Medicine, McGill University, Montreal, Canada

**Keywords:** Missing values, Diagnostic study, Index test, AUC, Simulation study, Neutral comparison study

## Abstract

**Background:**

Most diagnostic accuracy studies have applied a complete case analysis (CCA) or single imputation approach to address missing values in the index test, which may lead to biased results. Therefore, this simulation study aims to compare the performance of different methods in estimating the AUC of a continuous index test with missing values in a single-test diagnostic accuracy study.

**Methods:**

We simulated data for a reference standard, continuous index test, and three covariates using different sample sizes, prevalences of the target condition, correlations between index test and covariates, and true AUCs. Subsequently, missing values were induced for the continuous index test, assuming varying proportions of missing values and missingness mechanisms. Seven methods (multiple imputation (MI), empirical likelihood, and inverse probability weighting approaches) were compared to a CCA in terms of their performance to estimate the AUC given missing values in the index test.

**Results:**

Under missing completely at random (MCAR) and many missing values, CCA gives good results for a small sample size and all methods perform well for a large sample size. If missing values are missing at random (MAR), all methods are severely biased if the sample size and prevalence are small. An augmented inverse probability weighting method and standard MI methods perform well with higher prevalence and larger sample size, respectively. Most methods give biased results if missing values are missing not at random (MNAR) and the correlation or the sample size and prevalence are low. Methods using the covariates improve with increasing correlation.

**Conclusions:**

Most methods perform well if the proportion of missing values is small. Given a higher proportion of missing values and MCAR, we would recommend to conduct a CCA and standard MI methods for a small and large sample size, respectively. In the absence of better alternatives we recommend to conduct a CCA and to discuss its limitations, if the sample size is small, and missing values are M(N)AR. Standard MI methods and the augmented inverse probability approach may be a good alternative, if the sample size and/or correlation increases. All methods are biased under MNAR and a low correlation.

**Supplementary Information:**

The online version contains supplementary material available at 10.1186/s12874-025-02594-2.

## Background

The key purpose of a diagnostic accuracy study is to determine how well a newly developed test commonly referred to as the index test performs in a certain target population. Accuracy describes the ability of a test to differentiate between individuals with and without the target condition. To estimate accuracy, the index test results are compared to the outcome of a reference standard, which is defined as the “the best available method for establishing the presence or absence of the target condition” [1], and sometimes additionally to a comparator test [2]. If the reference test cannot perfectly identify the presence or absence of the target condition, it can be called an imperfect reference standard [3]. Parameters of accuracy for an index test producing a binary result include sensitivity and specificity, defined as the probability of observing a positive index test result for an individual with the target condition and the probability of observing a negative index test result for an individual without the target condition, respectively. For continuous tests, accuracy can be expressed as the area under the receiver operating characteristic (ROC) curve (AUC) [4].

Biased estimates for accuracy can have detrimental implications for clinical practice [1]. For instance, overestimating the accuracy of a test may lead to misdiagnosis and incorrect treatment of patients. One serious threat to the validity of accuracy estimates are missing values or inconclusive results [1]. Missing values and inconclusive results can occur in the reference standard, index test, comparator test, or in other available test results or characteristics of study participants. There are many different reasons for missing values, including patient refusal, an invasive reference standard – which should not be applied to all patients (leading to partial or differential verification) –, failure of study equipment, or other interfering events [5, 6]. Thus, different patterns of missing values can co-exist in a study, and, depending on the reason for missingness, missing values can be missing completely at random (MCAR), missing at random (MAR), or missing not at random (MNAR) [7]. In practice, loss of a sample or missing data entry may lead to MCAR. MAR may be the result of limited quality of ultrasound of abdominal organs in subjects with obesity (if the information about weight status is available). Questionnaires for the diagnosis of mental illnesses can contain MNAR if severely impaired subjects refuse to answer certain questions about their symptoms or behavior.

To prevent biased results, they must be addressed adequately in the analysis [1]. A detailed overview of possible biases some of which may be related to missing values and inconclusive results can be found in Kohn et al. [8].

Previous reviews have already described a range of strategies to handle missing values in the reference standard or how to address an imperfect reference standard [3, 9, 10]. These strategies can be classified mainly into imputation, bias correction, differential verification, constructing a composite reference standard, using latent class analysis, or employing an expert panel [10]. Several diagnostic studies have already applied those methods in their analysis. In addition, we recently identified several methods that can be applied to handle missing values or inconclusive results in an index test or in both a reference test and an index test [11]. These strategies encompass single and multiple imputation, frequentist and Bayesian likelihood approaches, model-based methods, and latent class analysis. These methods have hardly been used in practice, in contrast to the methods for missing values in the reference standard. Consequently, many diagnostic studies commonly exclude missing values in the analysis or employ naive methods (e.g. single imputation) [12, 13] and, thus, are at risk of producing biased estimates for accuracy estimates.

Possible reasons for neglecting complex methods may include the lack of available analysis code, Shiny apps, or R packages which would facilitate their implementation. Furthermore, it may be difficult to find these methodological articles owing to a lack of clear keywords or a concise title [11].

Most of these methods were only proposed in so-called phase I and phase II methodological studies [14]. That is to say, the authors presented their newly developed method and compared it with one or two competing methods in a small set of scenarios. Despite the necessity of such studies in developing new methods, they also tend to be over-optimistic in favor of the newly proposed method [14, 15]. As a result, there is a definite need for a phase III (and later on for a phase IV) study which compares multiple methods in a wide range of settings and is designed as a neutral comparison study [14]. While our previous review has already provided a structured overview of available strategies to handle missing values and inconclusive results in the index test, a systematic evaluation of these methods under different scenarios – following a neutral comparison design – is still lacking. This may be one reason why well-founded recommendations for choosing a method when facing missing values in the index test of a diagnostic study are missing. A phase III/neutral comparison study will provide a more objective and comprehensive comparison of the identified methods and help other researchers to make better-informed decisions about using an adequate method for their specific diagnostic study [15, 16].

Therefore, this simulation study aims to compare the performance of different methods for estimating the AUC of an index test producing a continuous test result in a diagnostic study when different patterns of missing index test results are present. To cover a range of different situations, we simulated a large set of scenarios and, additionally, applied the methods to a modified case study [16].

The paper is structured as follows. In Sect."Methods", we explain the methodology of our simulation study following the ADEMP framework [17] and the case study. Subsequently, the results of the simulation and case study are presented in Sect."Results"and discussed in Sect."Discussion". Finally, our conclusions and recommendations are summarized in Sect."Conclusion".

## Methods

Our simulation study is designed as a phase III/neutral comparison study following the recommendations by Heinze et al. [14], Strobl and Leisch [16], and Boulestix et al. [15]. Thus, the focus was on comparing the identified methods comprehensively, and not on developing or improving a (new) method [15]. Furthermore, none of this paper’s authors was involved in the development of one of the compared methods and, therefore, they are as neutral as possible.

### Aims of the simulation study

This simulation study aims to compare a range of methods for handling missing values in an index test producing a continuous result with respect to their bias and precision in estimating the AUC in the context of a single-test diagnostic study. Based on the simulation results, recommendations will be derived on which method is most adequate in which scenario.

### Data generation mechanism

The simulated data were mimicking a diagnostic accuracy study evaluating a single index test producing continuous test results in a population of interest suspected for a target condition. This can be considered a classical, cross-sectional diagnostic accuracy study in which we have an error-free reference standard applied in all study participants and a number of additional participant characteristics reflecting demographic, clinical information or other test results (from here on referred to as covariates). The number of observations for both reference standard groups were calculated by n*p for the sample with target condition present (and n–n*p for the sample with target condition absent), in which n is the total sample size and p the prevalence as defined in the simulation parameters. The following parametric models were used to simulate the data. Continuous index test values (x1), as well as three continuous covariates (x2, x3, x4), were simulated separately for both reference standard groups (target condition present or absent) according to the following multivariate normal distribution:

$$\begin{pmatrix}x_1\\x_2\\x_3\\x_4\end{pmatrix}\sim N\left[\begin{pmatrix}\mu\\0\\5\\35\end{pmatrix},\begin{pmatrix}1&r&r&r\\r&1&r&r\\r&r&1&r\\r&r&r&1\end{pmatrix}\right]$$

µ was set to 0 for the non-diseased sample and to ${\Phi }^{-1}\left({AUC}_{true}\right)*\sqrt{2}$ for the diseased sample. The R package mvtnorm [18] was used to model the index test and covariates under the multivariate normal distribution. The covariate ${x}_{2}$ was afterwards dichotomized at its mean (µ = 0). Finally, specific patterns of missing values were generated in the index test values with the function ampute of the mice package [19]. For this procedure, we defined the mechanism of missing values (MCAR, MAR, or MNAR), the proportion of missing values (pm) and the pattern of missing values (missing values only in the index test values). The different missingness mechanisms are simulated in the ampute function accordingly: All values (in the index test in our study) have the same probability of being amputed under MCAR. In sum, as many values are amputed as defined in the proportion of missing values. In contrast, a weighted sum score of all variables in the dataset is constructed to model the probability of being missing under MAR and MNAR. In case of MAR, all covariates have a weight of 1 and the index test has a weight of 0. Therefore, the probability of being missing is dependent on the available covariates. Under MNAR, all covariates have a weight of 0 and only the index test has a weight of 1. Thus, the probability of being missing is only dependent on the index test variable itself. For details we refer to the documentation of the mice package [19] and Schouten et al. [20]. The following table presents the parameters, which were varied factorially resulting into 729 scenarios. To restrict computing time to a feasible limit, each scenario was repeated over 1,000 iterations.

Some methods could only be investigated in a small set of supplemental scenarios (reasons are explained below for the methods concerned). Set up and results of these supplemental scenarios are presented in Additional file 1. In our main analysis, we used the default argument “type = RIGHT” in the ampute function for generating the MNAR scenarios. Thus, higher index test values are more likely to be missing. To explore another MNAR mechanism, we examined the “type = MID” mechanism in which mid-range (medium) values are more likely to be missing. These results are provided in the supplementary Additional file 2.

### Estimand

The AUC and its confidence interval (CI) were calculated as the main estimand by each method. For the estimation of the CI, the significance level was set to α = 0.05 two-sided.

### Description of the methods

**Fig. 1:**
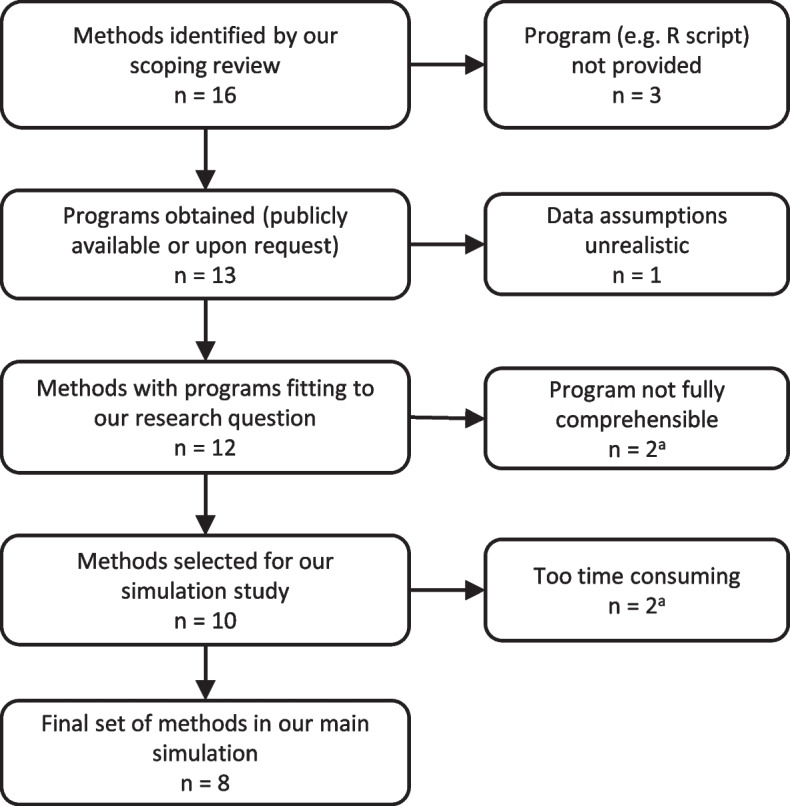
Flow chart of the method selection process. ^a^These four methods are examined in a reduced set of scenarios and repetitions

Based on the review of Stahlmann et al. [11], 16 methods were initially selected that estimate the AUC given missing values in the index test. These methods can be classified into three main approaches, namely multiple imputation [19, 21–23], empirical likelihood [24–29], inverse probability (propensity and prediction score) based approaches [30–32] and the traditional complete case analysis (CCA). Nonetheless, this classification is not very clear as some methods bear similarities across the identified categories. Most of them estimate the AUC including its confidence intervals. Some also estimate sensitivities at different false positive rates (FPR) in order to draw the ROC. Unfortunately, not all authors (*n* = 3 [27–29]) provide their programming script publicly or upon request and, thus, cannot be included in our simulation study. Furthermore, Lin et al. [32] assume that missing values only occur in the index test of diseased subjects, which does not fit our research question (missing values in the index test of both diseased and non-diseased subjects). There were also two methods (IPL-LG and IPL-NP, described below and in Additional file 1) proposed by Cheng and Tang [26] whose program was not fully comprehensible. In addition, one method (CONV, described below and in Additional file 1) by Bianco et al. [31] was too time-consuming, and one multiple imputation method (R package mi [21]) could not be executed on the high-performance cluster. Therefore, we excluded them from our main simulation study but examined them in a small set of scenarios (Additional file 1). Finally, we compared eight methods (described shortly below and in detail in Additional file 4) across the scenarios specified in Table 1: mice, mix, MI2, MIB2, AIPW, KER, HDEL, CCA. The Flow Chart in Fig. 1 shows the selection process of the methods and Table 2 provides an overview of the methods’ technical aspects.

**Table 1:** Fully factorial design (3 x 3 x 3 x 3 x 3 x 3)

	**1,000 simulation runs per scenario**
Total sample size (*N*)	100; 500; 1,000
Prevalence of target condition (*p*)	10%; 30%; 50%
True AUC (*AUC*_*true*_)	0.7; 0.85; 0.9
Correlation between index test and covariates (*r*)	0.2; 0.5; 0.9
Missingness pattern	MCAR; MAR; MNAR
Proportion of missing index test values (*pm*)	10%; 30%; 50%
	**All combinations result in 729 scenarios**

**Table 2:** Overview of the methods’ technical aspects

**Method**	**Description**	**Output**	**Advantages**	**Limitations**
CCA	Complete case analysis	AUC + CIs ROC + CIs	Easy to implement; fast	
*Multiple imputation*				
MI2 [23]	k-nearest neighbor MI using propensity and prediction score	AUC + CIs ROC + CIs	Easy to understand	Only available as R code
MIB2 [23]	MI2 method including a bootstrap step	AUC + CIs ROC + CIs	Easy to understand	Only available as R code
Mice [19]	Multivariate imputation by chained equations (fully conditional specification)	AUC + CIs ROC + CIs	Missing values in the reference standard can also be handled; implemented as R package	May take some time
Mix [22]	Joint modelling	AUC + CIs ROC + CIs	Missing values in the reference standard can also be handled; implemented as R package	May take some time
Mi [21]	Multivariate imputation by chained equations (fully conditional specification)	AUC + CIs ROC + CIs	Missing values in the reference standard can also be handled; implemented as R package	Results are only reproducible if the argument Parallel is set to false; May take some time
*Empirical likelihood*				
HDEL [24, 25]	Hot deck imputation with empirical likelihood	AUC + CIs ROC + CIs		For very low and very high true positive values, no CI boundaries can be found; only available as R code; only for MCAR
IPL-LG [26]	Hybrid imputation- using empirical likelihood parametric	AUC + CIs ROC + CIs		Takes 30 min to run per analysis; only available as R code; only one covariate is selected
IPL-NP [26]	Hybrid imputation- using empirical likelihood non-parametric	AUC + CIs ROC + CIs		Takes 30 min to run per analysis; only available as R code; only one covariate is selected
*IPW and prediction*				
AIPW [30]	Augmented inverse probability weighting using a propensity and prediction score	AUC + CIs	Easy to understand	Only available as R code, results in missing values for the AUC in some scenarios
CONV [31]	Convolution based approach	AUC + CIs ROC		Only available as R code, takes ca. 15 min to run per analysis
KER [31]	Kernel-based approach	AUC + CIs ROC		Only available as R code

We used available R packages for three MI methods (mice, mix and mi) and for the CCA. All other methods are only available as written R code. We will describe the methods shortly: The R package mice [19] employs multivariate imputation by chained equations based on fully conditional specification. Predictive mean matching (PMM) was selected as imputation method in the main simulation study and Bayesian linear regression was additionally examined as imputation method in supplemental simulation scenarios. Similarly, the R package mi by Su et al. [21] employs fully conditional specification. To be consistent, PMM was selected as imputation method, too. In contrast to the previous packages, the mix package by Schafer [22] employs joint modeling under an unrestricted general location model for mixed data. All variables (index test, the three covariates and the reference standard) without interaction terms were included in the imputation models of each standard MI approach and 20 imputed datasets were generated. The methods MI2 and MIB2 by Long et al. [23] follow a nonparametric multiple imputation approach. K-nearest neighbors are identified based on a propensity (the index test being missing yes vs. no as outcome) and prediction score (the index test as outcome) using all covariates. Stratified by target condition, missing values are subsequently multiply imputed (10 imputed datasets) with a random draw of one of the k nearest neighbors. MIB2 conducts one bootstrap step (one random draw of the observed data with replacement) before the estimation of the propensity and prediction score. For all MI methods, we used the pROC package [33] with the auc() function for estimating the AUC by the trapezoidal rule. Under application of Rubin’s rules [34] we calculated the CI based on DeLong et al. [35]. Long et al. [30] proposed a doubly robust augmented inverse probability method – AIPW – based on a propensity and prediction score. The AUC is a weighted average of both scores and its CI is computed using 200 bootstrap samples. Another inverse probability weighting approach is the kernel-based method KER by Bianco et al. [31]. The AUC is calculated as the mean of all sensitivity values for each false positive value between 0 and 1. The CI is calculated following Hanley and McNeil [36]. Both AIPW and KER use all three covariates for calculating the propensity/prediction scores. HDEL by Wang and Qin [24, 25] is the only method – apart from CCA – which does not use covariate information. It estimates the non-parametric AUC based on the Wilcoxon-Mann–Whitney two-sample rank statistic [36] by using hot deck imputation and an empirical likelihood based confidence interval assuming the missing values to be MCAR. As described above, IPL-LG, IPL-NP and CONV were only examined within supplemental scenarios. IPL-LG and IPL-NP [26] are smoothed empirical likelihood methods combining multiple imputation and inverse probability-weighted imputation (i.e. hybrid imputation based on estimating equations). They employ a parametric and non-parametric propensity function, respectively, using all covariates. Both aim to estimate sensitivity values and their CI for defined FPR values. We further extended their code to estimate the AUC and its CI by using the sensitivity values as described for KER. CONV [31] is a convolution-based estimator assuming that the covariates are related to the index test values through a parametric linear regression model. The AUC and its CI are calculated following KER. All methods are compared to CCA which excludes all subjects with missing values in the index test (or reference standard) from the calculations. We used the ci.auc function of the pROC package [33] for its calculation. It estimates the AUC following the trapezoidal rule and the CI according to DeLong et al. [35]. The formulas for AUC estimation by each method are provided in Additional file 4. As recommended by Boulestix et al. [15], the method parameters are set to the standard practice rules or to the default parameters as described in the original papers.

### Performance measures

The performance measures calculated in our simulation study included bias, root mean squared error (RMSE), coverage probability, power, and each method’s running time. The coverage probability is calculated as the percentage of repetitions in which the two-sided 95% confidence intervals cover the true value. The power is calculated as the percentage of repetitions in which the null hypothesis is rejected at the two-sided significance level of 5% given the null hypothesis is false. The statistical hypotheses were defined as follows:

$${\mathrm H}_0:\mathrm{AUC}\;\leq\;{\mathrm{AUC}}_{\min}\;\mathrm{versus}\;{\mathrm H}_{1}:\;\mathrm{AUC} > {\mathrm{AUC}}_{\min}$$

The AUC_min_ was calculated for each scenario using the sample size formula by Obuchowski et [37] (p. 1123, 2):

$${\mathrm{AUC}}_{\min} = \;-\;\left(\sqrt{\frac{\left(z_{\alpha}\sqrt{0.0792\;\cdot\;\left(1+^{1\!\!}/_\text{k}\right)+z_{\beta}\sqrt{\mathrm V\left(\mathrm\theta\right)}}\right)^2}{{\mathrm n}_{\mathrm D}}-\theta}\right)$$

where θ, n_D,_ and k are the AUC_true_, the number of diseased subjects, and the ratio of non-diseased to diseased subjects (based on the prevalence p) to the number of diseased subjects as defined in the respective scenario; z_α_ and z_β_ are the upper αth and βth percentile of the standard normal distribution. In this simulation study, α was set to 0.05 and β to 0.2 as we assumed a theoretical power of 80%. The variance function of V(θ) is described by $\mathrm V\left(\mathrm\theta\right)=\left(0.0099\cdot\mathrm e^{-\mathrm A^{2}/2}\right)\cdot\left(\left(5A^{\mathit2}+8\right)+\left(\mathrm A^2+8\right)/\mathrm k\right)$ where $\mathrm A=\mathrm\phi^{-1}\left(\mathrm\theta\right)\cdot1.414$ [37] (p. 1123, 3).

In addition, the respective Monte Carlo Standard errors (MCE) were computed to give a measure of simulation uncertainty.

### Metamodels

Following van Smeden et al. [38], we used metamodels to quantify the influence of the simulation parameters on the performance measures. Two different approaches were performed. In the first approach, linear regression models were conducted stratified by missingness mechanism for each method separately using all other simulation parameters as covariates (sample size (N), prevalence of the target condition (p), correlation between index test and covariates (r), true AUC (AUC_0), and proportion of missing values (pm)). Then, all two-way interaction terms between covariates were included in the model and backward elimination was conducted. In the second approach, mixed linear regression models were conducted stratified by missingness mechanism including all methods as covariates and scenario as random intercept. In a next step, the simulation parameters and interaction terms between methods and simulation parameters were included as additional covariates. In line with the first approach, backward elimination was performed. The following performance measure were examined as outcome in separate models: bias (as absolute values), and RMSE. To obtain interpretable regression estimates, the outcome variable was multiplied by 100.

### Case study

As Heinze et al. [14] called for realistic comparative example data analyses in phase III studies and Strobl and Leisch [16] argued that simulated data are only realistic to a limited extent, we decided to apply the methods additionally to a modified case study. The complete dataset of this case study was systematically modified with respect to inserting missing values following MCAR, MAR and MNAR resulting in three alternative case study datasets. This has the advantage that we can compare the results obtained in these three alternative case study datasets with the results from the complete case study dataset.

Data of the case study stem from the Identification of Neonatal Hearing Impairment study and were obtained from the Diagnostic and Biomarkers Statistical (DABS) Center [39]. Norton et al. [40] provide a detailed description of this study about neonatal hearing impairment, its data and results. They aimed to compare three index tests (transient evoked otoacoustic emissions, distortion product otoacoustic emissions (DPOAEs) and auditory brain stem responses) for diagnosing neonatal hearing impairment in 353 well babies at risk of hearing impairment, 80 healthy babies and 4478 babies recently discharged from the intensive care unit. Behavioral threshold measurements were conducted when the babies were between 8 and 12 months and served as reference standard. Specifically, minimum response levels < 30 db HL indicated normal hearing and ≥ 30 db HL for at least one stimulus was defined as impaired hearing. However, not all newborns returned for the behavioral examinations at follow-up. Although the case study dataset had no missing values in the reference standard (or index tests), we assume that this loss to follow-up may have resulted in incomplete records. We do not know whether this publicly available dataset has been prepared in terms of missing data in the main variables beforehand. Further details on this case study were reported by Widen et al. [41].

As the methods in our simulation study are designed for a single test design, we restricted the case study data to only data from the left ear and the DPOAE 65 at 2 kHz as an index test. We also excluded two subjects with missing values in the variable age, which is used as a covariate. The variables site (i.e. hospital where the newborn babies were assessed) and gender were selected as the other covariates (which are needed for some methods). Using the ampute function of the mice package [19], we inserted 30% of missing values in this index test following MCAR (scenario 1), MAR (scenario 2), and MNAR (scenario 3). For each scenario, we calculated the AUC and its CI by the described methods and compared them with the CCA based on the complete case study data (without any missing values). Except for the AIPW method, all other methods can model the ROC curve (i.e. they provide sensitivity and specificity or false positive rate pairs). Thus, we included the comparison of the ROC curves in the case study example as well.

### Software

As some of the above methods need much time to complete, it was not possible to simulate all scenarios in one R script on one local machine. Thus, we divided the simulation study into several scripts and executed them on a high-performance cluster (operated by the University of Hamburg) running on CentOS 7 Linux using R (version 4.1.0). The simulated data were generated using the R package mvtnorm [18], and the R package doFuture [42] was used for parallelization. The analysis of the simulated data and case study was performed using R (version 4.4.1) on a local machine. The simulated data and R programs for the simulation study and case study are provided at GitHub (latest version: 10.5281/zenodo.15279454).

## Results

The findings of our simulation study and case study are presented below. The figures visualize the results of selected scenarios. Results of the supplemental scenarios of the additional methods, MNAR scenarios and of the case study are summarized in Additional files 1 to 3. Furthermore, Additional files 4 and 5 provide additional tables and figures on selected scenarios of the main simulations study, and numeric results for each main scenario in an Excel sheet, respectively.

### Results of the simulation study

It is noteworthy, that not all methods can estimate an AUC in every scenario. CCA and AIPW result in a failed result in 3,182 and 30,676 of 729,000 iterations, respectively. Table 3 shows the scenarios in which most failed results occur. The other methods are not displayed as they did not fail to estimate the AUC in any iteration. For both methods, this is particularly the case if the sample size is small (*N* = 100), the prevalence of the target condition is low (*p* = 0.1), and the proportion of missing values is high (pm = 0.3 or 0.5).

**Table 3:** Overview of the sum of failed AUC estimates by each method^a^ across iterations and scenarios. Only combinations with failed AUC results in at least one method are displayed

**Sample size**	**Prevalence of the target condition**	**Proportion of missing values**	**CCA**^a^	**AIPW**^a^
1,000	0.1	0.5	0 (0%)	5 (0.02%)
500	0.1	0.3	0 (0%)	3 (0.01%)
		0.5	0 (0%)	322 (1.19%)
100	0.1	0.1	0 (0%)	649 (2.4%)
		0.3	299 (1.11%)	9411 (34.86%)
		0.5	2880 (10.67%)	19680 (72.89%)
	0.3	0.3	0 (0%)	7 (0.03%)
		0.5	3 (0.01%)	581 (2.15%)
	0.5	0.5	0 (0%)	18 (0.07%)

^a^Only CCA and AIPW are displayed, as none of the other methods failed to estimate the AUC in an iteration

Comparing all scenarios in a nested loop plot (Additional file 4 Figures 30 and 31), provides an overall picture of the performance regarding bias. Generally, all methods are more biased under MAR and MNAR than under MCAR. While MI2 and MIB2 tend to have a positive bias – especially under MCAR –, the other methods rather underestimate the true AUC. Most methods are more biased if the sample size is small and prevalence of the target condition is low.

### Missing completely at random

Overall, CCA and HDEL provide the least biased results for MCAR across all scenarios, especially if the sample size is small (unless the true AUC and prevalence of the target condition are small and the proportion of missing values is high). In case of many missing values, a low prevalence of the target condition (*p* = 0.1), and small sample size (*N* = 100), mice, mix and MIB2 are the most biased methods, with AIPW and MI following (Additional file 4 Figures 1 and 7). This is also true if the prevalence of the target condition is high with the exception that KER is more biased and AIPW less (Additional file 4 Figures 2 and 7). If the sample size is larger (*N* = 500, Additional file 4 Figure 8), nearly all methods perform well under MCAR except for MI2 and MIB2, which overestimate the true AUC slightly.

Regarding RMSE (Additional file 4 Figures 13 and 14), we see pronounced differences in the methods’ performance by varying sample sizes and prevalence of the target condition. While HDEL shows the highest RMSE across all scenarios, KER has a high RMSE if the sample size and the true AUC are small. In all other scenarios, KER shows a medium or low RMSE. Whereas AIPW has a high RMSE if the sample size is small, prevalence of the target condition, and the correlation between index test and covariates are low, its RMSE improves with higher prevalence and higher correlation between index test and covariates. CCA, MI2, and MIB2 perform quite mediocre across most scenarios. However, MI2 and MIB2 improve with larger sample size and higher correlation, while CCA aggravates. The methods with the lowest RMSE are mix for a low correlation and mice, AIPW, and mix for a high correlation. If the prevalence of the target condition increases, all methods are closer together if the correlation is low.

All methods show a close to 95% coverage (Additional file 4 Figures 19 and 20) regardless of sample size if the prevalence of the target condition is high and the proportion of missing values is low. If the prevalence and the proportion of missing values are high, KER and MI2 have a coverage considerably lower (ca. 80–85%) than the other methods (ca. 90%), especially with a low correlation. If the prevalence of the target condition and the proportion of missing values are low, the performance of most methods lies between 85 and 95% except for KER, HDEL, and MIB2, which show a coverage above 95%. With an increasing proportion of missing values and low prevalence of the target condition, performance decreases severely for KER, MI2, MIB2, CCA, and partly mice – particularly under a small sample size.

Concerning the power (Additional file 4 Figure 25), HDEL and MIB2 differ from the other methods. While HDEL has the highest power if the sample size is small, the prevalence of the target condition, and the true AUC are low, its performance decreases sharply with increasing prevalence of the target condition and higher true AUC, regardless of sample size. MIB2 and CCA – in case of higher correlation – also have a low power whereas KER and MI2 perform well across scenarios. Regardless of sample size, the power of AIPW, mix, and mice decreases sharply with a higher proportion of missing values if the correlation is low.

In summary, CCA and HDEL provide the most valid estimates for a small sample size while all methods perform well with a large sample size.

### Missing at random

**Fig. 2:**
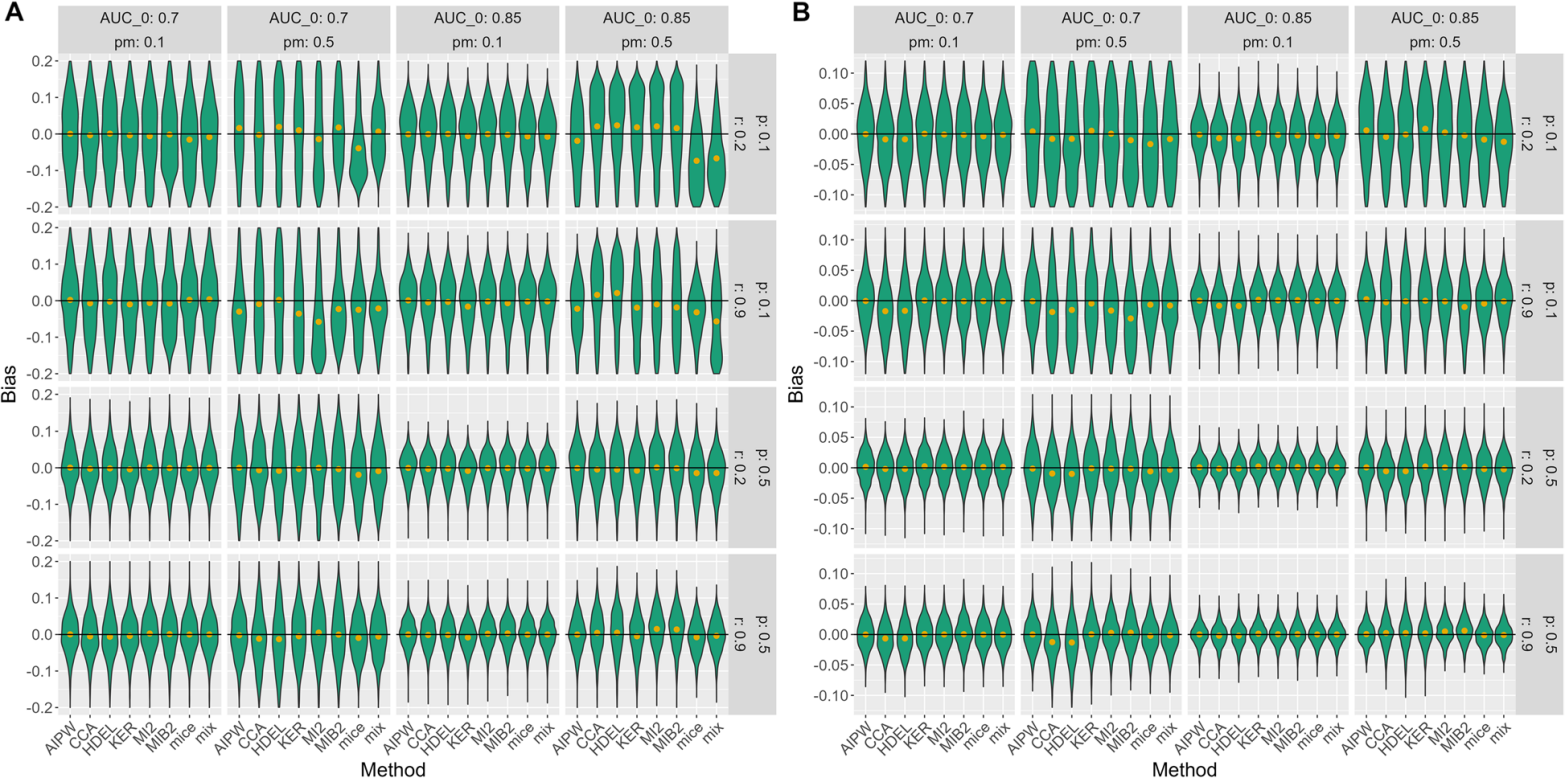
Bias under MAR and a sample size of **A** 100, **B** 500. Pm: proportion of missing values, r: correlation between index test and covariates, AUC_0: true AUC, p: prevalence of the target condition

If the sample size and prevalence of the target condition are small, all methods give biased results for data with many missing values under MAR, especially mice and mix. All methods provide better estimates for a high prevalence of the target condition. Still, mice and mix are more biased than the other methods if the correlation is small, while MI2, MIB2 and, to a lesser extent, CCA and HDEL are more biased with a higher correlation. If sample size increases, estimates of all methods improve except for CCA, HDEL, MI2, and MIB2 which over- or underestimate the AUC if there are many missing values while AIPW performs best in this case. Mice and mix also improve for a larger sample size, especially if the correlation is high (Fig. 2). Nonetheless, they still provide biased results if the prevalence is low.

**Fig. 3:**
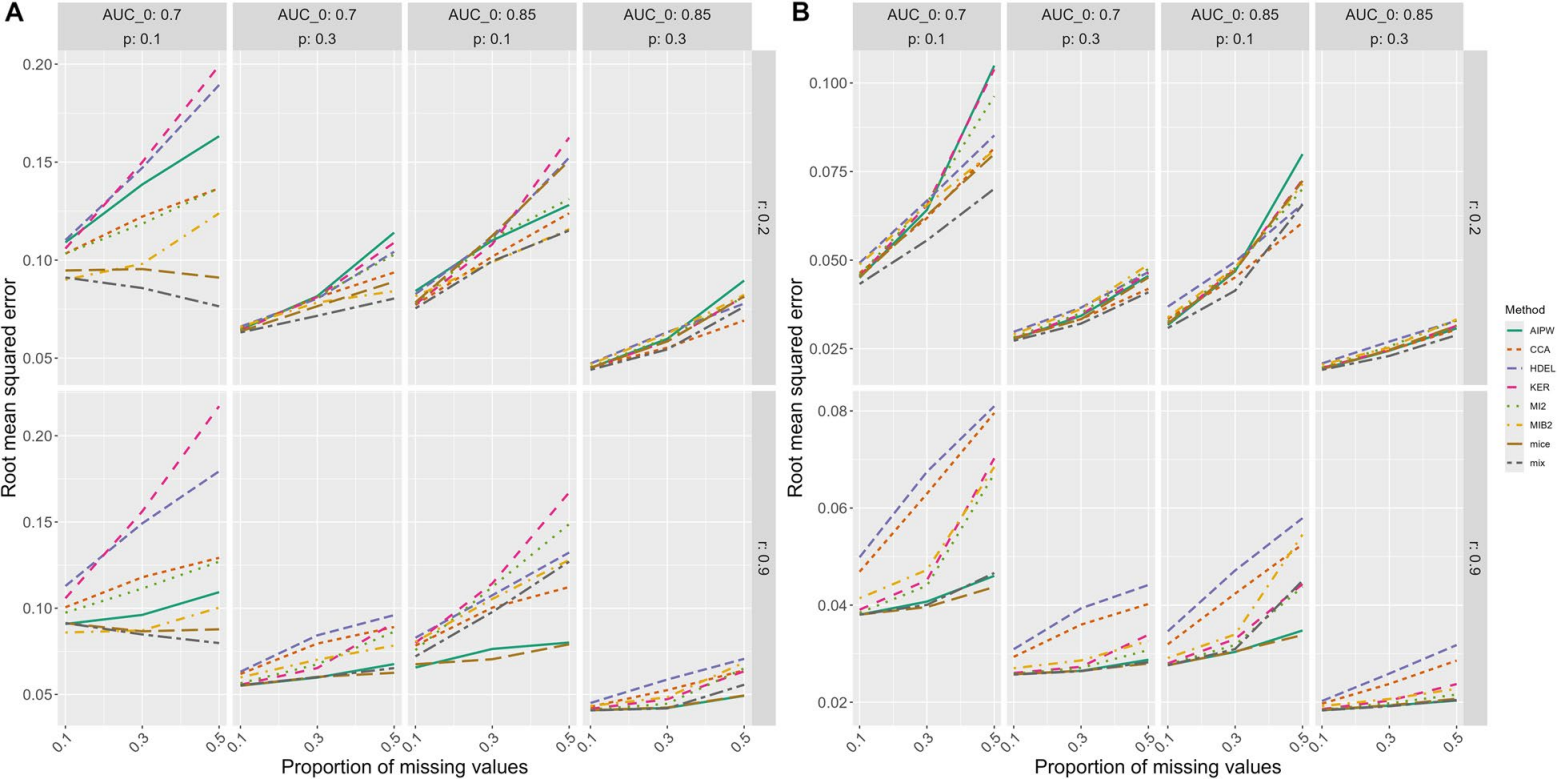
RMSE under MAR and a sample size of **A** 100, **B** 500. Pm: proportion of missing values, r: correlation between index test and covariates, AUC_0: true AUC, p: prevalence of the target condition

Generally, the RMSE increases with an increasing proportion of missing values and is higher for MAR and MNAR compared to MCAR. The pattern between the methods, however, is similar across MCAR (Additional file 4 Figures 13 and 14), MAR (Fig. 3), and MNAR (Additional file 4 Figures 17 and 18). Similar to MCAR, HDEL has a high RMSE overall. However, the RMSE of KER exceeds HDEL if the prevalence of the target condition is low, and the sample size is small and if the prevalence and correlation between index test and covariates are low and the sample size is large. AIPW has also a high RMSE if the prevalence and correlation are low regardless of sample size and if the correlation and sample size are small but the prevalence increases. The RMSE of AIPW is low if the correlation increases regardless of sample size. The remaining methods (CCA, MI2, MIB2, mice and mix) perform similar as under MCAR. Overall, mix has the lowest RMSE if the correlation is low and mice, mix and AIPW if the correlation is high regardless of sample size.

**Fig. 4:**
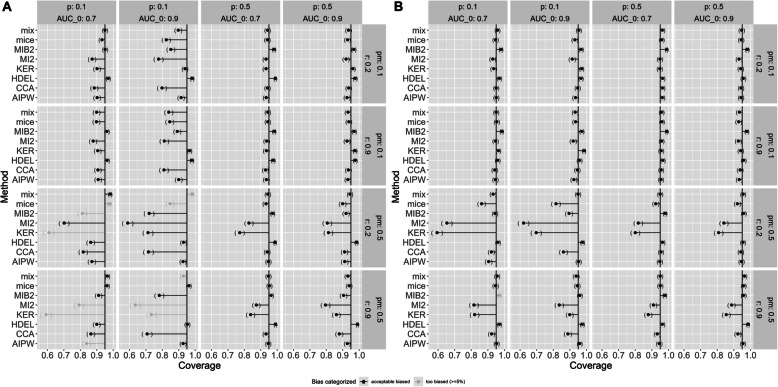
Coverage under MAR and a sample size of **A** 100, **B** 500. Pm: proportion of missing values, r: correlation between index test and covariates, AUC_0: true AUC, p: prevalence of the target condition

Similar to MCAR, most methods show a coverage close to 90% or higher under MAR if the prevalence of the target condition is high except for KER and MI2 which have a considerably lower coverage with more missing values. The same is true for a low prevalence and a large sample size. In case of low prevalence of the target condition, and small sample size, the performance of most methods falls below or close to 90%. With an increasing proportion of missing values and low prevalence of the target condition, performance decreases severely for KER, MI2, MIB2, CCA, and partly mice. However, this may be due to biased estimates if the sample size is small (methods with a bias ≥ 5% are marked in grey in the Figures). Figure 4 shows the coverage probability for a small and large sample size, respectively.

**Fig. 5:**
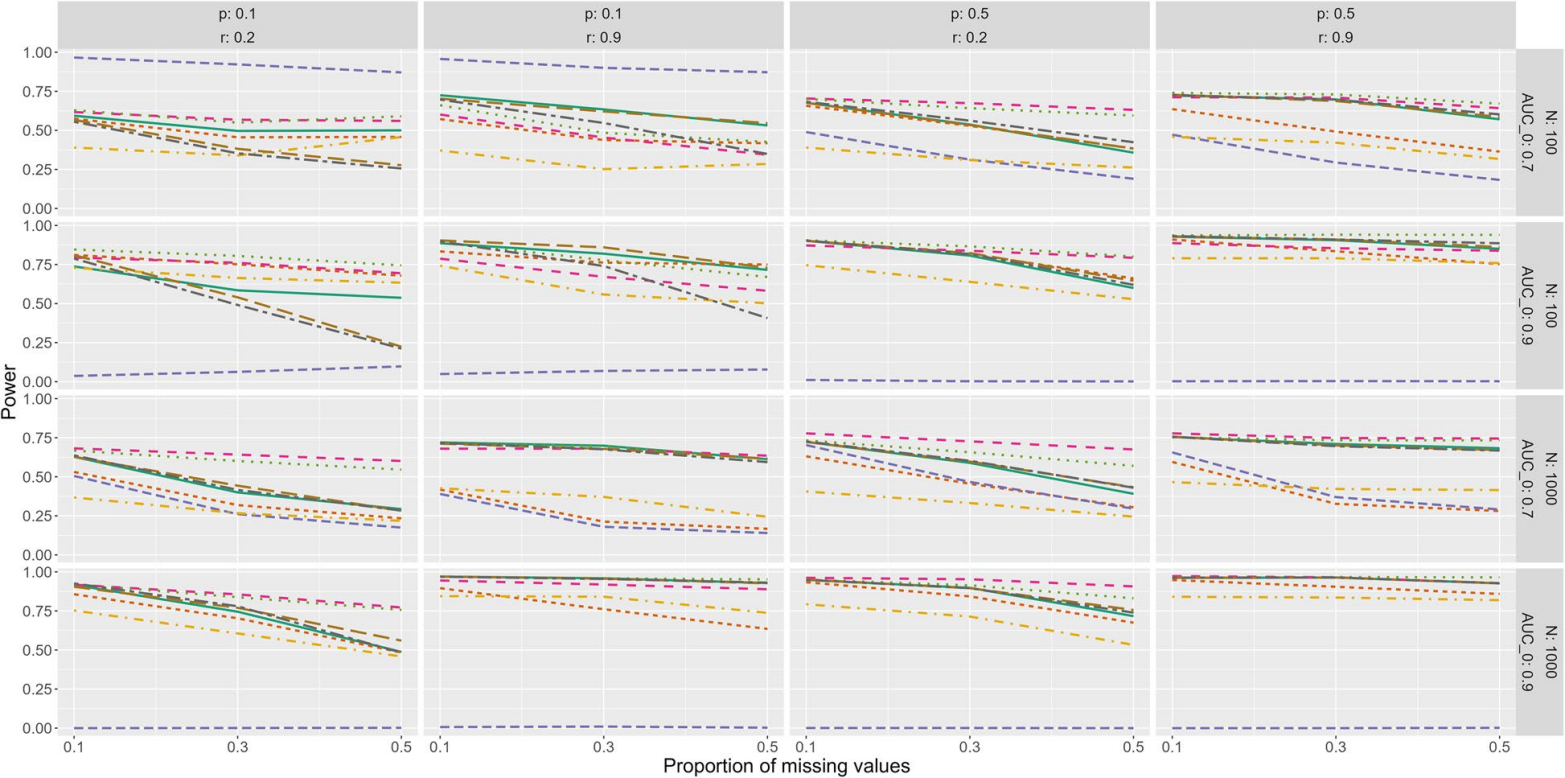
Power under MAR. r: correlation between index test and covariates, AUC_0: true AUC, p: prevalence of the target condition

The power of all methods decreases under MAR (Fig. 5) and MNAR (Additional file 4 Figure 27) compared to MCAR (Additional file 4 Figure 25) while the pattern of the methods’ performance is similar between these three missingness mechanisms. In contrast to MCAR, the power of mice, and mix but not AIPW decreases more sharply if the correlation between index test and covariates is low. On the other hand, they show a higher power than KER and MI2 if the correlation is high.

To conclude, all methods are severely biased if the prevalence and sample size are small, but AIPW improves with increasing prevalence. Mice, mix and AIPW perform well given a large sample size (especially if the correlation is also high).

### Missing not at random

Similar to MCAR and MAR, AIPW, mice, and mix are severely biased under MNAR if the sample size is small, prevalence of the target condition is low and the proportion of missing values is high. If the correlation is high, also MI2, MIB2 and KER are severely biased. In contrast, CCA and HDEL provide good results for a small sample size and low prevalence. With the increasing prevalence of the target condition, the performance of all methods improves (Additional file 4 Figures 5 and 11). Nonetheless, all methods tend to underestimate the AUC if the correlation is low, and CCA and HDEL still underestimate the AUC if the correlation is high. Regardless of the prevalence of the target condition, all methods underestimate the true AUC similarly for larger sample size (Additional file 4 Figures 6 and 12) and low correlation. For a higher correlation, most methods – especially AIPW – perform better apart from CCA and HDEL, which are still very biased. If the proportion of missing values is low, all methods give quite unbiased results although they tend to underestimate the AUC if the prevalence is low. Notably, there are some extreme outliers with regard to the underestimation of the true AUC for HDEL and KER in the case of *N* = 100 and for mix, CCA, MI2, and MIB2 in the case of *N* = 500.

Regarding the RMSE (Additional file 4 Figures 17 and 18), all methods perform similarly under MNAR as under MAR if the sample size is small (mix has the lowest RMSE if the correlation is low and mice, AIPW, and mix if the correlation is high). If the sample size is large and the correlation is low, mix and KER have the lowest RMSE. All methods perform quite similarly though. With higher correlation and large sample size, CCA and HDEL show considerably higher RMSE than the other methods. Following MAR and MCAR, mice, AIPW, and mix have the lowest RMSE in these scenarios.

The coverage probability under MNAR is mostly similar to MCAR and MAR if the prevalence of the target condition is high (Additional file 4 Figures 23 and 24). With some exceptions, most methods have a coverage probability close to 85% and 95% given a small and large sample size, respectively, if the proportion of missing values is low. Coverage probability decreases severely with an increasing proportion of missing values for KER, MI2, MIB2, CCA, and partly mice if the sample size is small and for KER and MI2 if the sample size is large. However, some of these methods are too biased to interpret the coverage probability adequately.

The pattern for power across the methods is comparable between MNAR and MAR.

The supplemental MNAR scenarios (Additional file 2) show that all methods are biased in every scenario except AIPW, KER, mice and mix if the sample size is 500 and the prevalence of the target condition is 10%. Compared to the MNAR scenarios of the main simulation, the methods are overall more biased in these supplemental scenarios (see Figures 2 and 3 in Additional file 2, and compare Additional file 4 Table with Additional file 2 Table 10).

While CCA and HDEL outperform the other methods given a small sample size and low prevalence, they are similarly biased in other cases if the correlation is low. In contrast to CCA and HDEL, all other methods improve with higher correlation though. However, the supplemental MNAR scenarios show that the methods’ performance is dependent on the type of MNAR mechanism.

### Running time and Monte Carlo standard errors

The methods’ running time is mainly influenced by the sample size (Additional file 4 Table 3, Figures 28 and 29). The fastest methods are CCA (mean time per simulation run 0.004 sec, SD 0.001), HDEL (mean 0.031 s, SD 0.016), MI2 (mean 0.108 s, SD 0.053), and KER (mean 0.214 s, SD 0.219). The other multiple imputation methods need more time: MIB2 (mean 0.504 s, SD 0.337), mix (mean 0.602 s, SD 0.245), and mice (mean 1.602 s, SD 0.18). AIPW requires the most time, especially with a larger sample size (mean 14.531 s, SD 14.325; if *N* = 100: mean 2.038 s, SD 0.132 compared to *N* = 1,000: mean 31.985 s, SD 11.046).

The MCE (Additional file 4 Table 6) for all methods across all scenarios are on average 0.001 (SD 0.001) and < 0.001 (SD < 0.001) for bias and mean squared error, respectively. They are slightly higher for the other performance parameter.

### Metamodels

The metamodels of the first approach show that simulation parameters vary in their influence on the performance measures depending on the method and performance measure (Additional file 4 Tables 7–8). The bias of CCA and HDEL is more influenced by the correlation between index test and covariates under MAR, and by the interaction of AUC with correlation and with prevalence under MNAR. In contrast, the sample size, prevalence, proportion of missing values and their interactions have a higher effect on the bias of AIPW, KER, mice and mix under MAR/MNAR. The bias of MI2 and MIB2 is affected by some simulation parameters to a lesser extent. Nonetheless, the performance of all methods is dependent on the proportion of missing values. The explained variance is higher under MAR and MNAR compared to MCAR for most variables. Regarding the RMSE, there are little differences between the methods. While the true AUC affects the performance of some MI methods only slightly, it is more important for the other methods. Overall, the sample size, prevalence, and proportion of missing values are most important. The explained variance is higher for RMSE than bias regardless of missingness mechanism.

The second approach (Additional file 4 Tables 9–10) shows that, adjusted for simulation parameters and their interaction, all methods (except HDEL) are associated with lower bias than CCA under MAR. However, this is only the case for mice and mix for RMSE. There are only a few differences in bias or RMSE under MCAR or MNAR. Based on the interaction terms, mice and mix tend to have a smaller bias with higher prevalence and correlation between index test and covariates. In contrast, a higher bias is associated with KER, AIPW, MI2, MIB2, mice, and mix if the true AUC increases. This is also true for mice and mix for RMSE. Concerning the RMSE, KER shows a better performance with increasing prevalence and mice, mix, and AIPW with increasing correlation.

Overall, the results of the metamodels confirm the patterns observed in the figures above.

### Additional methods in supplemental scenarios

Detailed results of the performance of the CONV, mi, mice (norm), mice (pmm), IPL LG, and IPL NP methods are presented in Additional file 1. Both IPL methods tend to underestimate the true AUC with an increasing proportion of missing values, especially under MAR and MNAR. In contrast, the CONV, mi, and both mice methods perform well under MCAR and MAR. However, CONV overestimates the AUC if the proportion of missing values is high under MAR while the MI methods somewhat underestimate the AUC in some cases. Under MNAR, all methods underestimate the AUC. CONV shows the least biased results though. Similarly, IPL LG and IPL NP have a higher RMSE than the other methods. Overall, CONV and mice (norm) have the lowest RMSE. While the IPL methods show a (too) high coverage probability in case of a few missing values, their coverage probability decreases sharply with increasing missing values, which can also be seen for the CONV and mi in a less pronounced way. Overall, CCA, mice (pmm) and mice (norm) show a coverage probability close to 95% except for CCA and mice (pmm) if many missing values are MNAR. Regarding the power, CONV outperforms the other methods while IPL-LG and IPL-NP perform better than CCA but worse than the MI methods.

### Results of the case study

Results of the case study are presented in Additional file 3. The case study data restricted to left ear measurements comprise 2540 newborns of which 80 newborns are hearing impaired according to the reference test. Mean age and sex distribution did not differ between those with impaired and non-impaired hearing. Mean DPOAE was −4.9 and −9.1 for newborns with and without impaired hearing, respectively. The AUC estimated by a complete case analysis based on this complete dataset (ACU_complete_) was 0.64 (95% CI: 0.57, 0.70).

For MCAR, the AUC estimates of all methods are close to the true value and range from 0.63 (mix, mi) to 0.65 (MI2). All methods overlap the AUC_complete_ with their CI, whereby MIB2 has the widest CI. Overall, the ROC curves estimated by the HDEL, mice, KER, MI2, MIB2 and CCA methods lie closest to the ROC based on the complete dataset (ROC_complete_).

**Fig. 6:**
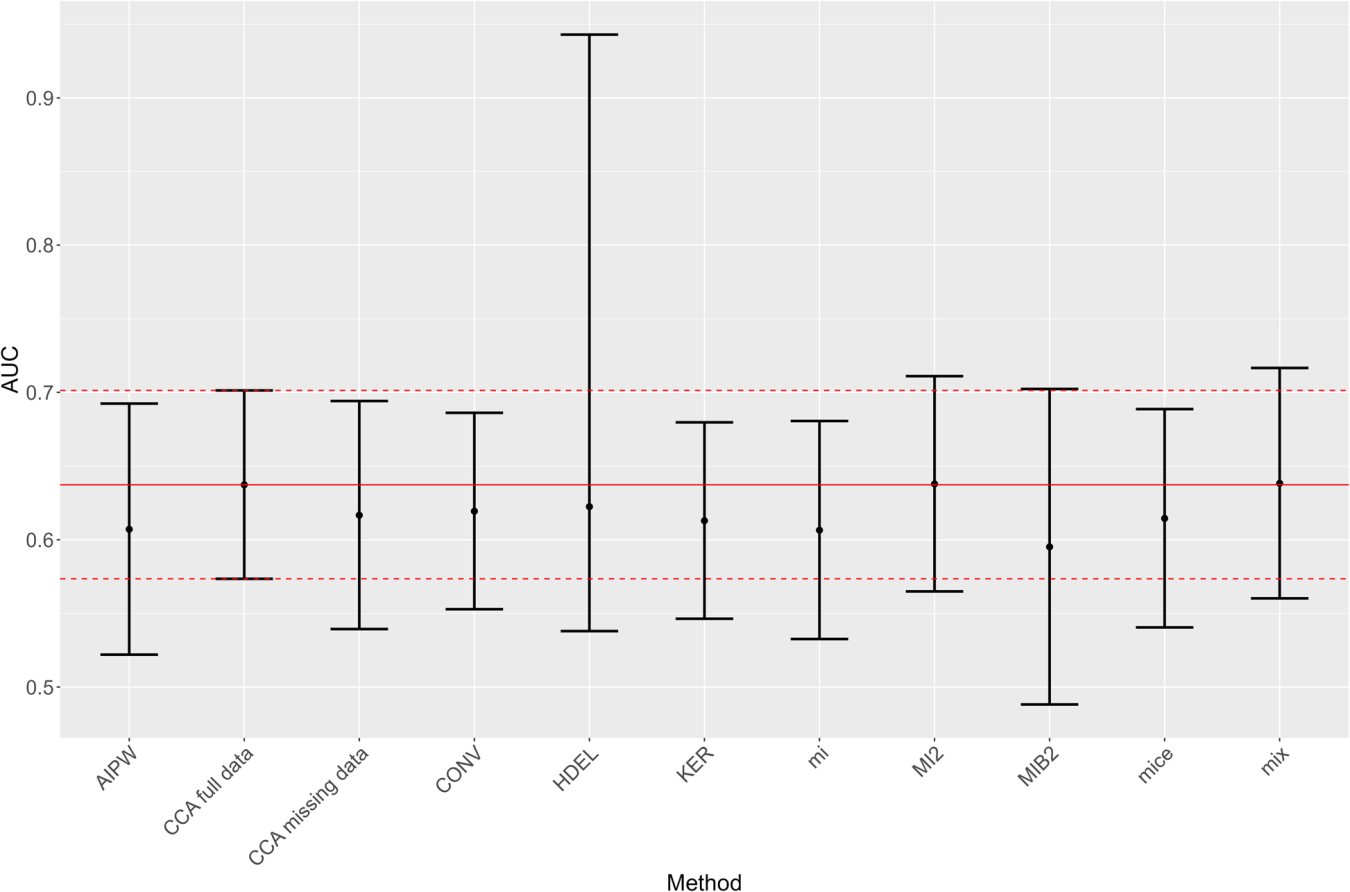
The AUC and its CI estimated by each method under MAR

Apart from mix and MI2, all methods tend to underestimate the AUC under MAR and give AUC values ranging from 0.60 (MIB2) to 0.64 (mix and MI2) (Fig. 6, and Table 3 in Additional file 3). It is noteworthy that the upper confidence limit of the HDEL method seems to be an outlier, which results in a very wide CI for the HDEL method. The AUC and CI estimates of mix and MI2 are closest to the AUC_complete_ and its CI.

**Fig. 7:**
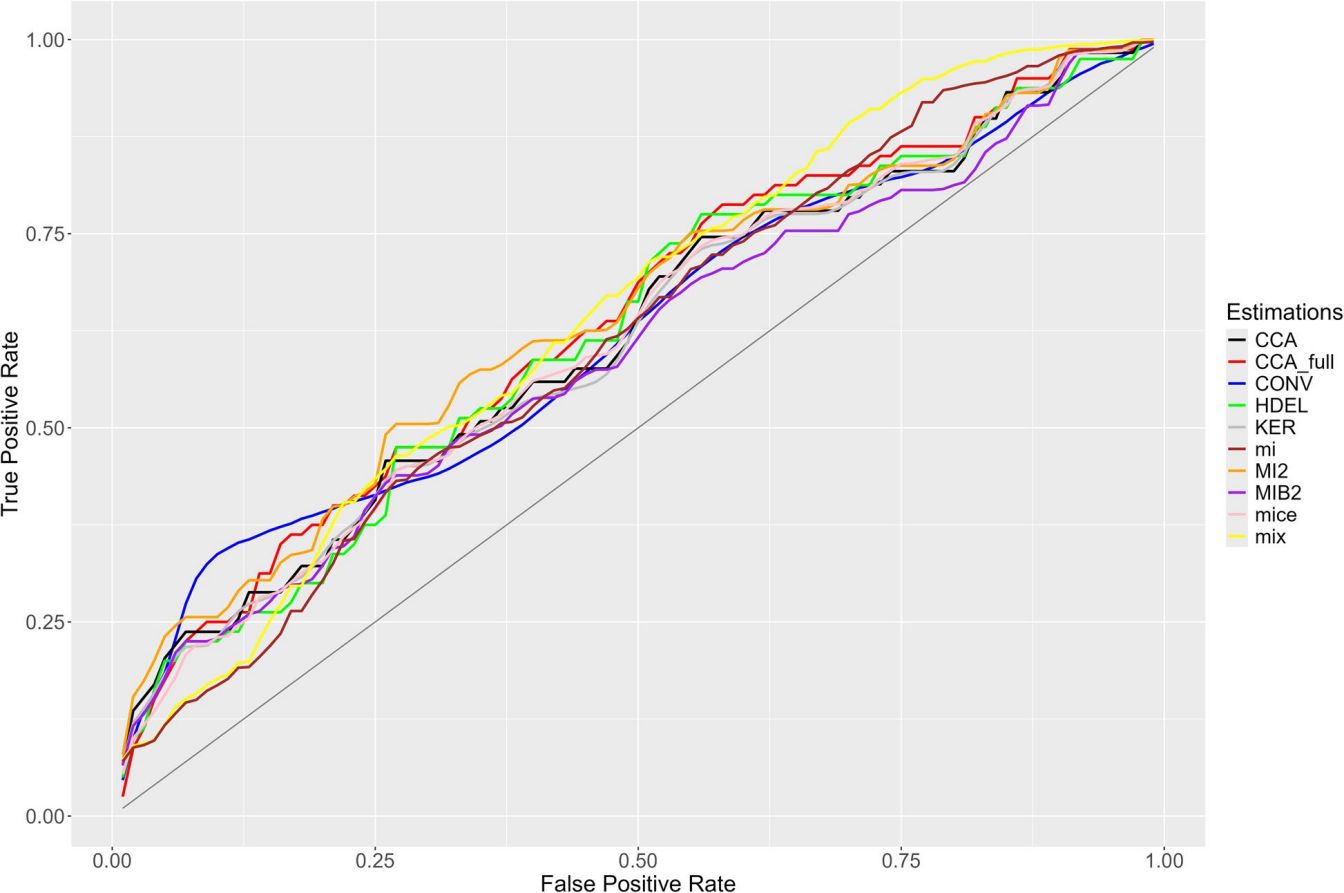
The ROC curve estimated by each method under MAR

Comparable with the ROC curves under MCAR, the ROC curves of the KER, mice, CCA and HDEL methods lie closest to the true ROC curve. However, they differ more from the ROC_complete_ than under MCAR. The ROC curves of all methods are closer to the true ROC curve if the FPR is between 25 and 70% (Fig. 7).

All methods tend to underestimate the true AUC slightly more under MNAR than under MAR. Their AUC estimates range from 0.57 (MIB2) to 0.61 (mice and mi). Overall, the methods using standard multiple imputation approaches (mice, mix, mi) tend to perform better under MNAR than the other methods. Similar to MAR, the upper confidence limit of the HDEL is rather extreme. Similar to the AUC results, all methods tend to underestimate the ROC. Of all methods, the ROC curves of mice lies closer to the true ROC curve than the ROC curves of the other methods.

## Discussion

This study compared the standard CCA with MI methods, inverse probability weighting, and empirical likelihood methods regarding their handling of missing values in the index test of a single-test design diagnostic study. The methods were applied to simulated data and a modified case study.

In summary, it can be concluded that there is no method suitable for every situation. It rather depends on certain parameters and their combinations. Particularly the prevalence of the target condition and the proportion of missing values have a high effect on bias and RMSE under MAR and MNAR while they are less (but still) important for MCAR. If missing values are MCAR, all methods give good estimates if the sample size is large. For a small sample size, many methods tend to be biased for many missing values except for CCA and HDEL (and AIPW for high prevalence). This is even more pronounced if missing values are MAR. In this case, all methods are severely biased if the sample size and the prevalence of the target condition are small. If the prevalence increases, MI methods are still biased while AIPW gives good estimates. Mice and mix perform well for a large sample size and correlation. In contrast to MAR, CCA and HDEL provide quite unbiased results under MNAR if the sample size and prevalence are small whereas all other methods are biased. Given a higher prevalence and a small sample size and given a large sample size in general, all methods underestimate the AUC if the correlation is low. All methods except for CCA and HDEL improve with increasing correlation. It is noteworthy, that all methods provide unbiased estimates for the AUC given a small proportion of missing values except for KER under MCAR, MAR and MNAR if the sample size is small and except for CCA and HDEL under MAR and MNAR if the sample size is large. In contrast to bias, mice, mix and AIPW (only for high correlation) outperform the other methods in terms of RMSE regardless of missingness mechanism.

In addition to the simulation study, the modified case study gives further insights into the performance of the methods under more “realistic” conditions. Whereas all methods give close estimates for the AUC and its CI under MCAR, they tend to underestimate the AUC_complete_ under MAR and MNAR. Mix and MI2 approximates the AUC_complete_ best under MAR, while mice, mi, CONV, AIPW, and KER are also close. Looking at the ROC curve, mice, KER, HDEL and CCA produce ROC curves which lie nearest to the true ROC curve under MCAR and MAR. Under MNAR, however, it is more difficult to identify the best method(s) as all curves underestimate the ROC_complete_ similarly.

Comparing the performance of the methods, it has to be noted that HDEL and CCA are designed for MCAR and not for MAR unlike the other methods. Thus, it is not surprising that HDEL performs quite similarly to CCA in terms of bias and should not be preferred for MAR or MNAR. However, it should be used with caution even under MCAR as the case study shows that its CI may be prone to outliers. The IPW methods (AIPW, KER, and CONV) perform differently depending on the scenario. Besides CCA, AIPW is the only method resulting in failed AUC results since the prediction score needed for the IPW estimate cannot be calculated if there are too many observations with missing values in one index test group. This is especially the case when the sample size and the prevalence are small. If AIPW produces an estimate for the AUC under these scenarios, it performs worse than the other methods. Nonetheless, AIPW may be a suitable method if the sample size is larger and the prevalence of the target condition is higher. If, in addition, the correlation between the index test and covariates is high, it is also quite robust under MNAR. In contrast to all other methods, AIPW is, however, the only one that cannot calculate the ROC easily. Interestingly, KER is more biased than all other methods given a few missing values (except for CCA and HDEL under MAR or MNAR) but similar with increasing proportion of missing values (with lower coverage probability though). This is surprising, as Bianco et al. [31] state that KER performs better than its competitors for complete data in a small sample. Our supplemental calculations and the case study show promising results for CONV. Nonetheless, its long running time (ca. 15 min for one run) makes this method rather impracticable. Future simulation studies should also include this method in their main investigation. If its good performance is confirmed by more comprehensive explorations, the CONV method may be an alternative to other methods. Schafer and Graham [43] argue that IPW methods may be easy to apply for univariate and monotone missingness but it is much more difficult to calculate weights for more than one variable with missing values. Therefore, it has to be noted that the examined IPW methods are solely designed to handle missing values in the index test and cannot be used if missing values also occur in the reference test or covariates. In contrast, this is an advantage of the standard MI methods (mice, mix and mi) which can address missing values in more than one variable. Past research recommends their application to missing values in the reference standard [44]. To our knowledge, no study has investigated their performance in diagnostic studies with missing values in the index test, reference test, and covariates. Facing missing values solely in the index test, mice and mix perform well for MCAR and MAR in our simulation study if the sample size is large. This could be expected, as MI relies on a large sample [43]. Moreover, their performance increases with a higher correlation between index test and covariates, and, in this case, they are also quite robust to MNAR. This is also in accordance with previous research claiming that its performance may be improved by including more auxiliary variables associated with the index test [43]. In both our case study and simulation study, mix slightly outperformed mice. This somewhat meets previous research which observed, that MI using joint distribution is slightly more efficient than conditional MI if the model is correctly specified [45]. Both approaches perform similarly if the variable of interest is binary or continuous [46, 47]. If the variable of interest is categorical, conditional MI, particularly using PMM, outperforms joint MI [46, 47]. The supplemental method mi performs comparably to mice (pmm) which is not surprising as it also employs conditional specification. Nonetheless, mi is slightly outperformed by mice (pmm and norm) in terms of RMSE and coverage. Van Buuren [47] state that choosing Bayesian linear regression (norm) as imputation method for mice could be more efficient if the variable of interest is normally distributed. Our supplement data confirm this as mice (norm) slightly outperforms mice (pmm) and mi in terms of RMSE and coverage probability. This is probably due to simulating multivariate normal distributed data. If data are deviating from the normality assumption or the imputation model is wrongly specified, PMM may be the better option as it is more robust [47, 48]. The other MI methods, MI2 and MIB2, show an ambivalent performance across our simulation and case study. While they perform well in some instances, they tend to over- or underestimate the AUC in many scenarios. Previously, a similar approach [49], even with extension to MNAR [50], was investigated in another research context with good results. However, the authors examined the performance in a few scenarios and iterations, and did not compare it to standard MI methods (such as mice or mix). In contrast to the standard MI methods, they would not be able to handle missing values in the reference test or covariates since the imputation step is performed stratified by target condition status. Owing to this very mixed performance, we would not recommend these methods. The same applies to the IPL methods, examined in the supplemental scenarios. In addition to taking much time, they result in biased results, a high RMSE, and undercoverage if the proportion of missing values is high. However, this may be due to some ambiguities in the code which we were not able to solve.

Upon a reviewer’s suggestion we additionally explored different correlations between the index test and covariates and the covariates themselves. The results indicated that the methods using covariate information benefit most from an increase in the correlation between the index test and covariates. The correlation between the covariates themselves has little impact on the methods’ performance.

Although none of the considered methods are designed for MNAR, AIPW, KER, mice, and mix perform better than expected under MNAR if the sample size is large and the correlation between the index test and covariates is high. Concerning the MI methods, this is consistent with Schafer and Graham [43] and Mustillo and Kwon [51], who state that MI is quite robust. We assume that the performance under MNAR is heavily dependent on the underlying MNAR mechanism and the data at hand. In our simulation study, the missingness in the index test increases as the index test values increase (higher index test values are more likely to be missing). This is the default setting in the ampute function used for inserting missing values. Consequently, the methods including the covariates tend to be less biased given a high correlation between index test and covariates. Such an association was also shown in the field of sociology by Mustillo and Kwon [51]. The supplemental MNAR scenarios using type = MID (medium index test values are more likely to be missing) are less likely to profit from including the covariates. This is reflected in the biases of the supplemental MNAR scenarios which is higher for all methods (with some small exceptions) than the biases of main MNAR scenarios). Therefore, it may be interesting for future research to explore the performance under further MNAR scenarios (e.g. if the missingness is dependent on unobserved variables).

Our simulation study addresses the question, of which is the most adequate method(s) from a methodological point of view. However, it must also be discussed how the methods are accepted from a practical point of view. For instance, MI methods may seem very complex to many clinicians conducting a diagnostic study. Many details need to be considered when using MI including the right imputation model, the imputation method and the number of imputations among others. Therefore, some researchers might be more open to use one of the IPW methods as they are more familiar with them and can use them straightforward without needing to define details. Nevertheless, mice and mix have already been implemented in software and comprehensive tutorial material is available. It is crucial for other methods (such as AIPW) to be implemented in ready-to-use software to encourage their application. We provide the R code of our calculations as supplemental material. Each method is programmed as a function, which can be used by other researchers for their data.

Furthermore, we deem it important to discuss possible strategies for handling missing values (as well as inconclusive results) already when planning a diagnostic study. In this phase, one should think about possible reasons for missing values and how to prevent them. If they nonetheless occur, it is important to consider how they may be related to the patient’s target condition: Will the missing values be completely random, independent of the patient’s target condition (but maybe related to other information), or informative with respect to the patient’s true target condition status? In combination with examining missingness patterns in the data, such considerations may help to make assumptions about possible missingness mechanisms. Furthermore, there are different tests implemented in various R packages to detect MCAR, such as the MCAR test by Little [52] in the package naniar [53], or a test by Jamshidian and Jalal [54] in mice [19]. Potthoff et al. [55] present an approach to explore special MAR patterns and medical examples for its application. Nonetheless, the decision about MAR or MNAR cannot be answered fully by data examination, but is rather based on assumptions about information on causes and patterns of missingess. Sisk et al. [56] discuss the issue of (causes for) missing values in the context of prediction studies and expand their considerations to the question whether missing values can also occur at the deployment stage (i.e. in practice) and how they are handled then. If they are likely to occur at the deployment stage of an index test as well, it may be more reasonable to report the missing values than to account for them using an advanced method. For instance, this could be the case if an index test does not work for patients with a certain characteristic (e.g. a certain medication). Consequently, it may not be of interest what their missing value would be hypothetically. Instead, this index test should then not be recommended for this patient group in practice. This example illustrates that it is not only about choosing the right method for missing values, but also about making well-founded considerations about their reasons, mechanisms and consequences. The latter fits to the estimand concept, which aims at defining an estimand and deriving strategies to handle interfering events (which may result into a missing value) for a given study [57]. While it has been proposed for therapeutic studies, it is currently under development for diagnostic studies [58]. Fierenz et al. [58] differentiate between results affected by an interfering event, which can lead to a non-existing value, and a missing value, which is not due to an interfering event. Additionally, they propose strategies to handle results affected by interfering events based on a defined estimand. To obtain valid estimates in line with the estimand defined a priori, it is important to combine strategies to handle interfering events with those handling missing values. Future research should focus on combining findings on handling missing values and the estimand concept in diagnostic studies into a joint framework.

Until then we propose the following recommendations for handling missing values in a continuous index test in a single- test diagnostic study. Since the occurrence and handling of missing value is often not reported adequately [12, 59], some recommendations apply generally to the handling of missing values in a diagnostic accuracy study. Recommendations specific to missing values in a continuous index test are mainly based on our results for RMSE and bias as these measures provide the most informative insight whether an estimate is close to the true value in a simulation setting:

**Table:** 

**Planning phase**			
• Put maximum effort into preventing missing values in index test results in a diagnostic accuracy study as there is no approach for handling missing values without concerns. A feasibility study prior to the main study may provide insight in the frequency and reasons for missing values.			
• It may be insightful to simulate likely mechanisms for missing values in the design phase of a diagnostic accuracy study. The performance of several approaches could then be compared. If simulating scenarios is not possible, specific scenarios can be examined in existing simulation studies (such as this one).			
• Select, describe and provide rationale for the main approach for handling missing index test results and planned sensitivity analyses in the protocol when designing an accuracy study. The selection should be based on the likely causes for missing values and in line with the estimand of the diagnostic accuracy study at hand.			
**Analysis of a continuous index test with missing values**			
• No approach for handling missing values is superior to others across all scenarios.			
• In case of prospective accuracy study, analyze the data according to the approach specified in the protocol. If information arises during the study or during the data analysis, a different approach may be performed and presented, but the rationale must be provided and the deviation from protocol highlighted. A sensitivity analysis disagreeing with the pre-specified main analysis is not a sufficient reason. This may be the case if the initial methods does not work and not if a sensitivity analysis disagrees with the initial approach.			
• CCA can be recommended if the percentage of missing values is so low that no meaningful change in AUC can be expected by using any statistical method. This can be checked by single imputation of extreme values.			
• HDEL, and MIB2 are not recommended as these methods are only adequate for handling missing values under MCAR (HDEL) and/or show inconsistent results across the main simulation and case study.			
• Statistical imputation techniques can lead to more bias than CCA if the effective sample size is small (a combination of small absolute sample size, low prevalence, and high frequency of missing values). Accuracy studies with a small total sample size and many missing values provide a low certainty of evidence irrespective of the approach of handling missing values.			
• Overall, we recommend the following methods depending on sample size and prevalence of the target condition:			
		**Prevalence of the target condition**	
		**Low**	**High**
**Sample size**	**Small**	CCA^1^	AIPW (high r), CCA (low r)
	**Large**	mix, AIPW (high r)	mice, mix, AIPW (high r)
^1^CCA gives valid estimates under MCAR but biased estimates under M(N)AR. There is no better method in this case though; r: correlation between index test and covariates			
• Irrespective of the main approach for handling missing index test results, provide the results for the CCA as sensitivity analysis to inform the reader about the magnitude of change.			
• Further sensitivity analyses may add value if the missingness mechanism is unclear or possibly MNAR. Under MNAR, KER may be a promising alternative to the main approach.			
• If model-based approaches such as MI are chosen for the main or sensitivity analysis: the outcome of the reference standard must be included in the imputation model [60] and suitable covariates should be selected [47].			
**Reporting**			
• Report the frequency of missing values among patients with and without the target condition and any reasons or factors related to their occurrence. Additionally, it should be discussed whether these are also likely to occur in future practice.			
• It is critical to completely and accurately report which approach has been applied [1] and highlight the rationale for the selected approach relative to other options.			

### Limitations

This simulation study has some limitations that need to be explained. First, our results are only valid for the scenarios and the case study considered in this paper. However, researchers may still use our results to a certain extent even if their data do not match our assumptions by transforming their variables. Nonetheless, other neutral comparison studies with a (slightly) different scope are likely to obtain other findings [15]. Further phase III and phase IV studies are certainly needed to make more comprehensive recommendations on using methods in diagnostic studies with missing values. Owing to ambiguities in the reporting of the IPL methods, the limited applicability of the method by Lin et al. [32], the long running time of the CONV method, and technical difficulties for mi, we could not include these methods in our main examinations. Nevertheless, we included the CONV and mi method in the case study and examined its performance together with the IPL methods in a reduced additional simulation set. For the application of the methods to the case study, we were not able to consider the clustered design of the original data. This is due to the fact that the methods were primarily developed for a single test design. At last, we could show results on the ROC curve only for the case study datasets and were not able to investigate missing values in both the index test and reference standard or a binary index test because of feasibility reasons. This would be beyond the scope of this paper and should be examined in further studies.

### Strengths

Nonetheless, our study has several strengths. Foremost, we simulated a wide range of scenarios and, in addition, applied the methods to modified case study data, which enhanced the representativeness of the results [14, 15]. The MCEs for all methods are ≤ 0.001 for bias and RMSE, which indicates that the number of iterations were sufficient to ensure a low level of uncertainty of our results. Furthermore, the methods were selected based on a recent systematic literature search [11] and, thus, as comprehensive as possible. As required for neutral comparison studies [15, 16], we focused on the comparison of competing methods and not the development and were neutral towards the selected methods. Finally, our paper provides the first systematic comparison of methods and recommendations to handle missing values in a continuous index test of diagnostic studies, and thereby, it enables other researchers to make informed decisions about the use of an appropriate method for their diagnostic study.

## Conclusion

In conclusion, our findings show that the choice of a suitable method for missing values in a continuous index test depends on the sample size, prevalence of the target condition, and, to a lesser extent, on the correlation between the index test and covariates. Given a medium to large sample size, standard MI methods, such as mice and mix, give good estimates. If the correlation between the index test and covariates is high, the augmented IPW method (AIPW) is also a good alternative. However, most methods perform poorly if the sample size and the prevalence of the target condition are small. In this case, CCA and AIPW may be the best options if the correlation is low and high, respectively. Future research is needed to put these results into the broader framework of the estimand concept and to investigate methods for handling missing values in more than one variable.

## Supplementary Information

Additional file 1. Additional methods to handle missing values in a continuous index test in a diagnostic study – supplemental simulation scenarios

Additional file 2. Methods to handle missing values in a continuous index test in a diagnostic study – supplemental MNAR scenarios

Additional file 3. Results of the case study

Additional file 4. Comparison of methods to handle missing values in a continuous index test in a diagnostic study – a simulation study (Additional file 4). Detailed results of the main simulation

Additional file 5. Results of the performance parameters for all main scenarios (aggregated across iterations)

## Data Availability

All (intermittent) data and R programs used for the simulation and case study are available at the MisEstiDiag/missing-values-diagnostic-studies repository (https://github.com/MisEstiDiag/missing-values-diagnostic-studies; latest version: 10.5281/zenodo.14761064). The case study data are available at the Diagnostic and Biomarkers Statistical (DABS) Center (https://research.fredhutch.org/diagnostic-biomarkers-center/en/datasets.html. Accessed 21 Dec 2023).

## Abbreviations

AIPW: Augmented Inverse Probability Weighting Method
AUC: Area under the Receiver Operating Characteristic Curve
AUC_complete_: AUC based on the complete dataset (without missing values)
AUC_min_: Minimum AUC defined
AUC_0/AUC_true_: True AUC used for the data generation
CCA: Complete Case Analysis
CI: Confidence Interval
CONV: Convolution-based method
DPOAE: Distortion product otoacoustic emissions (index test in the case study)
FPR: False positive rate
HDEL: Hot-deck Empirical Likelihood Approach
IPL-LG: Hybrid Imputation using a logistic approach
IPL-NP: Hybrid imputation using a non-parametric approach
KER: Kernel-based approach
r: Correlation between index test and covariates
MAR: Missing at Random
MCAR: Missing Completely at Random
MCE: Monte Carlo Standard Errors
MI: Multiple Imputation
MI2: Multiple Imputation using a prediction and propensity score
MIB2: MI2 with an additional bootstrap step
mice: Multiple Imputation by Chained Equations using the mice R package
mix: Multiple Imputation using joint modelling by the mix R package
mi: Multiple Imputation using chained equation by the mi R package
MNAR: Missing Not at Random
N: Sample size
p: Prevalence of the target condition
pm: Proportion of missing values
PMM/pmm: Predictive Mean Matching
RMSE: Root Mean Squared Error
ROC: Receiver Operating Characteristic Curve
ROC_complete_: ROC based on complete data (without missing values)
SD: Standard deviation
