# Supplementary material for: Comparison of methods to handle missing values in a continuous index test in a diagnostic accuracy study – a simulation study
Source: BMC Med Res Methodol. 2025 May 27;25:147. doi: 10.1186/s12874-025-02594-2 (PMC12107930; doi:10.1186/s12874-025-02594-2)
Supplement: Supplementary file 1 — Additional file 1. Additional methods to handle missing values in a continuous index test in a diagnostic study – supplemental simulation scenarios [file 12874_2025_2594_MOESM1_ESM.html]

Additional methods to handle missing values in a continuous index test in a diagnostic study – supplemental simulation scenarios (Additional file 1)


Code 

- Show All Code
- Hide All Code

# Additional methods to handle missing values in a continuous index test in a diagnostic study – supplemental simulation scenarios (Additional file 1)

#### Katharina Stahlmann, University Medical Center Hamburg-Eppendorf Institute of Medical Biometry and Epidemiology, k.stahlmann@uke.de,

#### Bastiaan Kellerhuis, Julius Center for Health Sciences and Primary Care, University Medical Center Utrecht, Utrecht University, Utrecht, The Netherlands; Blavatnik School of Government, University of Oxford, Oxford, UK

#### Johannes B. Reitsma, Julius Center for Health Sciences and Primary Care, University Medical Center Utrecht, Utrecht University, Utrecht, The Netherlands

#### Nandini Dendukuri, Department of Medicine, McGill University, Montreal, Canada

#### Antonia Zapf, University Medical Center Hamburg-Eppendorf Institute of Medical Biometry and Epidemiology

#### 2025-04-25

```
knitr::opts_knit$set(root.dir = rprojroot::find_rstudio_root_file())
                       
# set global chunk options
knitr::opts_chunk$set(echo = TRUE, message = FALSE, warning = FALSE,
                      fig.width = 12,
                       fig.asp = 0.8 ,
                       out.width = "120%")

# this options for word export
#knitr::opts_chunk$set(echo = FALSE, message = FALSE, warning = FALSE)

options(stringsAsFactors = F)
# always show NA is there is any in the table function
table = function (..., useNA = 'ifany') base::table(..., useNA = useNA)
```

# 1 Settings

## 1.1 Packages

```
library(ggplot2)
library(writexl)
library(ggpubr)
library(RColorBrewer)
library(dplyr)
library(arsenal)
library(rsimsum)
library(gt)
```

## 1.2 load functions

```
mycontrols = tableby.control(numeric.stats=c("Nmiss","mean", "sd", "medianq1q3", "range"),
                             cat.stats=c("Nmiss", "countpct"), 
                             stats.labels=list(Nmiss='Missing values', medianq1q3='Median (Q1, Q3)'),
                             test = F)

colors <- brewer.pal(7, "Dark2")
```

# 2 Simulation

## 2.1 Set up simulation parameters

```
nsim <- 100

#define simulation scenarios
grid = expand.grid(
  sim = 1:nsim 
  , N = c(500)
  , p = c(0.3)
  , AUC_0 = c(0.85)
  , r = c(0.5)
  , pm = c(0.1, 0.3, 0.5)
  , mech = c("MCAR", "MAR", "MNAR")
)

# methods to be compared
methods <- c("CCA", "CONV", "mi_", "mice_n", "mice_p", "IPL.LG", "IPL.NP")

Abbreviation = names(grid)
setup <- data.frame(
  Abbreviation = names(grid),
  Parameter = c("Number of simulations", "Sample size", "prevalence of the target condition", "True AUC", "Correlation between index test and covariates", "proportion of missing values", "Missingness Mechanism"),
  Values = c(
    nsim,
    sapply(Abbreviation[2:length(Abbreviation)], function(x) (paste(unique(grid[,x]), collapse = ',')))
  )
)  
setup %>%
  gt() %>% 
  tab_style(style = cell_text(weight = "bold"), locations = cells_column_labels(columns=c("Abbreviation", "Parameter", "Values"))) %>%
  tab_header(
    title = "Table 1. Overview of simulation parameter"
  )
```

|  |  |  |
| --- | --- | --- |
| Table 1. Overview of simulation parameter | | |
| Abbreviation | Parameter | Values |
| sim | Number of simulations | 100 |
| N | Sample size | 500 |
| p | prevalence of the target condition | 0.3 |
| AUC\_0 | True AUC | 0.85 |
| r | Correlation between index test and covariates | 0.5 |
| pm | proportion of missing values | 0.1,0.3,0.5 |
| mech | Missingness Mechanism | MCAR,MAR,MNAR |

## 2.2 Description of methods

Table 2. Overview of methods

| Methods |  |
| --- | --- |
| CCA | Complete case analysis |
| CONV | Convolution-based approach (Bianco et al. 2023) |
| mi | Multiple Imputation using chained equations with predictive mean matching (PMM) (Su et al. 2022) |
| mice (pmm) | Multiple Imputation using chained equations with predictive mean matching (PMM) (van Buuren et al. 2011) |
| mice (norm) | Multiple Imputation using chained equations with Bayesian linear regression (van Buuren et al. 2011) |
| IPL LG | Hybrid Imputation using a logistic model (Cheng and Tang 2020) |
| IPL NP | Hybrid Imputation non-parametric (Cheng and Tang 2020) |

### 2.2.1 Mice

Multiple imputations by chained equations is a standard multiple
imputation approach that employs fully conditional specification via
Markov Chain Monte Carlo methods. It can be accessed through the mice
package in R (van Buuren and Groothuis-Oudshoorn 2011). For each
variable with missing data, conditional densities are specified.
Multivariate imputation is performed variable-by-variable and iterating
over the conditional densities. We selected predictive mean matching as
imputation method since this is the default method for numeric variables
in mice and performs well overall (van Buuren and Groothuis-Oudshoorn
2011). As Bayesian linear regression may be more efficient for normally
distributed data (van Buuren 2018), we include mice using this
imputation method (called norm) in addition to mice with PMM in this
supplemental simulation scenarios. We set the number of iterations to 10
and generated 20 imputation datasets for our simulation as Schafer and
Graham (2002) deemed 20 imputations to remove most noise in the
estimates. All variables (index test, the three covariates and the
reference standard) without interaction terms were included in the
imputation model. After imputation, estimators for the AUC and
sensitivities for different FPR values are pooled by Rubin’s rules
(Rubin 1987).

### 2.2.2 Mi

The R package mi was published by Su et al. (2011) and employs fully
conditional specification as well. To be consistent, predictive mean
matching was selected as imputation method, too. In congruence with
mice, we selected 10 iterations, generated 20 imputation datasets and
used all variables but no interaction terms in the imputation model. The
results are only reproducible if the parallel argument is set to FALSE
in the function, and this method could not be executed on the
high-performance cluster. Thus, we decided to include this method only
in this small set of supplemental scenarios.

### 2.2.3 CONV

Bianco et al. (2023) calculated a convolution-based estimator
assuming that covariates (in this simulation study x2, x3, x4) are
related to the index test values through a regression model. This
regression function may be parametric, non-parametric, or
semi-parametric. Bianco et al. (2023) assumed a parametric linear
regression model. The convolution-based estimator is defined as the
convolution of the regression function distribution and the errors and
is a weighted empirical distribution. The sensitivity values for
different FPR values are calculated following this approach, and
subsequently, the AUC is calculated based on the sensitivity values.
Upon request, the authors provided this method as R code. As this method
takes very long to run, we excluded it from the main simulation study
but examined it within this reduced set of supplemental scenarios.

### 2.2.4 IPL-LG and IPL-NP

Cheng and Tang (2020) proposed two smoothed empirical likelihood
methods combining multiple imputation and inverse probability-weighted
imputation (i.e. hybrid imputation based on estimating equations).
Auxiliary variables can be incorporated into the propensity score
function. They proposed a parametric propensity score using a logistic
regression (IPL-LG) and a nonparametric propensity score function
(IPL-NP). Missing values are assumed to be MAR. Both methods are
available as R code in their supplemental material and are programmed to
estimate sensitivity values and their CI for defined FPR values. We
further extended their code to estimate the AUC and its CI as well and
set the same parameter options as Cheng and Tang (2020): the number of
imputation = 20. These both methods are not included in the main
simulation study but only in a reduced simulation study with 9 scenarios
and 100 repetitions. We decided to exclude them from the main
simulation, as it is not clear to us why and how they rescale the index
test and covariate values before running their method. In addition, both
methods take much time.

### 2.2.5 CCA

We compared the above explained methods with the standard complete
case analysis (CCA) which excludes all subjects with missing values in
the index test (or reference standard) from the calculations. For the
application of the CCA, we used the ci.auc function of the pROC package
(Robin et al. 2011).

## 2.3 Conduct the simulation

We cannot conduct the simulation programs within this Rmarkdown file,
as they were executed on a high performance cluster. However, we provide
the R programs and the results of the simulations to enable
reproduction. The seed for the parallel loop was set to 5273 and to
4730xi for data generation.

Load the simulation results

```
# Note: Owing to very long running times of some methods, we ran the simulation for each method separately. Thus we need to combine the data for each method for both parts in the following

data <- c("results_conv", "results_mi", "results_mice_n", "results_mice_p", "results_ipl")
list_dta <- list()

for (i in 1:length(data)) {
  
  load(file = paste0("./Analyse/hpc/Simulation_data/", data[i], ".Rdata"))
  res$id <- paste(res$sim, res$scenario, sep = "_")
  list_dta[[i]] <- res
  assign(paste0(data[i]), list_dta[[i]])
}

# merge results by id
res_dta <- merge(results_conv, results_mi[,c(10:ncol(results_mi))], by="id")
res_dta <- merge(res_dta, results_mice_n[,c(10:ncol(results_mice_n))], by="id")
res_dta <- merge(res_dta, results_mice_p[,c(10:ncol(results_mice_p))], by="id")
res_dta <- merge(res_dta, results_ipl[,c(10:ncol(results_ipl))], by="id")

res <- res_dta %>%
  arrange(scenario, sim) %>%
  select(-id) %>%
  rename(r = korr)
```

# 3 Calculate Performance parameter

The following performance parameter will be calculated: number of
missing values, bias, root mean squared error, empirical standard
deviation, coverage, bias-eliminated coverage and power as well as the
respective monte carlo standard errors for each performance
parameter.

```
fix_col <- c("N","p","AUC_0","r", "pm","mech", "AUC_min","scenario")
source("./Analyse/Simulation_performance3.R")
# input: res (file with simulation results as dataframe)
# out: raw_res (res + step 1 of performance calculation -> 1 row for each simulation run), scenario_res (dataframe with performance parameters, -> 1 row for each scenario aggregated over the runs)
```

# 4 Results

## 4.1 Table of performance parameter

```
# rearrange the scenario_res table columns, so that performance estimate and its MC standard error are located side by side
vars=list()
for (i in 1:length(methods)){
  vars_i <- grep(methods[i], names(scenario_res), value = T)
  vars[[i]] <- vars_i
}
vars_order <- unlist(vars)
dat_table1 <- scenario_res[,c(8,1:6)]
dat_table2 <- scenario_res[,vars_order]
dat_table <- cbind(dat_table1, dat_table2)
rownames(dat_table) <- NULL

# label selected variables in dat_table
dat_table <- dat_table %>%
  sjlabelled::var_labels(
    scenario = "Scenario",
    N = "Sample size",
    p = "Prevalence of target condition",
    AUC_0 = "True AUC",
    r = "Correlation",
    pm = "Proportion of missing values",
    mech = "Missingness mechanism",
    av.time.AUC.CCA = "Average running time for CCA",
    av.time.AUC.CONV = "Average running time for CONV",
    av.time.AUC.mi_ = "Average running time for mi",
    av.time.AUC.mice_n = "Average running time for mice (norm)",
    av.time.AUC.mice_p = "Average running time for mice (pmm)",
    av.time.AUC.IPL.LG = "Average running time for IPL LG",
    av.time.AUC.IPL.NP = "Average running time for IPL NP"
  )


# show table for bias
bias <- grep("bias", names(dat_table), value = FALSE)
knitr::kable(dat_table[,c(1:7,bias)], "simple", 
             col.names = gsub("[.]", " ", names(dat_table[,c(1:7,bias)])), 
             caption = "Table 3. Bias and its Monte Carlo Standard Error for each method",
             digits = 4, format.args = list(scientific = FALSE))
```

Table 3. Bias and its Monte Carlo Standard Error for each
method

| scenario | N | p | AUC\_0 | r | pm | mech | bias AUC CCA | MCE bias AUC CCA | bias AUC CONV | MCE bias AUC CONV | bias AUC mi\_ | MCE bias AUC mi\_ | bias AUC mice\_n | MCE bias AUC mice\_n | bias AUC mice\_p | MCE bias AUC mice\_p | bias AUC IPL LG | MCE bias AUC IPL LG | bias AUC IPL NP | MCE bias AUC IPL NP |
| --- | --- | --- | --- | --- | --- | --- | --- | --- | --- | --- | --- | --- | --- | --- | --- | --- | --- | --- | --- | --- |
| 1 | 500 | 0.3 | 0.85 | 0.5 | 0.1 | MCAR | -0.0026 | 0.0019 | 0.0001 | 0.0019 | -0.0031 | 0.0019 | -0.0029 | 0.0018 | -0.0032 | 0.0019 | -0.0032 | 0.0018 | -0.0033 | 0.0018 |
| 2 | 500 | 0.3 | 0.85 | 0.5 | 0.3 | MCAR | 0.0005 | 0.0022 | 0.0020 | 0.0022 | -0.0018 | 0.0022 | -0.0014 | 0.0021 | -0.0005 | 0.0021 | -0.0014 | 0.0022 | -0.0014 | 0.0022 |
| 3 | 500 | 0.3 | 0.85 | 0.5 | 0.5 | MCAR | -0.0013 | 0.0028 | -0.0006 | 0.0024 | -0.0048 | 0.0025 | -0.0050 | 0.0024 | -0.0046 | 0.0025 | -0.0052 | 0.0026 | -0.0073 | 0.0033 |
| 4 | 500 | 0.3 | 0.85 | 0.5 | 0.1 | MAR | -0.0021 | 0.0017 | 0.0041 | 0.0017 | 0.0008 | 0.0017 | 0.0012 | 0.0017 | 0.0010 | 0.0017 | -0.0021 | 0.0017 | -0.0021 | 0.0020 |
| 5 | 500 | 0.3 | 0.85 | 0.5 | 0.3 | MAR | -0.0102 | 0.0025 | 0.0004 | 0.0023 | -0.0034 | 0.0023 | -0.0040 | 0.0022 | -0.0039 | 0.0023 | -0.0148 | 0.0033 | -0.0120 | 0.0031 |
| 6 | 500 | 0.3 | 0.85 | 0.5 | 0.5 | MAR | 0.0005 | 0.0028 | 0.0073 | 0.0025 | 0.0013 | 0.0029 | 0.0031 | 0.0023 | -0.0003 | 0.0027 | -0.0061 | 0.0041 | -0.0083 | 0.0042 |
| 7 | 500 | 0.3 | 0.85 | 0.5 | 0.1 | MNAR | -0.0071 | 0.0020 | -0.0003 | 0.0018 | -0.0033 | 0.0018 | -0.0036 | 0.0018 | -0.0034 | 0.0018 | -0.0044 | 0.0019 | -0.0055 | 0.0020 |
| 8 | 500 | 0.3 | 0.85 | 0.5 | 0.3 | MNAR | -0.0151 | 0.0022 | -0.0043 | 0.0019 | -0.0101 | 0.0021 | -0.0088 | 0.0019 | -0.0098 | 0.0021 | -0.0107 | 0.0020 | -0.0121 | 0.0022 |
| 9 | 500 | 0.3 | 0.85 | 0.5 | 0.5 | MNAR | -0.0180 | 0.0036 | -0.0062 | 0.0029 | -0.0124 | 0.0031 | -0.0116 | 0.0028 | -0.0117 | 0.0031 | -0.0150 | 0.0033 | -0.0138 | 0.0033 |

```
# show table for RMSE 
mse <- grep("MSE", names(dat_table), value = FALSE)
knitr::kable(dat_table[,c(1:7,mse)], "simple", 
             col.names = gsub("[.]", " ", names(dat_table[,c(1:7,mse)])),
             caption = "Table 4. Rot mean squared error and its Monte Carlo Standard Error for each method",
             digits = 4, format.args = list(scientific = FALSE))
```

Table 4. Rot mean squared error and its Monte Carlo Standard
Error for each method

| scenario | N | p | AUC\_0 | r | pm | mech | RMSE AUC CCA | MCE MSE AUC CCA | RMSE AUC CONV | MCE MSE AUC CONV | RMSE AUC mi\_ | MCE MSE AUC mi\_ | RMSE AUC mice\_n | MCE MSE AUC mice\_n | RMSE AUC mice\_p | MCE MSE AUC mice\_p | RMSE AUC IPL LG | MCE MSE AUC IPL LG | RMSE AUC IPL NP | MCE MSE AUC IPL NP |
| --- | --- | --- | --- | --- | --- | --- | --- | --- | --- | --- | --- | --- | --- | --- | --- | --- | --- | --- | --- | --- |
| 1 | 500 | 0.3 | 0.85 | 0.5 | 0.1 | MCAR | 0.0188 | 0.0000 | 0.0185 | 0.0000 | 0.0190 | 0.0000 | 0.0185 | 0.0000 | 0.0189 | 0.0000 | 0.0187 | 0.0000 | 0.0184 | 0.0000 |
| 2 | 500 | 0.3 | 0.85 | 0.5 | 0.3 | MCAR | 0.0223 | 0.0001 | 0.0217 | 0.0001 | 0.0220 | 0.0001 | 0.0211 | 0.0001 | 0.0212 | 0.0001 | 0.0217 | 0.0001 | 0.0217 | 0.0001 |
| 3 | 500 | 0.3 | 0.85 | 0.5 | 0.5 | MCAR | 0.0276 | 0.0001 | 0.0244 | 0.0001 | 0.0253 | 0.0001 | 0.0248 | 0.0001 | 0.0252 | 0.0001 | 0.0265 | 0.0001 | 0.0333 | 0.0004 |
| 4 | 500 | 0.3 | 0.85 | 0.5 | 0.1 | MAR | 0.0174 | 0.0000 | 0.0175 | 0.0000 | 0.0169 | 0.0000 | 0.0167 | 0.0000 | 0.0167 | 0.0000 | 0.0173 | 0.0000 | 0.0202 | 0.0001 |
| 5 | 500 | 0.3 | 0.85 | 0.5 | 0.3 | MAR | 0.0264 | 0.0001 | 0.0225 | 0.0001 | 0.0231 | 0.0001 | 0.0220 | 0.0001 | 0.0228 | 0.0001 | 0.0364 | 0.0004 | 0.0332 | 0.0003 |
| 6 | 500 | 0.3 | 0.85 | 0.5 | 0.5 | MAR | 0.0274 | 0.0001 | 0.0257 | 0.0001 | 0.0293 | 0.0001 | 0.0227 | 0.0001 | 0.0265 | 0.0001 | 0.0411 | 0.0007 | 0.0425 | 0.0007 |
| 7 | 500 | 0.3 | 0.85 | 0.5 | 0.1 | MNAR | 0.0207 | 0.0001 | 0.0180 | 0.0000 | 0.0184 | 0.0000 | 0.0187 | 0.0000 | 0.0186 | 0.0000 | 0.0193 | 0.0000 | 0.0206 | 0.0001 |
| 8 | 500 | 0.3 | 0.85 | 0.5 | 0.3 | MNAR | 0.0267 | 0.0001 | 0.0197 | 0.0001 | 0.0234 | 0.0001 | 0.0211 | 0.0001 | 0.0233 | 0.0001 | 0.0230 | 0.0001 | 0.0247 | 0.0001 |
| 9 | 500 | 0.3 | 0.85 | 0.5 | 0.5 | MNAR | 0.0399 | 0.0002 | 0.0296 | 0.0001 | 0.0334 | 0.0002 | 0.0299 | 0.0001 | 0.0329 | 0.0002 | 0.0362 | 0.0002 | 0.0358 | 0.0002 |

```
# show table for empSE
empse <- grep("empSE", names(dat_table), value = FALSE)
knitr::kable(dat_table[,c(1:7,empse)], "simple", 
             col.names = gsub("[.]", " ", names(dat_table[,c(1:7,empse)])),
             caption = "Table 5. Empirical Standard Error and its Monte Carlo Standard Error for each method",
             digits = 4, format.args = list(scientific = FALSE))
```

Table 5. Empirical Standard Error and its Monte Carlo Standard
Error for each method

| scenario | N | p | AUC\_0 | r | pm | mech | empSE AUC CCA | MCE empSE AUC CCA | empSE AUC CONV | MCE empSE AUC CONV | empSE AUC mi\_ | MCE empSE AUC mi\_ | empSE AUC mice\_n | MCE empSE AUC mice\_n | empSE AUC mice\_p | MCE empSE AUC mice\_p | empSE AUC IPL LG | MCE empSE AUC IPL LG | empSE AUC IPL NP | MCE empSE AUC IPL NP |
| --- | --- | --- | --- | --- | --- | --- | --- | --- | --- | --- | --- | --- | --- | --- | --- | --- | --- | --- | --- | --- |
| 1 | 500 | 0.3 | 0.85 | 0.5 | 0.1 | MCAR | 0.0187 | 0.0013 | 0.0186 | 0.0013 | 0.0188 | 0.0013 | 0.0184 | 0.0013 | 0.0187 | 0.0013 | 0.0185 | 0.0013 | 0.0182 | 0.0013 |
| 2 | 500 | 0.3 | 0.85 | 0.5 | 0.3 | MCAR | 0.0224 | 0.0016 | 0.0217 | 0.0015 | 0.0220 | 0.0016 | 0.0211 | 0.0015 | 0.0213 | 0.0015 | 0.0218 | 0.0015 | 0.0218 | 0.0015 |
| 3 | 500 | 0.3 | 0.85 | 0.5 | 0.5 | MCAR | 0.0277 | 0.0020 | 0.0245 | 0.0017 | 0.0249 | 0.0018 | 0.0244 | 0.0017 | 0.0249 | 0.0018 | 0.0261 | 0.0019 | 0.0326 | 0.0023 |
| 4 | 500 | 0.3 | 0.85 | 0.5 | 0.1 | MAR | 0.0173 | 0.0012 | 0.0171 | 0.0012 | 0.0169 | 0.0012 | 0.0167 | 0.0012 | 0.0168 | 0.0012 | 0.0172 | 0.0012 | 0.0202 | 0.0014 |
| 5 | 500 | 0.3 | 0.85 | 0.5 | 0.3 | MAR | 0.0245 | 0.0017 | 0.0226 | 0.0016 | 0.0229 | 0.0016 | 0.0217 | 0.0015 | 0.0226 | 0.0016 | 0.0335 | 0.0024 | 0.0311 | 0.0022 |
| 6 | 500 | 0.3 | 0.85 | 0.5 | 0.5 | MAR | 0.0275 | 0.0020 | 0.0248 | 0.0018 | 0.0295 | 0.0021 | 0.0226 | 0.0016 | 0.0266 | 0.0019 | 0.0409 | 0.0029 | 0.0419 | 0.0030 |
| 7 | 500 | 0.3 | 0.85 | 0.5 | 0.1 | MNAR | 0.0196 | 0.0014 | 0.0181 | 0.0013 | 0.0182 | 0.0013 | 0.0185 | 0.0013 | 0.0184 | 0.0013 | 0.0189 | 0.0013 | 0.0199 | 0.0014 |
| 8 | 500 | 0.3 | 0.85 | 0.5 | 0.3 | MNAR | 0.0221 | 0.0016 | 0.0193 | 0.0014 | 0.0212 | 0.0015 | 0.0193 | 0.0014 | 0.0212 | 0.0015 | 0.0204 | 0.0015 | 0.0217 | 0.0015 |
| 9 | 500 | 0.3 | 0.85 | 0.5 | 0.5 | MNAR | 0.0358 | 0.0025 | 0.0291 | 0.0021 | 0.0312 | 0.0022 | 0.0277 | 0.0020 | 0.0309 | 0.0022 | 0.0332 | 0.0024 | 0.0332 | 0.0024 |

```
# show table for coverage
cov <- grep("cov", names(dat_table), value = FALSE)
knitr::kable(dat_table[,c(1:7,cov)], "simple", 
             col.names = gsub("[.]", " ", names(dat_table[,c(1:7,cov)])),
             caption = "Table 6. Coverage and its Monte Carlo Standard Error for each method",
             digits = 4, format.args = list(scientific = FALSE))
```

Table 6. Coverage and its Monte Carlo Standard Error for each
method

| scenario | N | p | AUC\_0 | r | pm | mech | cov AUC CCA | MCE cov AUC CCA | cov\_be AUC CCA | MCE cov\_be AUC CCA | cov AUC CONV | MCE cov AUC CONV | cov\_be AUC CONV | MCE cov\_be AUC CONV | cov AUC mi\_ | MCE cov AUC mi\_ | cov\_be AUC mi\_ | MCE cov\_be AUC mi\_ | cov AUC mice\_n | MCE cov AUC mice\_n | cov\_be AUC mice\_n | MCE cov\_be AUC mice\_n | cov AUC mice\_p | MCE cov AUC mice\_p | cov\_be AUC mice\_p | MCE cov\_be AUC mice\_p | cov AUC IPL LG | MCE cov AUC IPL LG | cov\_be AUC IPL LG | MCE cov\_be AUC IPL LG | cov AUC IPL NP | MCE cov AUC IPL NP | cov\_be AUC IPL NP | MCE cov\_be AUC IPL NP |
| --- | --- | --- | --- | --- | --- | --- | --- | --- | --- | --- | --- | --- | --- | --- | --- | --- | --- | --- | --- | --- | --- | --- | --- | --- | --- | --- | --- | --- | --- | --- | --- | --- | --- | --- |
| 1 | 500 | 0.3 | 0.85 | 0.5 | 0.1 | MCAR | 0.94 | 0.0237 | 1.00 | 0.0000 | 0.98 | 0.0140 | 1.00 | 0.0000 | 0.95 | 0.0218 | 1.00 | 0.0000 | 0.95 | 0.0218 | 1.00 | 0.0000 | 0.96 | 0.0196 | 1.00 | 0.0000 | 0.99 | 0.0099 | 1.00 | 0.0000 | 1.00 | 0.0000 | 1.00 | 0.0000 |
| 2 | 500 | 0.3 | 0.85 | 0.5 | 0.3 | MCAR | 0.91 | 0.0286 | 0.94 | 0.0237 | 0.95 | 0.0218 | 0.94 | 0.0237 | 0.94 | 0.0237 | 0.94 | 0.0237 | 0.95 | 0.0218 | 0.94 | 0.0237 | 0.95 | 0.0218 | 0.95 | 0.0218 | 0.94 | 0.0237 | 0.94 | 0.0237 | 0.95 | 0.0218 | 0.94 | 0.0237 |
| 3 | 500 | 0.3 | 0.85 | 0.5 | 0.5 | MCAR | 0.96 | 0.0196 | 0.86 | 0.0347 | 0.90 | 0.0300 | 0.86 | 0.0347 | 0.94 | 0.0237 | 0.87 | 0.0336 | 0.94 | 0.0237 | 0.87 | 0.0336 | 0.96 | 0.0196 | 0.87 | 0.0336 | 0.87 | 0.0336 | 0.86 | 0.0347 | 0.85 | 0.0357 | 0.87 | 0.0336 |
| 4 | 500 | 0.3 | 0.85 | 0.5 | 0.1 | MAR | 0.96 | 0.0196 | 0.96 | 0.0196 | 0.96 | 0.0196 | 0.98 | 0.0140 | 0.95 | 0.0218 | 0.97 | 0.0171 | 0.96 | 0.0196 | 0.97 | 0.0171 | 0.96 | 0.0196 | 0.97 | 0.0171 | 0.98 | 0.0140 | 0.96 | 0.0196 | 0.97 | 0.0171 | 0.96 | 0.0196 |
| 5 | 500 | 0.3 | 0.85 | 0.5 | 0.3 | MAR | 0.96 | 0.0196 | 0.85 | 0.0357 | 0.93 | 0.0255 | 0.89 | 0.0313 | 0.90 | 0.0300 | 0.87 | 0.0336 | 0.94 | 0.0237 | 0.87 | 0.0336 | 0.93 | 0.0255 | 0.87 | 0.0336 | 0.87 | 0.0336 | 0.86 | 0.0347 | 0.89 | 0.0313 | 0.86 | 0.0347 |
| 6 | 500 | 0.3 | 0.85 | 0.5 | 0.5 | MAR | 0.94 | 0.0237 | 0.85 | 0.0357 | 0.89 | 0.0313 | 0.84 | 0.0367 | 0.90 | 0.0300 | 0.85 | 0.0357 | 0.98 | 0.0140 | 0.86 | 0.0347 | 0.94 | 0.0237 | 0.85 | 0.0357 | 0.87 | 0.0336 | 0.81 | 0.0392 | 0.85 | 0.0357 | 0.80 | 0.0400 |
| 7 | 500 | 0.3 | 0.85 | 0.5 | 0.1 | MNAR | 0.98 | 0.0140 | 0.95 | 0.0218 | 0.98 | 0.0140 | 0.97 | 0.0171 | 0.96 | 0.0196 | 0.97 | 0.0171 | 0.97 | 0.0171 | 0.96 | 0.0196 | 0.96 | 0.0196 | 0.97 | 0.0171 | 0.98 | 0.0140 | 0.96 | 0.0196 | 0.97 | 0.0171 | 0.95 | 0.0218 |
| 8 | 500 | 0.3 | 0.85 | 0.5 | 0.3 | MNAR | 0.96 | 0.0196 | 0.93 | 0.0255 | 0.98 | 0.0140 | 0.95 | 0.0218 | 0.94 | 0.0237 | 0.96 | 0.0196 | 0.97 | 0.0171 | 0.96 | 0.0196 | 0.93 | 0.0255 | 0.96 | 0.0196 | 0.95 | 0.0218 | 0.96 | 0.0196 | 0.94 | 0.0237 | 0.96 | 0.0196 |
| 9 | 500 | 0.3 | 0.85 | 0.5 | 0.5 | MNAR | 0.89 | 0.0313 | 0.78 | 0.0414 | 0.86 | 0.0347 | 0.76 | 0.0427 | 0.83 | 0.0376 | 0.78 | 0.0414 | 0.94 | 0.0237 | 0.78 | 0.0414 | 0.89 | 0.0313 | 0.78 | 0.0414 | 0.76 | 0.0427 | 0.78 | 0.0414 | 0.77 | 0.0421 | 0.78 | 0.0414 |

```
# show table for power
power <- grep("power", names(dat_table), value = FALSE)
knitr::kable(dat_table[,c(1:7,power)], "simple", 
             col.names = gsub("[.]", " ", names(dat_table[,c(1:7,power)])),
             caption = "Table 7. Power and its Monte Carlo Standard Error for each method",
             digits = 4, format.args = list(scientific = FALSE))
```

Table 7. Power and its Monte Carlo Standard Error for each
method

| scenario | N | p | AUC\_0 | r | pm | mech | power AUC CCA | MCE power AUC CCA | power AUC CONV | MCE power AUC CONV | power AUC mi\_ | MCE power AUC mi\_ | power AUC mice\_n | MCE power AUC mice\_n | power AUC mice\_p | MCE power AUC mice\_p | power AUC IPL LG | MCE power AUC IPL LG | power AUC IPL NP | MCE power AUC IPL NP |
| --- | --- | --- | --- | --- | --- | --- | --- | --- | --- | --- | --- | --- | --- | --- | --- | --- | --- | --- | --- | --- |
| 1 | 500 | 0.3 | 0.85 | 0.5 | 0.1 | MCAR | 0.88 | 0.0325 | 0.91 | 0.0286 | 0.90 | 0.0300 | 0.90 | 0.0300 | 0.90 | 0.0300 | 0.87 | 0.0336 | 0.87 | 0.0336 |
| 2 | 500 | 0.3 | 0.85 | 0.5 | 0.3 | MCAR | 0.83 | 0.0376 | 0.89 | 0.0313 | 0.81 | 0.0392 | 0.85 | 0.0357 | 0.87 | 0.0336 | 0.84 | 0.0367 | 0.85 | 0.0357 |
| 3 | 500 | 0.3 | 0.85 | 0.5 | 0.5 | MCAR | 0.58 | 0.0494 | 0.79 | 0.0407 | 0.67 | 0.0470 | 0.66 | 0.0474 | 0.64 | 0.0480 | 0.72 | 0.0449 | 0.71 | 0.0454 |
| 4 | 500 | 0.3 | 0.85 | 0.5 | 0.1 | MAR | 0.89 | 0.0313 | 0.92 | 0.0271 | 0.93 | 0.0255 | 0.93 | 0.0255 | 0.92 | 0.0271 | 0.88 | 0.0325 | 0.86 | 0.0347 |
| 5 | 500 | 0.3 | 0.85 | 0.5 | 0.3 | MAR | 0.62 | 0.0485 | 0.83 | 0.0376 | 0.77 | 0.0421 | 0.78 | 0.0414 | 0.77 | 0.0421 | 0.65 | 0.0477 | 0.67 | 0.0470 |
| 6 | 500 | 0.3 | 0.85 | 0.5 | 0.5 | MAR | 0.64 | 0.0480 | 0.89 | 0.0313 | 0.78 | 0.0414 | 0.74 | 0.0439 | 0.68 | 0.0466 | 0.78 | 0.0414 | 0.79 | 0.0407 |
| 7 | 500 | 0.3 | 0.85 | 0.5 | 0.1 | MNAR | 0.73 | 0.0444 | 0.86 | 0.0347 | 0.84 | 0.0367 | 0.84 | 0.0367 | 0.85 | 0.0357 | 0.77 | 0.0421 | 0.74 | 0.0439 |
| 8 | 500 | 0.3 | 0.85 | 0.5 | 0.3 | MNAR | 0.50 | 0.0500 | 0.81 | 0.0392 | 0.61 | 0.0488 | 0.72 | 0.0449 | 0.69 | 0.0462 | 0.70 | 0.0458 | 0.67 | 0.0470 |
| 9 | 500 | 0.3 | 0.85 | 0.5 | 0.5 | MNAR | 0.36 | 0.0480 | 0.73 | 0.0444 | 0.59 | 0.0492 | 0.48 | 0.0500 | 0.55 | 0.0497 | 0.63 | 0.0483 | 0.61 | 0.0488 |

```
# save results in excel file
write_xlsx(scenario_res, path = "./Analyse/Ergebnisse/res_suppl.xlsx")
```

Table 8. Overview of average running time (in seconds) summarized
across all scenarios

```
# table with running time summarized across all scenarios 
time <- grep("time", names(dat_table), value = TRUE)
summary(tableby( ~ ., data = dat_table[,c(time)], control = mycontrols), pfootnote = T)
```

|  | Overall (N=9) |
| --- | --- |
| **Average running time for CCA** |  |
| Mean | 0.004 |
| SD | 0.000 |
| Median (Q1, Q3) | 0.004 (0.004, 0.004) |
| Range | 0.004 - 0.004 |
| **Average running time for CONV** |  |
| Mean | 31.678 |
| SD | 0.140 |
| Median (Q1, Q3) | 31.630 (31.600, 31.764) |
| Range | 31.512 - 31.933 |
| **Average running time for mi** |  |
| Mean | 4.272 |
| SD | 0.234 |
| Median (Q1, Q3) | 4.323 (4.072, 4.449) |
| Range | 3.942 - 4.572 |
| **Average running time for mice (norm)** |  |
| Mean | 1.400 |
| SD | 0.013 |
| Median (Q1, Q3) | 1.395 (1.392, 1.413) |
| Range | 1.382 - 1.417 |
| **Average running time for mice (pmm)** |  |
| Mean | 2.137 |
| SD | 0.026 |
| Median (Q1, Q3) | 2.136 (2.124, 2.157) |
| Range | 2.099 - 2.177 |
| **Average running time for IPL LG** |  |
| Mean | 1175.769 |
| SD | 17.211 |
| Median (Q1, Q3) | 1177.281 (1164.833, 1187.410) |
| Range | 1143.066 - 1200.861 |
| **Average running time for IPL NP** |  |
| Mean | 1175.769 |
| SD | 17.211 |
| Median (Q1, Q3) | 1177.281 (1164.833, 1187.410) |
| Range | 1143.066 - 1200.861 |

There are no missing values in the estimation of the AUC

## 4.2 Graphical display of performance results

### 4.2.1 Bias

```
# reshape from wide to long (only one column for bias and estimated AUC, respectively)
performparam <- list(auc_vars,names1)
performnames <- c("AUC","Bias")
dat_fig <- raw_res[,c("scenario","N","p","AUC_0","r","pm","mech",auc_vars,names1)]
dat_fig$id <- seq_along(1:nrow(dat_fig))
dat_long <- reshape(dat_fig, varying=performparam, v.names = performnames, times = methods, 
                    idvar = "id", direction = "long")
dat_long$scenario <- as.factor(dat_long$scenario)
colnames(dat_long)[colnames(dat_long) == "time"] <- "Method" # rename method variable
```

```
plot_bias <- ggplot(dat_long, aes(x = Method, y = Bias)) +
                          geom_violin(fill = colors[1], trim=FALSE) +
                          stat_summary(fun=mean, geom="point", size=2, color=colors[6]) +
                          xlab("Method") + ylab("Bias") +
                          geom_hline(yintercept=0) +
                          scale_y_continuous(limits = c(-0.15, 0.1)) +
                          #scale_y_continuous(breaks = c(-0.1,-0.05,0,0.05,0.1), limits = c(-0.125,0.1)) +
                          theme(axis.text.x = element_text(angle = 45, hjust = 1)) +
                          facet_grid(pm ~ mech, labeller = label_both) +
                          ggtitle("Figure 1. Violin plot of bias") +
                          theme(axis.title = element_text(size = 18),
                              axis.text = element_text(size = 16),
                              plot.title = element_text(size = 20),
                              strip.text.x = element_text(size = 14),
                              strip.text.y = element_text(size = 14))
plot_bias
```

```
# NOTE: the IPL methods cannot be shown fully as the y-axis is cut at -0.2
```

NOTE: the IPL methods cannot be shown fully as the y-axis is cut at
-0.2

```
# reshape summary results for further plots
performparam <- list(names1,names2,names3,names4,names13,names5,names6,names7,names8,names9,names14,names10,names11)
performnames <- c("Bias","RMSE","empirical SE","Coverage","be_coverage",
                  "Power","MCE_bias","MCE_MSE","MCE_empSE","MCE_cov", "MCE_be_cov", "MCE_power", "av_time")
scenario_res$id <- seq_along(1:nrow(scenario_res))
res_long <- reshape(scenario_res, varying=performparam, v.names = performnames, times = methods, 
                    idvar = "id", direction = "long")
colnames(res_long)[colnames(res_long) == "time"] <- "Method" # rename method variable
```

```
## plots for summary performance measures ##

plot_bias2 <- ggplot(res_long, aes(x = pm, y = abs(Bias), group = Method)) +
                      geom_line(aes(color=Method, linetype = Method), linewidth=1) +
                      scale_color_manual(values = colors) +
                      scale_x_continuous(breaks = c(0.1,0.3,0.5)) +
                      xlab("Proportion of missing values") + ylab("Absolute bias") +
                      facet_grid(~ mech, labeller = label_both) + 
                      ggtitle("Figure 2. Mean absolute bias") +
                      theme(axis.title = element_text(size = 18),
                              axis.text = element_text(size = 16),
                              plot.title = element_text(size = 20),
                              strip.text.x = element_text(size = 14),
                              strip.text.y = element_text(size = 14))
plot_bias2
```

### 4.2.2 Root mean squared error (RMSE)

```
## plots for summary performance measures ##

plot_rmse <- ggplot(res_long, aes(x = pm, y = RMSE, group = Method)) +
                      geom_line(aes(color=Method, linetype = Method), linewidth=1) +
                      scale_color_manual(values = colors) +
                      scale_x_continuous(breaks = c(0.1,0.3,0.5)) +
                      xlab("Proportion of missing values") + ylab("Root mean squared error") +
                      facet_grid(~ mech, labeller = label_both) + 
                      ggtitle("Figure 3. RMSE") +
                      theme(axis.title = element_text(size = 18),
                              axis.text = element_text(size = 16),
                              plot.title = element_text(size = 20),
                              strip.text.x = element_text(size = 14),
                              strip.text.y = element_text(size = 14))
plot_rmse
```

### 4.2.3 Coverage probability

```
# calculate 95% Monte carlo CI for coverage
res_long$MC_cov_ciu <- res_long$Coverage+1.96*res_long$MCE_cov
res_long$MC_cov_cil <- res_long$Coverage-1.96*res_long$MCE_cov

# variable indicating whether bias is "too high" (bias>5%)
res_long$rel_bias <- (res_long$Bias/res_long$AUC_0)*100 # relative bias in %
res_long$bias_cut <- as.factor(if_else(res_long$rel_bias>=5 | res_long$rel_bias<=(-5), "too biased (>=5%)", "acceptable biased"))

plot_conv <- ggplot(res_long, aes(y=Method, x=Coverage, color=bias_cut)) +
                      geom_segment( aes(y=Method, yend=Method, x=0.95, xend=Coverage)) +
                      geom_point(size=2) +
                      geom_vline(xintercept=0.95) +
                      geom_text(aes(MC_cov_ciu, Method, label = ")")) +
                      geom_text(aes(MC_cov_cil, Method, label = "(")) +
                      facet_grid(pm ~ mech, labeller = label_both) + # pm~p
                      scale_color_grey() +
                      ggtitle("Figure 4. Coverage probability") +
                      labs(color="Bias categorized") +
                      theme(axis.title = element_text(size = 18),
                              axis.text = element_text(size = 16),
                              plot.title = element_text(size = 20),
                              strip.text.x = element_text(size = 14),
                              strip.text.y = element_text(size = 14),
                            legend.position = "bottom")
plot_conv
```

### 4.2.4 Power

```
plot_power <- ggplot(res_long, aes(x = pm, y = Power, group = Method)) +
                            geom_line(aes(color=Method, linetype=Method), linewidth=1) +
                            scale_x_continuous(breaks = c(0.1,0.3,0.5)) +
                            scale_color_manual(values = colors) +
                            xlab("Proportion of missing values") + ylab("Power") +
                            facet_grid( ~ mech, labeller = label_both) +
                            ggtitle("Figure 5. Power") +
                            theme(axis.title = element_text(size = 18),
                              axis.text = element_text(size = 16),
                              plot.title = element_text(size = 20),
                              strip.text.x = element_text(size = 14),
                              strip.text.y = element_text(size = 14))
plot_power
```

## 4.3 Overview of Monte Carlo Errors

Table 9. Summary statistics of Monte Carlo Standard Errors summarized
across all scenarios and iterations

```
summary(tableby(Method ~ ., data = res_long[,c("Method", "MCE_bias","MCE_MSE", "MCE_cov", "MCE_power")], control = mycontrols), pfootnote = T)
```

|  | CCA (N=9) | CONV (N=9) | IPL.LG (N=9) | IPL.NP (N=9) | mi\_ (N=9) | mice\_n (N=9) | mice\_p (N=9) | Total (N=63) |
| --- | --- | --- | --- | --- | --- | --- | --- | --- |
| **MCE\_bias** |  |  |  |  |  |  |  |  |
| Mean | 0.002 | 0.002 | 0.003 | 0.003 | 0.002 | 0.002 | 0.002 | 0.002 |
| SD | 0.001 | 0.000 | 0.001 | 0.001 | 0.000 | 0.000 | 0.000 | 0.001 |
| Median (Q1, Q3) | 0.002 (0.002, 0.003) | 0.002 (0.002, 0.002) | 0.002 (0.002, 0.003) | 0.002 (0.002, 0.003) | 0.002 (0.002, 0.002) | 0.002 (0.002, 0.002) | 0.002 (0.002, 0.002) | 0.002 (0.002, 0.003) |
| Range | 0.002 - 0.004 | 0.002 - 0.003 | 0.002 - 0.004 | 0.002 - 0.004 | 0.002 - 0.003 | 0.002 - 0.003 | 0.002 - 0.003 | 0.002 - 0.004 |
| **MCE\_MSE** |  |  |  |  |  |  |  |  |
| Mean | 0.000 | 0.000 | 0.000 | 0.000 | 0.000 | 0.000 | 0.000 | 0.000 |
| SD | 0.000 | 0.000 | 0.000 | 0.000 | 0.000 | 0.000 | 0.000 | 0.000 |
| Median (Q1, Q3) | 0.000 (0.000, 0.000) | 0.000 (0.000, 0.000) | 0.000 (0.000, 0.000) | 0.000 (0.000, 0.000) | 0.000 (0.000, 0.000) | 0.000 (0.000, 0.000) | 0.000 (0.000, 0.000) | 0.000 (0.000, 0.000) |
| Range | 0.000 - 0.000 | 0.000 - 0.000 | 0.000 - 0.001 | 0.000 - 0.001 | 0.000 - 0.000 | 0.000 - 0.000 | 0.000 - 0.000 | 0.000 - 0.001 |
| **MCE\_cov** |  |  |  |  |  |  |  |  |
| Mean | 0.022 | 0.023 | 0.025 | 0.025 | 0.026 | 0.020 | 0.023 | 0.023 |
| SD | 0.005 | 0.008 | 0.011 | 0.013 | 0.006 | 0.004 | 0.004 | 0.008 |
| Median (Q1, Q3) | 0.020 (0.020, 0.024) | 0.022 (0.014, 0.030) | 0.024 (0.014, 0.034) | 0.024 (0.017, 0.036) | 0.024 (0.022, 0.030) | 0.022 (0.017, 0.024) | 0.022 (0.020, 0.026) | 0.022 (0.020, 0.029) |
| Range | 0.014 - 0.031 | 0.014 - 0.035 | 0.010 - 0.043 | 0.000 - 0.042 | 0.020 - 0.038 | 0.014 - 0.024 | 0.020 - 0.031 | 0.000 - 0.043 |
| **MCE\_power** |  |  |  |  |  |  |  |  |
| Mean | 0.043 | 0.035 | 0.041 | 0.042 | 0.040 | 0.039 | 0.040 | 0.040 |
| SD | 0.007 | 0.006 | 0.006 | 0.006 | 0.008 | 0.008 | 0.008 | 0.007 |
| Median (Q1, Q3) | 0.048 (0.038, 0.049) | 0.035 (0.031, 0.039) | 0.042 (0.037, 0.046) | 0.044 (0.036, 0.047) | 0.041 (0.037, 0.047) | 0.041 (0.036, 0.045) | 0.042 (0.034, 0.047) | 0.041 (0.034, 0.047) |
| Range | 0.031 - 0.050 | 0.027 - 0.044 | 0.032 - 0.048 | 0.034 - 0.049 | 0.026 - 0.049 | 0.026 - 0.050 | 0.027 - 0.050 | 0.026 - 0.050 |

## 4.4 Nested loop plots for the “big picture”

Figure 6. Nested loop plot of bias

```
# only pm=0.5
s1 <- simsum(data = dat_long, estvarname = "AUC", ref="CCA", true = "AUC_0", methodvar = "Method", by=c("pm", "mech"))
#summary(s1)

ap <- autoplot(s1, type = "nlp", stats = "bias")
ap + scale_color_manual(values = colors)
```

```
#autoplot(s1, type = "nlp", stats = "mse")
```

# 5 References

Bianco AM, Boente G, González–Manteiga W, Pérez–González A.
Estimators for ROC curves with missing biomarkers values and informative
covariates. Statistical Methods & Applications. 2023.

Cheng W, Tang N. Smoothed empirical likelihood inference for ROC
curve in the presence of missing biomarker values. Biom J.
2020;62(4):1038-59.

Robin X, Turck N, Hainard A, Tiberti N, Lisacek F, Sanchez J, et
al. pROC: an open-source package for R and S+ to analyze and compare ROC
curves. BMC Bioinformatics. 2011(12):77.

Rubin DB. Multiple Imputation for Nonresponse in Surveys. New York:
John Wiley & Sons, Inc.; 1987.

Su Y-S, Gelman A, Hill J, Yajima M. Multiple Imputation with
Diagnostics (mi) in R: Opening Windows into the Black Box. Journal of
Statistical Software. 2011;45(2):1 - 31.

van Buuren S. Flexible Imputation of Missing Data. 2nd ed. New York:
Chapman and Hall/CRC; 2018.

van Buuren S, Groothuis-Oudshoorn K. mice: Multivariate Imputation by
Chained Equations in R. Journal of Statistical Software. 2011;45(3):1 -
67.

# 6 Session info

```
sessioninfo::session_info()
```

```
## ─ Session info ───────────────────────────────────────────────────────────────
##  setting  value
##  version  R version 4.4.1 (2024-06-14 ucrt)
##  os       Windows 10 x64 (build 19045)
##  system   x86_64, mingw32
##  ui       RTerm
##  language (EN)
##  collate  German_Germany.utf8
##  ctype    German_Germany.utf8
##  tz       Europe/Berlin
##  date     2025-04-25
##  pandoc   3.2 @ C:/Program Files/RStudio/resources/app/bin/quarto/bin/tools/ (via rmarkdown)
## 
## ─ Packages ───────────────────────────────────────────────────────────────────
##  package      * version date (UTC) lib source
##  abind          1.4-8   2024-09-12 [1] CRAN (R 4.4.1)
##  arsenal      * 3.6.3   2021-06-04 [1] CRAN (R 4.4.1)
##  backports      1.5.0   2024-05-23 [1] CRAN (R 4.4.0)
##  broom          1.0.6   2024-05-17 [1] CRAN (R 4.4.1)
##  bslib          0.8.0   2024-07-29 [1] CRAN (R 4.4.1)
##  cachem         1.1.0   2024-05-16 [1] CRAN (R 4.4.1)
##  car            3.1-2   2023-03-30 [1] CRAN (R 4.4.1)
##  carData        3.0-5   2022-01-06 [1] CRAN (R 4.4.1)
##  checkmate      2.3.2   2024-07-29 [1] CRAN (R 4.4.1)
##  cli            3.6.3   2024-06-21 [1] CRAN (R 4.4.1)
##  colorspace     2.1-1   2024-07-26 [1] CRAN (R 4.4.1)
##  digest         0.6.37  2024-08-19 [1] CRAN (R 4.4.1)
##  dplyr        * 1.1.4   2023-11-17 [1] CRAN (R 4.4.1)
##  evaluate       1.0.3   2025-01-10 [1] CRAN (R 4.4.3)
##  fansi          1.0.6   2023-12-08 [1] CRAN (R 4.4.1)
##  farver         2.1.2   2024-05-13 [1] CRAN (R 4.4.1)
##  fastmap        1.2.0   2024-05-15 [1] CRAN (R 4.4.1)
##  generics       0.1.3   2022-07-05 [1] CRAN (R 4.4.1)
##  ggplot2      * 3.5.1   2024-04-23 [1] CRAN (R 4.4.1)
##  ggpubr       * 0.6.0   2023-02-10 [1] CRAN (R 4.4.1)
##  ggridges       0.5.6   2024-01-23 [1] CRAN (R 4.4.1)
##  ggsignif       0.6.4   2022-10-13 [1] CRAN (R 4.4.1)
##  glue           1.7.0   2024-01-09 [1] CRAN (R 4.4.1)
##  gt           * 0.11.0  2024-07-09 [1] CRAN (R 4.4.1)
##  gtable         0.3.5   2024-04-22 [1] CRAN (R 4.4.1)
##  highr          0.11    2024-05-26 [1] CRAN (R 4.4.1)
##  htmltools      0.5.8.1 2024-04-04 [1] CRAN (R 4.4.1)
##  insight        0.20.4  2024-09-01 [1] CRAN (R 4.4.1)
##  jquerylib      0.1.4   2021-04-26 [1] CRAN (R 4.4.1)
##  jsonlite       1.8.8   2023-12-04 [1] CRAN (R 4.4.1)
##  knitr          1.48    2024-07-07 [1] CRAN (R 4.4.1)
##  labeling       0.4.3   2023-08-29 [1] CRAN (R 4.4.0)
##  lifecycle      1.0.4   2023-11-07 [1] CRAN (R 4.4.1)
##  magrittr       2.0.3   2022-03-30 [1] CRAN (R 4.4.1)
##  munsell        0.5.1   2024-04-01 [1] CRAN (R 4.4.1)
##  pillar         1.9.0   2023-03-22 [1] CRAN (R 4.4.1)
##  pkgconfig      2.0.3   2019-09-22 [1] CRAN (R 4.4.1)
##  purrr          1.0.2   2023-08-10 [1] CRAN (R 4.4.1)
##  R6             2.5.1   2021-08-19 [1] CRAN (R 4.4.1)
##  RColorBrewer * 1.1-3   2022-04-03 [1] CRAN (R 4.4.0)
##  rlang          1.1.4   2024-06-04 [1] CRAN (R 4.4.1)
##  rmarkdown      2.28    2024-08-17 [1] CRAN (R 4.4.1)
##  rprojroot      2.0.4   2023-11-05 [1] CRAN (R 4.4.2)
##  rsimsum      * 0.13.0  2024-03-03 [1] CRAN (R 4.4.1)
##  rstatix        0.7.2   2023-02-01 [1] CRAN (R 4.4.1)
##  rstudioapi     0.16.0  2024-03-24 [1] CRAN (R 4.4.1)
##  sass           0.4.9   2024-03-15 [1] CRAN (R 4.4.1)
##  scales         1.3.0   2023-11-28 [1] CRAN (R 4.4.1)
##  sessioninfo    1.2.2   2021-12-06 [1] CRAN (R 4.4.1)
##  sjlabelled     1.2.0   2022-04-10 [1] CRAN (R 4.4.1)
##  tibble         3.2.1   2023-03-20 [1] CRAN (R 4.4.1)
##  tidyr          1.3.1   2024-01-24 [1] CRAN (R 4.4.1)
##  tidyselect     1.2.1   2024-03-11 [1] CRAN (R 4.4.1)
##  utf8           1.2.4   2023-10-22 [1] CRAN (R 4.4.1)
##  vctrs          0.6.5   2023-12-01 [1] CRAN (R 4.4.1)
##  withr          3.0.2   2024-10-28 [1] CRAN (R 4.4.3)
##  writexl      * 1.5.0   2024-02-09 [1] CRAN (R 4.4.1)
##  xfun           0.47    2024-08-17 [1] CRAN (R 4.4.1)
##  xml2           1.3.6   2023-12-04 [1] CRAN (R 4.4.1)
##  yaml           2.3.10  2024-07-26 [1] CRAN (R 4.4.1)
## 
##  [1] C:/Users/stahlmann/AppData/Local/R/win-library/4.4
##  [2] C:/Program Files/R/R-4.4.1/library
## 
## ──────────────────────────────────────────────────────────────────────────────
```
