# Supplementary material for: Comparison of methods to handle missing values in a continuous index test in a diagnostic accuracy study – a simulation study
Source: BMC Med Res Methodol. 2025 May 27;25:147. doi: 10.1186/s12874-025-02594-2 (PMC12107930; doi:10.1186/s12874-025-02594-2)
Supplement: Supplementary file 2 — Additional file 2. Methods to handle missing values in a continuous index test in a diagnostic study – supplemental MNAR scenarios [file 12874_2025_2594_MOESM2_ESM.html]

Methods to handle missing values in a continuous index test in a diagnostic study – supplemental mnar scenarios (Additional file 2)


Code 

- Show All Code
- Hide All Code

# Methods to handle missing values in a continuous index test in a diagnostic study – supplemental mnar scenarios (Additional file 2)

#### Katharina Stahlmann, University Medical Center Hamburg-Eppendorf Institute of Medical Biometry and Epidemiology, k.stahlmann@uke.de - Bastiaan Kellerhuis, Julius Center for Health Sciences and Primary Care, University Medical Center Utrecht, Utrecht University, Utrecht, The Netherlands; Blavatnik School of Government, University of Oxford, Oxford, UK - Johannes B. Reitsma, Julius Center for Health Sciences and Primary Care, University Medical Center Utrecht, Utrecht University, Utrecht, The Netherlands - Nandini Dendukuri, Department of Medicine, McGill University, Montreal, Canada - Antonia Zapf, University Medical Center Hamburg-Eppendorf Institute of Medical Biometry and Epidemiology

#### 2025-04-25

```
knitr::opts_knit$set(root.dir = rprojroot::find_rstudio_root_file())
                       
# set global chunk options
knitr::opts_chunk$set(echo = TRUE, message = FALSE, warning = FALSE,
                      fig.width = 12,
                       fig.asp = 0.8 ,
                       out.width = "120%")

# this options for word export
#knitr::opts_chunk$set(echo = FALSE, message = FALSE, warning = FALSE)

options(stringsAsFactors = F)
# always show NA is there is any in the table function
table = function (..., useNA = 'ifany') base::table(..., useNA = useNA)
```

# 1 Settings

## 1.1 Packages

```
library(ggplot2)
library(writexl)
library(ggpubr)
library(RColorBrewer)
library(dplyr)
library(arsenal)
library(rsimsum)
library(gt)
library(gtsummary)
```

## 1.2 load functions

```
mycontrols = tableby.control(numeric.stats=c("Nmiss","mean", "sd", "medianq1q3", "range"),
                             cat.stats=c("Nmiss", "countpct"), 
                             stats.labels=list(Nmiss='Missing values', medianq1q3='Median (Q1, Q3)'),
                             test = F)

colors <- brewer.pal(8, "Dark2")
```

# 2 Simulation

## 2.1 Set up simulation parameters

```
nsim <- 100

#define simulation scenarios
grid = expand.grid(
  sim = 1:nsim 
  , N = c(500, 1000)
  , p = c(0.1, 0.3, 0.5)
  , AUC_0 = c(0.85)
  , r = c(0.5)
  , pm = c(0.3)
  , mech = c("MNAR")
  , type = c("MID")
)


# methods to be compared
methods <- c("CCA", "MI2", "MIB2", "HDEL", "KER", "mice", "mix", "AIPW")


Abbreviation = names(grid)
setup <- data.frame(
  Abbreviation = names(grid),
  Parameter = c("Number of simulations", "Sample size", "prevalence of the target condition", "True AUC", "Correlation between index test and covariates", "proportion of missing values", "Missingness Mechanism", "Type"),
  Values = c(
    nsim,
    sapply(Abbreviation[2:length(Abbreviation)], function(x) (paste(unique(grid[,x]), collapse = ',')))
  )
)  
setup %>%
  gt() %>% 
  tab_style(style = cell_text(weight = "bold"), locations = cells_column_labels(columns=c("Abbreviation", "Parameter", "Values"))) %>%
  tab_header(
    title = "Table 1. Overview of simulation parameter"
  )
```

|  |  |  |
| --- | --- | --- |
| Table 1. Overview of simulation parameter | | |
| Abbreviation | Parameter | Values |
| sim | Number of simulations | 100 |
| N | Sample size | 500,1000 |
| p | prevalence of the target condition | 0.1,0.3,0.5 |
| AUC\_0 | True AUC | 0.85 |
| r | Correlation between index test and covariates | 0.5 |
| pm | proportion of missing values | 0.3 |
| mech | Missingness Mechanism | MNAR |
| type | Type | MID |

Table 2. Overview of methods

| Methods |  |
| --- | --- |
| CCA | Complete case analysis |
| MI2 | Multiple Imputation using prediction and propensity score (Long et al. 2011a) |
| MIB2 | MI2 + bootstrap step for calculating confidence intervals (Long et al. 2011a) |
| HDEL | Hot Deck Empirical Likelihood Approach (Wang and Qin 2012, 2014) |
| KER | Kernel-based Inverse Probability Weighting (Bianco et al. 2023) |
| mice | Multiple Imputation using chained equations (van Buuren et al. 2011) |
| mix | Multiple Imputation using joint modelling (Schafer 2022) |
| AIPW | Augmented Inverse Probability Weighting (Long et al. 2011b) |

The seed for the parallel loop was set to 5273 and to 4730xi for data
generation.

## 2.2 Conduct the simulation

```
# due to time reasons the simulation is not conducted but the data are loaded in the following chunk
source("./Analyse/Simulation_mnar.R")
```

Load the simulation results

```
name <- "results_mnar"
load(file = paste0("./Analyse/hpc/Simulation_data/", name, ".Rdata"))


res <- res %>%
  rename(r = korr) %>%
  select(-type)
```

# 3 Calculate Performance parameter

The following performance parameter will be calculated: number of
missing values, bias, root mean squared error, empirical standard
deviation, coverage, bias-eliminated coverage and power as well as the
respective monte carlo standard errors for each performance
parameter.

```
fix_col <- c("N","p","AUC_0","r", "pm","mech", "AUC_min","scenario")
source("Analyse/Simulation_performance3.R")
# input: res (file with simulation results as dataframe)
# out: raw_res (res + step 1 of performance calculation -> 1 row for each simulation run), scenario_res (dataframe with performance parameters, -> 1 row for each scenario aggregated over the runs)
```

# 4 Results

## 4.1 Table of performance parameter

```
# rearrange the scenario_res table columns, so that performance estimate and its MC standard error are located side by side
vars=list()
for (i in 1:length(methods)){
  vars_i <- grep(methods[i], names(scenario_res), value = T)
  vars[[i]] <- vars_i
}
vars_order <- unlist(vars)
dat_table1 <- scenario_res[,c(8,1:6)]
dat_table2 <- scenario_res[,vars_order]
dat_table <- cbind(dat_table1, dat_table2)
rownames(dat_table) <- NULL

# label selected variables in dat_table
dat_table <- dat_table %>%
  sjlabelled::var_labels(
    scenario = "Scenario",
    N = "Sample size",
    p = "Prevalence of target condition",
    AUC_0 = "True AUC",
    r = "Correlation",
    pm = "Proportion of missing values",
    mech = "Missingness mechanism",
    av.time.AUC.CCA = "Average running time for CCA",
    av.time.AUC.MI2 = "Average running time for MI2",
    av.time.AUC.MIB2 = "Average running time for MIB2",
    av.time.AUC.HDEL = "Average running time for HDEL",
    av.time.AUC.mice = "Average running time for mice",
    av.time.AUC.mix = "Average running time for mix",
    av.time.AUC.AIPW = "Average running time for AIPW",
    av.time.AUC.KER = "Average running time for KER"
  )


# show table for bias
bias <- grep("bias", names(dat_table), value = FALSE)
knitr::kable(dat_table[,c(1:7,bias)], "simple", 
             col.names = gsub("[.]", " ", names(dat_table[,c(1:7,bias)])), 
             caption = "Table 3. Bias and its Monte Carlo Standard Error for each method",
             digits = 4, format.args = list(scientific = FALSE))
```

Table 3. Bias and its Monte Carlo Standard Error for each
method

| scenario | N | p | AUC\_0 | r | pm | mech | bias AUC CCA | MCE bias AUC CCA | bias AUC MI2 | MCE bias AUC MI2 | bias AUC MIB2 | MCE bias AUC MIB2 | bias AUC HDEL | MCE bias AUC HDEL | bias AUC KER | MCE bias AUC KER | bias AUC mice | MCE bias AUC mice | bias AUC mix | MCE bias AUC mix | bias AUC AIPW | MCE bias AUC AIPW |
| --- | --- | --- | --- | --- | --- | --- | --- | --- | --- | --- | --- | --- | --- | --- | --- | --- | --- | --- | --- | --- | --- | --- |
| 1 | 500 | 0.1 | 0.85 | 0.5 | 0.3 | MNAR | 0.0085 | 0.0033 | 0.0024 | 0.0034 | 0.0033 | 0.0034 | 0.0089 | 0.0035 | -0.0010 | 0.0034 | -0.0014 | 0.0032 | -0.0017 | 0.0032 | -0.0004 | 0.0032 |
| 2 | 1000 | 0.1 | 0.85 | 0.5 | 0.3 | MNAR | 0.0121 | 0.0018 | 0.0056 | 0.0022 | 0.0049 | 0.0024 | 0.0123 | 0.0020 | 0.0045 | 0.0021 | 0.0027 | 0.0020 | 0.0025 | 0.0019 | 0.0038 | 0.0022 |
| 3 | 500 | 0.3 | 0.85 | 0.5 | 0.3 | MNAR | 0.0188 | 0.0020 | 0.0080 | 0.0021 | 0.0098 | 0.0021 | 0.0188 | 0.0020 | 0.0065 | 0.0020 | 0.0080 | 0.0020 | 0.0074 | 0.0019 | 0.0074 | 0.0020 |
| 4 | 1000 | 0.3 | 0.85 | 0.5 | 0.3 | MNAR | 0.0196 | 0.0013 | 0.0118 | 0.0015 | 0.0106 | 0.0015 | 0.0196 | 0.0015 | 0.0101 | 0.0014 | 0.0105 | 0.0015 | 0.0103 | 0.0014 | 0.0099 | 0.0014 |
| 5 | 500 | 0.5 | 0.85 | 0.5 | 0.3 | MNAR | 0.0171 | 0.0021 | 0.0092 | 0.0022 | 0.0100 | 0.0022 | 0.0187 | 0.0022 | 0.0058 | 0.0021 | 0.0085 | 0.0020 | 0.0081 | 0.0021 | 0.0073 | 0.0020 |
| 6 | 1000 | 0.5 | 0.85 | 0.5 | 0.3 | MNAR | 0.0232 | 0.0013 | 0.0127 | 0.0014 | 0.0135 | 0.0014 | 0.0233 | 0.0015 | 0.0115 | 0.0014 | 0.0126 | 0.0013 | 0.0128 | 0.0013 | 0.0118 | 0.0013 |

```
# show table for RMSE 
mse <- grep("MSE", names(dat_table), value = FALSE)
knitr::kable(dat_table[,c(1:7,mse)], "simple", 
             col.names = gsub("[.]", " ", names(dat_table[,c(1:7,mse)])),
             caption = "Table 4. Rot mean squared error and its Monte Carlo Standard Error for each method",
             digits = 4, format.args = list(scientific = FALSE))
```

Table 4. Rot mean squared error and its Monte Carlo Standard
Error for each method

| scenario | N | p | AUC\_0 | r | pm | mech | RMSE AUC CCA | MCE MSE AUC CCA | RMSE AUC MI2 | MCE MSE AUC MI2 | RMSE AUC MIB2 | MCE MSE AUC MIB2 | RMSE AUC HDEL | MCE MSE AUC HDEL | RMSE AUC KER | MCE MSE AUC KER | RMSE AUC mice | MCE MSE AUC mice | RMSE AUC mix | MCE MSE AUC mix | RMSE AUC AIPW | MCE MSE AUC AIPW |
| --- | --- | --- | --- | --- | --- | --- | --- | --- | --- | --- | --- | --- | --- | --- | --- | --- | --- | --- | --- | --- | --- | --- |
| 1 | 500 | 0.1 | 0.85 | 0.5 | 0.3 | MNAR | 0.0337 | 0.0002 | 0.0342 | 0.0002 | 0.0343 | 0.0001 | 0.0360 | 0.0002 | 0.0337 | 0.0002 | 0.0316 | 0.0001 | 0.0322 | 0.0001 | 0.0320 | 0.0001 |
| 2 | 1000 | 0.1 | 0.85 | 0.5 | 0.3 | MNAR | 0.0218 | 0.0001 | 0.0230 | 0.0001 | 0.0245 | 0.0001 | 0.0232 | 0.0001 | 0.0213 | 0.0001 | 0.0202 | 0.0001 | 0.0195 | 0.0001 | 0.0217 | 0.0001 |
| 3 | 500 | 0.3 | 0.85 | 0.5 | 0.3 | MNAR | 0.0273 | 0.0001 | 0.0226 | 0.0001 | 0.0234 | 0.0001 | 0.0275 | 0.0001 | 0.0210 | 0.0001 | 0.0215 | 0.0001 | 0.0208 | 0.0001 | 0.0210 | 0.0001 |
| 4 | 1000 | 0.3 | 0.85 | 0.5 | 0.3 | MNAR | 0.0237 | 0.0001 | 0.0187 | 0.0000 | 0.0180 | 0.0000 | 0.0246 | 0.0001 | 0.0175 | 0.0000 | 0.0179 | 0.0000 | 0.0172 | 0.0000 | 0.0172 | 0.0000 |
| 5 | 500 | 0.5 | 0.85 | 0.5 | 0.3 | MNAR | 0.0269 | 0.0001 | 0.0235 | 0.0001 | 0.0244 | 0.0001 | 0.0291 | 0.0001 | 0.0212 | 0.0001 | 0.0220 | 0.0001 | 0.0224 | 0.0001 | 0.0216 | 0.0001 |
| 6 | 1000 | 0.5 | 0.85 | 0.5 | 0.3 | MNAR | 0.0263 | 0.0001 | 0.0185 | 0.0000 | 0.0192 | 0.0000 | 0.0278 | 0.0001 | 0.0177 | 0.0000 | 0.0179 | 0.0000 | 0.0181 | 0.0000 | 0.0175 | 0.0000 |

```
# show table for empSE
empse <- grep("empSE", names(dat_table), value = FALSE)
knitr::kable(dat_table[,c(1:7,empse)], "simple", 
             col.names = gsub("[.]", " ", names(dat_table[,c(1:7,empse)])),
             caption = "Table 5. Empirical Standard Error and its Monte Carlo Standard Error for each method",
             digits = 4, format.args = list(scientific = FALSE))
```

Table 5. Empirical Standard Error and its Monte Carlo Standard
Error for each method

| scenario | N | p | AUC\_0 | r | pm | mech | empSE AUC CCA | MCE empSE AUC CCA | empSE AUC MI2 | MCE empSE AUC MI2 | empSE AUC MIB2 | MCE empSE AUC MIB2 | empSE AUC HDEL | MCE empSE AUC HDEL | empSE AUC KER | MCE empSE AUC KER | empSE AUC mice | MCE empSE AUC mice | empSE AUC mix | MCE empSE AUC mix | empSE AUC AIPW | MCE empSE AUC AIPW |
| --- | --- | --- | --- | --- | --- | --- | --- | --- | --- | --- | --- | --- | --- | --- | --- | --- | --- | --- | --- | --- | --- | --- |
| 1 | 500 | 0.1 | 0.85 | 0.5 | 0.3 | MNAR | 0.0328 | 0.0023 | 0.0343 | 0.0024 | 0.0343 | 0.0024 | 0.0350 | 0.0025 | 0.0339 | 0.0024 | 0.0318 | 0.0023 | 0.0324 | 0.0023 | 0.0321 | 0.0023 |
| 2 | 1000 | 0.1 | 0.85 | 0.5 | 0.3 | MNAR | 0.0183 | 0.0013 | 0.0224 | 0.0016 | 0.0241 | 0.0017 | 0.0197 | 0.0014 | 0.0210 | 0.0015 | 0.0201 | 0.0014 | 0.0195 | 0.0014 | 0.0215 | 0.0015 |
| 3 | 500 | 0.3 | 0.85 | 0.5 | 0.3 | MNAR | 0.0199 | 0.0014 | 0.0213 | 0.0015 | 0.0213 | 0.0015 | 0.0202 | 0.0014 | 0.0200 | 0.0014 | 0.0201 | 0.0014 | 0.0195 | 0.0014 | 0.0198 | 0.0014 |
| 4 | 1000 | 0.3 | 0.85 | 0.5 | 0.3 | MNAR | 0.0133 | 0.0009 | 0.0146 | 0.0010 | 0.0146 | 0.0010 | 0.0150 | 0.0011 | 0.0143 | 0.0010 | 0.0146 | 0.0010 | 0.0139 | 0.0010 | 0.0141 | 0.0010 |
| 5 | 500 | 0.5 | 0.85 | 0.5 | 0.3 | MNAR | 0.0209 | 0.0015 | 0.0217 | 0.0015 | 0.0224 | 0.0016 | 0.0224 | 0.0016 | 0.0205 | 0.0015 | 0.0204 | 0.0015 | 0.0211 | 0.0015 | 0.0205 | 0.0015 |
| 6 | 1000 | 0.5 | 0.85 | 0.5 | 0.3 | MNAR | 0.0126 | 0.0009 | 0.0135 | 0.0010 | 0.0137 | 0.0010 | 0.0152 | 0.0011 | 0.0136 | 0.0010 | 0.0128 | 0.0009 | 0.0128 | 0.0009 | 0.0129 | 0.0009 |

```
# show table for coverage
cov <- grep("cov", names(dat_table), value = FALSE)
knitr::kable(dat_table[,c(1:7,cov)], "simple", 
             col.names = gsub("[.]", " ", names(dat_table[,c(1:7,cov)])),
             caption = "Table 6. Coverage and its Monte Carlo Standard Error for each method",
             digits = 4, format.args = list(scientific = FALSE))
```

Table 6. Coverage and its Monte Carlo Standard Error for each
method

| scenario | N | p | AUC\_0 | r | pm | mech | cov AUC CCA | MCE cov AUC CCA | cov\_be AUC CCA | MCE cov\_be AUC CCA | cov AUC MI2 | MCE cov AUC MI2 | cov\_be AUC MI2 | MCE cov\_be AUC MI2 | cov AUC MIB2 | MCE cov AUC MIB2 | cov\_be AUC MIB2 | MCE cov\_be AUC MIB2 | cov AUC HDEL | MCE cov AUC HDEL | cov\_be AUC HDEL | MCE cov\_be AUC HDEL | cov AUC KER | MCE cov AUC KER | cov\_be AUC KER | MCE cov\_be AUC KER | cov AUC mice | MCE cov AUC mice | cov\_be AUC mice | MCE cov\_be AUC mice | cov AUC mix | MCE cov AUC mix | cov\_be AUC mix | MCE cov\_be AUC mix | cov AUC AIPW | MCE cov AUC AIPW | cov\_be AUC AIPW | MCE cov\_be AUC AIPW |
| --- | --- | --- | --- | --- | --- | --- | --- | --- | --- | --- | --- | --- | --- | --- | --- | --- | --- | --- | --- | --- | --- | --- | --- | --- | --- | --- | --- | --- | --- | --- | --- | --- | --- | --- | --- | --- | --- | --- |
| 1 | 500 | 0.1 | 0.85 | 0.5 | 0.3 | MNAR | 0.90 | 0.0300 | 0.93 | 0.0255 | 0.88 | 0.0325 | 0.94 | 0.0237 | 0.96 | 0.0196 | 0.94 | 0.0237 | 0.99 | 0.0099 | 0.93 | 0.0255 | 0.96 | 0.0196 | 0.95 | 0.0218 | 0.94 | 0.0237 | 0.95 | 0.0218 | 0.93 | 0.0255 | 0.95 | 0.0218 | 0.95 | 0.0218 | 0.95 | 0.0218 |
| 2 | 1000 | 0.1 | 0.85 | 0.5 | 0.3 | MNAR | 0.89 | 0.0313 | 0.96 | 0.0196 | 0.86 | 0.0347 | 0.92 | 0.0271 | 0.96 | 0.0196 | 0.92 | 0.0271 | 0.99 | 0.0099 | 0.96 | 0.0196 | 0.95 | 0.0218 | 0.92 | 0.0271 | 0.95 | 0.0218 | 0.92 | 0.0271 | 0.94 | 0.0237 | 0.92 | 0.0271 | 0.90 | 0.0300 | 0.92 | 0.0271 |
| 3 | 500 | 0.3 | 0.85 | 0.5 | 0.3 | MNAR | 0.84 | 0.0367 | 0.95 | 0.0218 | 0.87 | 0.0336 | 0.97 | 0.0171 | 0.97 | 0.0171 | 0.96 | 0.0196 | 1.00 | 0.0000 | 0.95 | 0.0218 | 0.94 | 0.0237 | 0.97 | 0.0171 | 0.92 | 0.0271 | 0.97 | 0.0171 | 0.95 | 0.0218 | 0.97 | 0.0171 | 0.93 | 0.0255 | 0.97 | 0.0171 |
| 4 | 1000 | 0.3 | 0.85 | 0.5 | 0.3 | MNAR | 0.70 | 0.0458 | 0.93 | 0.0255 | 0.83 | 0.0376 | 0.95 | 0.0218 | 0.97 | 0.0171 | 0.95 | 0.0218 | 0.98 | 0.0140 | 0.93 | 0.0255 | 0.89 | 0.0313 | 0.95 | 0.0218 | 0.90 | 0.0300 | 0.95 | 0.0218 | 0.89 | 0.0313 | 0.95 | 0.0218 | 0.89 | 0.0313 | 0.95 | 0.0218 |
| 5 | 500 | 0.5 | 0.85 | 0.5 | 0.3 | MNAR | 0.78 | 0.0414 | 0.93 | 0.0255 | 0.83 | 0.0376 | 0.93 | 0.0255 | 0.95 | 0.0218 | 0.94 | 0.0237 | 1.00 | 0.0000 | 0.93 | 0.0255 | 0.86 | 0.0347 | 0.91 | 0.0286 | 0.87 | 0.0336 | 0.93 | 0.0255 | 0.87 | 0.0336 | 0.93 | 0.0255 | 0.88 | 0.0325 | 0.93 | 0.0255 |
| 6 | 1000 | 0.5 | 0.85 | 0.5 | 0.3 | MNAR | 0.52 | 0.0500 | 0.88 | 0.0325 | 0.77 | 0.0421 | 0.97 | 0.0171 | 0.91 | 0.0286 | 0.97 | 0.0171 | 0.99 | 0.0099 | 0.88 | 0.0325 | 0.75 | 0.0433 | 0.97 | 0.0171 | 0.83 | 0.0376 | 0.97 | 0.0171 | 0.84 | 0.0367 | 0.97 | 0.0171 | 0.84 | 0.0367 | 0.97 | 0.0171 |

```
# show table for power
power <- grep("power", names(dat_table), value = FALSE)
knitr::kable(dat_table[,c(1:7,power)], "simple", 
             col.names = gsub("[.]", " ", names(dat_table[,c(1:7,power)])),
             caption = "Table 7. Power and its Monte Carlo Standard Error for each method",
             digits = 4, format.args = list(scientific = FALSE))
```

Table 7. Power and its Monte Carlo Standard Error for each
method

| scenario | N | p | AUC\_0 | r | pm | mech | power AUC CCA | MCE power AUC CCA | power AUC MI2 | MCE power AUC MI2 | power AUC MIB2 | MCE power AUC MIB2 | power AUC HDEL | MCE power AUC HDEL | power AUC KER | MCE power AUC KER | power AUC mice | MCE power AUC mice | power AUC mix | MCE power AUC mix | power AUC AIPW | MCE power AUC AIPW |
| --- | --- | --- | --- | --- | --- | --- | --- | --- | --- | --- | --- | --- | --- | --- | --- | --- | --- | --- | --- | --- | --- | --- |
| 1 | 500 | 0.1 | 0.85 | 0.5 | 0.3 | MNAR | 0.88 | 0.0325 | 0.85 | 0.0357 | 0.62 | 0.0485 | 0.05 | 0.0218 | 0.79 | 0.0407 | 0.82 | 0.0384 | 0.82 | 0.0384 | 0.77 | 0.0421 |
| 2 | 1000 | 0.1 | 0.85 | 0.5 | 0.3 | MNAR | 0.96 | 0.0196 | 0.92 | 0.0271 | 0.72 | 0.0449 | 0.08 | 0.0271 | 0.87 | 0.0336 | 0.90 | 0.0300 | 0.89 | 0.0313 | 0.87 | 0.0336 |
| 3 | 500 | 0.3 | 0.85 | 0.5 | 0.3 | MNAR | 0.98 | 0.0140 | 0.94 | 0.0237 | 0.71 | 0.0454 | 0.06 | 0.0237 | 0.92 | 0.0271 | 0.92 | 0.0271 | 0.93 | 0.0255 | 0.92 | 0.0271 |
| 4 | 1000 | 0.3 | 0.85 | 0.5 | 0.3 | MNAR | 0.97 | 0.0171 | 0.96 | 0.0196 | 0.84 | 0.0367 | 0.10 | 0.0300 | 0.95 | 0.0218 | 0.95 | 0.0218 | 0.95 | 0.0218 | 0.95 | 0.0218 |
| 5 | 500 | 0.5 | 0.85 | 0.5 | 0.3 | MNAR | 0.93 | 0.0255 | 0.93 | 0.0255 | 0.69 | 0.0462 | 0.05 | 0.0218 | 0.92 | 0.0271 | 0.88 | 0.0325 | 0.87 | 0.0336 | 0.87 | 0.0336 |
| 6 | 1000 | 0.5 | 0.85 | 0.5 | 0.3 | MNAR | 0.99 | 0.0099 | 0.96 | 0.0196 | 0.85 | 0.0357 | 0.05 | 0.0218 | 0.96 | 0.0196 | 0.98 | 0.0140 | 0.97 | 0.0171 | 0.96 | 0.0196 |

```
# save results in excel file
write_xlsx(scenario_res, path = "./Analyse/Ergebnisse/res_suppl_mnar.xlsx")
```

Table 8. Overview of average running time (in seconds) summarized
across all scenarios

```
# table with running time summarized across all scenarios 
time <- grep("time", names(dat_table), value = TRUE)
summary(tableby( ~ ., data = dat_table[,c(time)], control = mycontrols), pfootnote = T)
```

|  | Overall (N=6) |
| --- | --- |
| **Average running time for CCA** |  |
| Mean | 0.009 |
| SD | 0.002 |
| Median (Q1, Q3) | 0.009 (0.007, 0.009) |
| Range | 0.006 - 0.013 |
| **Average running time for MI2** |  |
| Mean | 0.238 |
| SD | 0.061 |
| Median (Q1, Q3) | 0.222 (0.203, 0.250) |
| Range | 0.179 - 0.351 |
| **Average running time for MIB2** |  |
| Mean | 1.027 |
| SD | 0.354 |
| Median (Q1, Q3) | 0.943 (0.808, 1.115) |
| Range | 0.677 - 1.662 |
| **Average running time for HDEL** |  |
| Mean | 0.075 |
| SD | 0.012 |
| Median (Q1, Q3) | 0.074 (0.068, 0.079) |
| Range | 0.060 - 0.096 |
| **Average running time for KER** |  |
| Mean | 0.420 |
| SD | 0.249 |
| Median (Q1, Q3) | 0.326 (0.271, 0.619) |
| Range | 0.148 - 0.754 |
| **Average running time for mice** |  |
| Mean | 1.617 |
| SD | 0.261 |
| Median (Q1, Q3) | 1.571 (1.465, 1.776) |
| Range | 1.284 - 2.000 |
| **Average running time for mix** |  |
| Mean | 0.875 |
| SD | 0.194 |
| Median (Q1, Q3) | 0.822 (0.762, 0.927) |
| Range | 0.680 - 1.221 |
| **Average running time for AIPW** |  |
| Mean | 34.401 |
| SD | 19.736 |
| Median (Q1, Q3) | 28.181 (21.044, 51.788) |
| Range | 12.791 - 58.929 |

There are no missing values in the estimation of the AUC

## 4.2 Graphical display of performance results

### 4.2.1 Bias

```
# reshape from wide to long (only one column for bias and estimated AUC, respectively)
performparam <- list(auc_vars,names1)
performnames <- c("AUC","Bias")
dat_fig <- raw_res[,c("scenario","N","p","AUC_0","r","pm","mech",auc_vars,names1)]
dat_fig$id <- seq_along(1:nrow(dat_fig))
dat_long <- reshape(dat_fig, varying=performparam, v.names = performnames, times = methods, 
                    idvar = "id", direction = "long")
dat_long$scenario <- as.factor(dat_long$scenario)
colnames(dat_long)[colnames(dat_long) == "time"] <- "Method" # rename method variable
```

```
plot_bias <- ggplot(dat_long, aes(x = Method, y = Bias)) +
                          geom_violin(fill = colors[1], trim=FALSE) +
                          stat_summary(fun=mean, geom="point", size=2, color=colors[6]) +
                          xlab("Method") + ylab("Bias") +
                          geom_hline(yintercept=0) +
                          scale_y_continuous(limits = c(-0.15,0.15)) +
                          theme(axis.text.x = element_text(angle = 45, hjust = 1)) +
                          facet_grid(N ~ p, labeller = label_both) +
                          ggtitle("Figure 1. Violin plot of bias") +
                          theme(axis.title = element_text(size = 18),
                              axis.text = element_text(size = 16),
                              plot.title = element_text(size = 20),
                              strip.text.x = element_text(size = 14),
                              strip.text.y = element_text(size = 14))
plot_bias
```

```
table_b <- dat_long %>%
  filter(N==500 & (p==0.1 | p==0.5)) %>%
  select(Method, Bias,p) %>%
  tbl_strata(strata=p, .tbl_fun =  ~ .x %>% 
             tbl_summary(by=Method,
                           type = list(all_continuous()~"continuous2"),
                           statistic = list(all_continuous() ~ c("{mean} ({sd})",
                                                    "{median} ({p25}, {p75})", 
                                                    "[{min}, {max}]")),
                           digits = list(all_continuous() ~4)))

tab_b <- table_b %>%
  as_gt() %>%
  tab_header(
    title = "Table 9. Summary statistics for bias for a small sample size (N=500)"
  )
tab_b
```

|  |  |  |  |  |  |  |  |  |  |  |  |  |  |  |  |  |
| --- | --- | --- | --- | --- | --- | --- | --- | --- | --- | --- | --- | --- | --- | --- | --- | --- |
| Table 9. Summary statistics for bias for a small sample size (N=500) | | | | | | | | | | | | | | | | |
| **Characteristic** | **0.1** | | | | | | | | **0.5** | | | | | | | |
|  | **AIPW** | **CCA** | **HDEL**  N = 100 | **KER**  N = 100 | **MI2**  N = 100 | **MIB2**  N = 100 | **mice**  N = 100 | **mix**  N = 100 | **AIPW**  N = 100 | **CCA**  N = 100 | **HDEL**  N = 100 | **KER**  N = 100 | **MI2**  N = 100 | **MIB2**  N = 100 | **mice**  N = 100 | **mix**  N = 100 |
| Bias |  |  |  |  |  |  |  |  |  |  |  |  |  |  |  |  |
| Mean (SD) | -0.0004 (0.0321) | 0.0085 (0.0328) | 0.0089 (0.0350) | -0.0010 (0.0339) | 0.0024 (0.0343) | 0.0033 (0.0343) | -0.0014 (0.0318) | -0.0017 (0.0324) | 0.0073 (0.0205) | 0.0171 (0.0209) | 0.0187 (0.0224) | 0.0058 (0.0205) | 0.0092 (0.0217) | 0.0100 (0.0224) | 0.0085 (0.0204) | 0.0081 (0.0211) |
| Median (Q1, Q3) | -0.0013 (-0.0202, 0.0205) | 0.0094 (-0.0126, 0.0307) | 0.0123 (-0.0171, 0.0351) | 0.0005 (-0.0233, 0.0208) | 0.0072 (-0.0204, 0.0233) | 0.0069 (-0.0275, 0.0287) | 0.0034 (-0.0242, 0.0201) | 0.0003 (-0.0249, 0.0191) | 0.0086 (-0.0089, 0.0207) | 0.0200 (0.0026, 0.0306) | 0.0173 (0.0034, 0.0362) | 0.0073 (-0.0102, 0.0200) | 0.0111 (-0.0088, 0.0248) | 0.0121 (-0.0053, 0.0281) | 0.0115 (-0.0064, 0.0219) | 0.0102 (-0.0071, 0.0214) |
| [Min, Max] | [-0.0780, 0.0830] | [-0.0873, 0.0828] | [-0.0724, 0.0969] | [-0.0805, 0.0838] | [-0.0817, 0.0899] | [-0.0723, 0.0888] | [-0.0899, 0.0781] | [-0.0789, 0.0806] | [-0.0456, 0.0496] | [-0.0327, 0.0638] | [-0.0403, 0.0689] | [-0.0486, 0.0470] | [-0.0453, 0.0506] | [-0.0482, 0.0564] | [-0.0409, 0.0567] | [-0.0453, 0.0508] |

```
# reshape summary results for further plots
performparam <- list(names1,names2,names3,names4,names13,names5,names6,names7,names8,names9,names14,names10,names11)
performnames <- c("Bias","RMSE","empirical_SE","Coverage","be_coverage",
                  "Power","MCE_bias","MCE_MSE","MCE_empSE","MCE_cov", "MCE_be_cov", "MCE_power", "av_time")
scenario_res$id <- seq_along(1:nrow(scenario_res))
res_long <- reshape(scenario_res, varying=performparam, v.names = performnames, times = methods, 
                    idvar = "id", direction = "long")
colnames(res_long)[colnames(res_long) == "time"] <- "Method" # rename method variable
```

Comparison between additional MNAR scenarios (type=MID) and main
simulation MNAR scenarios (type=RIGHT)

```
load("./Analyse/mnar_test.RData")
mnar_test$type <- "RIGHT"

res_long$type <- "MID"
mnar_dta <- rbind(res_long, mnar_test)
```

```
comp_bias <- ggplot(mnar_dta, aes(x = p, y = Bias, group = Method)) +
                      geom_line(aes(color=Method, linetype = Method), linewidth=1) +
                      scale_color_manual(values = colors) +
                      xlab("Prevalence") + ylab("Bias") +
                      facet_grid(N ~ type, labeller = label_both) + 
                      scale_x_continuous(breaks = c(0.1,0.3,0.5)) +
                      ggtitle("Figure 2. Bias for MNAR type=mid vs. type=right (pm=0.3, r=0.5, true AUC=0.85)") +
                      theme(axis.title = element_text(size = 18),
                              axis.text = element_text(size = 16),
                              plot.title = element_text(size = 20),
                              strip.text.x = element_text(size = 14),
                              strip.text.y = element_text(size = 14))
comp_bias
```

All methods tend to overestimate the AUC under type=MID whereas they
tend to underestimate the AUC under type=RIGHT. Additionally, the bias
increases with higher prevalence given type=MID while it stays the same
or slightly decreases with higher prevalence given type=RIGHT.

### 4.2.2 Root mean squared error (RMSE)

Comparison between additional MNAR scenarios (type=MID) and main
simulation MNAR scenarios (type=RIGHT)

```
comp_rmse <- ggplot(mnar_dta, aes(x = p, y = RMSE, group = Method)) +
                      geom_line(aes(color=Method, linetype = Method), linewidth=1) +
                      scale_color_manual(values = colors) +
                      xlab("Prevalence") + ylab("Root mean squared error") +
                      facet_grid(N ~ type, labeller = label_both) + 
                      scale_x_continuous(breaks = c(0.1,0.3,0.5)) +
                      ggtitle("Figure 3. RMSE for MNAR type=mid vs. type=right (pm=0.3, r=0.5, true AUC=0.85)") +
                      theme(axis.title = element_text(size = 18),
                              axis.text = element_text(size = 16),
                              plot.title = element_text(size = 20),
                              strip.text.x = element_text(size = 14),
                              strip.text.y = element_text(size = 14))
comp_rmse
```

Regardless of method, the RMSE is higher for type=RIGHT than for
type=MID if the prevalence of the target condition is low. However,
there are no differences in RMSE between type=RIGHT and type=MID with
higher prevalence.

### 4.2.3 Coverage probability

```
# calculate 95% Monte carlo CI for coverage
res_long$MC_cov_ciu <- res_long$Coverage+1.96*res_long$MCE_cov
res_long$MC_cov_cil <- res_long$Coverage-1.96*res_long$MCE_cov

# variable indicating whether bias is "too high" (bias>5%)
res_long$rel_bias <- (res_long$Bias/res_long$AUC_0)*100 # relative bias in %
res_long$bias_cut <- as.factor(if_else(res_long$rel_bias>=5 | res_long$rel_bias<=(-5), "too biased (>=5%)", "acceptable biased"))


plot_conv <- ggplot(res_long, aes(y=Method, x=Coverage, color=bias_cut)) +
                      geom_segment( aes(y=Method, yend=Method, x=0.95, xend=Coverage)) +
                      geom_point(size=2) +
                      geom_vline(xintercept=0.95) +
                      geom_text(aes(MC_cov_ciu, Method, label = ")")) +
                      geom_text(aes(MC_cov_cil, Method, label = "(")) +
                      facet_grid(N ~ p, labeller = label_both) + # pm~p
                      scale_color_grey() +
                      ggtitle("Figure 4. Coverage probability") +
                      labs(color="Bias categorized") +
                      theme(axis.title = element_text(size = 18),
                              axis.text = element_text(size = 16),
                              plot.title = element_text(size = 20),
                              strip.text.x = element_text(size = 14),
                              strip.text.y = element_text(size = 14),
                              legend.position = "bottom")
plot_conv
```

### 4.2.4 Power

```
plot_power <- ggplot(res_long, aes(x = p, y = Power, group = Method)) +
                            geom_line(aes(color=Method, linetype=Method), linewidth=1) +
                            scale_x_continuous(breaks = c(0.1,0.3,0.5)) +
                            scale_color_manual(values = colors) +
                            xlab("Proportion of missing values") + ylab("Power") +
                            facet_grid( ~ N, labeller = label_both) +
                            ggtitle("Figure 5. Power") +
                            theme(axis.title = element_text(size = 18),
                              axis.text = element_text(size = 16),
                              plot.title = element_text(size = 20),
                              strip.text.x = element_text(size = 14),
                              strip.text.y = element_text(size = 14))
plot_power
```

## 4.3 Overview of Monte Carlo Errors

Table 10. Summary statistics of Monte Carlo Standard Errors
summarized across all scenarios and iterations

```
summary(tableby(Method ~ ., data = res_long[,c("Method", "MCE_bias","MCE_MSE", "MCE_cov", "MCE_power")], control = mycontrols), pfootnote = T)
```

|  | AIPW (N=6) | CCA (N=6) | HDEL (N=6) | KER (N=6) | MI2 (N=6) | MIB2 (N=6) | mice (N=6) | mix (N=6) | Total (N=48) |
| --- | --- | --- | --- | --- | --- | --- | --- | --- | --- |
| **MCE\_bias** |  |  |  |  |  |  |  |  |  |
| Mean | 0.002 | 0.002 | 0.002 | 0.002 | 0.002 | 0.002 | 0.002 | 0.002 | 0.002 |
| SD | 0.001 | 0.001 | 0.001 | 0.001 | 0.001 | 0.001 | 0.001 | 0.001 | 0.001 |
| Median (Q1, Q3) | 0.002 (0.002, 0.002) | 0.002 (0.001, 0.002) | 0.002 (0.002, 0.002) | 0.002 (0.002, 0.002) | 0.002 (0.002, 0.002) | 0.002 (0.002, 0.002) | 0.002 (0.002, 0.002) | 0.002 (0.002, 0.002) | 0.002 (0.001, 0.002) |
| Range | 0.001 - 0.003 | 0.001 - 0.003 | 0.001 - 0.004 | 0.001 - 0.003 | 0.001 - 0.003 | 0.001 - 0.003 | 0.001 - 0.003 | 0.001 - 0.003 | 0.001 - 0.004 |
| **MCE\_MSE** |  |  |  |  |  |  |  |  |  |
| Mean | 0.000 | 0.000 | 0.000 | 0.000 | 0.000 | 0.000 | 0.000 | 0.000 | 0.000 |
| SD | 0.000 | 0.000 | 0.000 | 0.000 | 0.000 | 0.000 | 0.000 | 0.000 | 0.000 |
| Median (Q1, Q3) | 0.000 (0.000, 0.000) | 0.000 (0.000, 0.000) | 0.000 (0.000, 0.000) | 0.000 (0.000, 0.000) | 0.000 (0.000, 0.000) | 0.000 (0.000, 0.000) | 0.000 (0.000, 0.000) | 0.000 (0.000, 0.000) | 0.000 (0.000, 0.000) |
| Range | 0.000 - 0.000 | 0.000 - 0.000 | 0.000 - 0.000 | 0.000 - 0.000 | 0.000 - 0.000 | 0.000 - 0.000 | 0.000 - 0.000 | 0.000 - 0.000 | 0.000 - 0.000 |
| **MCE\_cov** |  |  |  |  |  |  |  |  |  |
| Mean | 0.030 | 0.039 | 0.007 | 0.029 | 0.036 | 0.021 | 0.029 | 0.029 | 0.027 |
| SD | 0.005 | 0.008 | 0.006 | 0.009 | 0.003 | 0.004 | 0.006 | 0.006 | 0.011 |
| Median (Q1, Q3) | 0.031 (0.027, 0.032) | 0.039 (0.033, 0.045) | 0.010 (0.002, 0.010) | 0.028 (0.022, 0.034) | 0.036 (0.034, 0.038) | 0.020 (0.018, 0.021) | 0.029 (0.025, 0.033) | 0.028 (0.024, 0.033) | 0.030 (0.022, 0.035) |
| Range | 0.022 - 0.037 | 0.030 - 0.050 | 0.000 - 0.014 | 0.020 - 0.043 | 0.032 - 0.042 | 0.017 - 0.029 | 0.022 - 0.038 | 0.022 - 0.037 | 0.000 - 0.050 |
| **MCE\_power** |  |  |  |  |  |  |  |  |  |
| Mean | 0.030 | 0.020 | 0.024 | 0.028 | 0.025 | 0.043 | 0.027 | 0.028 | 0.028 |
| SD | 0.008 | 0.008 | 0.003 | 0.008 | 0.006 | 0.005 | 0.009 | 0.008 | 0.009 |
| Median (Q1, Q3) | 0.030 (0.023, 0.034) | 0.018 (0.015, 0.024) | 0.023 (0.022, 0.026) | 0.027 (0.023, 0.032) | 0.025 (0.021, 0.027) | 0.045 (0.039, 0.046) | 0.029 (0.023, 0.032) | 0.028 (0.023, 0.033) | 0.027 (0.022, 0.034) |
| Range | 0.020 - 0.042 | 0.010 - 0.032 | 0.022 - 0.030 | 0.020 - 0.041 | 0.020 - 0.036 | 0.036 - 0.049 | 0.014 - 0.038 | 0.017 - 0.038 | 0.010 - 0.049 |

## 4.4 Nested loop plots for the “big picture”

Figure 6. Nested loop plot for bias

```
s1 <- simsum(data = dat_long, estvarname = "AUC", ref="CCA", true = "AUC_0", methodvar = "Method", by=c("p", "N"))
#summary(s1)

ap <- autoplot(s1, type = "nlp", stats = "bias")
ap + scale_color_manual(values = colors)
```

```
#autoplot(s1, type = "nlp", stats = "mse")
```

# 5 References

Bianco AM, Boente G, González–Manteiga W, Pérez–González A.
Estimators for ROC curves with missing biomarkers values and informative
covariates. Statistical Methods & Applications. 2023.

Long Q, Zhang X, Hsu C-H. Nonparametric multiple imputation for
receiver operating characteristics analysis when some biomarker values
are missing at random. Stat Med. 2011a;30(26):3149-61.

Long Q, Zhang X, Johnson BA. Robust estimation of area under ROC
curve using auxiliary variables in the presence of missing biomarker
values. Biometrics. 2011b;67(2):559-67.

Schafer J. mix: Estimation/Multiple Imputation for Mixed Categorical
and Continuous Data. R package version 10-11. 2022.

van Buuren S, Groothuis-Oudshoorn K. mice: Multivariate Imputation by
Chained Equations in R. Journal of Statistical Software. 2011;45(3):1 -
67.

van Smeden M, Moons KG, de Groot JA, et al. Sample size for binary
logistic prediction models: Beyond events per variable criteria. Stat
Methods Med Res. 2019;28(8):2455-2474. doi:10.1177/0962280218784726

Wang B, Qin G. Imputation-based empirical likelihood inference for
the area under the ROC curve with missing data. Stat Interface.
2012;5(3):319-29.

Wang B, Qin G. Empirical likelihood-based confidence intervals for
the sensitivity of a continuous-scale diagnostic test with missing data.
Commun Stat Theory Methods. 2014;43(15):3248-68.

# 6 Session info

```
sessioninfo::session_info()
```

```
## ─ Session info ───────────────────────────────────────────────────────────────
##  setting  value
##  version  R version 4.4.1 (2024-06-14 ucrt)
##  os       Windows 10 x64 (build 19045)
##  system   x86_64, mingw32
##  ui       RTerm
##  language (EN)
##  collate  German_Germany.utf8
##  ctype    German_Germany.utf8
##  tz       Europe/Berlin
##  date     2025-04-25
##  pandoc   3.2 @ C:/Program Files/RStudio/resources/app/bin/quarto/bin/tools/ (via rmarkdown)
## 
## ─ Packages ───────────────────────────────────────────────────────────────────
##  package      * version date (UTC) lib source
##  abind          1.4-8   2024-09-12 [1] CRAN (R 4.4.1)
##  arsenal      * 3.6.3   2021-06-04 [1] CRAN (R 4.4.1)
##  backports      1.5.0   2024-05-23 [1] CRAN (R 4.4.0)
##  broom          1.0.6   2024-05-17 [1] CRAN (R 4.4.1)
##  bslib          0.8.0   2024-07-29 [1] CRAN (R 4.4.1)
##  cachem         1.1.0   2024-05-16 [1] CRAN (R 4.4.1)
##  car            3.1-2   2023-03-30 [1] CRAN (R 4.4.1)
##  carData        3.0-5   2022-01-06 [1] CRAN (R 4.4.1)
##  cards          0.2.2   2024-09-02 [1] CRAN (R 4.4.1)
##  checkmate      2.3.2   2024-07-29 [1] CRAN (R 4.4.1)
##  cli            3.6.3   2024-06-21 [1] CRAN (R 4.4.1)
##  colorspace     2.1-1   2024-07-26 [1] CRAN (R 4.4.1)
##  commonmark     1.9.1   2024-01-30 [1] CRAN (R 4.4.1)
##  digest         0.6.37  2024-08-19 [1] CRAN (R 4.4.1)
##  dplyr        * 1.1.4   2023-11-17 [1] CRAN (R 4.4.1)
##  evaluate       1.0.3   2025-01-10 [1] CRAN (R 4.4.3)
##  fansi          1.0.6   2023-12-08 [1] CRAN (R 4.4.1)
##  farver         2.1.2   2024-05-13 [1] CRAN (R 4.4.1)
##  fastmap        1.2.0   2024-05-15 [1] CRAN (R 4.4.1)
##  generics       0.1.3   2022-07-05 [1] CRAN (R 4.4.1)
##  ggplot2      * 3.5.1   2024-04-23 [1] CRAN (R 4.4.1)
##  ggpubr       * 0.6.0   2023-02-10 [1] CRAN (R 4.4.1)
##  ggridges       0.5.6   2024-01-23 [1] CRAN (R 4.4.1)
##  ggsignif       0.6.4   2022-10-13 [1] CRAN (R 4.4.1)
##  glue           1.7.0   2024-01-09 [1] CRAN (R 4.4.1)
##  gt           * 0.11.0  2024-07-09 [1] CRAN (R 4.4.1)
##  gtable         0.3.5   2024-04-22 [1] CRAN (R 4.4.1)
##  gtsummary    * 2.0.2   2024-09-05 [1] CRAN (R 4.4.1)
##  highr          0.11    2024-05-26 [1] CRAN (R 4.4.1)
##  htmltools      0.5.8.1 2024-04-04 [1] CRAN (R 4.4.1)
##  insight        0.20.4  2024-09-01 [1] CRAN (R 4.4.1)
##  jquerylib      0.1.4   2021-04-26 [1] CRAN (R 4.4.1)
##  jsonlite       1.8.8   2023-12-04 [1] CRAN (R 4.4.1)
##  knitr          1.48    2024-07-07 [1] CRAN (R 4.4.1)
##  labeling       0.4.3   2023-08-29 [1] CRAN (R 4.4.0)
##  lifecycle      1.0.4   2023-11-07 [1] CRAN (R 4.4.1)
##  magrittr       2.0.3   2022-03-30 [1] CRAN (R 4.4.1)
##  markdown       1.13    2024-06-04 [1] CRAN (R 4.4.1)
##  munsell        0.5.1   2024-04-01 [1] CRAN (R 4.4.1)
##  pillar         1.9.0   2023-03-22 [1] CRAN (R 4.4.1)
##  pkgconfig      2.0.3   2019-09-22 [1] CRAN (R 4.4.1)
##  purrr          1.0.2   2023-08-10 [1] CRAN (R 4.4.1)
##  R6             2.5.1   2021-08-19 [1] CRAN (R 4.4.1)
##  RColorBrewer * 1.1-3   2022-04-03 [1] CRAN (R 4.4.0)
##  rlang          1.1.4   2024-06-04 [1] CRAN (R 4.4.1)
##  rmarkdown      2.28    2024-08-17 [1] CRAN (R 4.4.1)
##  rprojroot      2.0.4   2023-11-05 [1] CRAN (R 4.4.2)
##  rsimsum      * 0.13.0  2024-03-03 [1] CRAN (R 4.4.1)
##  rstatix        0.7.2   2023-02-01 [1] CRAN (R 4.4.1)
##  rstudioapi     0.16.0  2024-03-24 [1] CRAN (R 4.4.1)
##  sass           0.4.9   2024-03-15 [1] CRAN (R 4.4.1)
##  scales         1.3.0   2023-11-28 [1] CRAN (R 4.4.1)
##  sessioninfo    1.2.2   2021-12-06 [1] CRAN (R 4.4.1)
##  sjlabelled     1.2.0   2022-04-10 [1] CRAN (R 4.4.1)
##  tibble         3.2.1   2023-03-20 [1] CRAN (R 4.4.1)
##  tidyr          1.3.1   2024-01-24 [1] CRAN (R 4.4.1)
##  tidyselect     1.2.1   2024-03-11 [1] CRAN (R 4.4.1)
##  utf8           1.2.4   2023-10-22 [1] CRAN (R 4.4.1)
##  vctrs          0.6.5   2023-12-01 [1] CRAN (R 4.4.1)
##  withr          3.0.2   2024-10-28 [1] CRAN (R 4.4.3)
##  writexl      * 1.5.0   2024-02-09 [1] CRAN (R 4.4.1)
##  xfun           0.47    2024-08-17 [1] CRAN (R 4.4.1)
##  xml2           1.3.6   2023-12-04 [1] CRAN (R 4.4.1)
##  yaml           2.3.10  2024-07-26 [1] CRAN (R 4.4.1)
## 
##  [1] C:/Users/stahlmann/AppData/Local/R/win-library/4.4
##  [2] C:/Program Files/R/R-4.4.1/library
## 
## ──────────────────────────────────────────────────────────────────────────────
```
