# Supplementary material for: Comparison of methods to handle missing values in a continuous index test in a diagnostic accuracy study – a simulation study
Source: BMC Med Res Methodol. 2025 May 27;25:147. doi: 10.1186/s12874-025-02594-2 (PMC12107930; doi:10.1186/s12874-025-02594-2)
Supplement: Supplementary file 3 — Additional file 3. Results of the case study [file 12874_2025_2594_MOESM3_ESM.html]

Modified case study - Additional file 3


Code 

- Show All Code
- Hide All Code

# Modified case study - Additional file 3

#### Katharina Stahlmann, University Medical Center Hamburg-Eppendorf Institute of Medical Biometry and Epidemiology, k.stahlmann@uke.de,

#### Bastiaan Kellerhuis, Julius Center for Health Sciences and Primary Care, University Medical Center Utrecht, Utrecht University, Utrecht, The Netherlands; Blavatnik School of Government, University of Oxford, Oxford, UK

#### Johannes B. Reitsma, Julius Center for Health Sciences and Primary Care, University Medical Center Utrecht, Utrecht University, Utrecht, The Netherlands

#### Nandini Dendukuri, Department of Medicine, McGill University, Montreal, Canada

#### Antonia Zapf, University Medical Center Hamburg-Eppendorf Institute of Medical Biometry and Epidemiology

#### 2025-04-25

# 1 Settings

## 1.1 Packages

```
library(mice)
library(pROC)
library(mitml)
library(mix)
library(mi)
library(ggplot2)
library(writexl)
library(ggpubr)
library(haven)
library(dplyr)
library(gt)
library(arsenal)
library(ks)
```

## 1.2 load functions

```
source("./Case study/functions_AUC_ROC.R")

# set global controls for tableby command
mycontrols = tableby.control(numeric.stats=c("Nmiss","mean", "sd", "medianq1q3", "range"),
                             cat.stats=c("Nmiss", "countpct"), 
                             stats.labels=list(Nmiss='Missing values', medianq1q3='Median (Q1, Q3)'),
                             test = F)
```

# 2 Methods compared in this modified case study

Table 1. Overview of methods

| Methods |  |
| --- | --- |
| CCA | Complete case analysis |
| HDEL | Hot Deck Empirical Likelihood Approach (Wang and Qin 2012, 2014) |
| MI2 | Multiple Imputation using prediction and propensity score (Long et al. 2011a) |
| MIB2 | MI2 + bootstrap step for calculating confidence intervals (Long et al. 2011a) |
| mice | Multiple Imputation using chained equations (van Buuren et al. 2011) |
| mix | Multiple Imputation using joint modelling (Schafer 2022) |
| KER | Kernel-based Inverse Probability Weighting (Bianco et al. 2023) |
| AIPW | Augmented Inverse Probability Weighting (Long et al. 2011b) |
| CONV | Convolution-based approach (Bianco et al. 2023) |
| mi | Multiple Imputation using chained equations with predictive mean matching (PMM) (Su et al. 2022) |

# 3 Load and prepare dataset

The dataset nnhs2.dta from the Diagnostic and Biomarkers Statistical
(DABS) Center is used. A detailed description of this study can be found
in Norton et al. (2000).

```
nnhs2 <- read_dta("./Case study/nnhs2.dta")

# ear: 1=left 2=right
# sitenum: site
# currage: corrected age
# d: disease (reference test)
# y1: DPOAE
# y2: TEOAE
# y3: ABR

summary(nnhs2)
table(nnhs2$d)
```

The data are restricted to the left year, as the methods are not
designed for clustered data. In this subset, missing values (n=1) occur
only in the variable currage, neither in the index test(s) nor reference
standard. These observations are excluded from the analysis.

```
# generate original dataset without any missing values
# use only left ear (=1) (no clustered data)
data_full <- nnhs2 %>%
  filter(ear==1 & !is.na(currage)) %>%
  mutate(
    id = as_factor(id),
    ear = as_factor(ear),
    sitenum = as_factor(sitenum),
    gender = as_factor(gender),
    d = as_factor(d)
  )

attr(data_full$gender,'label')  <- 'Gender'
attr(data_full$currage,'label')  <- 'Age corrected for premature birth'
attr(data_full$sitenum,'label') <- 'Site (i.e. hospital)'

# generate dataset with missing values
# a priori: define missingness pattern
mypattern <- matrix(nrow = 1, ncol = 9)
mypattern[1, ] <- c(1,1,1,1,1,1,0,1,1) # only y1 (DPOAE) is missing
```

Table 2. Descriptive overview of complete dataset for the total
sample and stratified by reference test (hearing impaired yes/no)

```
summary(tableby(d ~ ., data = data_full[,c("sitenum", "currage", "gender", "d", "y1")], control = mycontrols), pfootnote = T)
```

|  | no (N=2460) | yes (N=80) | Total (N=2540) |
| --- | --- | --- | --- |
| **Site (i.e. hospital)** |  |  |  |
| 1 | 675 (27.4%) | 10 (12.5%) | 685 (27.0%) |
| 2 | 132 (5.4%) | 5 (6.2%) | 137 (5.4%) |
| 3 | 113 (4.6%) | 7 (8.8%) | 120 (4.7%) |
| 4 | 912 (37.1%) | 33 (41.2%) | 945 (37.2%) |
| 5 | 394 (16.0%) | 16 (20.0%) | 410 (16.1%) |
| 6 | 234 (9.5%) | 9 (11.2%) | 243 (9.6%) |
| **Age corrected for premature birth** |  |  |  |
| Mean | 38.478 | 38.670 | 38.484 |
| SD | 3.434 | 3.705 | 3.442 |
| Median (Q1, Q3) | 38.280 (35.710, 40.710) | 38.070 (35.532, 41.175) | 38.280 (35.710, 40.710) |
| Range | 29.570 - 53.140 | 32.850 - 49.710 | 29.570 - 53.140 |
| **Gender** |  |  |  |
| F | 1088 (44.2%) | 36 (45.0%) | 1124 (44.3%) |
| M | 1372 (55.8%) | 44 (55.0%) | 1416 (55.7%) |
| **DPOAE 65 at 2kHz** |  |  |  |
| Mean | -9.104 | -4.856 | -8.970 |
| SD | 7.717 | 8.577 | 7.780 |
| Median (Q1, Q3) | -8.000 (-14.200, -3.975) | -4.850 (-9.300, -2.850) | -7.900 (-14.100, -3.900) |
| Range | -36.200 - 34.200 | -24.300 - 19.500 | -36.200 - 34.200 |

# 4 MCAR and 30% percent of missing values

```
pm = 0.3
mech = "MCAR"
```

The original dataset (data\_full) with restriction to only left ears
is used as “true comparison” for the results based on the amputed
dataset (data). The latter dataset was generated using the function
ampute of the mice package (van Buuren 2011) and generates missing
values in the variable y1 (index test). A percentage of missing values
of 0.3 and a missingness mechanism of MCAR is defined.

## 4.1 Generating the missing data and calculate AUC and ROC

```
# load data
res_auc_mcar <- readRDS("./Case study/Ergebnisse/res_auc_MCAR.rds")
roc_mcar <- readRDS("./Case study/Ergebnisse/res_roc_MCAR.rds")
```

## 4.2 Results

### 4.2.1 Results for the AUC of y1 (DPOAE)

Table 3. The AUC and its CI estimated by each method

```
tab1 <- res_auc_mcar %>%
  mutate(
    across(c(AUC, CI_lower, CI_upper), ~round(.x, 2)),
    ci = paste0("[", CI_lower, ", ", CI_upper, "]")
  ) %>%
  select(-c(CI_lower, CI_upper)) %>%
  gt() %>%
  cols_label(
    Method = "Method",
    AUC = "AUC",
    ci = "Confidence interval"
  )
tab1
```

| Method | AUC | Confidence interval |
| --- | --- | --- |
| CCA full data | 0.64 | [0.57, 0.7] |
| CCA missing data | 0.64 | [0.57, 0.71] |
| HDEL | 0.64 | [0.56, 0.72] |
| MI2 | 0.65 | [0.59, 0.72] |
| MIB2 | 0.64 | [0.53, 0.75] |
| mice | 0.64 | [0.57, 0.71] |
| mix | 0.63 | [0.56, 0.71] |
| mi | 0.63 | [0.56, 0.71] |
| KER | 0.64 | [0.58, 0.71] |
| AIPW | 0.64 | [0.56, 0.71] |
| CONV | 0.64 | [0.57, 0.71] |

Figure 1. The AUC and its confidence interval estimated by each
method under MCAR

```
# get values for AUC of full dataset
AUC <- res_auc_mcar[which(res_auc_mcar$Method=="CCA full data"), "AUC"]
CIL <- res_auc_mcar[which(res_auc_mcar$Method=="CCA full data"), "CI_lower"]
CIU <- res_auc_mcar[which(res_auc_mcar$Method=="CCA full data"), "CI_upper"]

# plot comparing the AUC estimates
plot1 <- ggplot(res_auc_mcar, aes(x=Method, y=AUC)) +
  geom_point(size=2) +
  geom_errorbar(aes(ymin=CI_lower, ymax=CI_upper), linewidth=1, width=0.5)  +
  geom_hline(yintercept = AUC, col = "red") +
  geom_hline(yintercept = c( CIL, CIU), col = "red", linetype = "dashed") +
  theme(axis.title = element_text(size = 18),
        axis.text = element_text(size = 16),
        plot.title = element_text(size = 20),
        axis.text.x = element_text(angle=45, vjust=1, hjust=1))
plot1
```

```
#ggsave("./Case study/Ergebnisse/mcar_AUC.pdf", width = 20, height = 10)
```

The solid red line indicates the AUC estimate calculated by standard
complete case analysis based on the full dataset. The dashed red lines
show its confidence limits.

### 4.2.2 Results of the ROC

Figure 2. The ROC estimated by each method under MCAR

```
# plot comparing the ROC curves (without CI),
cols <- c("CONV"="blue","HDEL"="green", "CCA_full"="red", "CCA"="black", "KER"="grey", "MI2"="orange", "MIB2"="purple",
          "mice"="pink", "mix"="yellow", "mi"="brown")

ggplot(data=roc_mcar, aes(x=tt))+
  geom_line(aes(y=ROC.CONV,color="CONV"), linewidth=1)+
  geom_line(aes(y=ROC.CCA_full,color="CCA_full"), linewidth=1)+
  geom_line(aes(y=ROC.CCA,color="CCA"), linewidth=1)+
  geom_line(aes(y=ROC.HDEL,color="HDEL"), linewidth=1)+
  geom_line(aes(y=ROC.KER,color="KER"), linewidth=1)+
  geom_line(aes(y=ROC.MIB2,color="MIB2"), linewidth=1)+
  geom_line(aes(y=ROC.MI2,color="MI2"), linewidth=1)+
  geom_line(aes(y=ROC.mice,color="mice"), linewidth=1)+
  geom_line(aes(y=ROC.mix,color="mix"), linewidth=1)+
  geom_line(aes(y=ROC.mi_,color="mi"), linewidth=1)+
  scale_colour_manual(name="Methods",values=cols) +
  ylab("True Positive Rate") + xlab("False Positive Rate") +
  geom_line(aes(y=tt,color="black")) +
  theme(axis.title = element_text(size = 18),
        axis.text = element_text(size = 16),
        plot.title = element_text(size = 20),
        legend.text = element_text(size = 16),
        legend.title = element_text(size = 18))
```

```
#ggsave("./Case study/Ergebnisse/mcar_ROC.pdf", width = 15, height = 10)
```

The method AIPW is not included in this figure of ROCs as it does not
calculate the ROC.

# 5 MAR and 30% percent of missing values

```
pm = 0.3
mech = "MAR"
```

The original dataset (data\_full) with restriction to only left ears
is used as “true comparison” for the results based on the amputed
dataset (data). The latter dataset was generated using the function
ampute of the mice package (van Buuren 2011) and generates missing
values in the variable y1 (index test). A percentage of missing values
of 0.3 and a missingness mechanism of MAR is defined.

## 5.1 Generating the missing data and calculate AUC and ROC

```
# load data
res_auc_mar <- readRDS("./Case study/Ergebnisse/res_auc_MAR.rds")
roc_mar <- readRDS("./Case study/Ergebnisse/res_roc_MAR.rds")
```

## 5.2 Results

### 5.2.1 Results for the AUC of y1

Table 4. The AUC and its CI estimated by each method under MAR

```
tab1 <- res_auc_mar %>%
  mutate(
    across(c(AUC, CI_lower, CI_upper), ~round(.x, 2)),
    ci = paste0("[", CI_lower, ", ", CI_upper, "]")
  ) %>%
  select(-c(CI_lower, CI_upper)) %>%
  gt() %>%
  cols_label(
    Method = "Method",
    AUC = "AUC",
    ci = "Confidence interval"
  )
tab1
```

| Method | AUC | Confidence interval |
| --- | --- | --- |
| CCA full data | 0.64 | [0.57, 0.7] |
| CCA missing data | 0.62 | [0.54, 0.69] |
| HDEL | 0.62 | [0.54, 0.94] |
| MI2 | 0.64 | [0.56, 0.71] |
| MIB2 | 0.60 | [0.49, 0.7] |
| mice | 0.61 | [0.54, 0.69] |
| mix | 0.64 | [0.56, 0.72] |
| mi | 0.61 | [0.53, 0.68] |
| KER | 0.61 | [0.55, 0.68] |
| AIPW | 0.61 | [0.52, 0.69] |
| CONV | 0.62 | [0.55, 0.69] |

Figure 3. The AUC and its CI estimated by each method under MAR (Fig.
6 in the manuscript)

```
#### this chunk produces Figure 6 in the manuscript ####

AUC <- res_auc_mar[which(res_auc_mar$Method=="CCA full data"), "AUC"]
CIL <- res_auc_mar[which(res_auc_mar$Method=="CCA full data"), "CI_lower"]
CIU <- res_auc_mar[which(res_auc_mar$Method=="CCA full data"), "CI_upper"]

plot1 <- ggplot(res_auc_mar, aes(x=Method, y=AUC)) +
  geom_point(size=2) +
  geom_errorbar(aes(ymin=CI_lower, ymax=CI_upper), linewidth=1, width=0.5)  +
  geom_hline(yintercept = AUC, col = "red") +
  geom_hline(yintercept = c( CIL, CIU), col = "red", linetype = "dashed") +
  theme(axis.title = element_text(size = 18),
        axis.text = element_text(size = 16),
        axis.text.x = element_text(angle=45, vjust=1, hjust=1))
plot1
```

```
#ggsave("Ergebnisse/mar_AUC.pdf", width = 20, height = 10)
ggsave("./Case study/Ergebnisse/mar_auc.png", width = 15, height = 10)
```

The solid red line indicates the AUC estimate calculated by standard
complete case analysis based on the full dataset. The dashed red lines
show its confidence limits.

### 5.2.2 Results of the ROC

Figure 4. The ROC estimated by each method under MAR (Fig. 7 in the
manuscript)

```
#### this chunk produces Figure 7 in the manuscript ####

cols <- c("CONV"="blue","HDEL"="green", "CCA_full"="red", "CCA"="black", "KER"="grey", "MI2"="orange", "MIB2"="purple",
          "mice"="pink", "mix"="yellow", "mi"="brown")

ggplot(data=roc_mar, aes(x=tt))+
  geom_line(aes(y=ROC.CONV,color="CONV"), linewidth=1)+
  geom_line(aes(y=ROC.CCA_full,color="CCA_full"), linewidth=1)+
  geom_line(aes(y=ROC.CCA,color="CCA"), linewidth=1)+
  geom_line(aes(y=ROC.HDEL,color="HDEL"), linewidth=1)+
  geom_line(aes(y=ROC.KER,color="KER"), linewidth=1)+
  geom_line(aes(y=ROC.MIB2,color="MIB2"), linewidth=1)+
  geom_line(aes(y=ROC.MI2,color="MI2"), linewidth=1)+
  geom_line(aes(y=ROC.mice,color="mice"), linewidth=1)+
  geom_line(aes(y=ROC.mix,color="mix"), linewidth=1)+
  geom_line(aes(y=ROC.mi_,color="mi"), linewidth=1)+
  scale_colour_manual(name="Estimations",values=cols) +
  ylab("True Positive Rate") + xlab("False Positive Rate") +
  geom_line(aes(y=tt,color="black")) +
  theme(axis.title = element_text(size = 18),
        axis.text = element_text(size = 16),
        plot.title = element_text(size = 20),
        legend.text = element_text(size = 16),
        legend.title = element_text(size = 18))
```

```
#ggsave("Ergebnisse/mar_ROC.pdf", width = 15, height = 10)
ggsave("./Case study/Ergebnisse/mar_roc.png", width = 15, height = 10)
```

# 6 MNAR and 30% percent of missing values

```
pm = 0.3
mech = "MNAR"
```

The original dataset (data\_full) with restriction to only left ears
is used as “true comparison” for the results based on the amputed
dataset (data). The latter dataset was generated using the function
ampute of the mice package (van Buuren 2011) and generates missing
values in the variable y1 (index test). A percentage of missing values
of 0.3 and a missingness mechanism of MNAR is defined.

## 6.1 Generating the missing data and calculate AUC and ROC

```
# load data
res_auc_mnar <- readRDS("./Case study/Ergebnisse/res_auc_MNAR.rds")
roc_mnar <- readRDS("./Case study/Ergebnisse/res_roc_MNAR.rds")
```

## 6.2 Results

### 6.2.1 Results for the AUC of y1

Table 5. The AUC and its CI estimated by each method

```
tab1 <- res_auc_mnar %>%
  mutate(
    across(c(AUC, CI_lower, CI_upper), ~round(.x, 2)),
    ci = paste0("[", CI_lower, ", ", CI_upper, "]")
  ) %>%
  select(-c(CI_lower, CI_upper)) %>%
  gt() %>%
  cols_label(
    Method = "Method",
    AUC = "AUC",
    ci = "Confidence interval"
  )
tab1
```

| Method | AUC | Confidence interval |
| --- | --- | --- |
| CCA full data | 0.64 | [0.57, 0.7] |
| CCA missing data | 0.60 | [0.52, 0.68] |
| HDEL | 0.60 | [0.5, 0.95] |
| MI2 | 0.58 | [0.49, 0.66] |
| MIB2 | 0.57 | [0.44, 0.7] |
| mice | 0.61 | [0.53, 0.69] |
| mix | 0.60 | [0.52, 0.68] |
| mi | 0.61 | [0.53, 0.69] |
| KER | 0.59 | [0.52, 0.65] |
| AIPW | 0.58 | [0.49, 0.67] |
| CONV | 0.58 | [0.51, 0.65] |

Figure 5. The AUC and its confidence interval estimated by each
method under MNAR

```
AUC <- res_auc_mnar[which(res_auc_mnar$Method=="CCA full data"), "AUC"]
CIL <- res_auc_mnar[which(res_auc_mnar$Method=="CCA full data"), "CI_lower"]
CIU <- res_auc_mnar[which(res_auc_mnar$Method=="CCA full data"), "CI_upper"]

plot1 <- ggplot(res_auc_mnar, aes(x=Method, y=AUC)) +
  geom_point(size=2) +
  geom_errorbar(aes(ymin=CI_lower, ymax=CI_upper), linewidth=1, width=0.5)  +
  geom_hline(yintercept = AUC, col = "red") +
  geom_hline(yintercept = c( CIL, CIU), col = "red", linetype = "dashed") +
  theme(axis.title = element_text(size = 18),
        axis.text = element_text(size = 16),
        plot.title = element_text(size = 20),
        axis.text.x = element_text(angle=45, vjust=1, hjust=1))
plot1
```

```
#ggsave("./Case study/Ergebnisse/mnar_AUC.pdf", width = 20, height = 10)
```

The solid red line indicates the AUC estimate calculated by standard
complete case analysis based on the full dataset. The dashed red lines
show its confidence limits.

### 6.2.2 Results of the ROC

Figure 6. The ROC estimated by each method under MNAR

```
cols <- c("CONV"="blue","HDEL"="green", "CCA_full"="red", "CCA"="black", "KER"="grey", "MI2"="orange", "MIB2"="purple",
          "mice"="pink", "mix"="yellow", "mi"="brown")

ggplot(data=roc_mnar, aes(x=tt))+
  geom_line(aes(y=ROC.CONV,color="CONV"), linewidth=1)+
  geom_line(aes(y=ROC.CCA_full,color="CCA_full"), linewidth=1)+
  geom_line(aes(y=ROC.CCA,color="CCA"), linewidth=1)+
  geom_line(aes(y=ROC.HDEL,color="HDEL"), linewidth=1)+
  geom_line(aes(y=ROC.KER,color="KER"), linewidth=1)+
  geom_line(aes(y=ROC.MIB2,color="MIB2"), linewidth=1)+
  geom_line(aes(y=ROC.MI2,color="MI2"), linewidth=1)+
  geom_line(aes(y=ROC.mice,color="mice"), linewidth=1)+
  geom_line(aes(y=ROC.mix,color="mix"), linewidth=1)+
  geom_line(aes(y=ROC.mi_,color="mi"), linewidth=1)+
  scale_colour_manual(name="Estimations",values=cols) +
  ylab("True Positive Rate") + xlab("False Positive Rate") +
  geom_line(aes(y=tt,color="black")) +
  theme(axis.title = element_text(size = 18),
        axis.text = element_text(size = 16),
        plot.title = element_text(size = 20),
        legend.text = element_text(size = 16),
        legend.title = element_text(size = 18))
```

```
#ggsave("./Case study/Ergebnisse/mnar_ROC.pdf", width = 15, height = 10)
```

# 7 References

Bianco AM, Boente G, González–Manteiga W, Pérez–González A.
Estimators for ROC curves with missing biomarkers values and informative
covariates. Statistical Methods & Applications. 2023.

Diagnostic and Biomarkers Statistical (DABS) Center. Fred Hutch
Cancer Center. [https://research.fredhutch.org/diagnostic-biomarkers-center/en/datasets.html.
Accessed 21 Dec 2023.

Long Q, Zhang X, Hsu C-H. Nonparametric multiple imputation for
receiver operating characteristics analysis when some biomarker values
are missing at random. Stat Med. 2011a;30(26):3149-61.

Long Q, Zhang X, Johnson BA. Robust estimation of area under ROC
curve using auxiliary variables in the presence of missing biomarker
values. Biometrics. 2011b;67(2):559-67.

Norton SJ, Gorga MP, Widen JE, Folsom RC, Sininger Y, Cone-Wesson B,
et al. Identification of neonatal hearing impairment: evaluation of
transient evoked otoacoustic emission, distortion product otoacoustic
emission, and auditory brain stem response test performance. Ear Hear.
2000;21(5):508-28.

Schafer J. mix: Estimation/Multiple Imputation for Mixed Categorical
and Continuous Data. R package version 10-11. 2022.

Su Y-S, Gelman A, Hill J, Yajima M. Multiple Imputation with
Diagnostics (mi) in R: Opening Windows into the Black Box. Journal of
Statistical Software. 2011;45(2):1 - 31.

van Buuren S, Groothuis-Oudshoorn K. mice: Multivariate Imputation by
Chained Equations in R. Journal of Statistical Software. 2011;45(3):1 -
67.

Wang B, Qin G. Imputation-based empirical likelihood inference for
the area under the ROC curve with missing data. Stat Interface.
2012;5(3):319-29.

Wang B, Qin G. Empirical likelihood-based confidence intervals for
the sensitivity of a continuous-scale diagnostic test with missing data.
Commun Stat Theory Methods. 2014;43(15):3248-68.

# 8 Session Info

```
sessionInfo()
```

```
## R version 4.4.1 (2024-06-14 ucrt)
## Platform: x86_64-w64-mingw32/x64
## Running under: Windows 10 x64 (build 19045)
## 
## Matrix products: default
## 
## 
## locale:
## [1] LC_COLLATE=German_Germany.utf8  LC_CTYPE=German_Germany.utf8   
## [3] LC_MONETARY=German_Germany.utf8 LC_NUMERIC=C                   
## [5] LC_TIME=German_Germany.utf8    
## 
## time zone: Europe/Berlin
## tzcode source: internal
## 
## attached base packages:
## [1] stats4    stats     graphics  grDevices utils     datasets  methods  
## [8] base     
## 
## other attached packages:
##  [1] ks_1.14.3     arsenal_3.6.3 gt_0.11.0     dplyr_1.1.4   haven_2.5.4  
##  [6] ggpubr_0.6.0  writexl_1.5.0 ggplot2_3.5.1 mi_1.1        Matrix_1.7-0 
## [11] mix_1.0-12    mitml_0.4-5   pROC_1.18.5   mice_3.16.0  
## 
## loaded via a namespace (and not attached):
##  [1] tidyselect_1.2.1   farver_2.1.2       fastmap_1.2.0      pracma_2.4.4      
##  [5] digest_0.6.37      rpart_4.1.23       lifecycle_1.0.4    survival_3.6-4    
##  [9] magrittr_2.0.3     compiler_4.4.1     rlang_1.1.4        sass_0.4.9        
## [13] tools_4.4.1        utf8_1.2.4         yaml_2.3.10        knitr_1.48        
## [17] ggsignif_0.6.4     labeling_0.4.3     mclust_6.1.1       plyr_1.8.9        
## [21] xml2_1.3.6         abind_1.4-8        KernSmooth_2.23-24 withr_3.0.2       
## [25] purrr_1.0.2        nnet_7.3-19        grid_4.4.1         fansi_1.0.6       
## [29] jomo_2.7-6         colorspace_2.1-1   scales_1.3.0       iterators_1.0.14  
## [33] MASS_7.3-60.2      cli_3.6.3          mvtnorm_1.3-1      rmarkdown_2.28    
## [37] ragg_1.3.3         generics_0.1.3     rstudioapi_0.16.0  tzdb_0.4.0        
## [41] minqa_1.2.8        cachem_1.1.0       splines_4.4.1      vctrs_0.6.5       
## [45] boot_1.3-30        glmnet_4.1-8       jsonlite_1.8.8     carData_3.0-5     
## [49] car_3.1-2          hms_1.1.3          rstatix_0.7.2      systemfonts_1.1.0 
## [53] foreach_1.5.2      tidyr_1.3.1        jquerylib_0.1.4    glue_1.7.0        
## [57] nloptr_2.1.1       pan_1.9            codetools_0.2-20   shape_1.4.6.1     
## [61] gtable_0.3.5       lme4_1.1-35.5      munsell_0.5.1      tibble_3.2.1      
## [65] pillar_1.9.0       htmltools_0.5.8.1  R6_2.5.1           textshaping_0.4.0 
## [69] rprojroot_2.0.4    evaluate_1.0.3     lattice_0.22-6     highr_0.11        
## [73] readr_2.1.5        backports_1.5.0    broom_1.0.6        arm_1.14-4        
## [77] bslib_0.8.0        Rcpp_1.0.13        coda_0.19-4.1      nlme_3.1-164      
## [81] xfun_0.47          forcats_1.0.0      pkgconfig_2.0.3
```
