# Supplementary material for: Comparison of methods to handle missing values in a continuous index test in a diagnostic accuracy study – a simulation study
Source: BMC Med Res Methodol. 2025 May 27;25:147. doi: 10.1186/s12874-025-02594-2 (PMC12107930; doi:10.1186/s12874-025-02594-2)
Supplement: Supplementary file 4 — Additional file 4. Comparison of methods to handle missing values in a continuous index test in a diagnostic study – a simulation study (Additional file 4). Detailed results of the main simulation [file 12874_2025_2594_MOESM4_ESM.html]

Comparison of methods to handle missing values in a continuous index test in a diagnostic study – a simulation study (Additional file 4)


Code 

- Show All Code
- Hide All Code

# Comparison of methods to handle missing values in a continuous index test in a diagnostic study – a simulation study (Additional file 4)

#### Katharina Stahlmann, University Medical Center Hamburg-Eppendorf Institute of Medical Biometry and Epidemiology, k.stahlmann@uke.de

#### Bastiaan Kellerhuis, Julius Center for Health Sciences and Primary Care, University Medical Center Utrecht, Utrecht University, Utrecht, The Netherlands; Blavatnik School of Government, University of Oxford, Oxford, UK

#### Johannes B. Reitsma, Julius Center for Health Sciences and Primary Care, University Medical Center Utrecht, Utrecht University, Utrecht, The Netherlands

#### Nandini Dendukuri, Department of Medicine, McGill University, Montreal, Canada

#### Antonia Zapf, University Medical Center Hamburg-Eppendorf Institute of Medical Biometry and Epidemiology

#### 2025-04-25

# 1 Settings

## 1.1 Packages

```
library(ggplot2)
library(writexl)
library(ggpubr)
library(RColorBrewer)
library(dplyr)
library(arsenal)
library(rsimsum)
library(gt)
library(gtsummary)
library(lme4)
library(lmerTest)
```

```
# set global controls for tableby command
mycontrols = tableby.control(numeric.stats=c("Nmiss","mean", "sd", "medianq1q3", "range"),
                             cat.stats=c("Nmiss", "countpct"), 
                             stats.labels=list(Nmiss='Missing values', medianq1q3='Median (Q1, Q3)'),
                             test = F)


#display.brewer.all(n=8, type="qual", select=NULL, exact.n=TRUE, colorblindFriendly=TRUE)
colors <- brewer.pal(8, "Dark2")
```

```
tabno <- 0
figno <- 0
```

# 2 Simulation

## 2.1 Set up simulation parameters

```
nsim <- 1000

grid = expand.grid(
  sim = 1:nsim 
  , N = c(100, 500, 1000)
  , p = c(0.1, 0.3, 0.5)
  , AUC_0 = c(0.7, 0.85, 0.9)
  , r = c(0.2, 0.5, 0.9)
  , pm = c(0.1, 0.3, 0.5)
  , mech = c("MCAR", "MAR", "MNAR")
)

# methods to be compared
methods <- c("CCA", "MI2", "MIB2", "HDEL", "KER", "mice", "mix", "AIPW")
tabno <- tabno+1
title <- paste0("Table ", tabno, ". Overview of simulation parameters")
  
  
Abbreviation = names(grid)
setup <- data.frame(
  Abbreviation = names(grid),
  Parameter = c("Number of simulations", "Sample size", "prevalence of the target condition", "True AUC", "Correlation between index test and covariates", "proportion of missing values", "Missingness Mechanism"),
  Values = c(
    nsim,
    sapply(Abbreviation[2:length(Abbreviation)], function(x) (paste(unique(grid[,x]), collapse = ',')))
  )
)  
setup %>%
  gt() %>% 
  tab_style(style = cell_text(weight = "bold"), locations = cells_column_labels(columns=c("Abbreviation", "Parameter", "Values"))) %>%
  tab_header(
    title = title
  )
```

|  |  |  |
| --- | --- | --- |
| Table 1. Overview of simulation parameters | | |
| Abbreviation | Parameter | Values |
| sim | Number of simulations | 1000 |
| N | Sample size | 100,500,1000 |
| p | prevalence of the target condition | 0.1,0.3,0.5 |
| AUC\_0 | True AUC | 0.7,0.85,0.9 |
| r | Correlation between index test and covariates | 0.2,0.5,0.9 |
| pm | proportion of missing values | 0.1,0.3,0.5 |
| mech | Missingness Mechanism | MCAR,MAR,MNAR |

## 2.2 Description of methods

Table 2. Overview of methods

| Methods |  |
| --- | --- |
| CCA | Complete case analysis |
| HDEL | Hot Deck Empirical Likelihood Approach (Wang and Qin 2012, 2014) |
| MI2 | Multiple Imputation using prediction and propensity score (Long et al. 2011a) |
| MIB2 | MI2 + bootstrap step for calculating confidence intervals (Long et al. 2011a) |
| mice | Multiple Imputation using chained equations (van Buuren et al. 2011) |
| mix | Multiple Imputation using joint modelling (Schafer 2022) |
| KER | Kernel-based Inverse Probability Weighting (Bianco et al. 2023) |
| AIPW | Augmented Inverse Probability Weighting (Long et al. 2011b) |

### 2.2.1 CCA

We compared the below explained methods with the standard complete
case analysis (CCA) which excludes all subjects with missing values in
the index test (or reference standard) from the calculations. For the
application of the CCA, we used the ci.auc function of the pROC package
(Robin et al. 2011). It estimates the AUc by the trapezoidal rule
(Fawcett 2005), with \[\widehat{AUC}\_{(t\_{i}-t\_{i-1})} = (t\_{i}-t\_{i-1})
\times \frac{f(t\_{i})+f(t\_{i-1}))}{2}\] for each value of the
false positive value \(t\_{i}\). The AUC
for the full interval \([a,b]\) is then
estimated by \[\widehat{AUC}\_{(b-a)} =
\sum\_{t=a+1}^{b} AUC\_{(t\_{i}-t\_{i-1})}\] The confidence interval
for the AUC is calculated by the same funciton according to DeLong
(1988). For the case study, the ROC is estimated by the roc() function
in the pROC package (Robin et al. 2011).

### 2.2.2 HDEL

The HDEL method was developed by Wang and Qin (2012; 2014) and
provided by them as R code upon request. First, hot deck imputation is
performed under the MCAR assumption. The AUC is then estimated based on
the imputed dataset folowing the Wilcoxon Mann-Whitney statistic (Hanley
and McNeil 1982): \[\widehat{AUC} =
\frac{1}{mn} \sum\_{i=1}^{m} \sum\_{j=1}^{n}I(Y\_{i} \ge X\_{j})\]
where \(X\_{1},...,X\_{m}\) are the index
test values of the subjects without the target condition and \(Y\_{1},...,Y\_{n}\) are the index test values
of subjects with the target condition. For the ROC, sensitivity values
for some false positive values are calculated. Subsequently, empirical
likelihood confidence intervals are estimated for the AUC and the
sensitivities using the scaled chi-square distribution. For calculating
the CI of the sensitivities, Wang and Qin (2012; 2014) proposed the
imputation-based profile empirical likelihood and the imputation-based
hybrid bootstrap empirical likelihood approach. We only used the former
approach in our simulation study, as the code for the latter was not
available. This method assumes missing values to be MCAR.

### 2.2.3 Mice

Multiple imputations by chained equations is a standard multiple
imputation approach that employs fully conditional specification via
Markov Chain Monte Carlo methods. It can be accessed through the mice
package in R (van Buuren and Groothuis-Oudshoorn 2011). For each
variable with missing data, conditional densities are specified.
Multivariate imputation is performed variable-by-variable and iterating
over the conditional densities. We selected predictive mean matching as
imputation method since this is the default method for numeric variables
in mice and performs well overall (van Buuren and Groothuis-Oudshoorn
2011). As Bayesian linear regression may be more efficient for normally
distributed data (van Buuren 2018), we include mice using this
imputation method (called norm) in addition to mice with PMM in
supplemental simulation scenarios. We set the number of iterations to 10
and generated 20 imputation datasets for our simulation as Schafer and
Graham (2002) deemed 20 imputations to remove most noise in the
estimates. All variables (index test, the three covariates and the
reference standard) without interaction terms were included in the
imputation model. For each imputed dataset, AUC and ROC values are
estimated with the auc() and roc() function, respectively, of the pROC
package (Robin et al. 2011) and pooled by Rubin’s rules (1987). The
respective AUC formula is given in the decription of CCA. The confidence
interval is estimated based on DeLong et al. (1988) using Rubin’s rules
(1987).

### 2.2.4 Mix

In contrast to the previous packages, the mix package by Schafer
(2022) employs joint modeling under an unrestricted general location
model for mixed data. After defining the multivariate distribution for
the missing data, missing values are imputed from their conditional
distribution using Markov chain Monte Carlo methods. This approach is
described in Schafer (2022). For this simulation study, we used the same
settings as Schafer (2022) and set the number of iterations to 1,000 and
the number of steps to 100. In congruence with the MI approach described
above, we used all variables without interaction terms in the imputation
model and estimated the AUC, its confidence interval and ROC values in
the same way.

### 2.2.5 MI2 and MIB2

Both methods MI2 and MIB2 follow a nonparametric multiple imputation
approach and were developed by Long et al. (2011a). They apply k-nearest
neighbor matching based on a propensity and prediction score, which can
be used if index test values are MAR. Firstly, the propensity of
missingness of the index test is modeled by means of a logistic
regression using available covariates (in this simulation study x2, x3,
x4) as predictors. Secondly, a linear regression is performed to model
the observed index test values against available covariates (in this
simulation study x2, x3, x4). Based on the model estimates, a prediction
score and a propensity score are computed for each subject and
standardized to mean=0 and standard deviation SD=1. A distance measure
between subjects with missing and observed index test values is computed
by combining both scores. Different weights can be assigned to both
scores (in sum equal to 1). In this simulation study, equal weights
(each 0.5) were used following Long et al. (2011a). Stratified by target
condition, missing values are subsequently multiply imputed with a
random draw of one of the k nearest neighbors (subjects with the
shortest distance and observed index test values). The AUC and its
confidence interval are estimated based on the imputed datasets as
described for mice. The combination of both the propensity and
prediction models results in a doubly robust model. That is to say, at
least one model must be correctly specified to be consistent (Long et
al. 2011a). The MIB2 method further includes a bootstrap step before the
estimation of both models to reflect the uncertainty in estimating the
model parameters. Both methods are only available as R code. Following
Long et al. (2011a), the number of nearest neighbors was set to 3, and
the number of imputation datasets to 10.

### 2.2.6 KER

Bianco et al. (2023) proposed a kernel-based approach according to
Pulit (2016) which was extended by inverse probability weighting.
Thereby, they constructed pseudo observations and estimated a propensity
model via a logistic regression using available covariates (here x2, x3,
x4). Based on the following model the ROC and AUC wre estimated: \[ \widehat{ROC}\_{KER}(p) = \frac{1}
{\sum\_{l=1}^{n\_{D}}
\frac{\delta\_{D,l}}{\hat{\pi}\_{D}(\mathbf{x}\_{D,l})}} \sum\_{j=1}^{n\_{D}}
\frac{\delta\_{D,j}}{\hat{\pi}\_{D}(\mathbf{x\_{D,j}})} \varkappa \left(
\frac{p-\hat{Z}\_{j}}{h} \right) \] where \(\varkappa(t) = \int\_{-\infty}^{t}K(u)du\)
and \(K\) is a continuous symetric
density function with support \([-1,1]\), \(h\) is a smoothing parameter, \(\delta\) is the missingness indicator for
the index test \(Y\) with \(\delta=1\) when \(Y\) is observed and \(\delta=0\) when \(Y\) is missing, \(\mathbf{x}\) denotes available covariates
and \(\hat{\pi}\) is an estimate of
\(Pr(\delta=1)\) conditional on
covariates and true target condition. \(\hat{Z}\_{j}\) are pseudo-observations for
each \(1\le j \le n\_{D}\) with \(\delta\_{D,j}=1\) defined by \(\hat{Z}\_{j} = 1-
\hat{F}\_{H,IPW}(y\_{D,j})\). The AUC is then estimated by \[\widehat{AUC}\_{KER} = (1/N)\sum\_{l=1}^N
\widehat{ROC}\_{KER}(p\_{l}) \] with \(\left\{ p\_{l} \right\}\_{1\le l\le N}\) an
equisdistant grid over \((0,1)\)
(Bianco et al. 2023). The confidence interval is estimated following
Hanley and McNeil (1982). Upon request, the authors provided this method
as R code.

### 2.2.7 AIPW

Long et al. (2011b) proposed a doubly robust augmented inverse
probability method that is applicable for missing index test values
under MAR. Similar to the MI2 and MIB2 methods, the propensity of
missingness of the index test is modeled through a logistic regression
using available covariates (in this simulation study x2, x3, x4) as
predictors. Then, a linear regression is performed to model the observed
index test values against available covariates (here x2, x3, x4). The
AUC is a weighted average of the linear regression and the inverse
probability (propensity score) estimator:

\[\widehat{AUC} = \frac{1}{\sum\_{i \ne j}
\frac{\delta\_{i} \delta\_{j}}{\hat{\pi}\_{i} \hat{\pi}\_{j}}
D\_{i}(1-D\_{j})} \sum\_{i\neq j} D\_{i}(1-D\_{j}) \left\{ \frac{\delta\_{i}
\delta\_{j}} {\hat{\pi}\_{i} \hat{\pi}\_{j}} I\_{ij} - \frac{\delta\_{i}
\delta\_{j} - \hat{\pi}\_{i} \hat{\pi}\_{j}}{\hat{\pi}\_{i} \hat{\pi}\_{j}}
E(I\_{i,j}| \mathbf{Z\_{i}},\mathbf{Z\_{j}},D\_{i}=1,D\_{j}=0)
\right\}\]

where \(i=1,...,n\) and \(j=1,...,n\) are the subjects with target
condition \((D\_{i}=1)\) and without
\((D\_{i}=0)\), \(\delta\) is the missingness indicator for
the index test \(X\) with \(\delta=1\) when \(X\) is observed and \(\delta=0\) when \(X\) is missing, \(\mathbf{Z}\) denotes available covariates
and \(\hat{\pi}\) is an estimate of
\(Pr(\delta=1)\) conditional on \(\mathbf{Z}\) and \(D\) (Long et al. 2011b). Its CI is computed
using bootstrap (200 bootstrap samples). Long et al. (2011b)
additionally proposed a sensitivity analysis to investigate MNAR.
Unfortunately, their original code was not available. However, the code
was included in the code of Cheng and Tang (2020) as they used the AIPW
method under MAR for comparison.

## 2.3 Conduct the simulation

As some methods take much time to run, we decided to split the
simulation study in two parts. First, the scenarios of the 1st grid
(grid1) are run and then the scenarios of the 2nd and 3rd (grid2, grid3)
are run (see simulation program for details). For this purpose, the seed
for the parallel loop was set to 5273 and to 4730xi for data generation
for the first simulation run and to 952 and 810xi for the second
simulation run. As the scenarios of the first and second part still
needed too much time, we did run some methods separately. We cannot
conduct the simulation programs within this Rmarkdown file, as they were
executed on a high performance cluster. However, we provide the R
programs and the results of the simulations to enable reproduction.

```
# Result of Part 1

data <- c("results_part1", "results_micemix", "part1_aipw1", "part1_aipw2")
list_part1 <- list()

for (i in 1:length(data)) {
  
  load(file = paste0("./Analyse/hpc/Simulation_data/", data[i], ".Rdata"))
  res$id <- paste(res$sim, res$scenario, sep = "_")
  if (data[i]=="part1_aipw2") {
    list_part1[[i-1]] <- rbind(list_part1[[i-1]], res) # part 1 of method AIPW was divided in to programs: must be combined first
    assign(paste0("part1_", i-1), list_part1[[i-1]])
  } else{
    list_part1[[i]] <- res
    assign(paste0("part1_", i), list_part1[[i]])
  }
  
}

# merge results by id
res_part1 <- merge(part1_1, part1_2[,c(10:ncol(part1_2))], by="id")
res_part1 <- merge(res_part1, part1_3[,c(10:ncol(part1_3))], by="id")

# --> reihenfolge ändert sich!!!!
res_part1 <- res_part1 %>%
  arrange(scenario, sim) %>%
  select(-id)
```

```
# Part 2
data <- c("results_part2_1", "results_part2_2", "results_part2_3", "results_micemix2_1", "results_micemix2_2", "results_micemix2_3", "results_micemix2_4", "part2_aipw1", "part2_aipw2", "part2_aipw3", "part2_aipw4", "part2_aipw5", "part2_aipw6", "part2_aipw7")
list_part2 <- list()

for (i in 1:length(data)) {
  
  load(file = paste0("./Analyse/hpc/Simulation_data/", data[i], ".Rdata"))
  res$id <- paste(res$sim, res$scenario, sep = "_")
  list_part2[[i]] <- res
  assign(paste0(data[i]), list_part2[[i]])
}

# part 2 of several methods was divided in to multiple programs: must be combined first
part2_1 <- rbind(results_part2_1, results_part2_2, results_part2_3)
part2_2 <- rbind(results_micemix2_1, results_micemix2_2, results_micemix2_3, results_micemix2_4)
part2_3 <- rbind(part2_aipw1, part2_aipw2, part2_aipw3, part2_aipw4, part2_aipw5, part2_aipw6, part2_aipw7)

# merge results by id
res_part2 <- merge(part2_1, part2_2[,c(10:ncol(part2_2))], by="id")
res_part2 <- merge(res_part2, part2_3[,c(11:ncol(part2_3))], by="id") # without column row.grid

# --> reihenfolge ändert sich!!!!
res_part2 <- res_part2 %>%
  arrange(scenario, sim) %>%
  select(-id)

## combine both parts
res <- rbind(res_part1, res_part2)

#include a new number for the scenario (otherwise there would be some scenario numbers twice)
res$scenario <- rep(1:(nrow(res)/nsim), each = nsim)
res <- rename(res, r = korr)
```

# 3 Calculate Performance parameters

The following performance parameter will be calculated: number of
missing values, bias, root mean squared error, empirical standard
deviation, coverage probability and power as well as the respective
monte carlo standard errors for each performance parameter.

```
fix_col <- c("N","p","AUC_0","r", "pm","mech", "AUC_min","scenario")
source("./Analyse/Simulation_performance3.R")
# input: res (file with simulation results as dataframe)
# out: raw_res (res + step 1 of performance calculation -> 1 row for each simulation run), scenario_res (dataframe with performance parameters, -> 1 row for each scenario aggregated over the iterations)

# save results in excel file
write_xlsx(scenario_res, path = "./Analyse/Ergebnisse/res_sim_performance.xlsx")
```

# 4 Results

## 4.1 Running time and missing values

```
# rearrange the scenario_res table columns, so that performance estimate and its MC standard error are located side by side
vars=list()
for (i in 1:length(methods)){
  vars_i <- grep(methods[i], names(scenario_res), value = T)
  vars[[i]] <- vars_i
}
vars_order <- unlist(vars)
dat_table1 <- scenario_res[,c(8,1:6)]
dat_table2 <- scenario_res[,vars_order]
dat_table <- cbind(dat_table1, dat_table2)
rownames(dat_table) <- NULL

# label selected variables in dat_table
dat_table <- dat_table %>%
  sjlabelled::var_labels(
    scenario = "Scenario",
    N = "Sample size",
    p = "Prevalence of the target condition",
    AUC_0 = "True AUC",
    r = "Correlation",
    pm = "Proportion of missing values",
    mech = "Missingness mechanism",
    av.time.AUC.CCA = "Average running time for CCA",
    av.time.AUC.MI2 = "Average running time for MI2",
    av.time.AUC.MIB2 = "Average running time for MIB2",
    av.time.AUC.HDEL = "Average running time for HDEL",
    av.time.AUC.mice = "Average running time for mice",
    av.time.AUC.mix = "Average running time for mix",
    av.time.AUC.AIPW = "Average running time for AIPW",
    av.time.AUC.KER = "Average running time for KER"
  )
```

Table 3. Overview of average running time (in seconds) summarized
across all scenarios, stratified by sample size

```
# table with running time summarized across all scenarios 
time <- grep("time", names(dat_table), value = TRUE)
summary(tableby(N ~ ., data = dat_table[,c("N",time)], control = mycontrols), pfootnote = T)
```

|  | 100 (N=243) | 500 (N=243) | 1000 (N=243) | Total (N=729) |
| --- | --- | --- | --- | --- |
| **Average running time for CCA** |  |  |  |  |
| Mean | 0.002 | 0.004 | 0.005 | 0.004 |
| SD | 0.000 | 0.000 | 0.000 | 0.001 |
| Median (Q1, Q3) | 0.002 (0.002, 0.002) | 0.004 (0.003, 0.004) | 0.005 (0.005, 0.005) | 0.004 (0.002, 0.005) |
| Range | 0.002 - 0.003 | 0.003 - 0.005 | 0.004 - 0.006 | 0.002 - 0.006 |
| **Average running time for MI2** |  |  |  |  |
| Mean | 0.051 | 0.100 | 0.174 | 0.108 |
| SD | 0.002 | 0.009 | 0.026 | 0.053 |
| Median (Q1, Q3) | 0.051 (0.049, 0.052) | 0.099 (0.091, 0.108) | 0.173 (0.144, 0.198) | 0.099 (0.052, 0.144) |
| Range | 0.046 - 0.057 | 0.085 - 0.117 | 0.138 - 0.228 | 0.046 - 0.228 |
| **Average running time for MIB2** |  |  |  |  |
| Mean | 0.185 | 0.437 | 0.892 | 0.504 |
| SD | 0.011 | 0.093 | 0.276 | 0.337 |
| Median (Q1, Q3) | 0.183 (0.176, 0.193) | 0.431 (0.331, 0.528) | 0.869 (0.579, 1.132) | 0.431 (0.193, 0.587) |
| Range | 0.158 - 0.218 | 0.314 - 0.600 | 0.528 - 1.429 | 0.158 - 1.429 |
| **Average running time for HDEL** |  |  |  |  |
| Mean | 0.019 | 0.029 | 0.047 | 0.031 |
| SD | 0.007 | 0.010 | 0.013 | 0.016 |
| Median (Q1, Q3) | 0.019 (0.011, 0.026) | 0.027 (0.018, 0.038) | 0.044 (0.035, 0.060) | 0.028 (0.018, 0.042) |
| Range | 0.008 - 0.030 | 0.015 - 0.051 | 0.029 - 0.075 | 0.008 - 0.075 |
| **Average running time for KER** |  |  |  |  |
| Mean | 0.020 | 0.147 | 0.476 | 0.214 |
| SD | 0.003 | 0.052 | 0.174 | 0.219 |
| Median (Q1, Q3) | 0.020 (0.016, 0.023) | 0.161 (0.079, 0.196) | 0.528 (0.245, 0.640) | 0.161 (0.023, 0.244) |
| Range | 0.014 - 0.026 | 0.074 - 0.218 | 0.230 - 0.699 | 0.014 - 0.699 |
| **Average running time for mice** |  |  |  |  |
| Mean | 1.483 | 1.561 | 1.761 | 1.602 |
| SD | 0.178 | 0.109 | 0.111 | 0.180 |
| Median (Q1, Q3) | 1.441 (1.335, 1.687) | 1.603 (1.475, 1.642) | 1.758 (1.674, 1.866) | 1.634 (1.454, 1.719) |
| Range | 1.192 - 1.779 | 1.224 - 1.711 | 1.454 - 2.009 | 1.192 - 2.009 |
| **Average running time for mix** |  |  |  |  |
| Mean | 0.309 | 0.592 | 0.905 | 0.602 |
| SD | 0.013 | 0.024 | 0.040 | 0.245 |
| Median (Q1, Q3) | 0.312 (0.299, 0.319) | 0.591 (0.578, 0.610) | 0.902 (0.878, 0.930) | 0.591 (0.319, 0.877) |
| Range | 0.274 - 0.332 | 0.495 - 0.648 | 0.824 - 1.009 | 0.274 - 1.009 |
| **Average running time for AIPW** |  |  |  |  |
| Mean | 2.038 | 9.569 | 31.985 | 14.531 |
| SD | 0.132 | 2.819 | 11.046 | 14.325 |
| Median (Q1, Q3) | 1.991 (1.946, 2.085) | 10.602 (5.829, 12.229) | 36.228 (17.186, 42.640) | 10.602 (2.086, 17.177) |
| Range | 1.811 - 2.380 | 5.427 - 12.967 | 15.951 - 44.307 | 1.811 - 44.307 |

Table 4. Overview of the sum of missing values for the estimated AUC
summarized over all repetitions and scenarios stratified by N, p and pm
(only combinations with missing values in at least one method are
displayed; Table 3 in the manuscript)

```
# this chunk produces Table 3 in the manuscript

miss <- grep("Missing", names(dat_table), value = TRUE)
dat_table_miss2 <- dat_table %>%
  select(c(1:8, all_of(miss))) %>%
  group_by(N, p, pm) %>%
  summarise(
    across(miss, list(sum=sum)),
    n = n()
    ) %>%
  mutate(
    rowmiss = rowSums(across(starts_with("Missing_")), na.rm=TRUE) ,
    CCA = paste0(Missing_AUC.AUC.CCA_sum,  " (", round(Missing_AUC.AUC.CCA_sum/(n*1000)*100,2), "%)"),
    MI2 = paste0(Missing_AUC.AUC.MI2_sum,  " (", round(Missing_AUC.AUC.MI2_sum/(n*1000)*100,2), "%)"),
    MIB2 = paste0(Missing_AUC.AUC.MIB2_sum,  " (", round(Missing_AUC.AUC.MIB2_sum/(n*1000)*100,2), "%)"),
    HDEL = paste0(Missing_AUC.AUC.HDEL_sum,  " (", round(Missing_AUC.AUC.HDEL_sum/(n*1000)*100,2), "%)"),
    KER = paste0(Missing_AUC.AUC.KER_sum,  " (", round(Missing_AUC.AUC.KER_sum/(n*1000)*100,2), "%)"),
    mice = paste0(Missing_AUC.AUC.mice_sum,  " (", round(Missing_AUC.AUC.mice_sum/(n*1000)*100,2), "%)"),
    mix = paste0(Missing_AUC.AUC.mix_sum, " (", round(Missing_AUC.AUC.mix_sum/(n*1000)*100,2), "%)"),
    AIPW = paste0(Missing_AUC.AUC.AIPW_sum,  " (", round(Missing_AUC.AUC.AIPW_sum/(n*1000)*100,2), "%)")
  ) %>%
  filter(rowmiss>0) %>% # only keep combinations with missing values in at least one method
  select(c(N,p,pm,CCA,MI2,MIB2,HDEL,KER,mice,mix,AIPW)) 


table <- dat_table_miss2 %>%
  gt(groupname_col = c("N"), row_group_as_column = TRUE) %>%
  tab_row_group(
    label = "Sample size = 100",
    rows = N==100
  ) %>%
  tab_row_group(
    label = "Sample size = 500",
    rows = N==500
  ) %>%
  tab_row_group(
    label = "Sample size = 1000",
    rows = N==1000
  )

table
```

|  | Prevalence of the target condition | Proportion of missing values | CCA | MI2 | MIB2 | HDEL | KER | mice | mix | AIPW |
| --- | --- | --- | --- | --- | --- | --- | --- | --- | --- | --- |
| Sample size = 1000 | 0.1 | 0.5 | 0 (0%) | 0 (0%) | 0 (0%) | 0 (0%) | 0 (0%) | 0 (0%) | 0 (0%) | 5 (0.02%) |
| Sample size = 500 | 0.1 | 0.3 | 0 (0%) | 0 (0%) | 0 (0%) | 0 (0%) | 0 (0%) | 0 (0%) | 0 (0%) | 3 (0.01%) |
|  | 0.1 | 0.5 | 0 (0%) | 0 (0%) | 0 (0%) | 0 (0%) | 0 (0%) | 0 (0%) | 0 (0%) | 322 (1.19%) |
| Sample size = 100 | 0.1 | 0.1 | 0 (0%) | 0 (0%) | 0 (0%) | 0 (0%) | 0 (0%) | 0 (0%) | 0 (0%) | 649 (2.4%) |
|  | 0.1 | 0.3 | 299 (1.11%) | 0 (0%) | 0 (0%) | 0 (0%) | 0 (0%) | 0 (0%) | 0 (0%) | 9411 (34.86%) |
|  | 0.1 | 0.5 | 2880 (10.67%) | 0 (0%) | 0 (0%) | 0 (0%) | 0 (0%) | 0 (0%) | 0 (0%) | 19680 (72.89%) |
|  | 0.3 | 0.3 | 0 (0%) | 0 (0%) | 0 (0%) | 0 (0%) | 0 (0%) | 0 (0%) | 0 (0%) | 7 (0.03%) |
|  | 0.3 | 0.5 | 3 (0.01%) | 0 (0%) | 0 (0%) | 0 (0%) | 0 (0%) | 0 (0%) | 0 (0%) | 581 (2.15%) |
|  | 0.5 | 0.5 | 0 (0%) | 0 (0%) | 0 (0%) | 0 (0%) | 0 (0%) | 0 (0%) | 0 (0%) | 18 (0.07%) |

```
tabno <- tabno+1

# show table for bias
bias <- grep("bias", names(dat_table), value = FALSE)
knitr::kable(dat_table[,c(1:7,bias)], "simple", 
             col.names = gsub("[.]", " ", names(dat_table[,c(1:7,bias)])), 
             caption = paste0("Table", tabno, ". Bias and its Monte Carlo Standard Error for each method"),
             digits = 4, format.args = list(scientific = FALSE))

# show table for RMSE 
tabno <- tabno+1
mse <- grep("MSE", names(dat_table), value = FALSE)
knitr::kable(dat_table[,c(1:7,mse)], "simple", 
             col.names = gsub("[.]", " ", names(dat_table[,c(1:7,mse)])),
             caption = paste0("Table", tabno, ". Root mean squared error and its Monte Carlo Standard Error for each method"),
             digits = 4, format.args = list(scientific = FALSE))

# show table for empSE
tabno <- tabno+1
empse <- grep("empSE", names(dat_table), value = FALSE)
knitr::kable(dat_table[,c(1:7,empse)], "simple", 
             col.names = gsub("[.]", " ", names(dat_table[,c(1:7,empse)])),
             caption = paste0("Table", tabno, ". Empirical Standard Error and its Monte Carlo Standard Error for each method"),
             digits = 4, format.args = list(scientific = FALSE))

# show table for coverage
tabno <- tabno+1
cov <- grep("cov", names(dat_table), value = FALSE)
knitr::kable(dat_table[,c(1:7,cov)], "simple", 
             col.names = gsub("[.]", " ", names(dat_table[,c(1:7,cov)])),
             caption = paste0("Table", tabno, ". Coverage and its Monte Carlo Standard Error for each method"),
             digits = 4, format.args = list(scientific = FALSE))

# show table for power
tabno <- tabno+1
power <- grep("power", names(dat_table), value = FALSE)
knitr::kable(dat_table[,c(1:7,power)], "simple", 
             col.names = gsub("[.]", " ", names(dat_table[,c(1:7,power)])),
             caption = paste0("Table", tabno, ". Power and its Monte Carlo Standard Error for each method"),
             digits = 4, format.args = list(scientific = FALSE))
```

## 4.2 Graphical display of performance results

### 4.2.1 Bias

```
# reshape from wide to long (only one column for bias and estimated AUC, respectively)
performparam <- list(auc_vars,names1)
performnames <- c("AUC","Bias")
dat_fig <- raw_res[,c("scenario","N","p","AUC_0","r","pm","mech",auc_vars,names1)]
dat_fig$id <- seq_along(1:nrow(dat_fig))
dat_long <- reshape(dat_fig, varying=performparam, v.names = performnames, times = methods, 
                    idvar = "id", direction = "long")
dat_long$scenario <- as.factor(dat_long$scenario)
colnames(dat_long)[colnames(dat_long) == "time"] <- "Method" # rename method variable
```

```
# bias plot for a specific combination of parameters
# MCAR
idx1 <- which(dat_long$mech == "MCAR" & dat_long$N==100 & dat_long$p==0.1 & dat_long$pm!=0.3 & dat_long$r!=0.5 & dat_long$AUC_0!=0.9) #  limits = c(-0.4,0.4)
idx2 <- which(dat_long$mech == "MCAR" & dat_long$N==100 & dat_long$p==0.5 & dat_long$pm!=0.3 & dat_long$r!=0.5 & dat_long$AUC_0!=0.9) #  limits = c(-0.3,0.3)

idx3 <- which(dat_long$mech == "MAR" & dat_long$N==100 & dat_long$p!=0.3  & dat_long$pm!=0.3 & dat_long$r!=0.5 & dat_long$AUC_0!=0.9)#  limits = c(-0.2,0.2)
idx4 <- which(dat_long$mech == "MAR" & dat_long$N==500 & dat_long$p!=0.3  & dat_long$pm!=0.3 & dat_long$r!=0.5 & dat_long$AUC_0!=0.9) #  limits = c(-0.15,0.15)

idx5 <- which(dat_long$mech == "MNAR" & dat_long$N==100 & dat_long$p!=0.3 & dat_long$pm!=0.3 & dat_long$r!=0.5 & dat_long$AUC_0!=0.9)
idx6 <- which(dat_long$mech == "MNAR" & dat_long$N==500 & dat_long$p!=0.3 & dat_long$pm!=0.3 & dat_long$r!=0.5 & dat_long$AUC_0!=0.9)

idxlist <- list(idx1, idx2, idx3, idx4, idx5, idx6)
limits <- list(
  c(-0.3,0.3),
  c(-0.25,0.25),
  c(-0.2,0.2),
  c(-0.12,0.12),
  c(-0.3,0.3),
  c(-0.15,0.15)
)
title <- c("Bias for MCAR, p=0.1 and N=100", "Bias for MCAR, p=0.5 and N=100",
           "Bias for MAR and N=100", "Bias for MAR and N=500",
           "Bias for MNAR and N=100", "Bias for MNAR and N=500")

plotlist <- list()
for (i in 1:length(idxlist)) {
  plotlist[[i]] <- ggplot(dat_long[idxlist[[i]],], aes(x = Method, y = Bias)) +
                          geom_violin(fill = colors[1], trim=FALSE) +
                          stat_summary(fun=mean, geom="point", size=2, color=colors[6]) +
                          xlab("Method") + ylab("Bias") +
                          geom_hline(yintercept=0) +
                          scale_y_continuous(limits = limits[[i]]) +
                          theme(axis.text.x = element_text(angle = 45, hjust = 1)) +
                          facet_grid(p + r ~ AUC_0 + pm, labeller = label_context) +
                          ggtitle(title[i]) +
                          labs(caption = paste0("The y-axis was cut at ", limits[[i]][1], " and ",limits[[i]][2], " to enable comparison across the panels.")) +
                          theme(axis.title = element_text(size = 18),
                              axis.text = element_text(size = 16),
                              plot.title = element_text(size = 20),
                              strip.text.x = element_text(size = 14),
                              strip.text.y = element_text(size = 14),
                              plot.caption = element_text(hjust = 0))
}
```

MCAR

Figure 1

```
plotlist[[1]]
```

Figure 2

```
plotlist[[2]]
```

MAR

Figure 3

```
plotlist[[3]]
```

Figure 4

```
plotlist[[4]]
```

MNAR

Figure 5

```
plotlist[[5]]
```

Figure 6

```
plotlist[[6]]
```

```
### this produces Figure 2 in the manuscript ###

idx3 <- which(dat_long$mech == "MAR" & dat_long$N==100 & dat_long$p!=0.3  & dat_long$pm!=0.3 & dat_long$r!=0.5 & dat_long$AUC_0!=0.9)#  limits = c(-0.2,0.2)
idx4 <- which(dat_long$mech == "MAR" & dat_long$N==500 & dat_long$p!=0.3  & dat_long$pm!=0.3 & dat_long$r!=0.5 & dat_long$AUC_0!=0.9) #  limits = c(-0.15,0.15)


idxlist <- list(idx3, idx4)
limits <- list(
  c(-0.2,0.2),
  c(-0.12,0.12)
)

plot_pub1 <- list()
for (i in 1:length(idxlist)) {
  plot_pub1[[i]] <- ggplot(dat_long[idxlist[[i]],], aes(x = Method, y = Bias)) +
                          geom_violin(fill = colors[1], trim=FALSE) +
                          stat_summary(fun=mean, geom="point", size=2, color=colors[6]) +
                          xlab("Method") + ylab("Bias") +
                          geom_hline(yintercept=0) +
                          scale_y_continuous(limits = limits[[i]]) +
                          theme(axis.text.x = element_text(angle = 45, hjust = 1)) +
                          facet_grid(p + r ~ AUC_0 + pm, labeller = label_context) +
                          theme(axis.title = element_text(size = 8),
                              axis.text = element_text(size = 6),
                              strip.text.x = element_text(size = 6),
                              strip.text.y = element_text(size = 6))
}

fig1 <- ggarrange(plot_pub1[[1]], plot_pub1[[2]], labels=c("A","B"), ncol=2)
fig1
ggsave("./Analyse/Ergebnisse/Plots/Bias_MAR_pub.png", plot=fig1, width = 20, height = 10)
```

```
table_b <- dat_long %>%
  filter(N==500 & r==0.5 & AUC_0==0.85 & pm==0.3 & mech=="MNAR" & (p==0.1 | p==0.5)) %>%
  select(Method, Bias,p) %>%
  tbl_strata(strata=p, .tbl_fun =  ~ .x %>% 
             tbl_summary(by=Method,
                           type = list(all_continuous()~"continuous2"),
                           statistic = list(all_continuous() ~ c("{mean} ({sd})",
                                                    "{median} ({p25}, {p75})", 
                                                    "[{min}, {max}]")),
                           digits = list(all_continuous() ~4)))
tabno <- tabno+1
tab_b <- table_b %>%
  as_gt() %>%
  tab_header(
    title = paste0("Table ", tabno, ". Summary statistics for bias for N=500, r=0.5, AUC_0=0.85, pm=0.3 and mech=MNAR")
  )
tab_b
```

|  |  |  |  |  |  |  |  |  |  |  |  |  |  |  |  |  |
| --- | --- | --- | --- | --- | --- | --- | --- | --- | --- | --- | --- | --- | --- | --- | --- | --- |
| Table 5. Summary statistics for bias for N=500, r=0.5, AUC\_0=0.85, pm=0.3 and mech=MNAR | | | | | | | | | | | | | | | | |
| **Characteristic** | **0.1** | | | | | | | | **0.5** | | | | | | | |
|  | **AIPW** | **CCA** | **HDEL**  N = 1,000 | **KER**  N = 1,000 | **MI2**  N = 1,000 | **MIB2**  N = 1,000 | **mice**  N = 1,000 | **mix**  N = 1,000 | **AIPW**  N = 1,000 | **CCA**  N = 1,000 | **HDEL**  N = 1,000 | **KER**  N = 1,000 | **MI2**  N = 1,000 | **MIB2**  N = 1,000 | **mice**  N = 1,000 | **mix**  N = 1,000 |
| Bias |  |  |  |  |  |  |  |  |  |  |  |  |  |  |  |  |
| Mean (SD) | -0.0054 (0.0376) | -0.0153 (0.0422) | -0.0144 (0.0469) | -0.0031 (0.0381) | -0.0057 (0.0396) | -0.0062 (0.0411) | -0.0117 (0.0376) | -0.0110 (0.0352) | -0.0057 (0.0194) | -0.0131 (0.0209) | -0.0128 (0.0233) | -0.0045 (0.0195) | -0.0053 (0.0202) | -0.0057 (0.0206) | -0.0065 (0.0196) | -0.0069 (0.0193) |
| Median (Q1, Q3) | -0.0032 (-0.0292, 0.0208) | -0.0138 (-0.0421, 0.0145) | -0.0110 (-0.0435, 0.0187) | -0.0012 (-0.0276, 0.0240) | -0.0035 (-0.0295, 0.0207) | -0.0041 (-0.0316, 0.0225) | -0.0094 (-0.0368, 0.0144) | -0.0081 (-0.0331, 0.0132) | -0.0048 (-0.0192, 0.0077) | -0.0129 (-0.0271, 0.0018) | -0.0125 (-0.0283, 0.0032) | -0.0038 (-0.0179, 0.0090) | -0.0044 (-0.0196, 0.0088) | -0.0051 (-0.0201, 0.0083) | -0.0065 (-0.0198, 0.0074) | -0.0065 (-0.0203, 0.0064) |
| [Min, Max] | [-0.1345, 0.0931] | [-0.1573, 0.0961] | [-0.1898, 0.1102] | [-0.1417, 0.0927] | [-0.1520, 0.1017] | [-0.1546, 0.1054] | [-0.1476, 0.0983] | [-0.1460, 0.0845] | [-0.0694, 0.0475] | [-0.0838, 0.0399] | [-0.1087, 0.0559] | [-0.0699, 0.0486] | [-0.0727, 0.0469] | [-0.0743, 0.0480] | [-0.0723, 0.0480] | [-0.0734, 0.0423] |

```
# reshape summary results for further plots 

performparam <- list(names1,names2,names3,names4,names13,names5,names6,names7,names8,names9,names14,names10,names11)
performnames <- c("Bias","RMSE","empirical_SE","Coverage","be_coverage", "Power","MCE_bias","MCE_MSE","MCE_empSE","MCE_cov","MCE_be_cov", "MCE_power", "av_time")
scenario_res$id <- seq_along(1:nrow(scenario_res))
res_long <- reshape(scenario_res, varying=performparam, v.names = performnames, times = methods, 
                    idvar = "id", direction = "long")
colnames(res_long)[colnames(res_long) == "time"] <- "Method" # rename method variable

# label selected variables in res_long
res_long <- res_long %>%
  sjlabelled::var_labels(
    MCE_bias = "Monte Carlo Standard Error for Bias",
    MCE_MSE = "Monte Carlo Standard Error for Mean Squared Error", 
    MCE_cov = "Monte Carlo Standard Error for Coverage Probability", 
    MCE_be_cov = "Monte Carlo Standard Error for bias-eliminated Coverage Probability", 
    MCE_power = "Monte Carlo Standard Error for Power"
  )

# save specific MNAR scenarios to compare them with the MNAR supplemental scenarios
mnar_test <- res_long[which(res_long$pm==0.3 & res_long$mech=="MNAR" & res_long$N!=100 & res_long$AUC_0==0.85 & res_long$r==0.5),]
save(mnar_test, file="./Analyse/mnar_test.RData")
```

```
## plots for summary performance measures ##
idx1 <- which(res_long$mech == "MCAR" & res_long$p!=0.5 & res_long$AUC_0!=0.9 & res_long$r!=0.5 & res_long$N==100)
idx2 <- which(res_long$mech == "MCAR" & res_long$p!=0.5 & res_long$AUC_0!=0.9 & res_long$r!=0.5 & res_long$N==500)

idx3 <- which(res_long$mech == "MAR" & res_long$p!=0.5 & res_long$AUC_0!=0.9 & res_long$r!=0.5 & res_long$N==100)
idx4 <- which(res_long$mech == "MAR" & res_long$p!=0.5 & res_long$AUC_0!=0.9 & res_long$r!=0.5 & res_long$N==500 )

idx5 <- which(res_long$mech == "MNAR" & res_long$p!=0.5 & res_long$AUC_0!=0.9 & res_long$N==100 & res_long$r!=0.5)
idx6 <- which(res_long$mech == "MNAR" & res_long$p!=0.5 & res_long$AUC_0!=0.9 & res_long$N==500 & res_long$r!=0.5)

idxlist <- list(idx1, idx2, idx3, idx4, idx5, idx6)
```

```
title <- c("Absolute bias for MCAR and N=100", "Absolute bias for MCAR and N=500",
           "Absolute bias for MAR and N=100", "Absolute bias for MAR and N=500", 
           "Absolute bias for MNAR and N=100", "Absolute bias for MNAR and N=500")

# line plot for bias
plotlist2.2 <- list()
for (i in 1:length(idxlist)) {
  plotlist2.2[[i]] <- ggplot(res_long[idxlist[[i]],], aes(x = pm, y = abs(Bias), group = Method)) +
                      geom_line(aes(color=Method, linetype = Method), linewidth=1) +
                      scale_color_manual(values = colors) +
                      scale_x_continuous(breaks = c(0.1,0.3,0.5)) +
                      xlab("Proportion of missing values") + ylab("Absolute bias") +
                      facet_grid(r + N  ~ AUC_0 + p, labeller = label_both) + 
                      ggtitle(title[i]) +
                          theme(axis.title = element_text(size = 18),
                              axis.text = element_text(size = 16),
                              plot.title = element_text(size = 20),
                              strip.text.x = element_text(size = 14),
                              strip.text.y = element_text(size = 14))
}
```

MCAR

Figure 7

```
plotlist2.2[[1]]
```

Figure 8

```
plotlist2.2[[2]]
```

MAR

Figure 9

```
plotlist2.2[[3]]
```

Figure 10

```
plotlist2.2[[4]]
```

MNAR

Figure 11

```
plotlist2.2[[5]]
```

Figure 12

```
plotlist2.2[[6]]
```

### 4.2.2 Root mean squared error (RMSE)

```
title <- c("RMSE for MCAR and N=100", "RMSE for MCAR and N=500",
           "RMSE for MAR and N=100", "RMSE for MAR and N=500", 
           "RMSE for MNAR and N=100", "RMSE for MNAR and N=500")

# line plot for RMSE
plotlist2 <- list()
for (i in 1:length(idxlist)) {
  plotlist2[[i]] <- ggplot(res_long[idxlist[[i]],], aes(x = pm, y = RMSE, group = Method)) +
                      geom_line(aes(color=Method, linetype = Method), linewidth=1) +
                      scale_color_manual(values = colors) +
                      scale_x_continuous(breaks = c(0.1,0.3,0.5)) +
                      xlab("Proportion of missing values") + ylab("Root mean squared error") +
                      facet_grid(r + N  ~ AUC_0 + p, labeller = label_both) + 
                      ggtitle(title[i]) +
                          theme(axis.title = element_text(size = 18),
                              axis.text = element_text(size = 16),
                              plot.title = element_text(size = 20),
                              strip.text.x = element_text(size = 14),
                              strip.text.y = element_text(size = 14))
}
```

MCAR

Figure 13

```
plotlist2[[1]]
```

Figure 14

```
plotlist2[[2]]
```

MAR

Figure 15

```
plotlist2[[3]]
```

Figure 16

```
plotlist2[[4]]
```

MNAR

Figure 17

```
plotlist2[[5]]
```

Figure 18

```
plotlist2[[6]]
```

```
### this produces Figure 3 in the manuscript ###

idx3 <- which(res_long$mech == "MAR" & res_long$p!=0.5 & res_long$AUC_0!=0.9 & res_long$r!=0.5 & res_long$N==100)
idx4 <- which(res_long$mech == "MAR" & res_long$p!=0.5 & res_long$AUC_0!=0.9 & res_long$r!=0.5 & res_long$N==500 )


 plot_pub2.1 <- ggplot(res_long[idx3,], aes(x = pm, y = RMSE, group = Method)) +
                      geom_line(aes(color=Method, linetype = Method), linewidth=1) +
                      scale_color_manual(values = colors) +
                      scale_x_continuous(breaks = c(0.1,0.3,0.5)) +
                      xlab("Proportion of missing values") + ylab("Root mean squared error") +
                      facet_grid(r   ~ AUC_0 + p, labeller = label_both, scales="free") + 
                          theme(axis.title = element_text(size = 18),
                              axis.text = element_text(size = 14),
                              plot.title = element_text(size = 20),
                              strip.text.x = element_text(size = 14),
                              strip.text.y = element_text(size = 14),
                              legend.position = "none",
                              axis.text.x = element_text(angle = 45, hjust = 1))

plot_pub2.2 <- ggplot(res_long[idx4,], aes(x = pm, y = RMSE, group = Method)) +
                      geom_line(aes(color=Method, linetype = Method), linewidth=1) +
                      scale_color_manual(values = colors) +
                      scale_x_continuous(breaks = c(0.1,0.3,0.5)) +
                      xlab("Proportion of missing values") + ylab("Root mean squared error") +
                      facet_grid(r   ~ AUC_0 + p, labeller = label_both, scales="free") + 
                          theme(axis.title = element_text(size = 18),
                              axis.text = element_text(size = 14),
                              plot.title = element_text(size = 20),
                              strip.text.x = element_text(size = 14),
                              strip.text.y = element_text(size = 14),
                              axis.text.x = element_text(angle = 45, hjust = 1))

fig2 <- ggarrange(plot_pub2.1, plot_pub2.2, labels=c("A","B"), ncol=2)
fig2
ggsave("./Analyse/Ergebnisse/Plots/RMSE_MAR_pub.png", width = 20, height = 10)
```

### 4.2.3 Coverage probability

```
# calculate 95% Monte carlo CI for coverage
res_long$MC_cov_ciu <- res_long$Coverage+1.96*res_long$MCE_cov
res_long$MC_cov_cil <- res_long$Coverage-1.96*res_long$MCE_cov

# variable indicating whether bias is "too high" (bias>5%)
res_long$rel_bias <- (res_long$Bias/res_long$AUC_0)*100 # relative bias in %
res_long$bias_cut <- as.factor(if_else(res_long$rel_bias>=5 | res_long$rel_bias<=(-5), "too biased (>=5%)", "acceptable biased"))

idx1 <- which(res_long$mech == "MCAR" & res_long$N==100 & res_long$r!=0.5 & res_long$AUC_0!=0.85 & res_long$p!=0.3 & res_long$pm!=0.3)
idx2 <- which(res_long$mech == "MCAR" & res_long$N==500 & res_long$r!=0.5 & res_long$AUC_0!=0.85 & res_long$p!=0.3 & res_long$pm!=0.3)

idx3 <- which(res_long$mech == "MAR" & res_long$N==100 & res_long$r!=0.5 & res_long$AUC_0!=0.85 & res_long$p!=0.3 & res_long$pm!=0.3)
idx4 <- which(res_long$mech == "MAR" & res_long$N==500 & res_long$r!=0.5 & res_long$AUC_0!=0.85 & res_long$p!=0.3 & res_long$pm!=0.3)

idx5 <- which(res_long$mech == "MNAR" & res_long$N==100 & res_long$r!=0.5 & res_long$AUC_0!=0.85 & res_long$p!=0.3 & res_long$pm!=0.3)
idx6 <- which(res_long$mech == "MNAR" & res_long$N==500 & res_long$r!=0.5 & res_long$AUC_0!=0.85 & res_long$p!=0.3 & res_long$pm!=0.3)

idxlist <- list(idx1, idx2, idx3, idx4, idx5, idx6)
title <- c("Coverage for MCAR and N = 100", "Coverage for MCAR and N = 1000",
           "Coverage for MAR and N = 100", "Coverage for MAR and N = 1000",
           "Coverage for MNAR and N = 100", "Coverage for MNAR and N = 1000")

# lollipop plot coverage
plotlist3 <- list()
for (i in 1:length(idxlist)) {
  plotlist3[[i]] <- ggplot(res_long[idxlist[[i]],], aes(y=Method, x=Coverage, color=bias_cut)) +
                      geom_segment( aes(y=Method, yend=Method, x=0.95, xend=Coverage)) +
                      geom_point(size=2) +
                      geom_vline(xintercept=0.95) +
                      geom_text(aes(MC_cov_ciu, Method, label = ")")) +
                      geom_text(aes(MC_cov_cil, Method, label = "(")) +
                      facet_grid(pm + r ~ p + AUC_0, labeller = label_both) + # pm~p
                      scale_color_grey() +
                      ggtitle(title[i]) +
                      labs(color="Bias categorized") +
                      theme(axis.title = element_text(size = 18),
                              axis.text = element_text(size = 16),
                              plot.title = element_text(size = 20),
                              strip.text.x = element_text(size = 14),
                              strip.text.y = element_text(size = 14),
                              legend.position = "bottom")
}
```

MCAR

Figure 19

```
plotlist3[[1]]
```

Figure 20

```
plotlist3[[2]]
```

MAR

Figure 21

```
plotlist3[[3]]
```

Figure 22

```
plotlist3[[4]]
```

MNAR

Figure 23

```
plotlist3[[5]]
```

Figure 24

```
plotlist3[[6]]
```

```
### this produces Figure 4 in the manuscript ###

idx3 <- which(res_long$mech == "MAR" & res_long$N==100 & res_long$r!=0.5 & res_long$AUC_0!=0.85 & res_long$p!=0.3 & res_long$pm!=0.3)
idx4 <- which(res_long$mech == "MAR" & res_long$N==500 & res_long$r!=0.5 & res_long$AUC_0!=0.85 & res_long$p!=0.3 & res_long$pm!=0.3)


plot_pub3.1 <- ggplot(res_long[idx3,], aes(y=Method, x=Coverage, , color=bias_cut)) +
                      geom_segment( aes(y=Method, yend=Method, x=0.95, xend=Coverage)) +
                      geom_point(size=2) +
                      geom_vline(xintercept=0.95) +
                      geom_text(aes(MC_cov_ciu, Method, label = ")")) +
                      geom_text(aes(MC_cov_cil, Method, label = "(")) +
                      scale_color_grey() +
                      labs(color="Bias categorized") +
                      facet_grid(pm + r ~ p + AUC_0, labeller = label_both) + 
                          theme(axis.title = element_text(size = 18),
                              axis.text = element_text(size = 16),
                              plot.title = element_text(size = 20),
                              strip.text.x = element_text(size = 14),
                              strip.text.y = element_text(size = 14),
                              legend.position = "bottom")

plot_pub3.2 <- ggplot(res_long[idx4,], aes(y=Method, x=Coverage, , color=bias_cut)) +
                      geom_segment( aes(y=Method, yend=Method, x=0.95, xend=Coverage)) +
                      geom_point(size=2) +
                      geom_vline(xintercept=0.95) +
                      geom_text(aes(MC_cov_ciu, Method, label = ")")) +
                      geom_text(aes(MC_cov_cil, Method, label = "(")) +
                      scale_color_grey() +
                      labs(color="Bias categorized") +
                      facet_grid(pm + r ~ p + AUC_0, labeller = label_both) + 
                          theme(axis.title = element_text(size = 18),
                              axis.text = element_text(size = 16),
                              plot.title = element_text(size = 20),
                              strip.text.x = element_text(size = 14),
                              strip.text.y = element_text(size = 14),
                              legend.position = "bottom")


# lollipop plot coverage
plot_pub3 <- list()
for (i in 1:length(idxlist)) {
  plot_pub3[[i]] <- ggplot(res_long[idxlist[[i]],], aes(y=Method, x=Coverage, , color=bias_cut)) +
                      geom_segment( aes(y=Method, yend=Method, x=0.95, xend=Coverage)) +
                      geom_point(size=2) +
                      geom_vline(xintercept=0.95) +
                      geom_text(aes(MC_cov_ciu, Method, label = ")")) +
                      geom_text(aes(MC_cov_cil, Method, label = "(")) +
                      scale_color_grey() +
                      facet_grid(pm + r ~ p + AUC_0, labeller = label_both) + 
                          theme(axis.title = element_text(size = 18),
                              axis.text = element_text(size = 16),
                              plot.title = element_text(size = 20),
                              strip.text.x = element_text(size = 14),
                              strip.text.y = element_text(size = 14),
                              legend.position = "bottom")
}

fig3 <- ggarrange(plot_pub3.1, plot_pub3.2, labels=c("A","B"), ncol=2, common.legend = TRUE, legend = "bottom")
fig3
ggsave("./Analyse/Ergebnisse/Plots/cov_MAR_pub.png", width = 20, height = 10)
```

### 4.2.4 Power

```
idx1 <- which(res_long$mech == "MCAR" & res_long$N!=500 & res_long$p!=0.3 & res_long$r!=0.5 & res_long$AUC_0!=0.85)

idx2 <- which(res_long$mech == "MAR" & res_long$N!=500 & res_long$p!=0.3 & res_long$r!=0.5 & res_long$AUC_0!=0.85)

idx3 <- which(res_long$mech == "MNAR" & res_long$N!=500 & res_long$p!=0.3 & res_long$r!=0.5 & res_long$AUC_0!=0.85)

idxlist <- list(idx1, idx2, idx3)

title <- c("Power for MCAR", 
           "Power for MAR", 
           "Power for MNAR")

# lineplot for power
plotlist4 <- list()
for (i in 1:length(idxlist)) {
  plotlist4[[i]] <- ggplot(res_long[idxlist[[i]],], aes(x = pm, y = Power, group = Method)) +
                            geom_line(aes(color=Method, linetype=Method), linewidth=1) +
                            scale_x_continuous(breaks = c(0.1,0.3,0.5)) +
                            scale_color_manual(values = colors) +
                            xlab("Proportion of missing values") + ylab("Power") +
                            facet_grid(N + AUC_0 ~ p + r, labeller = label_both) +
                            ggtitle(title[i]) +
                            theme(axis.title = element_text(size = 18),
                              axis.text = element_text(size = 16),
                              plot.title = element_text(size = 20),
                              strip.text.x = element_text(size = 14),
                              strip.text.y = element_text(size = 14),
                              legend.title = element_blank(),
                              legend.text = element_text(size = 14),
                              legend.position = "right")
}
```

MCAR

Figure 25

```
plotlist4[[1]]
```

MAR

Figure 26

```
plotlist4[[2]]
```

MNAR

Figure 27

```
plotlist4[[3]]
```

```
### this produces Figure 5 in the manuscript ###

idx2 <- which(res_long$mech == "MAR" & res_long$N!=500 & res_long$p!=0.3 & res_long$r!=0.5 & res_long$AUC_0!=0.85)

# lineplot for power
plot_pub4 <- ggplot(res_long[idx2,], aes(x = pm, y = Power, group = Method)) +
                            geom_line(aes(color=Method, linetype=Method), linewidth=1) +
                            scale_x_continuous(breaks = c(0.1,0.3,0.5)) +
                            scale_color_manual(values = colors) +
                            xlab("Proportion of missing values") + ylab("Power") +
                            facet_grid(N + AUC_0 ~ p + r, labeller = label_both) +
                            theme(axis.title = element_text(size = 18),
                              axis.text = element_text(size = 14),
                              plot.title = element_text(size = 20),
                              strip.text.x = element_text(size = 14),
                              strip.text.y = element_text(size = 14),
                              legend.title = element_blank(),
                              legend.text = element_text(size = 14),
                              legend.position = "none")
plot_pub4
ggsave("./Analyse/Ergebnisse/Plots/Power_MAR_pub.png", width = 20, height = 10)
```

### 4.2.5 Average running time

```
idx1 <- which(res_long$N==100 &  res_long$AUC_0==0.85 & res_long$r==0.5 & res_long$p==0.3 )
idx2 <- which(res_long$N==1000 &  res_long$AUC_0==0.85 & res_long$r==0.5 & res_long$p==0.3 )
idxlist <- list(idx1, idx2)

title <- c("Average running time for N=100", 
           "Average running time for N=1000")

# lineplot for av running time (similar for different AUCs -> across all true AUCs)
plotlist5 <- list()
for (i in 1:length(idxlist)) {
  plotlist5[[i]] <- ggplot(res_long[idxlist[[i]],], aes(x = pm, y = av_time, group = Method)) +
                            geom_line(aes(color=Method, linetype = Method), linewidth=1) +
                            scale_x_continuous(breaks = c(0.1,0.3,0.5)) +
                            scale_color_manual(values = colors) +
                            xlab("Proportion of missing values") + ylab("Running time in seconds") +
                            facet_grid(  ~ mech, labeller = label_both) +
                            ggtitle(title[i]) +
                            theme(axis.title = element_text(size = 18),
                              axis.text = element_text(size = 16),
                              plot.title = element_text(size = 20),
                              strip.text.x = element_text(size = 14),
                              strip.text.y = element_text(size = 14),
                              legend.title = element_blank(),
                              legend.text = element_text(size = 14),
                              legend.position = "right")
}
```

Figure 28

```
plotlist5[[1]]
```

Figure 29

```
plotlist5[[2]]
```

## 4.3 Overview of Monte Carlo Standard Errors

Table 6. Summary statistics of Monte Carlo Standard Errors summarized
across all scenarios and iterations

```
# table with MCE across all scenarios 
summary(tableby(Method ~ ., data = res_long[,c("Method", "MCE_bias","MCE_MSE", "MCE_cov", "MCE_be_cov", "MCE_power")], control = mycontrols), pfootnote = T)
```

|  | AIPW (N=729) | CCA (N=729) | HDEL (N=729) | KER (N=729) | MI2 (N=729) | MIB2 (N=729) | mice (N=729) | mix (N=729) | Total (N=5832) |
| --- | --- | --- | --- | --- | --- | --- | --- | --- | --- |
| **Monte Carlo Standard Error for Bias** |  |  |  |  |  |  |  |  |  |
| Mean | 0.001 | 0.001 | 0.001 | 0.001 | 0.001 | 0.001 | 0.001 | 0.001 | 0.001 |
| SD | 0.001 | 0.001 | 0.001 | 0.001 | 0.001 | 0.001 | 0.001 | 0.001 | 0.001 |
| Median (Q1, Q3) | 0.001 (0.001, 0.001) | 0.001 (0.001, 0.002) | 0.001 (0.001, 0.002) | 0.001 (0.001, 0.002) | 0.001 (0.001, 0.002) | 0.001 (0.001, 0.002) | 0.001 (0.001, 0.001) | 0.001 (0.001, 0.002) | 0.001 (0.001, 0.002) |
| Range | 0.000 - 0.004 | 0.000 - 0.004 | 0.000 - 0.006 | 0.000 - 0.006 | 0.000 - 0.004 | 0.000 - 0.004 | 0.000 - 0.003 | 0.000 - 0.003 | 0.000 - 0.006 |
| **Monte Carlo Standard Error for Mean Squared Error** |  |  |  |  |  |  |  |  |  |
| Mean | 0.000 | 0.000 | 0.000 | 0.000 | 0.000 | 0.000 | 0.000 | 0.000 | 0.000 |
| SD | 0.000 | 0.000 | 0.000 | 0.000 | 0.000 | 0.000 | 0.000 | 0.000 | 0.000 |
| Median (Q1, Q3) | 0.000 (0.000, 0.000) | 0.000 (0.000, 0.000) | 0.000 (0.000, 0.000) | 0.000 (0.000, 0.000) | 0.000 (0.000, 0.000) | 0.000 (0.000, 0.000) | 0.000 (0.000, 0.000) | 0.000 (0.000, 0.000) | 0.000 (0.000, 0.000) |
| Range | 0.000 - 0.001 | 0.000 - 0.001 | 0.000 - 0.002 | 0.000 - 0.002 | 0.000 - 0.001 | 0.000 - 0.001 | 0.000 - 0.001 | 0.000 - 0.001 | 0.000 - 0.002 |
| **Monte Carlo Standard Error for Coverage Probability** |  |  |  |  |  |  |  |  |  |
| Mean | 0.008 | 0.008 | 0.006 | 0.009 | 0.010 | 0.006 | 0.008 | 0.007 | 0.008 |
| SD | 0.001 | 0.002 | 0.002 | 0.003 | 0.002 | 0.002 | 0.001 | 0.001 | 0.002 |
| Median (Q1, Q3) | 0.008 (0.007, 0.008) | 0.008 (0.007, 0.009) | 0.006 (0.005, 0.007) | 0.008 (0.007, 0.011) | 0.010 (0.008, 0.012) | 0.005 (0.004, 0.006) | 0.008 (0.007, 0.008) | 0.007 (0.007, 0.008) | 0.007 (0.007, 0.009) |
| Range | 0.004 - 0.012 | 0.005 - 0.015 | 0.002 - 0.012 | 0.004 - 0.016 | 0.006 - 0.016 | 0.002 - 0.015 | 0.004 - 0.012 | 0.004 - 0.012 | 0.002 - 0.016 |
| **Monte Carlo Standard Error for bias-eliminated Coverage Probability** |  |  |  |  |  |  |  |  |  |
| Mean | 0.008 | 0.009 | 0.009 | 0.008 | 0.008 | 0.008 | 0.008 | 0.008 | 0.008 |
| SD | 0.002 | 0.002 | 0.002 | 0.002 | 0.002 | 0.002 | 0.002 | 0.002 | 0.002 |
| Median (Q1, Q3) | 0.008 (0.007, 0.008) | 0.008 (0.008, 0.009) | 0.008 (0.008, 0.009) | 0.008 (0.007, 0.009) | 0.008 (0.007, 0.008) | 0.008 (0.007, 0.008) | 0.008 (0.007, 0.009) | 0.008 (0.007, 0.009) | 0.008 (0.007, 0.009) |
| Range | 0.006 - 0.016 | 0.006 - 0.016 | 0.006 - 0.016 | 0.006 - 0.016 | 0.006 - 0.016 | 0.006 - 0.016 | 0.006 - 0.016 | 0.005 - 0.016 | 0.005 - 0.016 |
| **Monte Carlo Standard Error for Power** |  |  |  |  |  |  |  |  |  |
| Mean | 0.013 | 0.014 | 0.008 | 0.012 | 0.012 | 0.015 | 0.013 | 0.013 | 0.012 |
| SD | 0.003 | 0.002 | 0.005 | 0.003 | 0.003 | 0.001 | 0.003 | 0.003 | 0.004 |
| Median (Q1, Q3) | 0.014 (0.010, 0.015) | 0.015 (0.012, 0.015) | 0.008 (0.003, 0.014) | 0.013 (0.010, 0.015) | 0.013 (0.010, 0.015) | 0.015 (0.014, 0.016) | 0.014 (0.010, 0.015) | 0.014 (0.010, 0.015) | 0.014 (0.010, 0.015) |
| Range | 0.005 - 0.016 | 0.006 - 0.016 | 0.000 - 0.016 | 0.005 - 0.016 | 0.004 - 0.016 | 0.011 - 0.016 | 0.004 - 0.016 | 0.004 - 0.016 | 0.000 - 0.016 |

## 4.4 Nested loop plots for the “big picture”

Figure 30. Nested loop plot of bias restricted to scenarios where the
proportion of missing values in the index test is set to 50%

```
# only pm=0.5
s1 <- simsum(data = dat_long[which(dat_long$pm==0.5),], estvarname = "AUC", ref="CCA", true = "AUC_0", methodvar = "Method", by=c("AUC_0","r","p", "N","mech"))
#summary(s1)

ap <- autoplot(s1, type = "nlp", stats = "bias")
ap + scale_color_manual(values = colors)
```

Figure 31. Nested loop plot of bias restricted to scenarios where the
proportion of missing values in the index test is set to 50% and
missingness mechanism is MAR

```
# only pm=0.5 and mech=MAR
s1 <- simsum(data = dat_long[which(dat_long$pm==0.5 & dat_long$mech=="MAR"),], estvarname = "AUC", ref="CCA", true = "AUC_0", methodvar = "Method", by=c("AUC_0","r","p", "N"))
#summary(s1)

ap <- autoplot(s1, type = "nlp", stats = "bias")
ap + scale_color_manual(values = colors)
```

## 4.5 Metamodels

Linear regression models were performed separately to quantify the
relative effect of the simulation factors and methods on the performance
outcome. This approach is based on the paper of van Smeden et
al. (2019).

### 4.5.1 Stratified by missingess mechanism and methods

In the first approach, linear regression models were conducted for
each method separately and stratified by missingness mechanism. The
other simulation factors were included as categorical covariates in the
regression models. Bias and RMSE were examined as outcome variables in
separate models.

```
# Function for first approach with simulation parameter (without interactions)
mm2 <- function(performance, method, mechanism) {
  
  data <- scenario_res %>%
    filter(mech == mechanism) %>%
    mutate(
      across(c(N,p,r,pm,AUC_0), as.factor),
      across(all_of(names1), abs)
    )
  
  form <- paste0(performance, ".AUC.", method, "*100", " ~ N + p + r + AUC_0 + pm")
  mm <- lm(as.formula(form), data=data)
  
  r2 <- summary(mm)$adj.r.squared
  names(r2) <- "Adjusted R squared"
  meth <- method
  names(meth) <- "Method"
  dta <- cbind(c(summary(mm)$coefficients[,1], r2),
               c(summary(mm)$coefficients[,4], NA))
  dta <- as.data.frame(t(dta))
  dta <- dta %>%
    mutate(
      Result = if_else(is.na(`Adjusted R squared`), "P-values", "Estimate"),
      Method = method,
      Mechanism = mechanism
    ) %>%
    relocate(any_of(c("Mechanism", "Method", "Result")), .before="(Intercept)")
  
  return((dta))
}

# Function for first approach with simulation parameter and interactions interactions)
mm3 <- function(performance, method, mechanism) {
  
  data <- scenario_res %>%
    filter(mech == mechanism) %>%
    mutate(
      across(c(N,p,r,pm,AUC_0), as.factor),
      across(all_of(names1), abs)
    ) 
  
  form <- paste0(performance, ".AUC.", method, "*100", " ~ 
                 ( N + p + r + AUC_0 + pm)^2")
  mm <- lm(as.formula(form), data=data)
  
  r2 <- summary(mm)$adj.r.squared
  names(r2) <- "Adjusted R squared"
  meth <- method
  names(meth) <- "Method"
  dta <- cbind(c(summary(mm)$coefficients[,1], r2),
               c(summary(mm)$coefficients[,4], NA))
  dta <- as.data.frame(t(dta))
  dta <- dta %>%
    mutate(
      Result = if_else(is.na(`Adjusted R squared`), "P-values", "Estimate"),
      Method = method,
      Mechanism = mechanism
    ) %>%
    relocate(any_of(c("Mechanism", "Method", "Result")), .before="(Intercept)")
  
  return((dta))
}

# Function for first approach with simulation parameter and interactions interactions (and backward selection)
mm4 <- function(performance, method, mechanism) {
  
  data <- scenario_res %>%
    filter(mech == mechanism) %>%
    mutate(
      across(c(N,p,r,pm,AUC_0), as.factor),
      across(all_of(names1), abs)
    )
  
  form <- paste0(performance, ".AUC.", method, "*100", " ~ 
                 (N + p + r + AUC_0 + pm)^2")
  mm <- lm(as.formula(form), data=data)
  
  invisible(capture.output(backward <- stats::step(mm, direction='backward')))
  #backward$anova
  #backward$coefficients
  
  r2 <- summary(backward)$adj.r.squared
  names(r2) <- "Adjusted R squared"
  meth <- method
  names(meth) <- "Method"
  dta <- cbind(c(summary(backward)$coefficients[,1], r2),
               c(summary(backward)$coefficients[,4], NA))
  dta <- as.data.frame(t(dta))
  dta <- dta %>%
    mutate(
      Result = if_else(is.na(`Adjusted R squared`), "P-values", "Estimate"),
      Method = method,
      Mechanism = mechanism
    ) %>%
    relocate(any_of(c("Mechanism", "Method", "Result")), .before="(Intercept)")
  
  return((dta))
}
```

```
# first approach without interactions; results are not shown

# bias
dta_bias2 <- c()
for (j in c("MCAR", "MAR", "MNAR")) {
  for (i in 1:length(methods)) {
    
      dta <- mm2("bias", methods[i], mechanism=j)
      dta_bias2 <- rbind(dta_bias2, dta)
    
  }
}

tabno <- tabno+1
tab_mm_bias2 <- dta_bias2 %>%
  arrange(Method, Mechanism) %>%
  gt(groupname_col = c("Method", "Mechanism")) %>%
  row_group_order(
    groups = c("CCA - MCAR", "CCA - MAR", "CCA - MNAR",
               "AIPW - MCAR", "AIPW - MAR", "AIPW - MNAR",
               "HDEL - MCAR", "HDEL - MAR", "HDEL - MNAR",
               "KER - MCAR", "KER - MAR", "KER - MNAR",
               "MI2 - MCAR", "MI2 - MAR", "MI2 - MNAR",
               "MIB2 - MCAR", "MIB2 - MAR", "MIB2 - MNAR",
               "mice - MCAR", "mice - MAR", "mice - MNAR",
               "mix - MCAR", "mix - MAR", "mix - MNAR"
               )
  ) %>%
  fmt_number(
    columns = where(is.numeric),
    rows = Result=="Estimate",
    decimals = 2
  ) %>%
  fmt_number(
    columns = where(is.numeric),
    rows = Result=="P-values",
    decimals = 3
  ) %>%
  sub_missing(
  columns = everything(),
  rows = everything(),
  missing_text = ""
  ) %>%
  tab_header(
    title = paste0("Table ", tabno, ". Results for the metamodels with the outcome: Bias")
  ) %>%
  tab_footnote(
    footnote = "Ref. (reference group): N (sample size) = 100",
    locations = cells_column_labels(columns = c(N500, N1000))
  ) %>%
  tab_footnote(
    footnote = "Ref.: p (prevalence) = 0.1",
    locations = cells_column_labels(columns = c(p0.3, p0.5))
  ) %>%
  tab_footnote(
    footnote = "Ref.: r (correlation) = 0.2",
    locations = cells_column_labels(columns = c(r0.5, r0.9))
  ) %>%
  tab_footnote(
    footnote = "Ref.: AUC_0 (true AUC) = 0.7",
    locations = cells_column_labels(columns = c(AUC_00.85, AUC_00.9))
  ) %>%
  tab_footnote(
    footnote = "Ref.: pm (proportion of missing values) = 0.1",
    locations = cells_column_labels(columns = c(pm0.3, pm0.5))
  ) %>%
  tab_options(footnotes.multiline = FALSE)
tab_mm_bias2
```

```
# first approach with interactions; results are not shown

# bias
dta_bias3 <- c()
for (j in c("MCAR", "MAR", "MNAR")) {
  for (i in 1:length(methods)) {
    
      dta <- mm3("bias", methods[i], mechanism=j)
      dta_bias3 <- rbind(dta_bias3, dta)
    
  }
}

tabno <- tabno+1
tab_mm_bias3 <- dta_bias3 %>%
  arrange(Method, Mechanism) %>%
  gt(groupname_col = c("Method", "Mechanism")) %>%
  row_group_order(
    groups = c("CCA - MCAR", "CCA - MAR", "CCA - MNAR",
               "AIPW - MCAR", "AIPW - MAR", "AIPW - MNAR",
               "HDEL - MCAR", "HDEL - MAR", "HDEL - MNAR",
               "KER - MCAR", "KER - MAR", "KER - MNAR",
               "MI2 - MCAR", "MI2 - MAR", "MI2 - MNAR",
               "MIB2 - MCAR", "MIB2 - MAR", "MIB2 - MNAR",
               "mice - MCAR", "mice - MAR", "mice - MNAR",
               "mix - MCAR", "mix - MAR", "mix - MNAR"
               )
  ) %>%
  fmt_number(
    columns = where(is.numeric),
    rows = Result=="Estimate",
    decimals = 2
  ) %>%
  fmt_number(
    columns = where(is.numeric),
    rows = Result=="P-values",
    decimals = 3
  ) %>%
  sub_missing(
  columns = everything(),
  rows = everything(),
  missing_text = ""
  ) %>%
  tab_header(
    title = paste0("Table ", tabno, ". Results for the metamodels with all interaction terms with the outcome: Bias")
  ) %>%
  tab_footnote(
    footnote = "Ref. (reference group): N (sample size) = 100",
    locations = cells_column_labels(columns = c(N500, N1000))
  ) %>%
  tab_footnote(
    footnote = "Ref.: p (prevalence) = 0.1",
    locations = cells_column_labels(columns = c(p0.3, p0.5))
  ) %>%
  tab_footnote(
    footnote = "Ref.: r (correlation) = 0.2",
    locations = cells_column_labels(columns = c(r0.5, r0.9))
  ) %>%
  tab_footnote(
    footnote = "Ref.: AUC_0 (true AUC) = 0.7",
    locations = cells_column_labels(columns = c(AUC_00.85, AUC_00.9))
  ) %>%
  tab_footnote(
    footnote = "Ref.: pm (proportion of missing values) = 0.1",
    locations = cells_column_labels(columns = c(pm0.3, pm0.5))
  ) %>%
  tab_options(footnotes.multiline = FALSE)
tab_mm_bias3
```

Bias: Backward elimination

```
# first approach with backward selection; results are shown

# bias
dta_bias4 <- c()
for (j in c("MCAR", "MAR", "MNAR")) {
  for (i in 1:length(methods)) {
    
      dta <- mm4("bias", methods[i], mechanism=j)
      dta_bias4 <- dta_bias4 %>%
        bind_rows(dta)
    
  }
}

tabno <- tabno+1
tab_mm_bias4 <- dta_bias4 %>%
  arrange(Method, Mechanism) %>%
  gt(groupname_col = c("Method", "Mechanism")) %>%
  row_group_order(
    groups = c("CCA - MCAR", "CCA - MAR", "CCA - MNAR",
               "AIPW - MCAR", "AIPW - MAR", "AIPW - MNAR",
               "HDEL - MCAR", "HDEL - MAR", "HDEL - MNAR",
               "KER - MCAR", "KER - MAR", "KER - MNAR",
               "MI2 - MCAR", "MI2 - MAR", "MI2 - MNAR",
               "MIB2 - MCAR", "MIB2 - MAR", "MIB2 - MNAR",
               "mice - MCAR", "mice - MAR", "mice - MNAR",
               "mix - MCAR", "mix - MAR", "mix - MNAR"
               )
  ) %>%
  fmt_number(
    columns = where(is.numeric),
    rows = Result=="Estimate",
    decimals = 2
  ) %>%
  fmt_number(
    columns = where(is.numeric),
    rows = Result=="P-values",
    decimals = 3
  ) %>%
  sub_missing(
  columns = everything(),
  rows = everything(),
  missing_text = ""
  ) %>%
  tab_header(
    title = paste0("Table ", tabno, ". Results for the metamodels with interactions and backward elimination and the outcome: Bias")
  ) %>%
  tab_footnote(
    footnote = "Ref. (reference group): N (sample size) = 100",
    locations = cells_column_labels(columns = c(N500, N1000))
  ) %>%
  tab_footnote(
    footnote = "Ref.: p (prevalence) = 0.1",
    locations = cells_column_labels(columns = c(p0.3, p0.5))
  ) %>%
  tab_footnote(
    footnote = "Ref.: r (correlation) = 0.2",
    locations = cells_column_labels(columns = c(r0.5, r0.9))
  ) %>%
  tab_footnote(
    footnote = "Ref.: AUC_0 (true AUC) = 0.7",
    locations = cells_column_labels(columns = c(AUC_00.85, AUC_00.9))
  ) %>%
  tab_footnote(
    footnote = "Ref.: pm (proportion of missing values) = 0.1",
    locations = cells_column_labels(columns = c(pm0.3, pm0.5))
  ) %>%
  tab_options(footnotes.multiline = FALSE)
tab_mm_bias4
```

|  |  |  |  |  |  |  |  |  |  |  |  |  |  |  |  |  |  |  |  |  |  |  |  |  |  |  |  |  |  |  |  |  |  |  |  |  |  |  |  |  |  |  |  |  |  |  |  |  |  |  |  |  |
| --- | --- | --- | --- | --- | --- | --- | --- | --- | --- | --- | --- | --- | --- | --- | --- | --- | --- | --- | --- | --- | --- | --- | --- | --- | --- | --- | --- | --- | --- | --- | --- | --- | --- | --- | --- | --- | --- | --- | --- | --- | --- | --- | --- | --- | --- | --- | --- | --- | --- | --- | --- | --- |
| Table 7. Results for the metamodels with interactions and backward elimination and the outcome: Bias | | | | | | | | | | | | | | | | | | | | | | | | | | | | | | | | | | | | | | | | | | | | | | | | | | | | |
| Result | (Intercept) | N5001 | N10001 | p0.32 | p0.52 | AUC\_00.853 | AUC\_00.93 | pm0.34 | pm0.54 | N500:p0.3 | N1000:p0.3 | N500:p0.5 | N1000:p0.5 | N500:AUC\_00.85 | N1000:AUC\_00.85 | N500:AUC\_00.9 | N1000:AUC\_00.9 | N500:pm0.3 | N1000:pm0.3 | N500:pm0.5 | N1000:pm0.5 | p0.3:AUC\_00.85 | p0.5:AUC\_00.85 | p0.3:AUC\_00.9 | p0.5:AUC\_00.9 | AUC\_00.85:pm0.3 | AUC\_00.9:pm0.3 | AUC\_00.85:pm0.5 | AUC\_00.9:pm0.5 | Adjusted R squared | r0.55 | r0.95 | N500:r0.5 | N1000:r0.5 | N500:r0.9 | N1000:r0.9 | r0.5:AUC\_00.85 | r0.9:AUC\_00.85 | r0.5:AUC\_00.9 | r0.9:AUC\_00.9 | r0.5:pm0.3 | r0.9:pm0.3 | r0.5:pm0.5 | r0.9:pm0.5 | p0.3:pm0.3 | p0.5:pm0.3 | p0.3:pm0.5 | p0.5:pm0.5 | p0.3:r0.5 | p0.5:r0.5 | p0.3:r0.9 | p0.5:r0.9 |
| CCA - MCAR | | | | | | | | | | | | | | | | | | | | | | | | | | | | | | | | | | | | | | | | | | | | | | | | | | | | |
| --- | --- | --- | --- | --- | --- | --- | --- | --- | --- | --- | --- | --- | --- | --- | --- | --- | --- | --- | --- | --- | --- | --- | --- | --- | --- | --- | --- | --- | --- | --- | --- | --- | --- | --- | --- | --- | --- | --- | --- | --- | --- | --- | --- | --- | --- | --- | --- | --- | --- | --- | --- | --- |
| Estimate | 0.33 | −0.16 | −0.25 | −0.22 | −0.19 | −0.18 | −0.19 | 0.02 | 0.19 | 0.11 | 0.14 | 0.10 | 0.13 | 0.11 | 0.14 | 0.10 | 0.13 | −0.03 | −0.02 | −0.13 | −0.09 | 0.09 | 0.07 | 0.11 | 0.09 | 0.01 | 0.01 | −0.11 | −0.11 | 0.44 |  |  |  |  |  |  |  |  |  |  |  |  |  |  |  |  |  |  |  |  |  |  |
| P-values | 0.000 | 0.000 | 0.000 | 0.000 | 0.000 | 0.000 | 0.000 | 0.618 | 0.000 | 0.002 | 0.000 | 0.008 | 0.000 | 0.002 | 0.000 | 0.007 | 0.001 | 0.383 | 0.493 | 0.000 | 0.009 | 0.013 | 0.045 | 0.004 | 0.011 | 0.719 | 0.848 | 0.002 | 0.002 |  |  |  |  |  |  |  |  |  |  |  |  |  |  |  |  |  |  |  |  |  |  |  |
| CCA - MAR | | | | | | | | | | | | | | | | | | | | | | | | | | | | | | | | | | | | | | | | | | | | | | | | | | | | |
| Estimate | 0.51 | 0.52 | 0.48 | −0.25 | −0.40 | 0.01 | −0.23 | 0.63 | 0.50 | −0.41 | −0.36 | −0.38 | −0.35 | −0.37 | −0.31 | −0.38 | −0.35 | 0.17 | 0.16 | 0.25 | 0.16 | 0.20 | 0.33 | 0.38 | 0.58 | −0.46 | −0.57 | −0.44 | −0.42 | 0.82 | 0.65 | 0.94 |  |  |  |  | −0.31 | −0.77 | −0.40 | −0.80 | 0.14 | 0.08 | 0.06 | −0.20 | −0.11 | −0.19 | 0.08 | 0.06 | −0.28 | −0.39 | −0.36 | −0.36 |
| P-values | 0.000 | 0.000 | 0.000 | 0.049 | 0.002 | 0.958 | 0.073 | 0.000 | 0.000 | 0.000 | 0.001 | 0.000 | 0.001 | 0.000 | 0.003 | 0.000 | 0.001 | 0.098 | 0.117 | 0.018 | 0.119 | 0.055 | 0.002 | 0.000 | 0.000 | 0.000 | 0.000 | 0.000 | 0.000 |  | 0.000 | 0.000 |  |  |  |  | 0.003 | 0.000 | 0.000 | 0.000 | 0.176 | 0.443 | 0.567 | 0.054 | 0.277 | 0.065 | 0.464 | 0.534 | 0.008 | 0.000 | 0.001 | 0.001 |
| CCA - MNAR | | | | | | | | | | | | | | | | | | | | | | | | | | | | | | | | | | | | | | | | | | | | | | | | | | | | |
| Estimate | 0.64 | 0.05 | 0.05 | 0.14 | 0.14 | 0.39 | 0.35 | 0.57 | 0.56 | −0.14 | −0.14 | −0.20 | −0.21 |  |  |  |  | 0.10 | 0.14 | 0.19 | 0.19 | −0.32 | −0.54 | −0.55 | −0.62 | 0.16 | 0.13 | 0.20 | 0.14 | 0.82 |  |  |  |  |  |  |  |  |  |  |  |  |  |  | −0.13 | −0.12 | 0.07 | 0.16 |  |  |  |  |
| P-values | 0.000 | 0.464 | 0.459 | 0.104 | 0.098 | 0.000 | 0.000 | 0.000 | 0.000 | 0.067 | 0.070 | 0.011 | 0.008 |  |  |  |  | 0.224 | 0.083 | 0.015 | 0.019 | 0.000 | 0.000 | 0.000 | 0.000 | 0.047 | 0.110 | 0.012 | 0.068 |  |  |  |  |  |  |  |  |  |  |  |  |  |  |  | 0.104 | 0.115 | 0.385 | 0.042 |  |  |  |  |
| AIPW - MCAR | | | | | | | | | | | | | | | | | | | | | | | | | | | | | | | | | | | | | | | | | | | | | | | | | | | | |
| Estimate | 0.48 | −0.45 | −0.52 | −0.43 | −0.48 |  |  | 0.28 | 0.68 | 0.41 | 0.45 | 0.46 | 0.50 |  |  |  |  | −0.16 | −0.15 | −0.42 | −0.38 |  |  |  |  |  |  |  |  | 0.58 | −0.11 | −0.26 | 0.15 | 0.13 | 0.24 | 0.26 |  |  |  |  | −0.07 | −0.08 | −0.14 | −0.21 | −0.13 | −0.13 | −0.23 | −0.25 | 0.09 | 0.10 | 0.16 | 0.18 |
| P-values | 0.000 | 0.000 | 0.000 | 0.000 | 0.000 |  |  | 0.000 | 0.000 | 0.000 | 0.000 | 0.000 | 0.000 |  |  |  |  | 0.021 | 0.028 | 0.000 | 0.000 |  |  |  |  |  |  |  |  |  | 0.137 | 0.000 | 0.028 | 0.064 | 0.001 | 0.000 |  |  |  |  | 0.344 | 0.265 | 0.038 | 0.002 | 0.062 | 0.054 | 0.001 | 0.000 | 0.172 | 0.144 | 0.021 | 0.010 |
| AIPW - MAR | | | | | | | | | | | | | | | | | | | | | | | | | | | | | | | | | | | | | | | | | | | | | | | | | | | | |
| Estimate | 1.29 | −1.38 | −1.58 | −1.29 | −1.52 |  |  | 0.84 | 2.11 | 1.63 | 1.80 | 1.83 | 2.01 |  |  |  |  | −0.48 | −0.48 | −1.14 | −1.30 |  |  |  |  |  |  |  |  | 0.74 | 0.17 | −0.57 | −0.13 | −0.08 | 0.27 | 0.39 |  |  |  |  |  |  |  |  | −0.53 | −0.52 | −1.14 | −1.24 | −0.15 | −0.10 | 0.21 | 0.31 |
| P-values | 0.000 | 0.000 | 0.000 | 0.000 | 0.000 |  |  | 0.000 | 0.000 | 0.000 | 0.000 | 0.000 | 0.000 |  |  |  |  | 0.005 | 0.005 | 0.000 | 0.000 |  |  |  |  |  |  |  |  |  | 0.286 | 0.000 | 0.463 | 0.622 | 0.116 | 0.025 |  |  |  |  |  |  |  |  | 0.002 | 0.002 | 0.000 | 0.000 | 0.384 | 0.561 | 0.225 | 0.066 |
| AIPW - MNAR | | | | | | | | | | | | | | | | | | | | | | | | | | | | | | | | | | | | | | | | | | | | | | | | | | | | |
| Estimate | 1.24 | −0.96 | −0.99 | −1.11 | −1.27 | 0.99 | 0.75 | 1.37 | 2.33 | 1.52 | 1.60 | 1.71 | 1.79 | −0.50 | −0.53 | −0.37 | −0.43 | −0.48 | −0.48 | −1.02 | −1.11 | −0.40 | −0.58 | −0.49 | −0.59 | 0.14 | 0.15 | 0.40 | 0.39 | 0.84 | −0.24 | −0.64 |  |  |  |  | −0.34 | −0.42 | −0.21 | −0.20 | −0.22 | −0.54 | −0.35 | −0.80 | −0.61 | −0.62 | −0.92 | −1.06 | 0.15 | 0.16 | 0.29 | 0.43 |
| P-values | 0.000 | 0.000 | 0.000 | 0.000 | 0.000 | 0.000 | 0.000 | 0.000 | 0.000 | 0.000 | 0.000 | 0.000 | 0.000 | 0.001 | 0.001 | 0.018 | 0.006 | 0.002 | 0.002 | 0.000 | 0.000 | 0.010 | 0.000 | 0.002 | 0.000 | 0.373 | 0.327 | 0.011 | 0.012 |  | 0.158 | 0.000 |  |  |  |  | 0.031 | 0.007 | 0.172 | 0.189 | 0.160 | 0.001 | 0.026 | 0.000 | 0.000 | 0.000 | 0.000 | 0.000 | 0.337 | 0.301 | 0.064 | 0.006 |
| HDEL - MCAR | | | | | | | | | | | | | | | | | | | | | | | | | | | | | | | | | | | | | | | | | | | | | | | | | | | | |
| Estimate | 0.21 | −0.06 | −0.10 | −0.04 | −0.05 | −0.06 | −0.07 | 0.01 | 0.03 |  |  |  |  |  |  |  |  |  |  |  |  |  |  |  |  |  |  |  |  | 0.28 |  |  |  |  |  |  |  |  |  |  |  |  |  |  |  |  |  |  |  |  |  |  |
| P-values | 0.000 | 0.000 | 0.000 | 0.005 | 0.000 | 0.000 | 0.000 | 0.288 | 0.030 |  |  |  |  |  |  |  |  |  |  |  |  |  |  |  |  |  |  |  |  |  |  |  |  |  |  |  |  |  |  |  |  |  |  |  |  |  |  |  |  |  |  |  |
| HDEL - MAR | | | | | | | | | | | | | | | | | | | | | | | | | | | | | | | | | | | | | | | | | | | | | | | | | | | | |
| Estimate | 0.91 | 0.00 | 0.02 | −0.71 | −0.85 | −0.30 | −0.61 | 0.85 | 1.17 |  |  |  |  |  |  |  |  | 0.06 | 0.07 | −0.14 | −0.21 | 0.44 | 0.56 | 0.68 | 0.88 | −0.55 | −0.69 | −0.52 | −0.55 | 0.87 | 0.72 | 1.03 |  |  |  |  | −0.42 | −0.89 | −0.50 | −0.90 | 0.13 | 0.06 | 0.06 | −0.24 | −0.20 | −0.27 | −0.25 | −0.26 | −0.29 | −0.39 | −0.36 | −0.34 |
| P-values | 0.000 | 0.961 | 0.782 | 0.000 | 0.000 | 0.004 | 0.000 | 0.000 | 0.000 |  |  |  |  |  |  |  |  | 0.552 | 0.463 | 0.133 | 0.026 | 0.000 | 0.000 | 0.000 | 0.000 | 0.000 | 0.000 | 0.000 | 0.000 |  | 0.000 | 0.000 |  |  |  |  | 0.000 | 0.000 | 0.000 | 0.000 | 0.193 | 0.559 | 0.538 | 0.014 | 0.038 | 0.005 | 0.011 | 0.008 | 0.003 | 0.000 | 0.000 | 0.001 |
| HDEL - MNAR | | | | | | | | | | | | | | | | | | | | | | | | | | | | | | | | | | | | | | | | | | | | | | | | | | | | |
| Estimate | 0.90 | −0.26 | −0.23 | −0.25 | −0.26 | 0.28 | 0.26 | 0.70 | 1.06 | 0.30 | 0.26 | 0.27 | 0.22 |  |  |  |  | 0.04 | 0.10 | −0.09 | −0.08 | −0.16 | −0.37 | −0.39 | −0.47 | 0.14 | 0.08 | 0.17 | 0.07 | 0.90 |  |  |  |  |  |  |  |  |  |  |  |  |  |  | −0.21 | −0.22 | −0.21 | −0.16 |  |  |  |  |
| P-values | 0.000 | 0.000 | 0.000 | 0.000 | 0.000 | 0.000 | 0.000 | 0.000 | 0.000 | 0.000 | 0.000 | 0.000 | 0.001 |  |  |  |  | 0.495 | 0.118 | 0.163 | 0.176 | 0.009 | 0.000 | 0.000 | 0.000 | 0.024 | 0.171 | 0.007 | 0.267 |  |  |  |  |  |  |  |  |  |  |  |  |  |  |  | 0.001 | 0.000 | 0.001 | 0.012 |  |  |  |  |
| KER - MCAR | | | | | | | | | | | | | | | | | | | | | | | | | | | | | | | | | | | | | | | | | | | | | | | | | | | | |
| Estimate | 0.25 | −0.10 | −0.10 | 0.03 | 0.29 | 0.32 | 0.47 |  |  | −0.05 | −0.05 | −0.33 | −0.34 | −0.28 | −0.22 | −0.45 | −0.35 |  |  |  |  |  |  |  |  |  |  |  |  | 0.84 |  |  |  |  |  |  |  |  |  |  |  |  |  |  |  |  |  |  |  |  |  |  |
| P-values | 0.000 | 0.009 | 0.012 | 0.333 | 0.000 | 0.000 | 0.000 |  |  | 0.263 | 0.275 | 0.000 | 0.000 | 0.000 | 0.000 | 0.000 | 0.000 |  |  |  |  |  |  |  |  |  |  |  |  |  |  |  |  |  |  |  |  |  |  |  |  |  |  |  |  |  |  |  |  |  |  |  |
| KER - MAR | | | | | | | | | | | | | | | | | | | | | | | | | | | | | | | | | | | | | | | | | | | | | | | | | | | | |
| Estimate | 2.03 | −2.22 | −2.25 | −2.11 | −2.18 | −0.50 | −0.74 | 0.98 | 2.68 | 2.69 | 2.83 | 2.79 | 2.98 |  |  |  |  | −0.61 | −0.61 | −1.50 | −1.69 | 0.58 | 0.64 | 0.85 | 0.92 |  |  |  |  | 0.76 | 0.94 | 1.62 | −0.47 | −0.52 | −0.94 | −0.94 |  |  |  |  |  |  |  |  | −0.55 | −0.60 | −1.42 | −1.63 | −0.61 | −0.67 | −1.02 | −1.04 |
| P-values | 0.000 | 0.000 | 0.000 | 0.000 | 0.000 | 0.008 | 0.000 | 0.000 | 0.000 | 0.000 | 0.000 | 0.000 | 0.000 |  |  |  |  | 0.022 | 0.021 | 0.000 | 0.000 | 0.027 | 0.017 | 0.002 | 0.001 |  |  |  |  |  | 0.000 | 0.000 | 0.073 | 0.048 | 0.000 | 0.000 |  |  |  |  |  |  |  |  | 0.036 | 0.023 | 0.000 | 0.000 | 0.021 | 0.012 | 0.000 | 0.000 |
| KER - MNAR | | | | | | | | | | | | | | | | | | | | | | | | | | | | | | | | | | | | | | | | | | | | | | | | | | | | |
| Estimate | 1.88 | −1.69 | −1.77 | −1.24 | −1.20 |  |  | 1.04 | 2.22 | 1.95 | 1.99 | 1.92 | 1.96 |  |  |  |  | −0.39 | −0.39 | −1.05 | −1.10 |  |  |  |  |  |  |  |  | 0.75 | 0.12 | 1.01 | −0.46 | −0.47 | −1.12 | −1.04 |  |  |  |  |  |  |  |  | −0.50 | −0.54 | −1.04 | −1.08 | −0.22 | −0.24 | −0.91 | −0.97 |
| P-values | 0.000 | 0.000 | 0.000 | 0.000 | 0.000 |  |  | 0.000 | 0.000 | 0.000 | 0.000 | 0.000 | 0.000 |  |  |  |  | 0.092 | 0.088 | 0.000 | 0.000 |  |  |  |  |  |  |  |  |  | 0.566 | 0.000 | 0.048 | 0.042 | 0.000 | 0.000 |  |  |  |  |  |  |  |  | 0.030 | 0.020 | 0.000 | 0.000 | 0.334 | 0.287 | 0.000 | 0.000 |
| MI2 - MCAR | | | | | | | | | | | | | | | | | | | | | | | | | | | | | | | | | | | | | | | | | | | | | | | | | | | | |
| Estimate | 0.29 | −0.04 | −0.05 | −0.27 | −0.35 | −0.16 | −0.22 | 0.13 | 0.29 | 0.09 | 0.11 | 0.13 | 0.15 |  |  |  |  | −0.17 | −0.17 | −0.28 | −0.34 | 0.10 | 0.17 | 0.19 | 0.22 |  |  |  |  | 0.66 | 0.05 | 0.13 | −0.02 | −0.06 | −0.21 | −0.28 | 0.01 | 0.14 | 0.01 | 0.12 | 0.04 | 0.17 | 0.09 | 0.37 |  |  |  |  |  |  |  |  |
| P-values | 0.000 | 0.548 | 0.400 | 0.000 | 0.000 | 0.002 | 0.000 | 0.018 | 0.000 | 0.122 | 0.062 | 0.023 | 0.010 |  |  |  |  | 0.005 | 0.004 | 0.000 | 0.000 | 0.091 | 0.004 | 0.001 | 0.000 |  |  |  |  |  | 0.410 | 0.044 | 0.794 | 0.294 | 0.001 | 0.000 | 0.884 | 0.017 | 0.849 | 0.048 | 0.489 | 0.003 | 0.119 | 0.000 |  |  |  |  |  |  |  |  |
| MI2 - MAR | | | | | | | | | | | | | | | | | | | | | | | | | | | | | | | | | | | | | | | | | | | | | | | | | | | | |
| Estimate | 0.71 | −0.65 | −0.85 | −0.79 | −1.10 | −0.11 | −0.39 | 0.86 | 1.81 | 1.18 | 1.47 | 1.28 | 1.57 |  |  |  |  | −0.55 | −0.61 | −0.86 | −1.18 | −0.04 | 0.17 | 0.26 | 0.45 |  |  |  |  | 0.71 | 0.51 | 0.92 | −0.34 | −0.38 | −0.81 | −0.83 |  |  |  |  | 0.14 | 0.32 | 0.32 | 0.56 | −0.55 | −0.53 | −1.15 | −1.17 | −0.38 | −0.36 | −0.59 | −0.41 |
| P-values | 0.000 | 0.001 | 0.000 | 0.001 | 0.000 | 0.419 | 0.003 | 0.000 | 0.000 | 0.000 | 0.000 | 0.000 | 0.000 |  |  |  |  | 0.003 | 0.001 | 0.000 | 0.000 | 0.813 | 0.360 | 0.162 | 0.016 |  |  |  |  |  | 0.010 | 0.000 | 0.070 | 0.041 | 0.000 | 0.000 |  |  |  |  | 0.451 | 0.080 | 0.087 | 0.003 | 0.003 | 0.004 | 0.000 | 0.000 | 0.040 | 0.054 | 0.002 | 0.027 |
| MI2 - MNAR | | | | | | | | | | | | | | | | | | | | | | | | | | | | | | | | | | | | | | | | | | | | | | | | | | | | |
| Estimate | 0.79 | −0.41 | −0.40 | −0.57 | −0.70 | 0.15 | 0.03 | 1.09 | 1.66 | 1.05 | 1.15 | 1.11 | 1.21 |  |  |  |  | −0.38 | −0.40 | −0.54 | −0.69 |  |  |  |  |  |  |  |  | 0.63 | 0.10 | 0.76 | −0.37 | −0.41 | −1.10 | −1.29 |  |  |  |  |  |  |  |  | −0.55 | −0.54 | −0.76 | −0.76 | −0.27 | −0.33 | −0.77 | −0.65 |
| P-values | 0.000 | 0.055 | 0.060 | 0.008 | 0.001 | 0.065 | 0.671 | 0.000 | 0.000 | 0.000 | 0.000 | 0.000 | 0.000 |  |  |  |  | 0.055 | 0.043 | 0.006 | 0.001 |  |  |  |  |  |  |  |  |  | 0.591 | 0.000 | 0.059 | 0.041 | 0.000 | 0.000 |  |  |  |  |  |  |  |  | 0.006 | 0.007 | 0.000 | 0.000 | 0.168 | 0.098 | 0.000 | 0.001 |
| MIB2 - MCAR | | | | | | | | | | | | | | | | | | | | | | | | | | | | | | | | | | | | | | | | | | | | | | | | | | | | |
| Estimate | 0.95 | −0.66 | −0.73 | −0.71 | −0.81 | −0.78 | −0.83 | 0.23 | 0.57 | 0.43 | 0.43 | 0.47 | 0.46 | 0.45 | 0.46 | 0.45 | 0.48 | −0.21 | −0.21 | −0.41 | −0.43 | 0.47 | 0.56 | 0.56 | 0.59 |  |  |  |  | 0.49 | 0.01 | 0.05 |  |  |  |  |  |  |  |  | 0.03 | 0.13 | 0.11 | 0.39 | −0.09 | −0.06 | −0.26 | −0.27 |  |  |  |  |
| P-values | 0.000 | 0.000 | 0.000 | 0.000 | 0.000 | 0.000 | 0.000 | 0.069 | 0.000 | 0.000 | 0.000 | 0.000 | 0.000 | 0.000 | 0.000 | 0.000 | 0.000 | 0.078 | 0.079 | 0.001 | 0.000 | 0.000 | 0.000 | 0.000 | 0.000 |  |  |  |  |  | 0.890 | 0.585 |  |  |  |  |  |  |  |  | 0.791 | 0.261 | 0.363 | 0.001 | 0.441 | 0.606 | 0.028 | 0.023 |  |  |  |  |
| MIB2 - MAR | | | | | | | | | | | | | | | | | | | | | | | | | | | | | | | | | | | | | | | | | | | | | | | | | | | | |
| Estimate | 0.64 | −0.55 | −0.83 | −0.82 | −0.97 |  |  | 0.92 | 2.02 | 0.67 | 1.14 | 0.86 | 1.34 |  |  |  |  | −0.44 | −0.51 | −0.47 | −0.95 |  |  |  |  |  |  |  |  | 0.67 | 0.30 | 0.58 | −0.17 | −0.27 | −0.40 | −0.54 |  |  |  |  |  |  |  |  | −0.52 | −0.52 | −1.17 | −1.31 |  |  |  |  |
| P-values | 0.000 | 0.006 | 0.000 | 0.000 | 0.000 |  |  | 0.000 | 0.000 | 0.000 | 0.000 | 0.000 | 0.000 |  |  |  |  | 0.018 | 0.007 | 0.012 | 0.000 |  |  |  |  |  |  |  |  |  | 0.022 | 0.000 | 0.353 | 0.150 | 0.032 | 0.004 |  |  |  |  |  |  |  |  | 0.006 | 0.006 | 0.000 | 0.000 |  |  |  |  |
| MIB2 - MNAR | | | | | | | | | | | | | | | | | | | | | | | | | | | | | | | | | | | | | | | | | | | | | | | | | | | | |
| Estimate | 0.43 | 0.03 | −0.01 | −0.17 | −0.29 | 0.70 | 0.62 | 0.91 | 1.48 | 0.45 | 0.72 | 0.64 | 0.93 | −0.41 | −0.48 | −0.32 | −0.38 | −0.20 | −0.26 | −0.17 | −0.49 | −0.25 | −0.39 | −0.42 | −0.47 |  |  |  |  | 0.60 | 0.02 | 0.55 | −0.35 | −0.40 | −0.88 | −1.17 |  |  |  |  |  |  |  |  | −0.48 | −0.48 | −0.65 | −0.76 | −0.15 | −0.23 | −0.54 | −0.54 |
| P-values | 0.034 | 0.914 | 0.977 | 0.467 | 0.228 | 0.000 | 0.001 | 0.000 | 0.000 | 0.021 | 0.000 | 0.001 | 0.000 | 0.038 | 0.015 | 0.101 | 0.053 | 0.309 | 0.191 | 0.384 | 0.013 | 0.201 | 0.049 | 0.031 | 0.017 |  |  |  |  |  | 0.902 | 0.002 | 0.070 | 0.041 | 0.000 | 0.000 |  |  |  |  |  |  |  |  | 0.015 | 0.016 | 0.001 | 0.000 | 0.450 | 0.233 | 0.006 | 0.006 |
| mice - MCAR | | | | | | | | | | | | | | | | | | | | | | | | | | | | | | | | | | | | | | | | | | | | | | | | | | | | |
| Estimate | 0.46 | −0.47 | −0.62 | −0.42 | −0.42 | 0.34 | 0.32 | 0.47 | 1.67 | 0.49 | 0.56 | 0.53 | 0.63 | −0.26 | −0.23 | −0.24 | −0.21 | −0.31 | −0.29 | −1.08 | −1.08 | −0.17 | −0.27 | −0.22 | −0.27 |  |  |  |  | 0.73 | −0.16 | −0.56 | 0.20 | 0.21 | 0.53 | 0.59 |  |  |  |  | −0.10 | −0.15 | −0.23 | −0.45 | −0.11 | −0.14 | −0.39 | −0.45 | 0.11 | 0.12 | 0.27 | 0.31 |
| P-values | 0.000 | 0.000 | 0.000 | 0.002 | 0.001 | 0.001 | 0.001 | 0.000 | 0.000 | 0.000 | 0.000 | 0.000 | 0.000 | 0.015 | 0.032 | 0.025 | 0.045 | 0.004 | 0.007 | 0.000 | 0.000 | 0.118 | 0.013 | 0.037 | 0.012 |  |  |  |  |  | 0.171 | 0.000 | 0.063 | 0.049 | 0.000 | 0.000 |  |  |  |  | 0.335 | 0.158 | 0.035 | 0.000 | 0.292 | 0.200 | 0.000 | 0.000 | 0.281 | 0.246 | 0.012 | 0.004 |
| mice - MAR | | | | | | | | | | | | | | | | | | | | | | | | | | | | | | | | | | | | | | | | | | | | | | | | | | | | |
| Estimate | 1.64 | −1.70 | −2.05 | −1.68 | −2.04 | 1.14 | 1.12 | 1.82 | 4.77 | 1.91 | 2.24 | 2.32 | 2.65 | −0.76 | −0.74 | −0.79 | −0.74 | −0.86 | −0.96 | −2.27 | −2.62 | −0.73 | −0.74 | −0.84 | −0.73 |  |  |  |  | 0.76 | −0.60 | −1.84 | 0.41 | 0.42 | 1.12 | 1.33 |  |  |  |  | −0.15 | −0.46 | −0.30 | −1.07 | −0.79 | −0.90 | −1.89 | −2.24 | 0.45 | 0.39 | 1.24 | 1.34 |
| P-values | 0.000 | 0.000 | 0.000 | 0.000 | 0.000 | 0.000 | 0.000 | 0.000 | 0.000 | 0.000 | 0.000 | 0.000 | 0.000 | 0.014 | 0.016 | 0.011 | 0.017 | 0.005 | 0.002 | 0.000 | 0.000 | 0.018 | 0.017 | 0.007 | 0.018 |  |  |  |  |  | 0.070 | 0.000 | 0.177 | 0.167 | 0.000 | 0.000 |  |  |  |  | 0.630 | 0.136 | 0.326 | 0.001 | 0.011 | 0.004 | 0.000 | 0.000 | 0.142 | 0.203 | 0.000 | 0.000 |
| mice - MNAR | | | | | | | | | | | | | | | | | | | | | | | | | | | | | | | | | | | | | | | | | | | | | | | | | | | | |
| Estimate | 1.72 | −1.40 | −1.53 | −1.34 | −1.59 | 1.42 | 1.72 | 2.03 | 4.26 | 1.92 | 2.11 | 2.28 | 2.49 | −0.93 | −0.98 | −1.10 | −1.18 | −0.83 | −0.88 | −2.18 | −2.40 | −0.93 | −1.18 | −1.41 | −1.52 | 0.28 | 0.37 | 0.72 | 0.85 | 0.79 | −0.68 | −1.99 | 0.23 | 0.23 | 0.87 | 0.93 |  |  |  |  | −0.28 | −0.84 | −0.46 | −1.30 | −0.83 | −0.93 | −1.56 | −1.85 | 0.36 | 0.33 | 1.05 | 1.15 |
| P-values | 0.000 | 0.000 | 0.000 | 0.000 | 0.000 | 0.000 | 0.000 | 0.000 | 0.000 | 0.000 | 0.000 | 0.000 | 0.000 | 0.002 | 0.001 | 0.000 | 0.000 | 0.005 | 0.003 | 0.000 | 0.000 | 0.002 | 0.000 | 0.000 | 0.000 | 0.341 | 0.214 | 0.015 | 0.004 |  | 0.035 | 0.000 | 0.435 | 0.439 | 0.003 | 0.002 |  |  |  |  | 0.352 | 0.005 | 0.119 | 0.000 | 0.005 | 0.002 | 0.000 | 0.000 | 0.225 | 0.261 | 0.000 | 0.000 |
| mix - MCAR | | | | | | | | | | | | | | | | | | | | | | | | | | | | | | | | | | | | | | | | | | | | | | | | | | | | |
| Estimate | 0.21 | −0.22 | −0.37 | −0.21 | −0.18 | 0.47 | 0.57 | 0.40 | 1.26 | 0.44 | 0.51 | 0.46 | 0.56 | −0.46 | −0.45 | −0.52 | −0.53 | −0.30 | −0.28 | −0.91 | −0.94 | −0.24 | −0.33 | −0.33 | −0.38 | 0.11 | 0.16 | 0.35 | 0.36 | 0.72 | −0.05 | −0.30 | 0.15 | 0.17 | 0.40 | 0.49 |  |  |  |  | −0.12 | −0.17 | −0.21 | −0.38 | −0.12 | −0.14 | −0.36 | −0.41 |  |  |  |  |
| P-values | 0.064 | 0.079 | 0.004 | 0.067 | 0.109 | 0.000 | 0.000 | 0.002 | 0.000 | 0.000 | 0.000 | 0.000 | 0.000 | 0.000 | 0.000 | 0.000 | 0.000 | 0.005 | 0.007 | 0.000 | 0.000 | 0.023 | 0.002 | 0.001 | 0.000 | 0.277 | 0.134 | 0.001 | 0.001 |  | 0.562 | 0.002 | 0.147 | 0.109 | 0.000 | 0.000 |  |  |  |  | 0.240 | 0.109 | 0.047 | 0.000 | 0.266 | 0.167 | 0.001 | 0.000 |  |  |  |  |
| mix - MAR | | | | | | | | | | | | | | | | | | | | | | | | | | | | | | | | | | | | | | | | | | | | | | | | | | | | |
| Estimate | 0.46 | −0.75 | −0.95 | −0.81 | −1.06 | 1.64 | 1.93 | 1.23 | 3.21 | 1.97 | 2.30 | 2.31 | 2.64 | −1.26 | −1.33 | −1.52 | −1.59 | −0.78 | −0.85 | −2.03 | −2.35 | −1.10 | −1.15 | −1.34 | −1.34 | 0.31 | 0.37 | 0.92 | 1.16 | 0.73 | −0.13 | −0.31 |  |  |  |  |  |  |  |  |  |  |  |  | −0.78 | −0.83 | −1.85 | −2.13 |  |  |  |  |
| P-values | 0.126 | 0.023 | 0.004 | 0.014 | 0.001 | 0.000 | 0.000 | 0.000 | 0.000 | 0.000 | 0.000 | 0.000 | 0.000 | 0.000 | 0.000 | 0.000 | 0.000 | 0.011 | 0.006 | 0.000 | 0.000 | 0.000 | 0.000 | 0.000 | 0.000 | 0.310 | 0.228 | 0.003 | 0.000 |  | 0.280 | 0.014 |  |  |  |  |  |  |  |  |  |  |  |  | 0.011 | 0.007 | 0.000 | 0.000 |  |  |  |  |
| mix - MNAR | | | | | | | | | | | | | | | | | | | | | | | | | | | | | | | | | | | | | | | | | | | | | | | | | | | | |
| Estimate | 1.00 | −0.72 | −0.82 | −0.72 | −0.87 | 1.85 | 2.43 | 1.55 | 3.11 | 1.99 | 2.20 | 2.26 | 2.49 | −1.27 | −1.34 | −1.64 | −1.77 | −0.80 | −0.84 | −1.88 | −2.12 | −1.17 | −1.41 | −1.77 | −1.91 | 0.39 | 0.55 | 1.01 | 1.34 | 0.76 | −0.55 | −1.14 |  |  |  |  |  |  |  |  |  |  |  |  | −0.85 | −0.93 | −1.59 | −1.82 |  |  |  |  |
| P-values | 0.001 | 0.033 | 0.015 | 0.034 | 0.010 | 0.000 | 0.000 | 0.000 | 0.000 | 0.000 | 0.000 | 0.000 | 0.000 | 0.000 | 0.000 | 0.000 | 0.000 | 0.011 | 0.007 | 0.000 | 0.000 | 0.000 | 0.000 | 0.000 | 0.000 | 0.208 | 0.081 | 0.001 | 0.000 |  | 0.000 | 0.000 |  |  |  |  |  |  |  |  |  |  |  |  | 0.007 | 0.003 | 0.000 | 0.000 |  |  |  |  |
|  |  |  |  |  |  |  |  |  |  |  |  |  |  |  |  |  |  |  |  |  |  |  |  |  |  |  |  |  |  |  |  |  |  |  |  |  |  |  |  |  |  |  |  |  |  |  |  |  |  |  |  |  |
| --- | --- | --- | --- | --- | --- | --- | --- | --- | --- | --- | --- | --- | --- | --- | --- | --- | --- | --- | --- | --- | --- | --- | --- | --- | --- | --- | --- | --- | --- | --- | --- | --- | --- | --- | --- | --- | --- | --- | --- | --- | --- | --- | --- | --- | --- | --- | --- | --- | --- | --- | --- | --- |
| 1 Ref. (reference group): N (sample size) = 100 2 Ref.: p (prevalence) = 0.1 3 Ref.: AUC\_0 (true AUC) = 0.7 4 Ref.: pm (proportion of missing values) = 0.1 5 Ref.: r (correlation) = 0.2 | | | | | | | | | | | | | | | | | | | | | | | | | | | | | | | | | | | | | | | | | | | | | | | | | | | | |

```
# first approach without interactions; results are not shown

# RMSE
dta_RMSE2 <- c()
for (j in c("MCAR", "MAR", "MNAR")) {
  for (i in 1:length(methods)) {
    
    dta <- mm2("RMSE", methods[i], mechanism=j)
    dta_RMSE2 <- rbind(dta_RMSE2, dta)
    
  }   
}

tabno <- tabno+1
tab_mm_RMSE2 <- dta_RMSE2 %>%
  arrange(Method, Mechanism) %>%
  gt(groupname_col = c( "Method","Mechanism")) %>%
  row_group_order(
    groups = c("CCA - MCAR", "CCA - MAR", "CCA - MNAR",
               "AIPW - MCAR", "AIPW - MAR", "AIPW - MNAR",
               "HDEL - MCAR", "HDEL - MAR", "HDEL - MNAR",
               "KER - MCAR", "KER - MAR", "KER - MNAR",
               "MI2 - MCAR", "MI2 - MAR", "MI2 - MNAR",
               "MIB2 - MCAR", "MIB2 - MAR", "MIB2 - MNAR",
               "mice - MCAR", "mice - MAR", "mice - MNAR",
               "mix - MCAR", "mix - MAR", "mix - MNAR"
               )
  ) %>%
  fmt_number(
    columns = where(is.numeric),
    rows = Result=="Estimate",
    decimals = 2
  ) %>%
  fmt_number(
    columns = where(is.numeric),
    rows = Result=="P-values",
    decimals = 3
  ) %>%
  sub_missing(
  columns = everything(),
  rows = everything(),
  missing_text = ""
  ) %>%
  tab_header(
    title = paste0("Table ", tabno, ". Results for the metamodels with the outcome: RMSE")
  ) %>%
  tab_footnote(
    footnote = "Ref. (reference group): N (sample size) = 100",
    locations = cells_column_labels(columns = c(N500, N1000))
  ) %>%
  tab_footnote(
    footnote = "Ref.: p (prevalence) = 0.1",
    locations = cells_column_labels(columns = c(p0.3, p0.5))
  ) %>%
  tab_footnote(
    footnote = "Ref.: r (correlation) = 0.2",
    locations = cells_column_labels(columns = c(r0.5, r0.9))
  ) %>%
  tab_footnote(
    footnote = "Ref.: AUC_0 (true AUC) = 0.7",
    locations = cells_column_labels(columns = c(AUC_00.85, AUC_00.9))
  ) %>%
  tab_footnote(
    footnote = "Ref.: pm (proportion of missing values) = 0.1",
    locations = cells_column_labels(columns = c(pm0.3, pm0.5))
  ) %>%
  tab_options(footnotes.multiline = FALSE)
  
tab_mm_RMSE2
```

```
# first approach with interactions; results are not shown

dta_RMSE3 <- c()
for (j in c("MCAR", "MAR", "MNAR")) {
  for (i in 1:length(methods)) {
    
    dta <- mm3("RMSE", methods[i], mechanism=j)
    dta_RMSE3 <- rbind(dta_RMSE3, dta)
    
  }   
}

tabno <- tabno+1
tab_mm_RMSE3 <- dta_RMSE3 %>%
  arrange(Method, Mechanism) %>%
  gt(groupname_col = c( "Method","Mechanism")) %>%
  row_group_order(
    groups = c("CCA - MCAR", "CCA - MAR", "CCA - MNAR",
               "AIPW - MCAR", "AIPW - MAR", "AIPW - MNAR",
               "HDEL - MCAR", "HDEL - MAR", "HDEL - MNAR",
               "KER - MCAR", "KER - MAR", "KER - MNAR",
               "MI2 - MCAR", "MI2 - MAR", "MI2 - MNAR",
               "MIB2 - MCAR", "MIB2 - MAR", "MIB2 - MNAR",
               "mice - MCAR", "mice - MAR", "mice - MNAR",
               "mix - MCAR", "mix - MAR", "mix - MNAR"
               )
  ) %>%
  fmt_number(
    columns = where(is.numeric),
    rows = Result=="Estimate",
    decimals = 2
  ) %>%
  fmt_number(
    columns = where(is.numeric),
    rows = Result=="P-values",
    decimals = 3
  ) %>%
  sub_missing(
  columns = everything(),
  rows = everything(),
  missing_text = ""
  ) %>%
  tab_header(
    title = paste0("Table ", tabno, ". Results for the metamodels with all interactions and the outcome: RMSE")
  ) %>%
  tab_footnote(
    footnote = "Ref. (reference group): N (sample size) = 100",
    locations = cells_column_labels(columns = c(N500, N1000))
  ) %>%
  tab_footnote(
    footnote = "Ref.: p (prevalence) = 0.1",
    locations = cells_column_labels(columns = c(p0.3, p0.5))
  ) %>%
  tab_footnote(
    footnote = "Ref.: r (correlation) = 0.2",
    locations = cells_column_labels(columns = c(r0.5, r0.9))
  ) %>%
  tab_footnote(
    footnote = "Ref.: AUC_0 (true AUC) = 0.7",
    locations = cells_column_labels(columns = c(AUC_00.85, AUC_00.9))
  ) %>%
  tab_footnote(
    footnote = "Ref.: pm (proportion of missing values) = 0.1",
    locations = cells_column_labels(columns = c(pm0.3, pm0.5))
  ) %>%
  tab_options(footnotes.multiline = FALSE)
  
tab_mm_RMSE3
```

RMSE: With backward elimination

```
# first approach with interactions and backward selection; results are shown

dta_RMSE4 <- c()
for (j in c("MCAR", "MAR", "MNAR")) {
  for (i in 1:length(methods)) {
    
    dta <- mm4("RMSE", methods[i], mechanism=j)
    dta_RMSE4 <- dta_RMSE4 %>%
        bind_rows(dta)
    
  }   
}

tabno <- tabno+1
tab_mm_RMSE4 <- dta_RMSE4 %>%
  arrange(Method, Mechanism) %>%
  gt(groupname_col = c( "Method","Mechanism")) %>%
  row_group_order(
    groups = c("CCA - MCAR", "CCA - MAR", "CCA - MNAR",
               "AIPW - MCAR", "AIPW - MAR", "AIPW - MNAR",
               "HDEL - MCAR", "HDEL - MAR", "HDEL - MNAR",
               "KER - MCAR", "KER - MAR", "KER - MNAR",
               "MI2 - MCAR", "MI2 - MAR", "MI2 - MNAR",
               "MIB2 - MCAR", "MIB2 - MAR", "MIB2 - MNAR",
               "mice - MCAR", "mice - MAR", "mice - MNAR",
               "mix - MCAR", "mix - MAR", "mix - MNAR"
               )
  ) %>%
  fmt_number(
    columns = where(is.numeric),
    rows = Result=="Estimate",
    decimals = 2
  ) %>%
  fmt_number(
    columns = where(is.numeric),
    rows = Result=="P-values",
    decimals = 3
  ) %>%
  sub_missing(
  columns = everything(),
  rows = everything(),
  missing_text = ""
  ) %>%
  tab_header(
    title = paste0("Table ", tabno, ". Results for the metamodels with all interactions and backward elimination and the outcome: RMSE")
  ) %>%
  tab_footnote(
    footnote = "Ref. (reference group): N (sample size) = 100",
    locations = cells_column_labels(columns = c(N500, N1000))
  ) %>%
  tab_footnote(
    footnote = "Ref.: p (prevalence) = 0.1",
    locations = cells_column_labels(columns = c(p0.3, p0.5))
  ) %>%
  tab_footnote(
    footnote = "Ref.: r (correlation) = 0.2",
    locations = cells_column_labels(columns = c(r0.5, r0.9))
  ) %>%
  tab_footnote(
    footnote = "Ref.: AUC_0 (true AUC) = 0.7",
    locations = cells_column_labels(columns = c(AUC_00.85, AUC_00.9))
  ) %>%
  tab_footnote(
    footnote = "Ref.: pm (proportion of missing values) = 0.1",
    locations = cells_column_labels(columns = c(pm0.3, pm0.5))
  ) %>%
  tab_options(footnotes.multiline = FALSE)
  
tab_mm_RMSE4
```

|  |  |  |  |  |  |  |  |  |  |  |  |  |  |  |  |  |  |  |  |  |  |  |  |  |  |  |  |  |  |  |  |  |  |  |  |  |  |  |  |  |  |  |  |  |  |  |  |  |  |  |  |  |
| --- | --- | --- | --- | --- | --- | --- | --- | --- | --- | --- | --- | --- | --- | --- | --- | --- | --- | --- | --- | --- | --- | --- | --- | --- | --- | --- | --- | --- | --- | --- | --- | --- | --- | --- | --- | --- | --- | --- | --- | --- | --- | --- | --- | --- | --- | --- | --- | --- | --- | --- | --- | --- |
| Table 8. Results for the metamodels with all interactions and backward elimination and the outcome: RMSE | | | | | | | | | | | | | | | | | | | | | | | | | | | | | | | | | | | | | | | | | | | | | | | | | | | | |
| Result | (Intercept) | N5001 | N10001 | p0.32 | p0.52 | AUC\_00.853 | AUC\_00.93 | pm0.34 | pm0.54 | N500:p0.3 | N1000:p0.3 | N500:p0.5 | N1000:p0.5 | N500:AUC\_00.85 | N1000:AUC\_00.85 | N500:AUC\_00.9 | N1000:AUC\_00.9 | N500:pm0.3 | N1000:pm0.3 | N500:pm0.5 | N1000:pm0.5 | p0.3:AUC\_00.85 | p0.5:AUC\_00.85 | p0.3:AUC\_00.9 | p0.5:AUC\_00.9 | p0.3:pm0.3 | p0.5:pm0.3 | p0.3:pm0.5 | p0.5:pm0.5 | AUC\_00.85:pm0.3 | AUC\_00.9:pm0.3 | AUC\_00.85:pm0.5 | AUC\_00.9:pm0.5 | Adjusted R squared | r0.55 | r0.95 | N500:r0.5 | N1000:r0.5 | N500:r0.9 | N1000:r0.9 | r0.5:AUC\_00.85 | r0.9:AUC\_00.85 | r0.5:AUC\_00.9 | r0.9:AUC\_00.9 | r0.5:pm0.3 | r0.9:pm0.3 | r0.5:pm0.5 | r0.9:pm0.5 | p0.3:r0.5 | p0.5:r0.5 | p0.3:r0.9 | p0.5:r0.9 |
| CCA - MCAR | | | | | | | | | | | | | | | | | | | | | | | | | | | | | | | | | | | | | | | | | | | | | | | | | | | | |
| --- | --- | --- | --- | --- | --- | --- | --- | --- | --- | --- | --- | --- | --- | --- | --- | --- | --- | --- | --- | --- | --- | --- | --- | --- | --- | --- | --- | --- | --- | --- | --- | --- | --- | --- | --- | --- | --- | --- | --- | --- | --- | --- | --- | --- | --- | --- | --- | --- | --- | --- | --- | --- |
| Estimate | 9.19 | −5.06 | −6.32 | −3.06 | −3.50 | −2.32 | −3.66 | 1.02 | 2.71 | 1.56 | 1.97 | 1.79 | 2.27 | 1.19 | 1.49 | 1.84 | 2.30 | −0.49 | −0.57 | −1.23 | −1.46 | 0.44 | 0.50 | 0.81 | 0.90 | −0.15 | −0.20 | −0.51 | −0.59 | −0.12 | −0.21 | −0.36 | −0.56 | 1.00 |  |  |  |  |  |  |  |  |  |  |  |  |  |  |  |  |  |  |
| P-values | 0.000 | 0.000 | 0.000 | 0.000 | 0.000 | 0.000 | 0.000 | 0.000 | 0.000 | 0.000 | 0.000 | 0.000 | 0.000 | 0.000 | 0.000 | 0.000 | 0.000 | 0.000 | 0.000 | 0.000 | 0.000 | 0.000 | 0.000 | 0.000 | 0.000 | 0.009 | 0.000 | 0.000 | 0.000 | 0.033 | 0.000 | 0.000 | 0.000 |  |  |  |  |  |  |  |  |  |  |  |  |  |  |  |  |  |  |  |
| CCA - MAR | | | | | | | | | | | | | | | | | | | | | | | | | | | | | | | | | | | | | | | | | | | | | | | | | | | | |
| Estimate | 10.12 | −5.37 | −6.75 | −3.72 | −4.55 | −2.05 | −3.69 | 2.06 | 3.99 | 1.72 | 2.31 | 2.14 | 2.84 | 0.80 | 1.07 | 1.56 | 2.00 | −0.54 | −0.73 | −1.05 | −1.48 | 0.38 | 0.59 | 0.95 | 1.26 | −0.72 | −0.93 | −1.33 | −1.64 | −0.28 | −0.47 | −0.42 | −0.76 | 0.99 | −0.11 | −0.13 | 0.19 | 0.19 | 0.17 | 0.23 | −0.12 | −0.25 | −0.10 | −0.23 | 0.02 | −0.05 | −0.02 | −0.30 | 0.07 | 0.08 | 0.16 | 0.26 |
| P-values | 0.000 | 0.000 | 0.000 | 0.000 | 0.000 | 0.000 | 0.000 | 0.000 | 0.000 | 0.000 | 0.000 | 0.000 | 0.000 | 0.000 | 0.000 | 0.000 | 0.000 | 0.000 | 0.000 | 0.000 | 0.000 | 0.000 | 0.000 | 0.000 | 0.000 | 0.000 | 0.000 | 0.000 | 0.000 | 0.001 | 0.000 | 0.000 | 0.000 |  | 0.305 | 0.203 | 0.028 | 0.028 | 0.041 | 0.006 | 0.164 | 0.003 | 0.240 | 0.006 | 0.854 | 0.560 | 0.834 | 0.001 | 0.375 | 0.338 | 0.054 | 0.002 |
| CCA - MNAR | | | | | | | | | | | | | | | | | | | | | | | | | | | | | | | | | | | | | | | | | | | | | | | | | | | | |
| Estimate | 9.56 | −5.31 | −6.68 | −3.10 | −3.91 | −1.51 | −2.76 | 1.78 | 3.61 | 1.70 | 2.33 | 2.30 | 3.04 | 0.84 | 1.10 | 1.51 | 1.92 | −0.58 | −0.69 | −1.19 | −1.59 | −0.11 | −0.17 | 0.04 | 0.10 | −0.62 | −0.81 | −0.98 | −1.41 | 0.18 | 0.10 | 0.26 | 0.09 | 0.99 |  |  |  |  |  |  |  |  |  |  |  |  |  |  |  |  |  |  |
| P-values | 0.000 | 0.000 | 0.000 | 0.000 | 0.000 | 0.000 | 0.000 | 0.000 | 0.000 | 0.000 | 0.000 | 0.000 | 0.000 | 0.000 | 0.000 | 0.000 | 0.000 | 0.000 | 0.000 | 0.000 | 0.000 | 0.142 | 0.026 | 0.600 | 0.206 | 0.000 | 0.000 | 0.000 | 0.000 | 0.019 | 0.183 | 0.001 | 0.262 |  |  |  |  |  |  |  |  |  |  |  |  |  |  |  |  |  |  |  |
| AIPW - MCAR | | | | | | | | | | | | | | | | | | | | | | | | | | | | | | | | | | | | | | | | | | | | | | | | | | | | |
| Estimate | 9.50 | −5.32 | −6.64 | −3.30 | −3.81 | −2.16 | −3.38 | 1.13 | 2.51 | 1.76 | 2.17 | 2.05 | 2.52 | 0.93 | 1.24 | 1.47 | 1.92 | −0.53 | −0.58 | −1.09 | −1.26 | 0.42 | 0.47 | 0.70 | 0.79 | −0.28 | −0.33 | −0.44 | −0.56 | −0.04 | −0.09 | −0.17 | −0.25 | 0.99 | −0.36 | −1.20 | 0.28 | 0.33 | 0.82 | 0.98 | 0.05 | 0.15 | 0.10 | 0.26 | −0.10 | −0.39 | −0.27 | −0.93 | 0.08 | 0.10 | 0.37 | 0.47 |
| P-values | 0.000 | 0.000 | 0.000 | 0.000 | 0.000 | 0.000 | 0.000 | 0.000 | 0.000 | 0.000 | 0.000 | 0.000 | 0.000 | 0.000 | 0.000 | 0.000 | 0.000 | 0.000 | 0.000 | 0.000 | 0.000 | 0.000 | 0.000 | 0.000 | 0.000 | 0.001 | 0.000 | 0.000 | 0.000 | 0.598 | 0.268 | 0.036 | 0.003 |  | 0.000 | 0.000 | 0.001 | 0.000 | 0.000 | 0.000 | 0.518 | 0.063 | 0.233 | 0.002 | 0.209 | 0.000 | 0.001 | 0.000 | 0.360 | 0.211 | 0.000 | 0.000 |
| AIPW - MAR | | | | | | | | | | | | | | | | | | | | | | | | | | | | | | | | | | | | | | | | | | | | | | | | | | | | |
| Estimate | 11.22 | −6.06 | −7.77 | −4.50 | −5.56 | −2.56 | −3.95 | 2.15 | 5.11 | 2.02 | 2.80 | 2.59 | 3.49 | 1.02 | 1.34 | 1.68 | 2.14 | −0.68 | −0.87 | −1.36 | −1.95 | 0.64 | 0.85 | 0.99 | 1.27 | −0.69 | −0.85 | −1.42 | −1.87 | −0.14 | −0.24 | −0.45 | −0.73 | 0.98 | −0.83 | −2.64 | 0.17 | 0.33 | 1.05 | 1.48 | 0.12 | 0.36 | 0.14 | 0.58 | −0.29 | −0.80 | −0.58 | −1.93 | 0.44 | 0.67 | 1.35 | 1.81 |
| P-values | 0.000 | 0.000 | 0.000 | 0.000 | 0.000 | 0.000 | 0.000 | 0.000 | 0.000 | 0.000 | 0.000 | 0.000 | 0.000 | 0.000 | 0.000 | 0.000 | 0.000 | 0.000 | 0.000 | 0.000 | 0.000 | 0.000 | 0.000 | 0.000 | 0.000 | 0.000 | 0.000 | 0.000 | 0.000 | 0.390 | 0.145 | 0.007 | 0.000 |  | 0.000 | 0.000 | 0.306 | 0.048 | 0.000 | 0.000 | 0.482 | 0.029 | 0.385 | 0.000 | 0.077 | 0.000 | 0.001 | 0.000 | 0.007 | 0.000 | 0.000 | 0.000 |
| AIPW - MNAR | | | | | | | | | | | | | | | | | | | | | | | | | | | | | | | | | | | | | | | | | | | | | | | | | | | | |
| Estimate | 10.24 | −5.90 | −7.30 | −3.80 | −4.77 | −1.33 | −2.47 | 2.14 | 4.22 | 2.08 | 2.74 | 2.83 | 3.59 | 0.62 | 0.84 | 1.36 | 1.64 | −0.77 | −0.88 | −1.40 | −1.87 |  |  |  |  | −0.68 | −0.86 | −0.94 | −1.52 |  |  |  |  | 0.98 | −0.66 | −2.08 | 0.26 | 0.26 | 1.07 | 1.29 |  |  |  |  | −0.32 | −0.83 | −0.48 | −1.75 | 0.35 | 0.45 | 1.02 | 1.41 |
| P-values | 0.000 | 0.000 | 0.000 | 0.000 | 0.000 | 0.000 | 0.000 | 0.000 | 0.000 | 0.000 | 0.000 | 0.000 | 0.000 | 0.000 | 0.000 | 0.000 | 0.000 | 0.000 | 0.000 | 0.000 | 0.000 |  |  |  |  | 0.000 | 0.000 | 0.000 | 0.000 |  |  |  |  |  | 0.000 | 0.000 | 0.068 | 0.078 | 0.000 | 0.000 |  |  |  |  | 0.026 | 0.000 | 0.001 | 0.000 | 0.017 | 0.002 | 0.000 | 0.000 |
| HDEL - MCAR | | | | | | | | | | | | | | | | | | | | | | | | | | | | | | | | | | | | | | | | | | | | | | | | | | | | |
| Estimate | 9.69 | −5.39 | −6.71 | −3.40 | −3.82 | −2.64 | −4.02 | 1.54 | 3.72 | 1.77 | 2.21 | 1.97 | 2.50 | 1.37 | 1.68 | 2.05 | 2.52 | −0.71 | −0.83 | −1.62 | −1.93 | 0.67 | 0.73 | 1.06 | 1.15 | −0.28 | −0.34 | −0.77 | −0.89 | −0.19 | −0.31 | −0.58 | −0.83 | 0.99 |  |  |  |  |  |  |  |  |  |  |  |  |  |  |  |  |  |  |
| P-values | 0.000 | 0.000 | 0.000 | 0.000 | 0.000 | 0.000 | 0.000 | 0.000 | 0.000 | 0.000 | 0.000 | 0.000 | 0.000 | 0.000 | 0.000 | 0.000 | 0.000 | 0.000 | 0.000 | 0.000 | 0.000 | 0.000 | 0.000 | 0.000 | 0.000 | 0.002 | 0.000 | 0.000 | 0.000 | 0.037 | 0.001 | 0.000 | 0.000 |  |  |  |  |  |  |  |  |  |  |  |  |  |  |  |  |  |  |  |
| HDEL - MAR | | | | | | | | | | | | | | | | | | | | | | | | | | | | | | | | | | | | | | | | | | | | | | | | | | | | |
| Estimate | 11.55 | −6.63 | −8.05 | −5.11 | −5.94 | −2.82 | −4.72 | 2.77 | 5.94 | 2.90 | 3.51 | 3.35 | 4.09 | 1.37 | 1.64 | 2.27 | 2.74 | −0.86 | −1.07 | −2.07 | −2.54 | 0.99 | 1.19 | 1.70 | 2.01 | −0.94 | −1.16 | −1.98 | −2.35 | −0.45 | −0.72 | −0.75 | −1.19 | 0.99 | 0.15 | 0.20 |  |  |  |  | −0.28 | −0.34 | −0.20 | −0.30 | 0.07 | −0.02 | −0.02 | −0.42 |  |  |  |  |
| P-values | 0.000 | 0.000 | 0.000 | 0.000 | 0.000 | 0.000 | 0.000 | 0.000 | 0.000 | 0.000 | 0.000 | 0.000 | 0.000 | 0.000 | 0.000 | 0.000 | 0.000 | 0.000 | 0.000 | 0.000 | 0.000 | 0.000 | 0.000 | 0.000 | 0.000 | 0.000 | 0.000 | 0.000 | 0.000 | 0.002 | 0.000 | 0.000 | 0.000 |  | 0.251 | 0.148 |  |  |  |  | 0.056 | 0.024 | 0.169 | 0.040 | 0.650 | 0.891 | 0.902 | 0.004 |  |  |  |  |
| HDEL - MNAR | | | | | | | | | | | | | | | | | | | | | | | | | | | | | | | | | | | | | | | | | | | | | | | | | | | | |
| Estimate | 10.69 | −6.31 | −7.74 | −4.25 | −5.08 | −1.75 | −3.13 | 2.52 | 5.45 | 2.83 | 3.47 | 3.48 | 4.23 | 0.92 | 1.20 | 1.70 | 2.12 | −0.93 | −1.07 | −2.18 | −2.64 | 0.17 | 0.08 | 0.43 | 0.42 | −0.84 | −1.08 | −1.73 | −2.19 | 0.08 | −0.07 | 0.11 | −0.23 | 0.99 |  |  |  |  |  |  |  |  |  |  |  |  |  |  |  |  |  |  |
| P-values | 0.000 | 0.000 | 0.000 | 0.000 | 0.000 | 0.000 | 0.000 | 0.000 | 0.000 | 0.000 | 0.000 | 0.000 | 0.000 | 0.000 | 0.000 | 0.000 | 0.000 | 0.000 | 0.000 | 0.000 | 0.000 | 0.142 | 0.463 | 0.000 | 0.000 | 0.000 | 0.000 | 0.000 | 0.000 | 0.491 | 0.563 | 0.349 | 0.047 |  |  |  |  |  |  |  |  |  |  |  |  |  |  |  |  |  |  |  |
| KER - MCAR | | | | | | | | | | | | | | | | | | | | | | | | | | | | | | | | | | | | | | | | | | | | | | | | | | | | |
| Estimate | 9.48 | −5.38 | −6.69 | −3.53 | −4.00 | −2.41 | −3.69 | 1.07 | 3.15 | 1.98 | 2.42 | 2.26 | 2.76 | 1.16 | 1.47 | 1.74 | 2.20 | −0.51 | −0.56 | −1.45 | −1.65 | 0.69 | 0.77 | 1.08 | 1.21 | −0.25 | −0.31 | −0.78 | −0.91 | −0.09 | −0.15 | −0.36 | −0.50 | 0.99 | −0.11 | −0.37 | 0.11 | 0.15 | 0.32 | 0.43 |  |  |  |  | −0.07 | −0.21 | −0.18 | −0.54 |  |  |  |  |
| P-values | 0.000 | 0.000 | 0.000 | 0.000 | 0.000 | 0.000 | 0.000 | 0.000 | 0.000 | 0.000 | 0.000 | 0.000 | 0.000 | 0.000 | 0.000 | 0.000 | 0.000 | 0.000 | 0.000 | 0.000 | 0.000 | 0.000 | 0.000 | 0.000 | 0.000 | 0.028 | 0.006 | 0.000 | 0.000 | 0.420 | 0.194 | 0.001 | 0.000 |  | 0.300 | 0.000 | 0.340 | 0.191 | 0.005 | 0.000 |  |  |  |  | 0.523 | 0.061 | 0.107 | 0.000 |  |  |  |  |
| KER - MAR | | | | | | | | | | | | | | | | | | | | | | | | | | | | | | | | | | | | | | | | | | | | | | | | | | | | |
| Estimate | 11.82 | −7.46 | −9.03 | −5.83 | −6.74 | −3.00 | −4.66 | 3.08 | 7.90 | 4.15 | 4.96 | 4.72 | 5.65 | 1.38 | 1.75 | 2.18 | 2.75 | −1.20 | −1.41 | −3.02 | −3.68 | 1.08 | 1.39 | 1.73 | 2.12 | −1.19 | −1.36 | −3.05 | −3.50 | −0.35 | −0.52 | −0.82 | −1.24 | 0.97 | −0.08 | −0.19 |  |  |  |  |  |  |  |  | −0.20 | −0.39 | −0.25 | −0.76 |  |  |  |  |
| P-values | 0.000 | 0.000 | 0.000 | 0.000 | 0.000 | 0.000 | 0.000 | 0.000 | 0.000 | 0.000 | 0.000 | 0.000 | 0.000 | 0.000 | 0.000 | 0.000 | 0.000 | 0.000 | 0.000 | 0.000 | 0.000 | 0.000 | 0.000 | 0.000 | 0.000 | 0.000 | 0.000 | 0.000 | 0.000 | 0.150 | 0.036 | 0.001 | 0.000 |  | 0.625 | 0.271 |  |  |  |  |  |  |  |  | 0.404 | 0.108 | 0.300 | 0.002 |  |  |  |  |
| KER - MNAR | | | | | | | | | | | | | | | | | | | | | | | | | | | | | | | | | | | | | | | | | | | | | | | | | | | | |
| Estimate | 10.88 | −6.92 | −8.28 | −4.92 | −5.76 | −1.48 | −3.04 | 2.55 | 6.28 | 4.11 | 4.76 | 4.81 | 5.58 | 0.66 | 0.89 | 1.42 | 1.78 | −1.24 | −1.40 | −3.08 | −3.58 | 0.04 | 0.08 | 0.47 | 0.59 | −1.12 | −1.30 | −2.50 | −3.05 |  |  |  |  | 0.97 | −0.33 | −0.53 |  |  |  |  |  |  |  |  |  |  |  |  |  |  |  |  |
| P-values | 0.000 | 0.000 | 0.000 | 0.000 | 0.000 | 0.000 | 0.000 | 0.000 | 0.000 | 0.000 | 0.000 | 0.000 | 0.000 | 0.007 | 0.000 | 0.000 | 0.000 | 0.000 | 0.000 | 0.000 | 0.000 | 0.868 | 0.730 | 0.054 | 0.015 | 0.000 | 0.000 | 0.000 | 0.000 |  |  |  |  |  | 0.001 | 0.000 |  |  |  |  |  |  |  |  |  |  |  |  |  |  |  |  |
| MI2 - MCAR | | | | | | | | | | | | | | | | | | | | | | | | | | | | | | | | | | | | | | | | | | | | | | | | | | | | |
| Estimate | 9.33 | −5.16 | −6.50 | −3.06 | −3.53 | −2.28 | −3.65 | 1.03 | 2.83 | 1.60 | 2.03 | 1.88 | 2.36 | 1.11 | 1.44 | 1.77 | 2.25 | −0.46 | −0.54 | −1.25 | −1.48 | 0.37 | 0.45 | 0.77 | 0.87 | −0.16 | −0.19 | −0.53 | −0.65 | −0.07 | −0.17 | −0.27 | −0.44 | 0.99 | −0.13 | −0.46 | 0.11 | 0.15 | 0.24 | 0.38 | 0.02 | 0.10 | 0.01 | 0.17 | −0.08 | −0.27 | −0.19 | −0.61 |  |  |  |  |
| P-values | 0.000 | 0.000 | 0.000 | 0.000 | 0.000 | 0.000 | 0.000 | 0.000 | 0.000 | 0.000 | 0.000 | 0.000 | 0.000 | 0.000 | 0.000 | 0.000 | 0.000 | 0.000 | 0.000 | 0.000 | 0.000 | 0.000 | 0.000 | 0.000 | 0.000 | 0.014 | 0.003 | 0.000 | 0.000 | 0.282 | 0.010 | 0.000 | 0.000 |  | 0.066 | 0.000 | 0.087 | 0.025 | 0.000 | 0.000 | 0.797 | 0.133 | 0.846 | 0.009 | 0.195 | 0.000 | 0.004 | 0.000 |  |  |  |  |
| MI2 - MAR | | | | | | | | | | | | | | | | | | | | | | | | | | | | | | | | | | | | | | | | | | | | | | | | | | | | |
| Estimate | 10.02 | −5.56 | −7.14 | −3.81 | −4.59 | −1.58 | −3.31 | 2.30 | 5.22 | 2.50 | 3.29 | 3.01 | 3.95 | 0.37 | 0.74 | 1.18 | 1.73 | −0.88 | −1.10 | −1.86 | −2.49 | −0.01 | 0.14 | 0.44 | 0.70 | −0.88 | −1.06 | −1.91 | −2.37 |  |  |  |  | 0.97 | −0.14 | −0.29 |  |  |  |  |  |  |  |  | −0.18 | −0.49 | −0.22 | −0.88 |  |  |  |  |
| P-values | 0.000 | 0.000 | 0.000 | 0.000 | 0.000 | 0.000 | 0.000 | 0.000 | 0.000 | 0.000 | 0.000 | 0.000 | 0.000 | 0.071 | 0.000 | 0.000 | 0.000 | 0.000 | 0.000 | 0.000 | 0.000 | 0.943 | 0.482 | 0.034 | 0.001 | 0.000 | 0.000 | 0.000 | 0.000 |  |  |  |  |  | 0.338 | 0.049 |  |  |  |  |  |  |  |  | 0.374 | 0.019 | 0.295 | 0.000 |  |  |  |  |
| MI2 - MNAR | | | | | | | | | | | | | | | | | | | | | | | | | | | | | | | | | | | | | | | | | | | | | | | | | | | | |
| Estimate | 9.61 | −5.56 | −6.95 | −3.62 | −4.46 | −1.10 | −2.24 | 2.41 | 4.99 | 2.49 | 3.22 | 3.24 | 4.08 | 0.34 | 0.57 | 1.02 | 1.35 | −0.97 | −1.12 | −1.97 | −2.53 |  |  |  |  | −0.84 | −1.03 | −1.54 | −2.11 |  |  |  |  | 0.97 | −0.16 | −0.36 |  |  |  |  |  |  |  |  | −0.28 | −0.52 | −0.32 | −0.72 |  |  |  |  |
| P-values | 0.000 | 0.000 | 0.000 | 0.000 | 0.000 | 0.000 | 0.000 | 0.000 | 0.000 | 0.000 | 0.000 | 0.000 | 0.000 | 0.106 | 0.007 | 0.000 | 0.000 | 0.000 | 0.000 | 0.000 | 0.000 |  |  |  |  | 0.000 | 0.000 | 0.000 | 0.000 |  |  |  |  |  | 0.292 | 0.018 |  |  |  |  |  |  |  |  | 0.193 | 0.014 | 0.135 | 0.001 |  |  |  |  |
| MIB2 - MCAR | | | | | | | | | | | | | | | | | | | | | | | | | | | | | | | | | | | | | | | | | | | | | | | | | | | | |
| Estimate | 8.79 | −4.49 | −5.90 | −2.53 | −3.01 | −1.69 | −3.00 | 0.86 | 2.35 | 1.15 | 1.61 | 1.43 | 1.95 | 0.60 | 0.97 | 1.24 | 1.77 | −0.40 | −0.47 | −1.01 | −1.22 | 0.12 | 0.16 | 0.43 | 0.53 | −0.13 | −0.16 | −0.40 | −0.50 | 0.00 | −0.09 | −0.15 | −0.28 | 0.99 | −0.09 | −0.25 | 0.08 | 0.11 | 0.09 | 0.22 |  |  |  |  | −0.08 | −0.18 | −0.17 | −0.50 |  |  |  |  |
| P-values | 0.000 | 0.000 | 0.000 | 0.000 | 0.000 | 0.000 | 0.000 | 0.000 | 0.000 | 0.000 | 0.000 | 0.000 | 0.000 | 0.000 | 0.000 | 0.000 | 0.000 | 0.000 | 0.000 | 0.000 | 0.000 | 0.091 | 0.024 | 0.000 | 0.000 | 0.071 | 0.021 | 0.000 | 0.000 | 0.968 | 0.195 | 0.036 | 0.000 |  | 0.169 | 0.000 | 0.226 | 0.121 | 0.203 | 0.002 |  |  |  |  | 0.266 | 0.010 | 0.018 | 0.000 |  |  |  |  |
| MIB2 - MAR | | | | | | | | | | | | | | | | | | | | | | | | | | | | | | | | | | | | | | | | | | | | | | | | | | | | |
| Estimate | 8.93 | −4.42 | −5.93 | −2.94 | −3.50 | −0.88 | −2.09 | 1.74 | 4.16 | 1.53 | 2.28 | 1.94 | 2.83 | −0.18 | 0.07 | 0.41 | 0.86 | −0.56 | −0.75 | −1.16 | −1.73 |  |  |  |  | −0.62 | −0.80 | −1.57 | −1.92 |  |  |  |  | 0.97 | −0.12 | −0.25 |  |  |  |  |  |  |  |  | −0.13 | −0.39 | −0.21 | −0.71 |  |  |  |  |
| P-values | 0.000 | 0.000 | 0.000 | 0.000 | 0.000 | 0.000 | 0.000 | 0.000 | 0.000 | 0.000 | 0.000 | 0.000 | 0.000 | 0.337 | 0.717 | 0.027 | 0.000 | 0.003 | 0.000 | 0.000 | 0.000 |  |  |  |  | 0.001 | 0.000 | 0.000 | 0.000 |  |  |  |  |  | 0.344 | 0.056 |  |  |  |  |  |  |  |  | 0.495 | 0.035 | 0.262 | 0.000 |  |  |  |  |
| MIB2 - MNAR | | | | | | | | | | | | | | | | | | | | | | | | | | | | | | | | | | | | | | | | | | | | | | | | | | | | |
| Estimate | 8.52 | −4.18 | −5.63 | −2.47 | −3.14 | −0.42 | −1.44 | 1.71 | 3.55 | 1.36 | 2.16 | 2.03 | 2.95 | −0.17 | 0.01 | 0.36 | 0.65 | −0.64 | −0.78 | −1.20 | −1.79 | −0.56 | −0.76 | −0.58 | −0.70 | −0.59 | −0.79 | −1.20 | −1.71 | 0.29 | 0.25 | 0.62 | 0.60 | 0.96 | −0.02 | 0.01 | −0.19 | −0.20 | −0.43 | −0.50 |  |  |  |  | −0.24 | −0.47 | −0.24 | −0.57 |  |  |  |  |
| P-values | 0.000 | 0.000 | 0.000 | 0.000 | 0.000 | 0.049 | 0.000 | 0.000 | 0.000 | 0.000 | 0.000 | 0.000 | 0.000 | 0.388 | 0.953 | 0.072 | 0.001 | 0.002 | 0.000 | 0.000 | 0.000 | 0.005 | 0.000 | 0.004 | 0.000 | 0.003 | 0.000 | 0.000 | 0.000 | 0.149 | 0.207 | 0.002 | 0.003 |  | 0.909 | 0.942 | 0.351 | 0.319 | 0.031 | 0.013 |  |  |  |  | 0.231 | 0.018 | 0.224 | 0.005 |  |  |  |  |
| mice - MCAR | | | | | | | | | | | | | | | | | | | | | | | | | | | | | | | | | | | | | | | | | | | | | | | | | | | | |
| Estimate | 8.80 | −4.71 | −5.99 | −2.75 | −3.20 | −1.69 | −2.82 | 0.72 | 2.04 | 1.31 | 1.69 | 1.54 | 1.98 | 0.64 | 0.92 | 1.11 | 1.53 | −0.31 | −0.35 | −0.87 | −1.02 | 0.21 | 0.24 | 0.46 | 0.53 | −0.06 | −0.09 | −0.24 | −0.31 |  |  |  |  | 0.99 | −0.36 | −0.88 | 0.31 | 0.37 | 0.69 | 0.85 |  |  |  |  | −0.16 | −0.38 | −0.40 | −0.97 | 0.11 | 0.16 | 0.26 | 0.34 |
| P-values | 0.000 | 0.000 | 0.000 | 0.000 | 0.000 | 0.000 | 0.000 | 0.000 | 0.000 | 0.000 | 0.000 | 0.000 | 0.000 | 0.000 | 0.000 | 0.000 | 0.000 | 0.002 | 0.001 | 0.000 | 0.000 | 0.035 | 0.016 | 0.000 | 0.000 | 0.522 | 0.393 | 0.018 | 0.002 |  |  |  |  |  | 0.001 | 0.000 | 0.002 | 0.000 | 0.000 | 0.000 |  |  |  |  | 0.100 | 0.000 | 0.000 | 0.000 | 0.282 | 0.119 | 0.009 | 0.001 |
| mice - MAR | | | | | | | | | | | | | | | | | | | | | | | | | | | | | | | | | | | | | | | | | | | | | | | | | | | | |
| Estimate | 9.24 | −4.80 | −6.01 | −3.59 | −4.28 | −0.79 | −1.40 | 1.68 | 4.15 | 1.75 | 2.32 | 2.15 | 2.83 |  |  |  |  | −0.45 | −0.61 | −1.16 | −1.55 |  |  |  |  | −0.51 | −0.66 | −1.27 | −1.63 |  |  |  |  | 0.92 | −0.89 | −2.02 | 0.38 | 0.44 | 0.96 | 1.24 |  |  |  |  | −0.35 | −0.83 | −0.67 | −1.87 | 0.62 | 0.73 | 1.30 | 1.61 |
| P-values | 0.000 | 0.000 | 0.000 | 0.000 | 0.000 | 0.000 | 0.000 | 0.000 | 0.000 | 0.000 | 0.000 | 0.000 | 0.000 |  |  |  |  | 0.107 | 0.030 | 0.000 | 0.000 |  |  |  |  | 0.073 | 0.020 | 0.000 | 0.000 |  |  |  |  |  | 0.004 | 0.000 | 0.174 | 0.120 | 0.001 | 0.000 |  |  |  |  | 0.215 | 0.004 | 0.019 | 0.000 | 0.028 | 0.010 | 0.000 | 0.000 |
| mice - MNAR | | | | | | | | | | | | | | | | | | | | | | | | | | | | | | | | | | | | | | | | | | | | | | | | | | | | |
| Estimate | 9.04 | −4.98 | −6.13 | −3.17 | −3.92 | −0.28 | −0.73 | 1.75 | 3.68 | 1.88 | 2.40 | 2.41 | 3.04 |  |  |  |  | −0.59 | −0.71 | −1.36 | −1.71 | −0.52 | −0.65 | −0.73 | −0.79 | −0.56 | −0.73 | −1.07 | −1.47 | 0.33 | 0.41 | 0.87 | 1.03 | 0.94 | −0.73 | −1.71 | 0.38 | 0.43 | 1.11 | 1.32 | −0.22 | −0.61 | −0.17 | −0.68 | −0.43 | −0.99 | −0.68 | −1.97 | 0.50 | 0.61 | 1.18 | 1.49 |
| P-values | 0.000 | 0.000 | 0.000 | 0.000 | 0.000 | 0.322 | 0.010 | 0.000 | 0.000 | 0.000 | 0.000 | 0.000 | 0.000 |  |  |  |  | 0.023 | 0.007 | 0.000 | 0.000 | 0.048 | 0.013 | 0.006 | 0.003 | 0.033 | 0.005 | 0.000 | 0.000 | 0.204 | 0.114 | 0.001 | 0.000 |  | 0.024 | 0.000 | 0.147 | 0.099 | 0.000 | 0.000 | 0.392 | 0.019 | 0.515 | 0.010 | 0.101 | 0.000 | 0.009 | 0.000 | 0.055 | 0.020 | 0.000 | 0.000 |
| mix - MCAR | | | | | | | | | | | | | | | | | | | | | | | | | | | | | | | | | | | | | | | | | | | | | | | | | | | | |
| Estimate | 8.53 | −4.56 | −5.83 | −2.66 | −3.06 | −1.63 | −2.66 | 0.64 | 1.81 | 1.43 | 1.80 | 1.64 | 2.07 | 0.58 | 0.88 | 1.00 | 1.43 | −0.30 | −0.33 | −0.83 | −0.95 | 0.18 | 0.22 | 0.37 | 0.45 | −0.07 | −0.09 | −0.30 | −0.36 |  |  |  |  | 0.99 | −0.14 | −0.44 | 0.16 | 0.20 | 0.39 | 0.53 |  |  |  |  | −0.11 | −0.28 | −0.21 | −0.65 |  |  |  |  |
| P-values | 0.000 | 0.000 | 0.000 | 0.000 | 0.000 | 0.000 | 0.000 | 0.000 | 0.000 | 0.000 | 0.000 | 0.000 | 0.000 | 0.000 | 0.000 | 0.000 | 0.000 | 0.001 | 0.000 | 0.000 | 0.000 | 0.046 | 0.015 | 0.000 | 0.000 | 0.455 | 0.324 | 0.001 | 0.000 |  |  |  |  |  | 0.083 | 0.000 | 0.082 | 0.026 | 0.000 | 0.000 |  |  |  |  | 0.240 | 0.003 | 0.020 | 0.000 |  |  |  |  |
| mix - MAR | | | | | | | | | | | | | | | | | | | | | | | | | | | | | | | | | | | | | | | | | | | | | | | | | | | | |
| Estimate | 8.49 | −4.55 | −5.74 | −2.64 | −3.26 | −0.58 | −1.09 | 1.28 | 2.86 | 2.00 | 2.73 | 2.48 | 3.31 |  |  |  |  | −0.60 | −0.75 | −1.21 | −1.73 | −0.70 | −0.65 | −0.81 | −0.77 | −0.68 | −0.79 | −1.40 | −1.81 | 0.22 | 0.32 | 0.74 | 1.06 | 0.92 | −0.25 | −0.58 |  |  |  |  |  |  |  |  |  |  |  |  |  |  |  |  |
| P-values | 0.000 | 0.000 | 0.000 | 0.000 | 0.000 | 0.033 | 0.000 | 0.000 | 0.000 | 0.000 | 0.000 | 0.000 | 0.000 |  |  |  |  | 0.043 | 0.012 | 0.000 | 0.000 | 0.019 | 0.028 | 0.006 | 0.010 | 0.022 | 0.008 | 0.000 | 0.000 | 0.454 | 0.284 | 0.012 | 0.000 |  | 0.043 | 0.000 |  |  |  |  |  |  |  |  |  |  |  |  |  |  |  |  |
| mix - MNAR | | | | | | | | | | | | | | | | | | | | | | | | | | | | | | | | | | | | | | | | | | | | | | | | | | | | |
| Estimate | 8.42 | −4.67 | −5.83 | −2.66 | −3.29 | −0.44 | −0.74 | 1.72 | 3.20 | 2.14 | 2.82 | 2.72 | 3.50 |  |  |  |  | −0.72 | −0.81 | −1.30 | −1.80 | −0.70 | −0.80 | −1.11 | −1.20 | −0.72 | −0.86 | −1.25 | −1.69 | 0.38 | 0.55 | 0.98 | 1.40 | 0.93 | −0.17 | −0.34 |  |  |  |  |  |  |  |  | −0.31 | −0.62 | −0.44 | −0.86 |  |  |  |  |
| P-values | 0.000 | 0.000 | 0.000 | 0.000 | 0.000 | 0.072 | 0.003 | 0.000 | 0.000 | 0.000 | 0.000 | 0.000 | 0.000 |  |  |  |  | 0.008 | 0.003 | 0.000 | 0.000 | 0.010 | 0.003 | 0.000 | 0.000 | 0.009 | 0.002 | 0.000 | 0.000 | 0.164 | 0.043 | 0.000 | 0.000 |  | 0.368 | 0.073 |  |  |  |  |  |  |  |  | 0.250 | 0.023 | 0.101 | 0.002 |  |  |  |  |
|  |  |  |  |  |  |  |  |  |  |  |  |  |  |  |  |  |  |  |  |  |  |  |  |  |  |  |  |  |  |  |  |  |  |  |  |  |  |  |  |  |  |  |  |  |  |  |  |  |  |  |  |  |
| --- | --- | --- | --- | --- | --- | --- | --- | --- | --- | --- | --- | --- | --- | --- | --- | --- | --- | --- | --- | --- | --- | --- | --- | --- | --- | --- | --- | --- | --- | --- | --- | --- | --- | --- | --- | --- | --- | --- | --- | --- | --- | --- | --- | --- | --- | --- | --- | --- | --- | --- | --- | --- |
| 1 Ref. (reference group): N (sample size) = 100 2 Ref.: p (prevalence) = 0.1 3 Ref.: AUC\_0 (true AUC) = 0.7 4 Ref.: pm (proportion of missing values) = 0.1 5 Ref.: r (correlation) = 0.2 | | | | | | | | | | | | | | | | | | | | | | | | | | | | | | | | | | | | | | | | | | | | | | | | | | | | |

### 4.5.2 Stratified by missingness machanism but all methods in one model

In the second approach, linear regression models were conducted
stratified by missingness mechanism, but the methods were included as
covariates in one model. The other simulation factors were also included
as covariates in the regression models. Bias and RMSE were examined as
outcome variables in separate models. The scenario number was included
as random intercept.

```
# function for 2nd approach without interactions
mm5 <- function(performance, mechanism) {
  
  data <- res_long %>%
    filter(mech == mechanism) %>%
    mutate(
      Method2 = as.factor(Method),
      Method2 = relevel(Method2, ref="CCA"),
      Bias = abs(Bias)
    )
  form <- paste0(performance, "*100", " ~ Method2 + (1|scenario)")
  mm <- lmer(as.formula(form), data=data)
  summary(mm)
  
  #r2 <- summary(mm)$adj.r.squared
  #names(r2) <- "Adjusted R squared"

  dta <- cbind(coef(summary(mm))[,"Estimate"], coef(summary(mm))[,"Pr(>|t|)"])
  colnames(dta) <- c("Estimate", "p-value")
  dta <- as.data.frame(t(dta))
 
  dta <- dta %>%
    tibble::rownames_to_column("Result") %>%
    mutate(
      Mechanism = mechanism
    ) %>%
    relocate(any_of(c("Mechanism", "Result")), .before="(Intercept)") %>%
    rename(
      AIPW = Method2AIPW,
      HDEL = Method2HDEL,
      KER = Method2KER,
      MI2 = Method2MI2,
      MIB2 = Method2MIB2,
      mice = Method2mice,
      mix = Method2mix
    )
  
  return(dta)
}

# function for 2nd approach with interactions
mm6 <- function(performance, mechanism) {
  
  data <- res_long %>%
    filter(mech == mechanism) %>%
    mutate(
      Method2 = as.factor(Method),
      Method2 = relevel(Method2, ref="CCA"),
      Bias = abs(Bias)
      #,across(c(N,p,r,pm,AUC_0), as.factor)
    )
  form <- paste0(performance, "*100", " ~ Method2*N + Method2*p + Method2*r + Method2*AUC_0 + Method2*pm + (1|scenario)")
  mm <- lmer(as.formula(form), data=data)

  dta <- cbind(coef(summary(mm))[,"Estimate"],
               coef(summary(mm))[,"Pr(>|t|)"])
  colnames(dta) <- c("Estimate", "p-value")
  dta <- as.data.frame(t(dta))
  dta <- dta %>%
    tibble::rownames_to_column("Result") %>%
    mutate(
      Mechanism = mechanism
    ) %>%
    relocate(any_of(c("Mechanism", "Result")), .before="(Intercept)") %>%
    rename(
      AIPW = Method2AIPW,
      HDEL = Method2HDEL,
      KER = Method2KER,
      MI2 = Method2MI2,
      MIB2 = Method2MIB2,
      mice = Method2mice,
      mix = Method2mix
    )
  
  return((dta))
}
```

```
# 2nd approach without interactions; results are not shown
# bias
dta_bias5 <- c()
for (j in c("MCAR", "MAR", "MNAR")) {
    
      dta <- mm5("Bias",  mechanism=j)
      dta_bias5 <- rbind(dta_bias5, dta)
      
}

tabno <- tabno+1
tab_mm_bias5 <- dta_bias5 %>%
  arrange(Mechanism) %>%
  gt(groupname_col = c("Mechanism")) %>%
  row_group_order(groups=c("MCAR", "MAR", "MNAR")) %>%
  fmt_number(
    columns = where(is.numeric),
    rows = Result=="Estimate",
    decimals = 2
  ) %>%
  fmt_number(
    columns = where(is.numeric),
    rows = Result=="p-value",
    decimals = 3
  ) %>%
  sub_missing(
  columns = everything(),
  rows = everything(),
  missing_text = ""
  ) %>%
  tab_header(
    title = paste0("Table ", tabno, ". Differences in bias by method stratified by missingness mechanism")
  ) %>%
  tab_footnote(
    footnote = "CCA as refernce group",
    locations = cells_column_labels(columns = c(AIPW,HDEL,KER,MI2,MIB2,mice,mix))
  )
tab_mm_bias5
```

```
# 2nd approach with interactions; results are not shown
# bias
dta_bias6 <- c()
for (j in c("MCAR", "MAR", "MNAR")) {
    
      dta <- mm6("Bias",  mechanism=j)
      dta_bias6 <- rbind(dta_bias6, dta)
      
}

tabno <- tabno+1
tab_mm_bias6 <- dta_bias6 %>%
  arrange(Mechanism) %>%
  gt(groupname_col = c("Mechanism")) %>%
  row_group_order(groups=c("MCAR", "MAR", "MNAR")) %>%
  fmt_number(
    columns = where(is.numeric),
    rows = Result=="Estimate",
    decimals = 2
  ) %>%
  fmt_number(
    columns = where(is.numeric),
    rows = Result=="p-value",
    decimals = 3
  ) %>%
  sub_missing(
  columns = everything(),
  rows = everything(),
  missing_text = ""
  ) %>%
  tab_header(
    title = paste0("Table ", tabno, ". Differences in bias by method and simulation parameters stratified by missingness mechanism")
  ) %>%
  tab_footnote(
    footnote = "CCA as refernce group",
    locations = cells_column_labels(columns = c(AIPW,HDEL,KER,MI2,MIB2,mice,mix))
  )
tab_mm_bias6
```

Bias: Backward elimination

```
# 2nd approach with interactions and backward selection; results are  shown
# bias
data1 <- res_long %>%
          mutate(
              Method2 = as.factor(Method),
              Method2 = relevel(Method2, ref="CCA"),
               Bias = abs(Bias)
           )
 
dta_bias7 <- c()
for (j in c("MCAR", "MAR", "MNAR")) {
    
              form <- paste0("Bias", "*100", " ~ Method2*N + Method2*p + Method2*r + Method2*AUC_0 + Method2*pm + (1|scenario)")
              mm <- lmer(as.formula(form), data=data1[which(data1$mech==j),])
              backward <- lmerTest::step(mm)
              final <- lmerTest::get_model(backward)
            
              dta <- cbind(coef(summary(final))[,"Estimate"],
                           coef(summary(final))[,"Pr(>|t|)"])
              colnames(dta) <- c("Estimate", "p-value")
              dta <- as.data.frame(t(dta))
              dta <- dta %>%
                tibble::rownames_to_column("Result") %>%
                mutate(
                  Mechanism = j
                ) %>%
                relocate(any_of(c("Mechanism", "Result")), .before="(Intercept)") %>%
                rename(
                  AIPW = Method2AIPW,
                  HDEL = Method2HDEL,
                  KER = Method2KER,
                  MI2 = Method2MI2,
                  MIB2 = Method2MIB2,
                  mice = Method2mice,
                  mix = Method2mix
                )
              
      dta_bias7 <- dta_bias7 %>%
        bind_rows(dta)
      
}

tabno <- tabno+1
tab_mm_bias7 <- dta_bias7 %>%
  arrange(Mechanism) %>%
  gt(groupname_col = c("Mechanism")) %>%
  row_group_order(groups=c("MCAR", "MAR", "MNAR")) %>%
  fmt_number(
    columns = where(is.numeric),
    rows = Result=="Estimate",
    decimals = 2
  ) %>%
  fmt_number(
    columns = where(is.numeric),
    rows = Result=="p-value",
    decimals = 3
  ) %>%
  sub_missing(
  columns = everything(),
  rows = everything(),
  missing_text = ""
  )  %>%
  tab_footnote(
    footnote = "CCA as refernce group",
    locations = cells_column_labels(columns = c(AIPW,HDEL,KER,MI2,MIB2,mice,mix))
  ) %>%
  tab_header(
    title = paste0("Table ", tabno, ". Differences in bias by method and simulation parameters stratified by missingness mechanism with backward elimination")
  )
tab_mm_bias7
```

|  |  |  |  |  |  |  |  |  |  |  |  |  |  |  |  |  |  |  |  |  |  |  |  |  |  |  |  |  |  |  |  |  |  |  |  |  |  |  |  |  |  |  |  |  |  |  |  |  |
| --- | --- | --- | --- | --- | --- | --- | --- | --- | --- | --- | --- | --- | --- | --- | --- | --- | --- | --- | --- | --- | --- | --- | --- | --- | --- | --- | --- | --- | --- | --- | --- | --- | --- | --- | --- | --- | --- | --- | --- | --- | --- | --- | --- | --- | --- | --- | --- | --- |
| Table 9. Differences in bias by method and simulation parameters stratified by missingness mechanism with backward elimination | | | | | | | | | | | | | | | | | | | | | | | | | | | | | | | | | | | | | | | | | | | | | | | | |
| Result | (Intercept) | AIPW1 | HDEL1 | KER1 | MI21 | MIB21 | mice1 | mix1 | N | p | r | AUC\_0 | pm | Method2AIPW:N | Method2HDEL:N | Method2KER:N | Method2MI2:N | Method2MIB2:N | Method2mice:N | Method2mix:N | Method2AIPW:p | Method2HDEL:p | Method2KER:p | Method2MI2:p | Method2MIB2:p | Method2mice:p | Method2mix:p | Method2AIPW:r | Method2HDEL:r | Method2KER:r | Method2MI2:r | Method2MIB2:r | Method2mice:r | Method2mix:r | Method2AIPW:AUC\_0 | Method2HDEL:AUC\_0 | Method2KER:AUC\_0 | Method2MI2:AUC\_0 | Method2MIB2:AUC\_0 | Method2mice:AUC\_0 | Method2mix:AUC\_0 | Method2AIPW:pm | Method2HDEL:pm | Method2KER:pm | Method2MI2:pm | Method2MIB2:pm | Method2mice:pm | Method2mix:pm |
| MCAR | | | | | | | | | | | | | | | | | | | | | | | | | | | | | | | | | | | | | | | | | | | | | | | | |
| --- | --- | --- | --- | --- | --- | --- | --- | --- | --- | --- | --- | --- | --- | --- | --- | --- | --- | --- | --- | --- | --- | --- | --- | --- | --- | --- | --- | --- | --- | --- | --- | --- | --- | --- | --- | --- | --- | --- | --- | --- | --- | --- | --- | --- | --- | --- | --- | --- |
| Estimate | 0.52 | −0.12 | −0.06 | −0.84 | −0.29 | 0.29 | 0.05 | −0.54 | 0.00 | −0.16 | 0.02 | −0.44 | 0.10 | 0.00 | 0.00 | 0.00 | 0.00 | 0.00 | 0.00 | 0.00 | −0.32 | 0.03 | 0.34 | −0.15 | −0.38 | −0.50 | −0.49 | −0.13 | −0.01 | −0.04 | 0.32 | 0.31 | −0.30 | −0.29 | 0.42 | 0.08 | 1.44 | 0.24 | −0.30 | 0.50 | 1.21 | 0.23 | −0.03 | −0.07 | 0.49 | 0.58 | 0.99 | 0.96 |
| p-value | 0.005 | 0.618 | 0.791 | 0.000 | 0.225 | 0.218 | 0.842 | 0.022 | 0.020 | 0.141 | 0.809 | 0.042 | 0.354 | 0.014 | 0.924 | 0.000 | 0.012 | 0.000 | 0.000 | 0.000 | 0.021 | 0.833 | 0.015 | 0.300 | 0.006 | 0.000 | 0.000 | 0.103 | 0.916 | 0.605 | 0.000 | 0.000 | 0.000 | 0.000 | 0.118 | 0.757 | 0.000 | 0.368 | 0.265 | 0.062 | 0.000 | 0.104 | 0.842 | 0.629 | 0.000 | 0.000 | 0.000 | 0.000 |
| MAR | | | | | | | | | | | | | | | | | | | | | | | | | | | | | | | | | | | | | | | | | | | | | | | | |
| Estimate | 4.46 | −3.03 | 0.48 | −2.15 | −3.14 | −3.29 | −2.76 | −4.88 | 0.00 | −1.58 | 0.17 | −4.48 | 0.87 | 0.00 | 0.00 | 0.00 | 0.00 | 0.00 | 0.00 | 0.00 | −0.32 | −0.40 | −1.04 | −0.32 | −0.53 | −1.77 | −1.48 | −0.43 | 0.01 | 0.27 | 0.31 | 0.20 | −1.13 | −0.61 | 4.12 | −0.39 | 3.74 | 3.80 | 4.06 | 5.06 | 7.13 | 0.39 | 0.30 | 0.63 | 0.76 | 0.92 | 2.39 | 1.92 |
| p-value | 0.000 | 0.000 | 0.451 | 0.001 | 0.000 | 0.000 | 0.000 | 0.000 | 0.372 | 0.000 | 0.397 | 0.000 | 0.012 | 0.000 | 0.315 | 0.000 | 0.000 | 0.000 | 0.000 | 0.000 | 0.401 | 0.291 | 0.006 | 0.394 | 0.161 | 0.000 | 0.000 | 0.047 | 0.963 | 0.209 | 0.158 | 0.353 | 0.000 | 0.005 | 0.000 | 0.595 | 0.000 | 0.000 | 0.000 | 0.000 | 0.000 | 0.300 | 0.436 | 0.098 | 0.047 | 0.016 | 0.000 | 0.000 |
| MNAR | | | | | | | | | | | | | | | | | | | | | | | | | | | | | | | | | | | | | | | | | | | | | | | | |
| Estimate | 0.38 | 0.83 | 0.21 | 1.46 | 0.75 | 0.52 | 0.79 | −0.61 | 0.00 | −0.93 | 0.03 | 0.53 | 2.20 | 0.00 | 0.00 | 0.00 | 0.00 | 0.00 | 0.00 | 0.00 | −1.22 | −0.33 | −1.19 | −0.79 | −0.88 | −2.41 | −2.36 | −1.53 | 0.00 | −0.49 | −0.73 | −0.71 | −2.00 | −1.65 | 0.50 | −0.10 | −0.24 | −0.16 | 0.12 | 1.61 | 3.04 | −0.09 | 0.20 | −0.19 | −0.33 | −0.23 | 1.64 | 1.36 |
| p-value | 0.487 | 0.147 | 0.708 | 0.010 | 0.189 | 0.363 | 0.167 | 0.287 | 0.740 | 0.004 | 0.884 | 0.396 | 0.000 | 0.000 | 0.453 | 0.000 | 0.000 | 0.000 | 0.000 | 0.000 | 0.000 | 0.333 | 0.000 | 0.020 | 0.010 | 0.000 | 0.000 | 0.000 | 0.981 | 0.012 | 0.000 | 0.000 | 0.000 | 0.000 | 0.448 | 0.879 | 0.708 | 0.802 | 0.852 | 0.014 | 0.000 | 0.801 | 0.547 | 0.577 | 0.327 | 0.500 | 0.000 | 0.000 |
|  |  |  |  |  |  |  |  |  |  |  |  |  |  |  |  |  |  |  |  |  |  |  |  |  |  |  |  |  |  |  |  |  |  |  |  |  |  |  |  |  |  |  |  |  |  |  |  |  |
| --- | --- | --- | --- | --- | --- | --- | --- | --- | --- | --- | --- | --- | --- | --- | --- | --- | --- | --- | --- | --- | --- | --- | --- | --- | --- | --- | --- | --- | --- | --- | --- | --- | --- | --- | --- | --- | --- | --- | --- | --- | --- | --- | --- | --- | --- | --- | --- | --- |
| 1 CCA as refernce group | | | | | | | | | | | | | | | | | | | | | | | | | | | | | | | | | | | | | | | | | | | | | | | | |

```
# 2nd approach without interactions; results are not shown
# RMSE

dta_rmse5 <- c()
for (j in c("MCAR", "MAR", "MNAR")) {
    
      dta <- mm5("RMSE",  mechanism=j)
      dta_rmse5 <- rbind(dta_rmse5, dta)
      
}

tabno <- tabno+1
tab_mm_rmse5 <- dta_rmse5 %>%
  arrange(Mechanism) %>%
  gt(groupname_col = c("Mechanism")) %>%
  row_group_order(groups=c("MCAR", "MAR", "MNAR")) %>%
  fmt_number(
    columns = where(is.numeric),
    rows = Result=="Estimate",
    decimals = 2
  ) %>%
  fmt_number(
    columns = where(is.numeric),
    rows = Result=="p-value",
    decimals = 3
  ) %>%
  sub_missing(
  columns = everything(),
  rows = everything(),
  missing_text = ""
  ) %>%
  tab_header(
    title = paste0("Table ", tabno, ". Differences in RMSE by method stratified by missingness mechanism")
  ) %>%
  tab_footnote(
    footnote = "CCA as refernce group",
    locations = cells_column_labels(columns = c(AIPW,HDEL,KER,MI2,MIB2,mice,mix))
  )
tab_mm_rmse5
```

```
# 2nd approach with interactions; results are not shown
# RMSE
dta_rmse6 <- c()
for (j in c("MCAR", "MAR", "MNAR")) {
    
      dta <- mm6("RMSE",  mechanism=j)
      dta_rmse6 <- rbind(dta_rmse6, dta)
      
}

tabno <- tabno+1
tab_mm_rmse6 <- dta_rmse6 %>%
  arrange(Mechanism) %>%
  gt(groupname_col = c("Mechanism")) %>%
  row_group_order(groups=c("MCAR", "MAR", "MNAR")) %>%
  fmt_number(
    columns = where(is.numeric),
    rows = Result=="Estimate",
    decimals = 2
  ) %>%
  fmt_number(
    columns = where(is.numeric),
    rows = Result=="p-value",
    decimals = 3
  ) %>%
  sub_missing(
  columns = everything(),
  rows = everything(),
  missing_text = ""
  ) %>%
  tab_header(
    title = paste0("Table ", tabno, ". Differences in RMSE by method and simulation parameter stratified by missingness mechanism")
  ) %>%
  tab_footnote(
    footnote = "CCA as refernce group",
    locations = cells_column_labels(columns = c(AIPW,HDEL,KER,MI2,MIB2,mice,mix))
  )
tab_mm_rmse6
```

RMSE: Backward elimination

```
# 2nd approach with interactions and backward selection; results are shown
# RMSE
dta_rmse7 <- c()
for (j in c("MCAR", "MAR", "MNAR")) {
    
      form <- paste0("RMSE", "*100", " ~ Method2*N + Method2*p + Method2*r + Method2*AUC_0 + Method2*pm + (1|scenario)")
      mm <- lmer(as.formula(form), data=data1[which(data1$mech==j),])
      backward <- lmerTest::step(mm)
      final <- lmerTest::get_model(backward)
            
      dta <- cbind(coef(summary(final))[,"Estimate"],
                           coef(summary(final))[,"Pr(>|t|)"])
      colnames(dta) <- c("Estimate", "p-value")
      dta <- as.data.frame(t(dta))
      dta <- dta %>%
              tibble::rownames_to_column("Result") %>%
              mutate(
                Mechanism = j
              ) %>%
              relocate(any_of(c("Mechanism", "Result")), .before="(Intercept)") %>%
              rename(
                  AIPW = Method2AIPW,
                  HDEL = Method2HDEL,
                  KER = Method2KER,
                  MI2 = Method2MI2,
                  MIB2 = Method2MIB2,
                  mice = Method2mice,
                  mix = Method2mix
              )
      dta_rmse7 <- dta_rmse7 %>%
        bind_rows(dta)
      
}

tabno <- tabno+1
tab_mm_rmse7 <- dta_rmse7 %>%
  arrange(Mechanism) %>%
  gt(groupname_col = c("Mechanism")) %>%
  row_group_order(groups=c("MCAR", "MAR", "MNAR")) %>%
  fmt_number(
    columns = where(is.numeric),
    rows = Result=="Estimate",
    decimals = 2
  ) %>%
  fmt_number(
    columns = where(is.numeric),
    rows = Result=="p-value",
    decimals = 3
  ) %>%
  sub_missing(
  columns = everything(),
  rows = everything(),
  missing_text = ""
  ) %>%
  tab_header(
    title = paste0("Table ", tabno, ". Differences in RMSE by method and simulation parameter stratified by missingness mechanism with backward elimination")
  ) %>%
  tab_footnote(
    footnote = "CCA as refernce group",
    locations = cells_column_labels(columns = c(AIPW,HDEL,KER,MI2,MIB2,mice,mix))
  )
tab_mm_rmse7
```

|  |  |  |  |  |  |  |  |  |  |  |  |  |  |  |  |  |  |  |  |  |  |  |  |  |  |  |  |  |  |  |  |  |  |  |  |  |  |  |  |  |  |  |  |  |  |  |  |  |
| --- | --- | --- | --- | --- | --- | --- | --- | --- | --- | --- | --- | --- | --- | --- | --- | --- | --- | --- | --- | --- | --- | --- | --- | --- | --- | --- | --- | --- | --- | --- | --- | --- | --- | --- | --- | --- | --- | --- | --- | --- | --- | --- | --- | --- | --- | --- | --- | --- |
| Table 10. Differences in RMSE by method and simulation parameter stratified by missingness mechanism with backward elimination | | | | | | | | | | | | | | | | | | | | | | | | | | | | | | | | | | | | | | | | | | | | | | | | |
| Result | (Intercept) | AIPW1 | HDEL1 | KER1 | MI21 | MIB21 | mice1 | mix1 | N | p | r | AUC\_0 | pm | Method2AIPW:N | Method2HDEL:N | Method2KER:N | Method2MI2:N | Method2MIB2:N | Method2mice:N | Method2mix:N | Method2AIPW:p | Method2HDEL:p | Method2KER:p | Method2MI2:p | Method2MIB2:p | Method2mice:p | Method2mix:p | Method2AIPW:r | Method2HDEL:r | Method2KER:r | Method2MI2:r | Method2MIB2:r | Method2mice:r | Method2mix:r | Method2AIPW:AUC\_0 | Method2HDEL:AUC\_0 | Method2KER:AUC\_0 | Method2MI2:AUC\_0 | Method2MIB2:AUC\_0 | Method2mice:AUC\_0 | Method2mix:AUC\_0 | Method2AIPW:pm | Method2HDEL:pm | Method2KER:pm | Method2MI2:pm | Method2MIB2:pm | Method2mice:pm | Method2mix:pm |
| MCAR | | | | | | | | | | | | | | | | | | | | | | | | | | | | | | | | | | | | | | | | | | | | | | | | |
| --- | --- | --- | --- | --- | --- | --- | --- | --- | --- | --- | --- | --- | --- | --- | --- | --- | --- | --- | --- | --- | --- | --- | --- | --- | --- | --- | --- | --- | --- | --- | --- | --- | --- | --- | --- | --- | --- | --- | --- | --- | --- | --- | --- | --- | --- | --- | --- | --- |
| Estimate | 14.52 | −0.44 | 1.05 | −0.23 | 0.07 | −0.39 | −1.32 | −1.56 | 0.00 | −4.87 | −0.02 | −9.53 | 2.86 | 0.00 | 0.00 | 0.00 | 0.00 | 0.00 | 0.00 | 0.00 | −0.05 | −0.41 | −0.32 | −0.03 | 0.19 | 0.53 | 0.49 | −0.87 | 0.00 | −0.51 | −0.63 | −0.52 | −0.86 | −0.61 | 1.07 | −0.86 | 0.67 | 0.33 | 0.87 | 1.78 | 1.90 | −0.71 | 0.92 | −0.28 | −0.28 | −0.51 | −0.93 | −1.08 |
| p-value | 0.000 | 0.057 | 0.000 | 0.317 | 0.754 | 0.092 | 0.000 | 0.000 | 0.000 | 0.000 | 0.929 | 0.000 | 0.000 | 0.020 | 0.000 | 0.434 | 0.695 | 0.285 | 0.000 | 0.000 | 0.688 | 0.003 | 0.018 | 0.835 | 0.174 | 0.000 | 0.000 | 0.000 | 0.954 | 0.000 | 0.000 | 0.000 | 0.000 | 0.000 | 0.000 | 0.001 | 0.010 | 0.212 | 0.001 | 0.000 | 0.000 | 0.000 | 0.000 | 0.042 | 0.040 | 0.000 | 0.000 | 0.000 |
| MAR | | | | | | | | | | | | | | | | | | | | | | | | | | | | | | | | | | | | | | | | | | | | | | | | |
| Estimate | 16.99 | −0.33 | 2.18 | 1.25 | −0.86 | −2.45 | −3.44 | −4.68 | 0.00 | −7.53 | −0.20 | −11.06 | 4.16 | 0.00 | 0.00 | 0.00 | 0.00 | 0.00 | 0.00 | 0.00 | 0.26 | −1.37 | −1.81 | −0.31 | 0.48 | 1.03 | 0.85 | −1.73 | −0.05 | −0.63 | −0.87 | −0.70 | −1.54 | −0.63 | 1.31 | −1.61 | −0.21 | 1.73 | 3.22 | 4.46 | 5.50 | 0.04 | 1.27 | 1.98 | 0.79 | 0.16 | −0.57 | −0.63 |
| p-value | 0.000 | 0.595 | 0.000 | 0.043 | 0.161 | 0.000 | 0.000 | 0.000 | 0.000 | 0.000 | 0.572 | 0.000 | 0.000 | 0.101 | 0.000 | 0.000 | 0.001 | 0.328 | 0.329 | 0.749 | 0.485 | 0.000 | 0.000 | 0.402 | 0.194 | 0.005 | 0.020 | 0.000 | 0.798 | 0.003 | 0.000 | 0.001 | 0.000 | 0.003 | 0.062 | 0.022 | 0.762 | 0.014 | 0.000 | 0.000 | 0.000 | 0.914 | 0.001 | 0.000 | 0.031 | 0.654 | 0.119 | 0.083 |
| MNAR | | | | | | | | | | | | | | | | | | | | | | | | | | | | | | | | | | | | | | | | | | | | | | | | |
| Estimate | 13.52 | 0.85 | 1.46 | 1.61 | 0.59 | −0.59 | −1.12 | −2.11 | −0.01 | −7.22 | −0.02 | −7.03 | 5.01 | 0.00 | 0.00 | 0.00 | 0.00 | 0.00 | 0.00 | 0.00 | 0.22 | −1.36 | −1.58 | −0.45 | 0.21 | 0.67 | 0.39 | −1.90 | 0.02 | −0.72 | −1.08 | −0.89 | −2.02 | −1.16 | 0.07 | −0.77 | −0.62 | 0.23 | 1.30 | 2.19 | 2.97 | −1.09 | 1.24 | 0.53 | −0.20 | −0.71 | −1.12 | −1.14 |
| p-value | 0.000 | 0.117 | 0.007 | 0.003 | 0.271 | 0.275 | 0.039 | 0.000 | 0.000 | 0.000 | 0.942 | 0.000 | 0.000 | 0.389 | 0.000 | 0.000 | 0.001 | 0.251 | 0.435 | 0.690 | 0.496 | 0.000 | 0.000 | 0.164 | 0.523 | 0.037 | 0.228 | 0.000 | 0.917 | 0.000 | 0.000 | 0.000 | 0.000 | 0.000 | 0.915 | 0.215 | 0.315 | 0.704 | 0.036 | 0.000 | 0.000 | 0.001 | 0.000 | 0.102 | 0.531 | 0.027 | 0.001 | 0.000 |
|  |  |  |  |  |  |  |  |  |  |  |  |  |  |  |  |  |  |  |  |  |  |  |  |  |  |  |  |  |  |  |  |  |  |  |  |  |  |  |  |  |  |  |  |  |  |  |  |  |
| --- | --- | --- | --- | --- | --- | --- | --- | --- | --- | --- | --- | --- | --- | --- | --- | --- | --- | --- | --- | --- | --- | --- | --- | --- | --- | --- | --- | --- | --- | --- | --- | --- | --- | --- | --- | --- | --- | --- | --- | --- | --- | --- | --- | --- | --- | --- | --- | --- |
| 1 CCA as refernce group | | | | | | | | | | | | | | | | | | | | | | | | | | | | | | | | | | | | | | | | | | | | | | | | |

# 5 References

Bianco AM, Boente G, González–Manteiga W, Pérez–González A.
Estimators for ROC curves with missing biomarkers values and informative
covariates. Statistical Methods & Applications. 2023.

DeLong ER, DeLong DM, Clarke-Pearson DL. Comparing the areas under
two or more correlated receiver operating characteristic curves: a
nonparametric approach. Biometrics. 1988;44(3):837-45.

Cheng W, Tang N. Smoothed empirical likelihood inference for ROC
curve in the presence of missing biomarker values. Biom J.
2020;62(4):1038-59.

Hanley JA, McNeil BJ. The meaning and use of the area under a
receiver operating characteristic (ROC) curve. Radiology.
1982;143(1):29-36.

Long Q, Zhang X, Hsu C-H. Nonparametric multiple imputation for
receiver operating characteristics analysis when some biomarker values
are missing at random. Stat Med. 2011a;30(26):3149-61.

Long Q, Zhang X, Johnson BA. Robust estimation of area under ROC
curve using auxiliary variables in the presence of missing biomarker
values. Biometrics. 2011b;67(2):559-67.

Pulit M. A new method of kernel-smoothing estimation of the ROC
curve. Metrika. 2016;79(5):603-34.

Robin X, Turck N, Hainard A, Tiberti N, Lisacek F, Sanchez J, et
al. pROC: an open-source package for R and S+ to analyze and compare ROC
curves. BMC Bioinformatics. 2011(12):77.

Rubin DB. Multiple Imputation for Nonresponse in Surveys. New York:
John Wiley & Sons, Inc.; 1987.

Schafer J. mix: Estimation/Multiple Imputation for Mixed Categorical
and Continuous Data. R package version 10-11. 2022.

Schafer JL, Graham JW. Missing data: our view of the state of the
art. Psychological methods. 2002;7(2):147.

van Buuren S. Flexible Imputation of Missing Data. 2nd ed. New York:
Chapman and Hall/CRC; 2018.

van Buuren S, Groothuis-Oudshoorn K. mice: Multivariate Imputation by
Chained Equations in R. Journal of Statistical Software. 2011;45(3):1 -
67.

van Smeden M, Moons KG, de Groot JA, et al. Sample size for binary
logistic prediction models: Beyond events per variable criteria. Stat
Methods Med Res. 2019;28(8):2455-2474. doi:10.1177/0962280218784726

Wang B, Qin G. Imputation-based empirical likelihood inference for
the area under the ROC curve with missing data. Stat Interface.
2012;5(3):319-29.

Wang B, Qin G. Empirical likelihood-based confidence intervals for
the sensitivity of a continuous-scale diagnostic test with missing data.
Commun Stat Theory Methods. 2014;43(15):3248-68.

# 6 Session info

```
sessioninfo::session_info()
```

```
## ─ Session info ───────────────────────────────────────────────────────────────
##  setting  value
##  version  R version 4.4.1 (2024-06-14 ucrt)
##  os       Windows 10 x64 (build 19045)
##  system   x86_64, mingw32
##  ui       RTerm
##  language (EN)
##  collate  German_Germany.utf8
##  ctype    German_Germany.utf8
##  tz       Europe/Berlin
##  date     2025-04-25
##  pandoc   3.2 @ C:/Program Files/RStudio/resources/app/bin/quarto/bin/tools/ (via rmarkdown)
## 
## ─ Packages ───────────────────────────────────────────────────────────────────
##  package      * version    date (UTC) lib source
##  abind          1.4-8      2024-09-12 [1] CRAN (R 4.4.1)
##  arsenal      * 3.6.3      2021-06-04 [1] CRAN (R 4.4.1)
##  backports      1.5.0      2024-05-23 [1] CRAN (R 4.4.0)
##  boot           1.3-30     2024-02-26 [2] CRAN (R 4.4.1)
##  broom          1.0.6      2024-05-17 [1] CRAN (R 4.4.1)
##  bslib          0.8.0      2024-07-29 [1] CRAN (R 4.4.1)
##  cachem         1.1.0      2024-05-16 [1] CRAN (R 4.4.1)
##  car            3.1-2      2023-03-30 [1] CRAN (R 4.4.1)
##  carData        3.0-5      2022-01-06 [1] CRAN (R 4.4.1)
##  cards          0.2.2      2024-09-02 [1] CRAN (R 4.4.1)
##  checkmate      2.3.2      2024-07-29 [1] CRAN (R 4.4.1)
##  cli            3.6.3      2024-06-21 [1] CRAN (R 4.4.1)
##  colorspace     2.1-1      2024-07-26 [1] CRAN (R 4.4.1)
##  commonmark     1.9.1      2024-01-30 [1] CRAN (R 4.4.1)
##  digest         0.6.37     2024-08-19 [1] CRAN (R 4.4.1)
##  dplyr        * 1.1.4      2023-11-17 [1] CRAN (R 4.4.1)
##  evaluate       1.0.3      2025-01-10 [1] CRAN (R 4.4.3)
##  fansi          1.0.6      2023-12-08 [1] CRAN (R 4.4.1)
##  farver         2.1.2      2024-05-13 [1] CRAN (R 4.4.1)
##  fastmap        1.2.0      2024-05-15 [1] CRAN (R 4.4.1)
##  generics       0.1.3      2022-07-05 [1] CRAN (R 4.4.1)
##  ggplot2      * 3.5.1      2024-04-23 [1] CRAN (R 4.4.1)
##  ggpubr       * 0.6.0      2023-02-10 [1] CRAN (R 4.4.1)
##  ggridges       0.5.6      2024-01-23 [1] CRAN (R 4.4.1)
##  ggsignif       0.6.4      2022-10-13 [1] CRAN (R 4.4.1)
##  glue           1.7.0      2024-01-09 [1] CRAN (R 4.4.1)
##  gt           * 0.11.0     2024-07-09 [1] CRAN (R 4.4.1)
##  gtable         0.3.5      2024-04-22 [1] CRAN (R 4.4.1)
##  gtsummary    * 2.0.2      2024-09-05 [1] CRAN (R 4.4.1)
##  highr          0.11       2024-05-26 [1] CRAN (R 4.4.1)
##  htmltools      0.5.8.1    2024-04-04 [1] CRAN (R 4.4.1)
##  insight        0.20.4     2024-09-01 [1] CRAN (R 4.4.1)
##  jquerylib      0.1.4      2021-04-26 [1] CRAN (R 4.4.1)
##  jsonlite       1.8.8      2023-12-04 [1] CRAN (R 4.4.1)
##  knitr          1.48       2024-07-07 [1] CRAN (R 4.4.1)
##  labeling       0.4.3      2023-08-29 [1] CRAN (R 4.4.0)
##  lattice        0.22-6     2024-03-20 [2] CRAN (R 4.4.1)
##  lifecycle      1.0.4      2023-11-07 [1] CRAN (R 4.4.1)
##  lme4         * 1.1-35.5   2024-07-03 [1] CRAN (R 4.4.1)
##  lmerTest     * 3.1-3      2020-10-23 [1] CRAN (R 4.4.1)
##  magrittr       2.0.3      2022-03-30 [1] CRAN (R 4.4.1)
##  markdown       1.13       2024-06-04 [1] CRAN (R 4.4.1)
##  MASS           7.3-60.2   2024-04-26 [2] CRAN (R 4.4.1)
##  Matrix       * 1.7-0      2024-04-26 [2] CRAN (R 4.4.1)
##  minqa          1.2.8      2024-08-17 [1] CRAN (R 4.4.1)
##  munsell        0.5.1      2024-04-01 [1] CRAN (R 4.4.1)
##  nlme           3.1-164    2023-11-27 [2] CRAN (R 4.4.1)
##  nloptr         2.1.1      2024-06-25 [1] CRAN (R 4.4.1)
##  numDeriv       2016.8-1.1 2019-06-06 [1] CRAN (R 4.4.0)
##  pillar         1.9.0      2023-03-22 [1] CRAN (R 4.4.1)
##  pkgconfig      2.0.3      2019-09-22 [1] CRAN (R 4.4.1)
##  purrr          1.0.2      2023-08-10 [1] CRAN (R 4.4.1)
##  R6             2.5.1      2021-08-19 [1] CRAN (R 4.4.1)
##  RColorBrewer * 1.1-3      2022-04-03 [1] CRAN (R 4.4.0)
##  Rcpp           1.0.13     2024-07-17 [1] CRAN (R 4.4.1)
##  rlang          1.1.4      2024-06-04 [1] CRAN (R 4.4.1)
##  rmarkdown      2.28       2024-08-17 [1] CRAN (R 4.4.1)
##  rprojroot      2.0.4      2023-11-05 [1] CRAN (R 4.4.2)
##  rsimsum      * 0.13.0     2024-03-03 [1] CRAN (R 4.4.1)
##  rstatix        0.7.2      2023-02-01 [1] CRAN (R 4.4.1)
##  rstudioapi     0.16.0     2024-03-24 [1] CRAN (R 4.4.1)
##  sass           0.4.9      2024-03-15 [1] CRAN (R 4.4.1)
##  scales         1.3.0      2023-11-28 [1] CRAN (R 4.4.1)
##  sessioninfo    1.2.2      2021-12-06 [1] CRAN (R 4.4.1)
##  sjlabelled     1.2.0      2022-04-10 [1] CRAN (R 4.4.1)
##  tibble         3.2.1      2023-03-20 [1] CRAN (R 4.4.1)
##  tidyr          1.3.1      2024-01-24 [1] CRAN (R 4.4.1)
##  tidyselect     1.2.1      2024-03-11 [1] CRAN (R 4.4.1)
##  utf8           1.2.4      2023-10-22 [1] CRAN (R 4.4.1)
##  vctrs          0.6.5      2023-12-01 [1] CRAN (R 4.4.1)
##  withr          3.0.2      2024-10-28 [1] CRAN (R 4.4.3)
##  writexl      * 1.5.0      2024-02-09 [1] CRAN (R 4.4.1)
##  xfun           0.47       2024-08-17 [1] CRAN (R 4.4.1)
##  xml2           1.3.6      2023-12-04 [1] CRAN (R 4.4.1)
##  yaml           2.3.10     2024-07-26 [1] CRAN (R 4.4.1)
## 
##  [1] C:/Users/stahlmann/AppData/Local/R/win-library/4.4
##  [2] C:/Program Files/R/R-4.4.1/library
## 
## ──────────────────────────────────────────────────────────────────────────────
```
